# Genome-wide identification and analysis of the *ALTERNATIVE OXIDASE* gene family in diploid and hexaploid wheat

**DOI:** 10.1371/journal.pone.0201439

**Published:** 2018-08-03

**Authors:** Rhoda A. T. Brew-Appiah, Zara B. York, Vandhana Krishnan, Eric H. Roalson, Karen A. Sanguinet

**Affiliations:** 1 Department of Crop and Soil Sciences, Washington State University, Pullman, Washington, United States of America; 2 Stanford Center for Genomics and Personalized Medicine, Department of Genetics, Stanford University, Stanford, United States of America; 3 School of Biological Sciences, Washington State University, Pullman, Washington, United States of America; Institute of Genetics and Developmental Biology Chinese Academy of Sciences, CHINA

## Abstract

A comprehensive understanding of wheat responses to environmental stress will contribute to the long-term goal of feeding the planet. *ALERNATIVE OXIDASE* (*AOX*) genes encode proteins involved in a bypass of the electron transport chain and are also known to be involved in stress tolerance in multiple species. Here, we report the identification and characterization of the *AOX* gene family in diploid and hexaploid wheat. Four genes each were found in the diploid ancestors *Triticum urartu*, and *Aegilops tauschii*, and three in *Aegilops speltoides*. In hexaploid wheat (*Triticum aestivum*), 20 genes were identified, some with multiple splice variants, corresponding to a total of 24 proteins for those with observed transcription and translation. These proteins were classified as AOX1a, AOX1c, AOX1e or AOX1d via phylogenetic analysis. Proteins lacking most or all signature AOX motifs were assigned to putative regulatory roles. Analysis of protein-targeting sequences suggests mixed localization to the mitochondria and other organelles. In comparison to the most studied AOX from *Trypanosoma brucei*, there were amino acid substitutions at critical functional domains indicating possible role divergence in wheat or grasses in general. In hexaploid wheat, *AOX* genes were expressed at specific developmental stages as well as in response to both biotic and abiotic stresses such as fungal pathogens, heat and drought. These *AOX* expression patterns suggest a highly regulated and diverse transcription and expression system. The insights gained provide a framework for the continued and expanded study of *AOX* genes in wheat for stress tolerance through breeding new varieties, as well as resistance to AOX-targeted herbicides, all of which can ultimately be used synergistically to improve crop yield.

## Introduction

Bread wheat (*Triticum aestivum*) feeds a significant portion of the world’s population and there has been substantial progress on boosting supply to meet the global increase in demand [1, 2]. While worldwide production and yield of wheat have gradually increased over the past decade (http://statistics.amis-outlook.org/data/index.html), these gains may be offset by predicted harvest losses to global climate change and a reduction in arable land [3, 4]. In addition to the decline in production quantity, the impending and growing environmental stress expected to cause a deterioration in wheat quality [5–7].

Stress response pathways in plants trigger changes in hormone biosynthesis, transcriptional activity and metabolic responses that are crucial for maintaining structural and functional integrity [8, 9]. One key biological component of plant metabolism and stress responses is the mitochondrion, which is the site of an ATP-generative electron shuffle involving multiple cytochrome oxidase and dehydrogenase complexes. Upon stress perception, electrons can also be shunted to an alternative oxidase, which is proposed to dissipate the energy as heat, reduces oxygen to water and limit reactive oxygen species (ROS) production [10–12]. In fact, thermogenic plants use ALTERNATIVE OXIDASE (AOX) to produce heat during respiration to facilitate pollen germination and to volatilize pollination attractants [13]. The *AOX* gene was first cloned from the thermogenic plant *Sauromatum guttatum* [14, 15]. Antibodies for this protein cross-reacted with similar proteins from non-thermogenic plants and this facilitated the study of these terminal oxidases in other species. With the availability of sequenced genomes and numerous molecular techniques, *AOX* genes have been identified and in some cases, functionally characterized in both dicots and monocots such as *Arabidopsis thaliana*, tobacco, carrot, mango, stone pine, cowpea, chickpea, barley, rice and maize [16–27]. *AOX* genes fall into two discrete subfamilies, Type 1 and Type 2. The former is present in both monocot and dicot species while the latter has so far only been found in dicots but is purported to have existed in ancient monocots [16, 17, 28]. *AOX1* (Type 1 *AOX*) genes are very responsive to stresses as well as irregularities in respiratory metabolism [29–31]. The *AOX2* (Type 2 *AOX*) genes control developmental processes such as germination, fertility and vegetative growth, but there is also some evidence for a role in stress response [32–35].

The initial cloning of two *AOX* genes from wheat [36] spurred a considerable amount of biochemical work and some expression studies indicating they are involved in numerous developmental processes as well as responses to stress [37–51]. On the genomic level, the number and spatiotemporal expression patterns of *AOX* genes has remained unclear in wheat. The availability of the wheat genome now makes it possible to conduct a genome-wide examination of the *AOX* family in the hexaploid and ancestral diploid species of this important monocot [52–54]. The current study investigated and identified the *AOX* gene family in hexaploid wheat and its diploid ancestors in the A (*Triticum urartu*), B (*Aegilops speltoides*) and D (*Aegilops tauschii*) subgenomes. Using multiple *in silico* resources and the latest transcriptome database [55], features such as phylogenetic evolutionary relationships, chromosomal locations, gene structures, promoter cis-elements, conserved motifs, subcellular localization and expression patterns were evaluated. Our findings provide a better understanding of the wheat *AOX* family members, promote our understanding of the regulation of this gene family and lay the groundwork for future study of *AOX* in wheat.

## Materials and methods

### Identification of the *AOX* gene family in wheat

The amino acid sequences of *Waox1a* and *Waox1c* (Genbank ID BAB88645.1 and BAB88646.1) were used in a BLASTP search (E-value threshold 1 e-1) on Ensembl Plants (http://plants.ensembl.org/index.html) and the International Wheat Genome Sequencing Consortium (IWGSC) URGI portal (https://urgi.versailles.inra.fr/blast/) in May 2018 [56, 57]. Concurrently the coding sequences of *Waox1a* and *Waox1c* (Genbank ID AB078882.1 and AB078883.1) were used in a BLASTN search (E-value threshold 10) on the aforementioned databases. Nucleotide sequences and unique protein IDs of matching sequences were obtained for *T*. *aestivum* as well as *T*. *urartu* (A subgenome), *A*. *speltoides* (B subgenome) and *A*. *tauschii* (D subgenome). The output from Ensembl Plants was classified by the databank as either high-confidence indicating that the data was fully supported by PacBio transcript sequencing as well as RNA-seq data, or low-confidence for sequences which had partial or no transcriptome data support (http://plants.ensembl.org/Triticum_aestivum/Info/Annotation/ - genebuild).

### Phylogeny

The amino acid sequences of the AOX proteins from wheat, barley, *Brachypodium distachyon*, rice, and maize obtained from Ensembl Plants (http://plants.ensembl.org/index.html) [56], Phytozome (https://phytozome.jgi.doe.gov/pz/portal.html) [58] and the National Center for Biotechnology Information (NCBI) (https://www.ncbi.nlm.nih.gov/) [59] were aligned with representative sequences from other monocots (*Anasus comosus*, *Asparagus officinalis*, *Musa acuminata*, *Oropetalum thomaeum*, *Panicum virgatum*, *Spirodela polyrhiza*, *Symplocarpus renifolius*, *Zostera marina*) using MUSCLE [60]. The amino acid alignment was analyzed using maximum likelihood (ML) with RAxML (7.7.1) [61] implementing the GTR-Gamma model and JTT substitution matrix with 100 bootstrap replicates. Bayesian inference (BI) analyses implementing a mixed AA model prior in MrBayes 3.2.2 [62] were run over 50 million generations with the first 25% removed for burn-in and assessed for convergence and stationarity using average standard deviation of split frequencies, potential scale reduction factor (PSRF) values approaching 1.0, and a large effective sample size assessed in Tracer v.1.4.1 [63].

### Gene structure and protein analyses

The coding sequence of each *AOX* gene was aligned with the genomic sequence in order to delineate the intron/exon boundaries using the Gene Structure Display Server program (http://gsds.cbi.pku.edu.cn/) [64]. Alignment of the protein sequences to search for relevant motifs and residues was done using Clustal Omega (https://www.ebi.ac.uk/Tools/msa/clustalo/) [65] and the results used in subclassification via a protocol described by previous researchers [16, 66]. When all four motifs were present in high-confidence protein sequences, it was designated an AOX. Furthermore, when a motif was absent, the protein was given the suffix “-like”, and when all motifs were missing the corresponding gene was proposed to have a putative regulatory function and given the prefix “reg”. The low-confidence proteins with all motifs were given the prefix “put” (putative), those missing a motif were given the prefix “put” and the suffix “-like” and those with no motifs received the prefix “put.reg”. In some cases, the proteins were given the prefix “ne” for “non-expressed” to indicate a complete lack of transcript data but a similarity to a particular class of AOX proteins. Where the non-expressed protein bore no resemblance to a particular subclassification, the suffix symbol “•” was added as a stand-in for a future subclassification pending the availability of transcript data. For all hexaploid proteins, an indication was made of the chromosomal location of the corresponding gene provided by the Ensembl Plants database. The genes with splice variants were given the alphanumerical suffix “sv” followed by a number. In order to determine the orientation of genes on the same chromosomal arm, the sequences we obtained were aligned to the respective arms via SnapGene. A Needleman-Wunsch alignment was performed to determine transcript and protein percent identities using the Global Align program from NCBI with default parameters. The subcellular localization was predicted using TargetP (http://www.cbs.dtu.dk/services/TargetP/) [67] and putative protein modification sites were predicted using the Plant Protein Phosphorylation Database (http://www.p3db.org/index.php) and Musite (http://musite.net/) [68, 69] with a threshold score of 0.5. CpG islands in the gene body were determined using Cpgplot available in the European Molecular Biology Open Software Suite (EMBOSS) (http://www.bioinformatics.nl/cgi-bin/emboss/cpgplot) [70].

### Promoter analyses

To identify cis-elements needed for various developmental cell functions as well as binding motifs for known regulators of *AOX* expression [71], 1500 bp upstream of the translation start site of the wheat *AOX* sequences was analyzed using plantCARE (http://bioinformatics.psb.ugent.be/webtools/plantcare/html/), PlantPan2.0 (http://plantpan2.itps.ncku.edu.tw/index.html) and the Plant Transcription Factor Database (http://planttfdb.cbi.pku.edu.cn/) [72–74]. CpG islands in the promoter region were found using Cpgplot available in EMBOSS (http://www.bioinformatics.nl/cgi-bin/emboss/cpgplot) [70].

### Molecular modeling

The three-dimensional structures of the wheat AOX proteins were obtained via modeling to solved protein structures, using the Protein Homology/Analogy Recognition Engine version 2.0 server (Phyre2) [75]. This server was also used to predict the transmembrane topology of AOX proteins in the diploid and hexaploid and wheat species. Modeling and residues involved in the diiron center of the AOX proteins were visualized using Chimera (http://www.rbvi.ucsf.edu/chimera/) [76].

### RNA expression analyses

The expression patterns of the hexaploid wheat *AOX* genes were obtained from the publicly available RNA-seq data from the wheat variety Chinese Spring on expVIP (http://www.wheat-expression.com/) [55]. The relative transcript abundance data from the seedling, vegetative and reproductive stages of development and over multiple tissue types were used to generate heat maps in order to visualize the similarities and differences in the *TaAOX* family. As previously described [77], the expression ratio for a given treatment compared to the control was used to generate heat maps for the transcripts under biotic and abiotic stress (https://www.rdocumentation.org/packages/gplots/versions/3.0.1/topics/heatmap.2).

## Results and discussion

### Identification and classification of the *AOX* gene family in wheat

**Fig 1:**
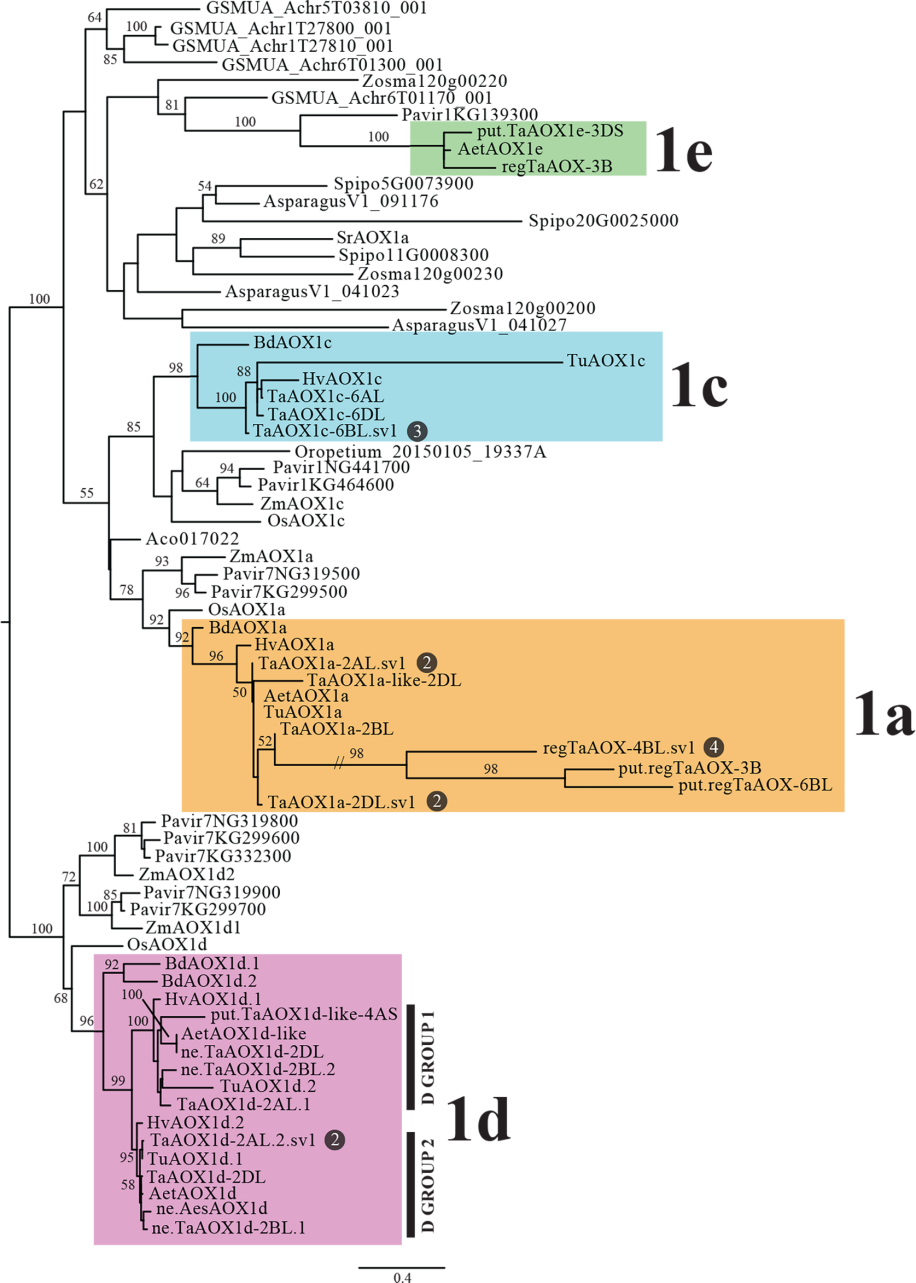
Maximum likelihood (ML) phylogeny of AOX. Numbers on branches are ML bootstrap percentages. The number of splice variant isomers for a protein are denoted in the dark gray circle when applicable. Colored boxes distinguish the different AOX clades.

**Fig 2:**
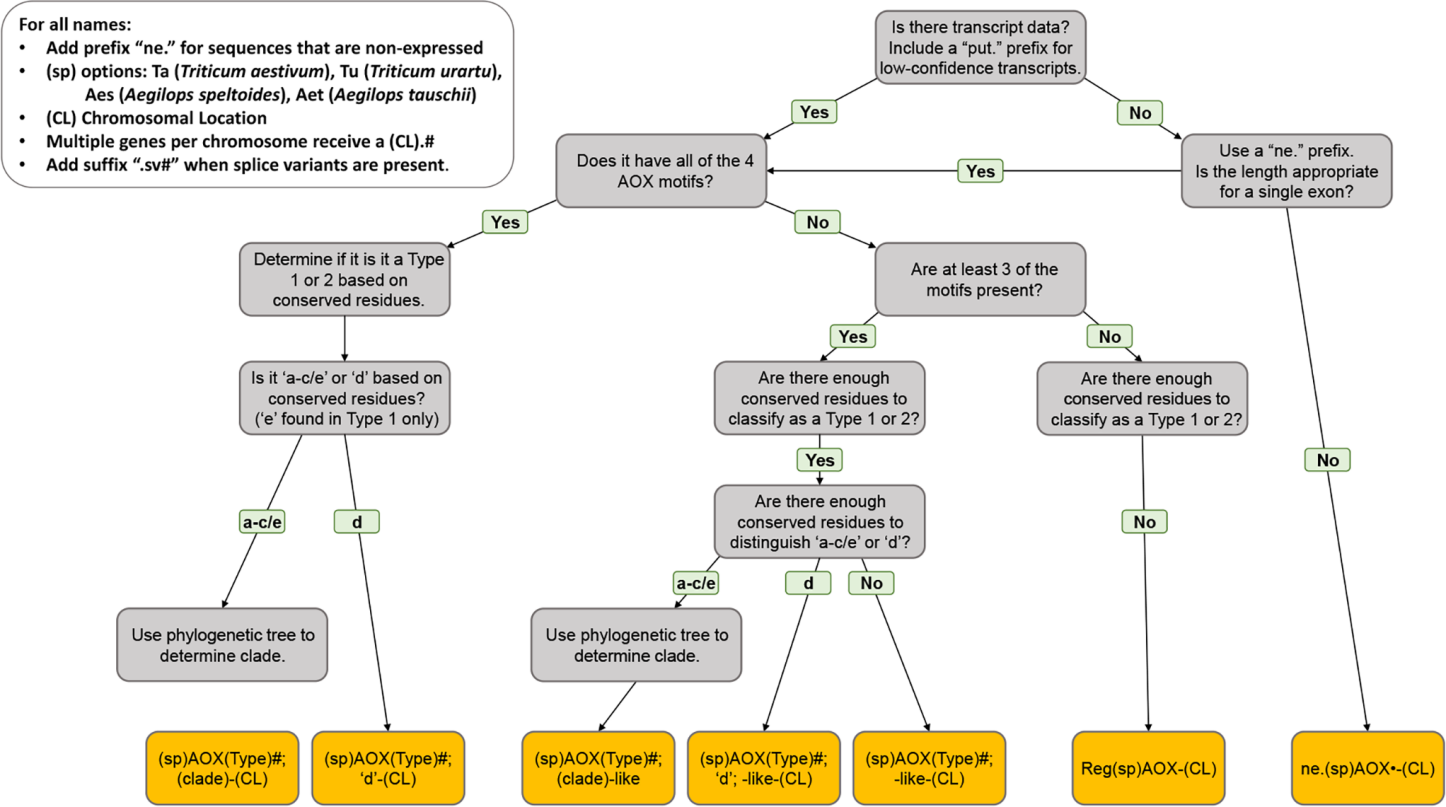
Summary of protocol for AOX protein classification in wheat.

Two *AOX* coding sequences were previously cloned from wheat and named *Waox1a* and *Waox1c* [36]. These sequences as well as the Waox1a and Waox1c protein sequences were used in BLAST searches in order to identify additional wheat *AOX* genes and proteins. The obtained nucleotide sequences broadly fell into three groups, high-confidence where there was ample transcriptome and RNA-seq data, low-confidence where there was partial or no transcriptome data, and non-expressed where there was nucleotide similarity but no transcript data (Table 1). Based on previous work, all the corresponding proteins of *AOX* genes show a trend of unique motifs and residues known to dictate functionality [16, 66]. Therefore, in order to classify these genes and the corresponding proteins, the protein sequences were used in phylogenetic analysis resulting in the categorization into the clades AOX1a, AOX1c or AOX1e or AOX1d (Figs 1, S1 and S2; S1 Table). The outcomes were also supported by the specific AOX motifs and subclassification residues peculiar to each clade. Subsequently, the proteins and corresponding genes were named with additional indicators of chromosomal locations where necessary (Figs 1 and 2 and Tables 1 and 2 and S1 and S3 Figs and S2 Table). It must be noted that the residues of the wheat AOX proteins deviated in some cases from the observed residues thought to be highly conserved in the clades in other monocot species (Table 2) [16].

**Table 1:** Summary of all accession numbers of the *AOX* gene family in wheat done via a BLASTN search.

**Promoter**	**Classification**	**Ensembl Plants Gene ID**
*TaAOX1a-2AL*	*TaAOX1a-2AL*.*sv1*	TRIAE_CS42_2AL_TGACv1_093624_AA0283900.1
	*TaAOX1a-2AL*.*sv2*	TRIAE_CS42_2AL_TGACv1_093624_AA0283900.2
*TaAOX1a-2BL*	*TaAOX1a-2BL*	TRIAE_CS42_2BL_TGACv1_132767_AA0439680.1
*TaAOX1a-2DL*	*TaAOX1a-2DL*.*sv1*	TRIAE_CS42_2DL_TGACv1_159044_AA0531270.1
	*TaAOX1a-2DL*.*sv2*	TRIAE_CS42_2DL_TGACv1_159044_AA0531270.2
*TaAOX1a-like-2DL*	*TaAOX1a-like-2DL*	TRIAE_CS42_2DL_TGACv1_160367_AA0549780.1
*regTaAOX-4BL*	*regTaAOX-4BL*.*sv1*	TRIAE_CS42_4BL_TGACv1_321481_AA1061160.1
	*regTaAOX-4BL*.*sv2*	TRIAE_CS42_4BL_TGACv1_321481_AA1061160.2
	*regTaAOX-4BL*.*sv3*	TRIAE_CS42_4BL_TGACv1_321481_AA1061160.3
	*regTaAOX-4BL*.*sv4*	TRIAE_CS42_4BL_TGACv1_321481_AA1061160.4
*put*.*regTaAOX-3B*	*put*.*regTaAOX-3B*	TRIAE_CS42_3B_TGACv1_221271_AA0735840
*put*.*regTaAOX-6BL*	*put*.*regTaAOX-6BL*	TRIAE_CS42_6BL_TGACv1_499339_AA1578450
*TaAOX1c-6AL*	*TaAOX1c-6AL*	TRIAE_CS42_6AL_TGACv1_471250_AA1505530.1
*TaAOX1c-6BL*	*TaAOX1c-6BL*.*sv1*	TRIAE_CS42_6BL_TGACv1_499881_AA1593950.1
	*TaAOX1c-6BL*.*sv2*	TRIAE_CS42_6BL_TGACv1_499881_AA1593950.2
	*TaAOX1c-6BL*.*sv3*	TRIAE_CS42_6BL_TGACv1_499881_AA1593950.3
*TaAOX1c-6DL*	*TaAOX1c-6DL*	TRIAE_CS42_6DL_TGACv1_528632_AA1715280.1
*regTaAOX-3B*	*regTaAOX-3B*	TRIAE_CS42_3B_TGACv1_221946_AA0753740.1
*put*.*TaAOX1e-3DS*	*put*.*TaAOX1e-3DS*	TRIAE_CS42_3DS_TGACv1_271978_AA0912170
*TaAOX1d-2AL*.*1*	*TaAOX1d-2AL*.*1*	TRIAE_CS42_2AL_TGACv1_094717_AA0302070.1
*TaAOX1d-2AL*.*2*	*TaAOX1d-2AL*.*2*.*sv1*	TRIAE_CS42_2AL_TGACv1_093545_AA0282360.1
	*TaAOX1d-2AL*.*2*.*sv2*	TRIAE_CS42_2AL_TGACv1_093545_AA0282360.2
*TaAOX1d-2DL*	*TaAOX1d-2DL*	TRIAE_CS42_2DL_TGACv1_162315_AA0562440.1
*put*.*TaAOX1d-like-4AS*	*put*.*TaAOX1d-like-4AS*	TRIAE_CS42_4AS_TGACv1_308389_AA1027660
*TuAOX1d*.1*	*TuAOX1d*.1*	TRIUR3_12374
*TuAOX1d*.2*	*TuAOX1d*.2*	TRIUR3_19476
TuAOX1c*	TuAOX1c*	TRIUR3_08189
TuAOX1a*	TuAOX1a*	TRIUR3_10307
AetAOX1d*	AetAOX1d*	F775_18387
AetAOX1d-like*	AetAOX1d-like*	F775_43125
AetAOX1e*	AetAOX1e*	F775_11948
AetAOX1a*	AetAOX1a*	F775_17784
**Non-Expressed**		
**Promoter**	**Classification**	**Ensembl Plants/IWGSC Fragment Location**
N/A	*ne*.*TaAOX1d-2BL*.*1*	RC.TGACv1_scaffold_129474_2BL:235,137–236,895
N/A	*ne*.*TaAOX1d-2BL*.*2*	RC.TGACv1_scaffold_129474_2BL:226867–227725
N/A	*ne*.*TaAOX1d-2DL*	RC.TGACv1_scaffold_160654_2DL:15,057–16,369
N/A	*ne*.*TaAOX•-2AL*	RC.TGACv1_scaffold_093545_2AL:13252–14775
N/A	*Ta*.*Fragment-7BL*	TGACv1_scaffold_576971_7BL:58792–58921
N/A	Tu.Fragment*	C163670370 1–226
N/A	*ne*.AesAOX1d*	RC.TGAC_WGS_speltoides_v1_contig_403763
N/A	*ne*.AesAOX•*	RC.TGAC_WGS_speltoides_v1_contig_239141
N/A	*ne*.AesAOX•*	TGAC_WGS_speltoides_v1_contig_195745
N/A	Fragment*	TGAC_WGS_speltoides_v1_contig_1601667
N/A	Fragment*	TGAC_WGS_speltoides_v1_contig_1653744
N/A	Fragment*	RC.TGAC_WGS_speltoides_v1_contig_2863348
N/A	Aet.Fragment*	RC.C137891329 48–258
N/A	Aet.Fragment*	RC.scaffold67708 39107–39328
N/A	Aet.Fragment*	RC.scaffold94414 27790–27998

RC prefix designates sequences that were reverse-complemented in order to achieve *AOX* sequence identity.

*Indicates diploid promoters or protein isoforms.

**Table 2:** Classification of wheat AOX proteins using Arabidopsis AOX1a as reference.

Protein Name	Type 1 or Type 2 Residues					Type 1 (a-c/e) or Type 2(d) Residues					
	**112**	**124**	**229**	**233**	**241**	**167**	**175**	**178**	**180**	**181**	**295**
TaAOX1a-2AL.sv1											
TaAOX1a-2AL.sv2											
TaAOX1a-2BL											
TaAOX1a-2DL.sv1											
TaAOX1a-2DL.sv2											
TaAOX1a-like-2DL											
regTaAOX-4BL.sv1											
regTaAOX-4BL.sv2											
regTaAOX-4BL.sv3											
regTaAOX-4BL.sv4											
put.regTaAOX-3B											
put.regTaAOX-6BL											
TaAOX1c-6AL											
TaAOX1c-6BL.sv1											
TaAOX1c-6BL.sv2											
TaAOX1c-6BL.sv3											
TaAOX1c-6DL											
regTaAOX-3B											
put.TaAOX1e-3DS											
TaAOX1d-2AL.2.sv1											
TaAOX1d-2AL.2.sv2											
TaAOX1d-2AL.1											
TaAOX1d-2DL											
put.TaAOX1d-like-4AS											
TuAOX1a*											
TuAOX1c*											
TuAOX1d.1*											
TuAOX1d.2*											
AetAOX1a*											
AetAOX1e*											
AetAOX1d*											
AetAOX1d-like*											
ne.TaAOX1d-2BL.1											
ne.TaAOX1d-2BL.2											
ne.TaAOX1d-2DL											
ne.AesAOX1d*											

Residues used are from Costa et al. 2014. Blue indicates presence of Type 1 residues. Red indicates a Type 2 residue. Green indicates residues for monocot Type 1(d). Yellow indicates Type 1(a-c/e). Purple represents amino acid residues that did not match either classification. Black represents residues that were absent.

*Denotes diploid wheat AOX proteins.

Consequently, 12 high-confidence *AOX* genes and four low-confidence genes were found in hexaploid wheat (Table 1 and S4 Fig). Four *AOX* genes were found in each of the two A and D subgenome diploid ancestors, *T*. *urartu* and *A*. *tauschii*. Four non-expressed hexaploid *AOX* genes and three non-expressed *A*. *speltoides* genes were also found. For all the genomes, gene fragments were found and these have been documented (Table 1). The genomes of the diploids are still being resolved and thus it is possible that with further work, some of these fragments discovered would be shown to be part of complete gene sequences. The current study focused on elucidating the information from both high- and low-confidence genes with the caveat that future work could resolve the low-confidence data provided. No analysis was done on the non-expressed genes beyond the phylogeny and protein classification as we have no experimental support for final transcript or translation features. These non-expressed sequences may be transcribed as given, or may undergo intronizations to give sequences which fall into the “-like” or regulatory categories. The artificial non-expressed protein sequences used in the phylogeny give an indication of evolutionary relationships and may indicate function if the protein form is maintained as we assume. However, it is possible that once transcribed and translated, putative intronizations may change the final structure. This simulated use of the non-expressed sequences was only possible for AOX1d as the “ne” nucleotide and protein sequences showed a strong alignment to the full coding regions and protein sequences of high-confidence *TaAOX1d* genes (S5 and S6 Figs). The assumption made was further validated by the observation that these AOX1d “ne” proteins grouped in the AOX1d clade in the phylogeny, an analysis also supported by all the required residues to meet this subclassification (Figs 1 and S1 and Table 2;) [16]. The other non-expressed genes in the *AOX1a*, *AOX1c* or *AOX1e* clades are multiexonic and therefore it was impossible to artificially determine intron exon boundaries and extrapolate the putative protein sequences.

With the exception of a few amino acid substitutions and an insertion, the proteins which were used in the initial search Waox1a (BAB88645.1) and Waox1c (BAB88646.1) were found to align most closely to TaAOX1a-2AL.sv1 and TaAOX1c-6AL respectively (S7 Fig). The substitutions may be due to a varietal difference since the Waox1a and Waox1c proteins were obtained from the wheat variety Mironovskaya 808 [36], whereas sequences from Chinese Spring have been used in this study. It must also be noted that the chromosomal locations noted by the previous researchers of the coding sequences *Waox1a* (AB078882.1) and *Waox1c* (AB078883.1) match that of TaAOX1a-2AL.sv1 and TaAOX1c-6AL respectively [36].

**Fig 3:**
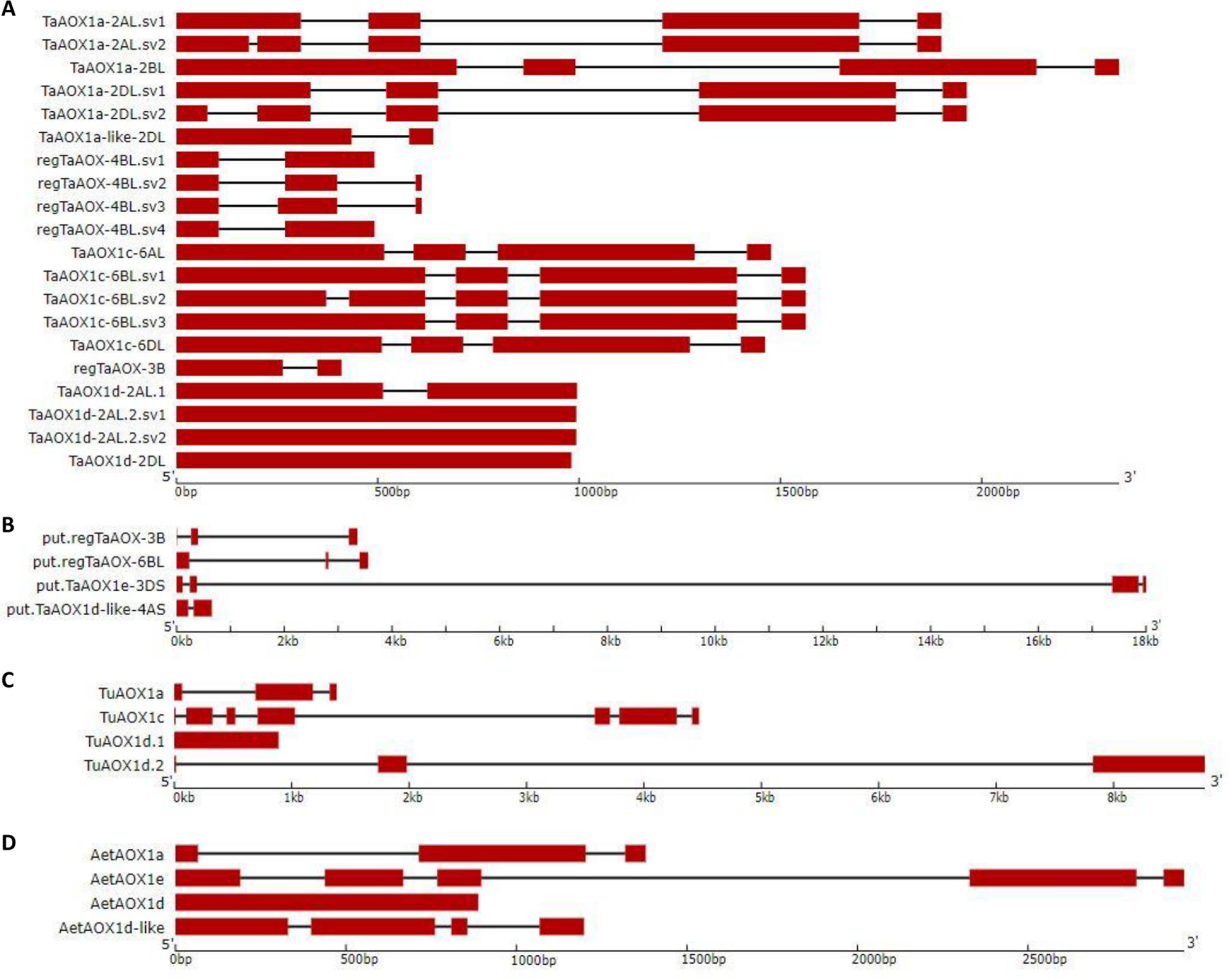
*AOX* gene structures of hexaploid and diploid wheat. Exons are depicted in red, with introns being represented by black lines for (A) high-confidence *T*. *aestivum AOX* gene family, (B) low-confidence *T*. *aestivum AOX* gene family (C) *T*. *urartu AOX* gene family, and (D) *A*. *tauschii AOX* gene family.

Hexaploid *AOX* transcripts ranged in size from 1180 bp to 3274 bp with coding regions between 249 bp and 1374 bp (Table 3 and S4 Fig). The notable exception was *put*.*TaAOX1e-3DS* where intron 2 was almost 17000 bp. There were splice variants in some *AOX* genes resulting from 5’ and 3’ alternative splice sites as well as intronizations (Table 4). Within the hexaploid coding regions, it was observed that the *AOX1a* clade generally had the longest introns while the *AOX1d* clade genes were intronless or single-intron (Fig 3). Given the few occurrences genes in the *AOX1e* clade, it was to conclude any gene structure patterns. The transcripts and coding sequences of the diploid *AOX* genes spanned 615 bp to 1305 bp (Table 3). There were one to six exons in the transcripts giving one to five exons within the start and stop codons (Fig 3 and Table 3). There was at least one gene from each of the diploid *AOX1d* clade which was also monoexonic within the coding regions, mirroring what was observed in the hexaploids (Fig 3 and Table 3). In contrast to the multi-exonic nature of most *AOX* genes described in the literature from a variety of species [27, 78, 79], it seems that hexaploid wheat shows clade-dependent differences in gene structures (Fig 3 and Table 3). In yeast, moss, *A*. *thaliana*, mice, rice and switchgrass it has been suggested that genes with fewer introns are rapidly activated or are highly responsive to environmental changes or stress [78, 80–82]. The simplicity of the gene structure denoted by the presence of few or no introns leads to faster processivity of the pre-mRNA, which in turn leads to faster accumulation of the protein. In contrast, other researchers have found that highly expressed genes had more complex gene organization as indicated by more and longer introns in both *A*. *thaliana* and rice [83, 84]. The expression strategies described above are both possible in the wheat *AOX* genes given the variation in gene structures between the clades and may be suggestive of a mosaic pattern of expression.

**Table 3:** Features of *AOX* genes in hexaploid and diploid wheat.

	Gene Name	Length of Gene (bp)	Transcript Length (bp)	Coding Sequence (bp)	# of Exons	# of Introns
**Hexaploid**	*TaAOX1a-2AL*.*sv1*	2369	1456	987	4	3
	*TaAOX1a-2AL*.*sv2*	2369	1432	963	5	4
	*TaAOX1a-2BL*	6169	2102	1374	4	3
	*TaAOX1a-2DL*.*sv1*	2419	1467	1011	4	3
	*TaAOX1a-2DL*.*sv2*	2419	1341	885	5	4
	*TaAOX1a-like-2DL*	1515	1372	495	2	1
	*regTaAOX-4BL*.*sv1*	4284	1571	327	5	4
	*regTaAOX-4BL*.*sv2*	4284	3158	249	4	3
	*regTaAOX-4BL*.*sv3*	4284	3176	267	4	3
	*regTaAOX-4BL*.*sv4*	4284	3274	327	4	3
	*put*.*regTaAOX-3B*	3652	585	291	3	2
	*put*.*regTaAOX-6BL*	3833	718	441	3	2
	*TaAOX1c-6AL*	2019	1736	1194	4	3
	*TaAOX1c-6BL*.*sv1*	2207	1940	1296	4	3
	*TaAOX1c-6BL*.*sv2*	2207	1806	1239	6	5
	*TaAOX1c-6BL*.*sv3*	2207	1863	1296	5	4
	*TaAOX1c-6DL*	2075	1801	1188	4	3
	*regTaAOX-3B*	1456	1370	324	2	1
	*put*.*TaAOX1e-3DS*	18352	1142	789	4	3
	*TaAOX1d-2AL*.*1*	1290	1180	885	2	1
	*TaAOX1d-2AL*.*2*.*sv1*	1423	1423	993	1	0
	*TaAOX1d-2AL*.*2*.*sv2*	2395	1269	993	2	1
	*TaAOX1d-2DL*	1405	1405	981	1	0
	*put*.*TaAOX1d-like-4AS*	1065	963	552	2	1
**A Genome**	TuAOX1a*	1383	615	615	3	2
	TuAOX1c*	4469	1305	1305	7	6
	*TuAOX1d*.1*	888	888	888	1	0
	*TuAOX1d*.2*	8777	1212	1212	3	2
**D Genome**	AetAOX1a*	1379	615	615	3	2
	AetAOX1e*	2958	1098	1098	5	4
	AetAOX1d*	888	888	888	1	0
	AetAOX1d-like*	1198	870	870	4	3

*Denotes diploid wheat AOX genes.

**Table 4:** Splice variants of hexaploid wheat *AOX* genes.

Gene Name	Status	Points of Difference
*TaAOX1a-2AL*.*sv1*	Wildtype	Intron retention: Portion of exon 1 in wildtype is intron 1 in variant.
*TaAOX1a-2AL*.*sv2*	Variant	
*TaAOX1a-2BL*	N/A	N/A
*TaAOX1a-2DL*.*sv1*	Wildtype	Intron retention: Portion of exon 1 in wildtype is intron 1 in variant.
*TaAOX1a-2DL*.*sv2*	Variant	
*TaAOX1a-like-2DL*	N/A	N/A
*regTaAOX-4BL*.*sv1*	Wildtype	Intron retention: Portion of exon 2 in wildtype is part of intron 2 in variant. Portion of exon 4 in variant is intron 4 in wildtype.
*regTaAOX-4BL*.*sv2*	Variant	
*regTaAOX-4BL*.*sv3*	Variant	Intron retention: Portion of exon 2 in wildtype is a part of intron 2 in variant. Portion of exon 2 in variant is part of intron 1 in wildtype. Portion of exon 4 in variant is intron 4 in wildtype.
*regTaAOX-4BL*.*sv4*	Variant	Intron retention: Portion of exon 4 in variant is intron 4 in wildtype.
*put*.*regTaAOX-3B*	N/A	N/A
*put*.*regTaAOX-6BL*	N/A	N/A
*TaAOX1c-6AL*	N/A	N/A
*TaAOX1c-6BL*.*sv1*	Wildtype	Intron retention: Portion of exon 1 in wildtype is part of intron 1 in variant. Portion of exon 4 in wildtype is part of intron 5 in variant.
*TaAOX1c-6BL*.*sv2*	Variant	
*TaAOX1c-6BL*.*sv3*	Variant	Intron retention: Portion of exon 4 in wildtype is intron 4 in variant.
*TaAOX1c-6DL*	N/A	N/A
*regTaAOX-3B*	N/A	N/A
*put*.*TaAOX1e-3DS*	N/A	N/A
*TaAOX1d-2AL*.*1*	N/A	N/A
*TaAOX1d-2AL*.*2*.*sv1*	Wildtype	Intron retention: Portion of exon 1 in wildtype is part of intron 1 in variant. Alternative 3'UTR site: Portion of exon2 in variant is downstream of the gene sequence of the wildtype.
*TaAOX1d-2AL*.*2*.*sv2*	Variant	Alternative 5'UTR site: part of the wildtype's 5'UTR is upstream of the gene sequence of the variant
*TaAOX1d-2DL*	N/A	N/A
*put*.*TaAOX1d-like-4AS*	N/A	N/A

**Fig 4:**
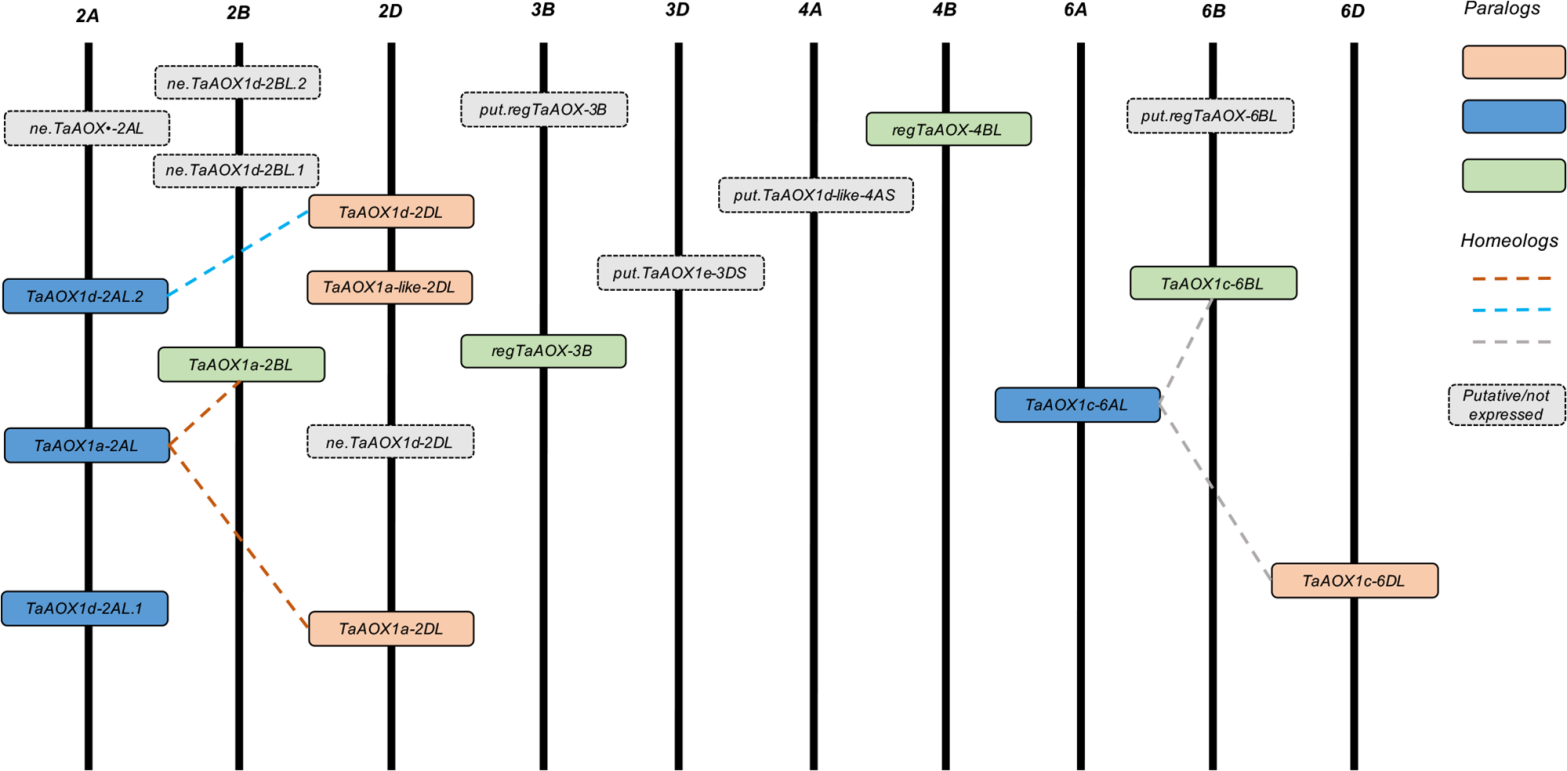
Distribution of *AOX* homeologs and paralogs on hexaploid wheat chromosomes. Boxes of the same color indicate paralogs, while lines of the same color indicate homeologous groups. Grey boxes with a dashed outline indicate putative or non-expressed genes.

The paralogs and homeologs for the hexaploid *AOXs* were also identified by Ensembl Plants. There were paralogs in all the high-confidence hexaploid genes all located on long chromosomal arms. With the exception of *TaAOX1a-like-2DL*, *regTaAOX-4BL*, *regTaAOX-3B* and *TaAOX1d-2AL*.*1* homeologs were found (Fig 4). The gene sequences were aligned with the draft physical genome sequences downloaded from Ensembl Plants (ftp://ftp.ensemblgenomes.org/pub/release-39/plants/fasta/triticum_aestivum/dna/). The higher resolution of the latest version of the genome facilitated the placement of the hexaploid genes on the chromosomal arms. It was clear that most of the *AOX* genes were on the long chromosomal arms with about half the total the long arms of the subgenomes of chromosome 2 (Fig 4). The two hexaploid *AOX* genes with the shortest distance on the same chromosome were *ne*.*TaAOX1d-2BL*.*1* and *ne*.*TaAOX1d-2BL*.2 which were 8.6 kbp apart. *TaAOX1a-2BL* and *ne*.*TaAOX1d-2BL*.*1* were the farthest apart with 301 Mbp between the two genes (S8 Fig). It is possible that with future work especially on the non-expressed and putative gene copies, the relationships between these genes and the high-confidence *AOX* gene copies will be better established. This will allow for a better study of the functionality of these paralogs within the hexaploid wheat genome.

Epigenetic gene regulation via CpG islands can lead to diversity and specificity in gene expression [85, 86]. The gene sequences discovered were input into the CpgPlot program in order to find putative CpG islands. The greatest CpG distribution was in the *AOX1a* clade followed by that of the *AOX1c* clade (Table 5). While no clear relationship could be established between these GC-rich regions and levels of transcription, it must be noted that these variations could become more relevant under other experimental conditions which were unexplored in the RNA-seq dataset used in the present study (exposure to phytohormones, salt stress, long-term heat and drought stress). CpG islands were not examined in the putative hexaploid genes and the diploids as significant gene regions were unresolved in the database.

**Table 5:** CpG islands in the promoters and gene bodies of the high-confidence *TaAOX* gene family.

Gene Name	Promoter			Gene Body		
	# of Islands	Regions	Length	# of Islands	Regions	Length
*TaAOX1a-2AL*.*sv1*	1	(1040..1444)	405	2	(47..845), (1192..1730)	799, 539
*TaAOX1a-2AL*.*sv2*						
*TaAOX1a-2BL*	1	(51..289)	239	2	(51..1249), (1622..2167)	1199, 546
*TaAOX1a-2DL*.*sv1*	1	(1207..1444)	238	2	(47..910), (1273..1819)	864, 547
*TaAOX1a-2DL*.*sv2*						
*TaAOX1a-like-2DL*	3	(139..355), (554..1045), (1064..1444)	217, 492, 381	1	(47..464)	418
*regTaAOX-4BL*.*sv1*	3	(427..690), (860..1203), (1208..1444)	264, 344, 237	1	(49..435)	387
*regTaAOX-4BL*.*sv2*				1	(49..511)	463	
*regTaAOX-4BL*.*sv3*				1	(49..511)	463	
*regTaAOX-4BL*.*sv4*				1	(49..435)	387	
*TaAOX1c-6AL*	1	(494..720)	227	1	(48..1356)	1309
*TaAOX1c-6BL*.*sv1*	0			2	(49..522), (586..1431)	474, 846
*TaAOX1c-6BL*.*sv2*						
*TaAOX1c-6BL*.*sv3*						
*TaAOX1c-6DL*	0			1	(48..1327)	1280
*regTaAOX-3B*	0			1	(48..317)	270
*TaAOX1d-2AL*.*1*	0			1	(48..938)	891
*TaAOX1d-2AL*.*2*.*sv1*	2	(49..380), (768..1408)	332, 641	1	(47..937)	891
*TaAOX1d-2AL*.*2*.*sv2*						
*TaAOX1d-2DL*	2	(702..1064), (1079..1402)	363, 324	1	(47..925)	879

### AOX protein phylogeny reveals four lineages of wheat *AOX* genes, each with multiple copies

In order to hypothesize the major lineages of wheat *AOX* gene copies and infer the number and timing of duplication events, amino acid sequences of the diploid and hexaploid wheat AOX proteins in combination with other monocot sequences were used to generate a phylogenetic hypothesis of gene family evolution (Figs 1 and S1). All putative AOX copies in wheat and as many putative AOX1 paralogs as possible from other species were included in order to better assess the timing of duplication events. Four lineages of wheat *AOX* genes were found, corresponding to the *AOX1a*, *AOX1c*, *AOX1e* and *AOX1d* genes previously found [16, 17]. Wheat gene copies within each lineage are unevenly distributed: the *AOX1a* lineage includes seven *T*. *aestivum* copies (including regulatory, putative regulatory, and -like copies), but only one each from *T*. *urartu* and *A*. *tauschii*; the *AOX1c* lineage includes three *T*. *aestivum* copies and one from *T*. *urartu*; the *AOX1d* lineage includes seven *T*. *aestivum* copies, two *T*. *urartu* and three *A*. *tauschii* copies; and the *AOX1e* lineage includes two *T*. *aestivum* and one *A*. *tauschii* copies. Given the incomplete and only partially annotated nature of the *T*. *urartu* (A-subgenome) and *A*. *tauschii* (D-subgenome) genomes and the lack of availability of the *A*. *speltoides* (B-subgenome) genome, it is unclear how to interpret the lack of copies or variable number of copies of some of the duplication types. Some gene types (e.g., *AOX1c*) fit what we would expect for a hexaploid (three copies), while other types have more variable copy number in wheat (*AOX1a*, seven copies). *AOX1d* appears to be a triticoid-specific duplication with what appears to be two clades of *AOX1d* genes in wheat and relatives, with the duplication after the divergence of triticoid grasses from *Brachypodium* (Figs 1 and S1). It is important to note that while *Brachypodium* also has two *AOX1d* gene copies, those are from a different duplication event than the triticoid duplication.

In addition to the variation in copy number among *AOX1* clades, the variation in the number of splice variants is unevenly distributed. As with the number of gene copies, the *AOX1a* clade also has the most splice variants with eight splice variants found from three genomic copies, whereas only three splice variants from one gene copy are found in the *AOX1c* lineage and two splice variants from one gene copy in the *AOX1d* lineage (Fig 3 and Table 1). No splice variants were found for the *AOX1e* genes. The pattern of duplication and divergence in the *AOX1a* gene lineage needs further study, but suggests that these genes are undergoing rapid duplication and divergence in sequence characteristics and, presumably, function. Whether the co-occurrence of multiple splice variants and duplication of genomic copies are connected will require further evaluation, but it is plausible that splice variant reintroduction to the genome as a duplication mechanism could drive the gene proliferation that we document here [87, 88]

### Promoter analyses reveal regulatory motifs in wheat *AOX* gene family

**Fig 5:**
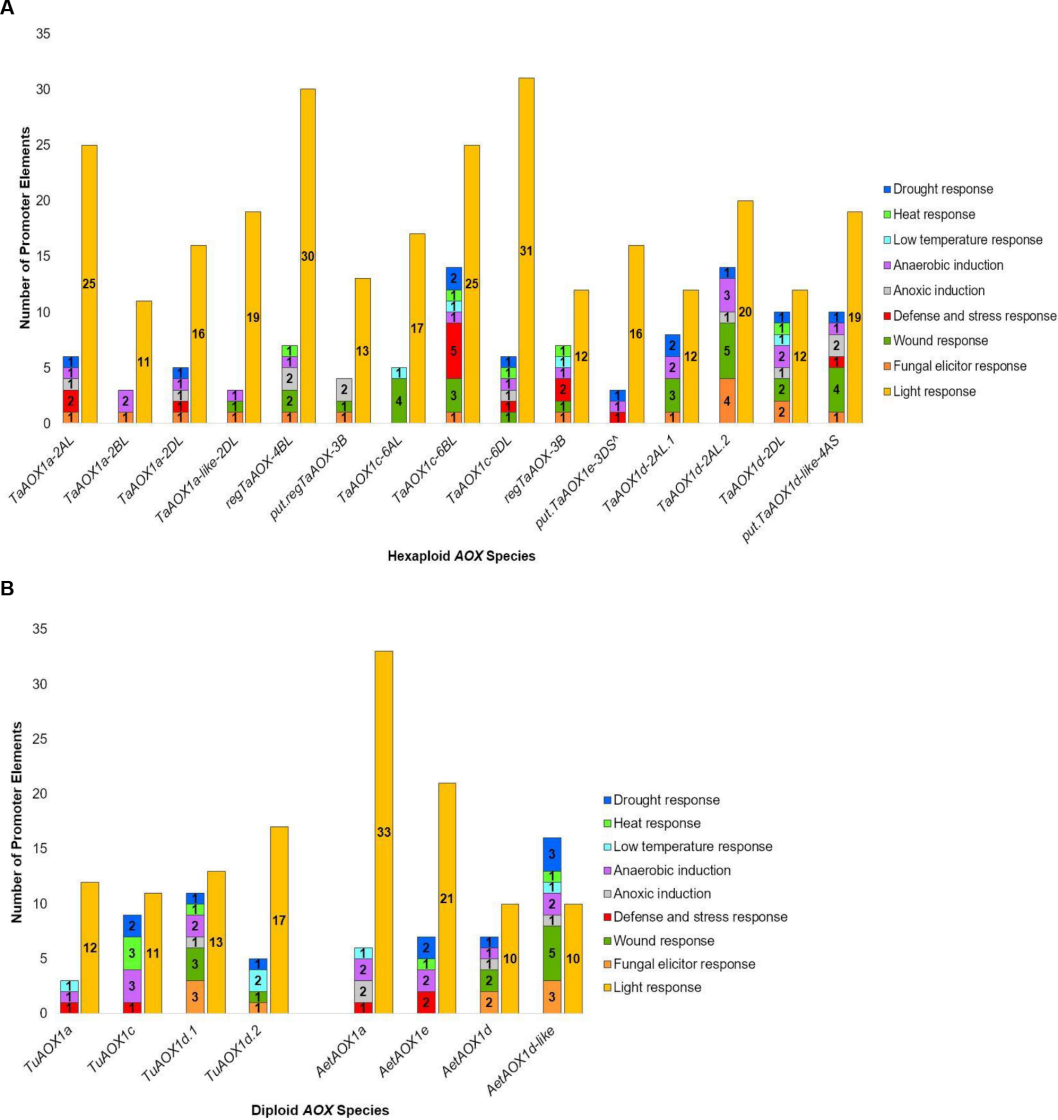
Putative cis-elements for abiotic response in promoter regions. (A) *TaAOX* gene family and (B) diploid *AOX* gene families. The promoter for *put*.*regTaAOX-6BL* was not analyzed due to the majority of the promoter sequence being unresolved.

**Fig 6:**
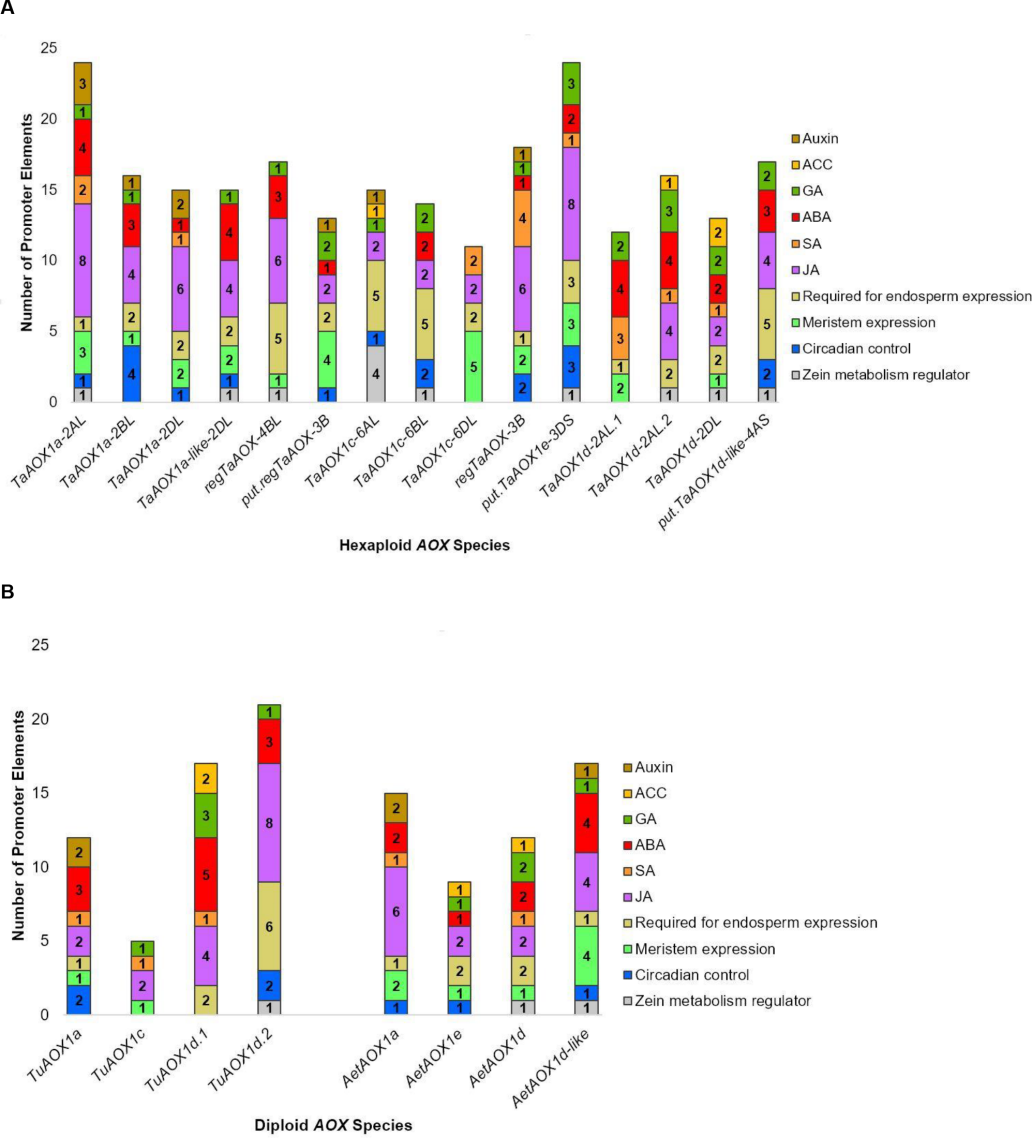
Putative cis-elements for hormonal and developmental responses in promoter regions. (A) *TaAOX* gene family and (B) diploid *AOX* gene families. The promoter for *put*.*regTaAOX-6BL* was not analyzed due to the majority of the promoter sequence being unresolved.

To identify putative regulatory elements of the wheat *AOX* genes, promoter elements were identified in the 1500 bp sequence upstream of the translation start site (S4 Fig). In the hexaploid and diploid species, the greatest proportion of elements was for light response (Fig 5). It was also apparent that there was diversity between family members with regards to elements involved in hormonal, developmental, biotic and abiotic environmental responses (Figs 5 and 6). Cumulatively, the greatest numbers of these hexaploid response elements were found in *TaAOX1a-2AL*, *regTaAOX-4BL* and *TaAOX1c-6BL*. The smallest numbers were observed in *TaAOX1a-2BL*, *put*.*regTaAOX3B*, *TaAOX1d-2AL*.*1* and *TaAOX1d-2DL* (Figs 5 and 6). Out of these the highest numbers of environmental response elements were found in *regTaAOX-4BL*, *TaAOX1c-6BL*, *TaAOX1c-6DL*, and *TaAOX1d-2AL*.*2* (Fig 5A). The highest numbers of hexaploid hormonal and developmental response elements were found in *TaAOX1a-2AL*, *regTaAOX-3B* and *put*.*TaAOX1e-3DS* (Fig 6A). The jasmonic acid (JA) and abscisic acid (ABA) response elements were also common across many family members in the hexaploid and diploid species (Fig 6). There was a large proportion of specific response elements in some promoters, e.g. *TaAOX1a-2AL* (JA), *TaAOX1a-2BL* (circadian control) and *TaAOX1d-2AL*.*2* (fungal elicitor response) (Figs 5A and 6A). While the low temperature response elements were absent in the hexaploid *AOX1a* promoters, they were present in the diploid *AOX1a* promoters and may indicate levels of control peculiar to the diploid species (Fig 5). Many of these factors such as light, heat, drought, ABA, and SA have already been shown to cause induction of *AOX* in wheat and other plants [10, 37–39, 42]. However, the presence of motifs for gibberellic acid (GA), jasmonic acid (JA), ethylene (ACC) and others (Fig 6) suggest that there is still a lot of work to be done in terms of how these phytohormones and developmental factors are integrated into the framework of *AOX* expression and regulation. The commonality of certain elements across many family members could also give ways of inducing the expression of some or all of the low-confidence and non-expressed *AOX* genes that have been reported in the current study. It must be noted that some regions of the promoters were unresolved and therefore additional motifs may be found that could be specific to the diploids or show similar elements as in the hexaploid counterparts.

Previous research has revealed a number of positive and negative regulators of *AOX* (S3 Table). Generally, regulator motifs were common in the *AOX1a* and *AOX1d* clades and absent in the *AOX1c* and *AOX1e* clades. Motifs were found for known positive regulators of *AOX* (Tables 6, 7, S3 and S4) in all the promoters except those of *put*.*regTaAOX-3B*, *put*.*regTaAOX-6BL*, *TaAOX1c-6AL*, *TaAOX1c-6BL*, *TaAOX1c-6DL*, *regTaAOX-3B*, *put*.*TaAOX1e-3DS*, *put*.*TaAOX1d-like-4AS*, *TuAOX1d*.*2*, *TuAOX1c* and *AetAOX1e*. The NAC Domain Containing Protein 17 (ANAC017) (At1g34190) is considered to be a critical positive regulator of *AOX* [89, 90] and motifs for this protein were well-represented in the *AOX1a* and *AOX1d* clades (*TaAOX1a-2AL*, *TaAOX1a-2BL*, *TaAOX1a-2DL*, *TaAOX1a-like-2DL*, *TaAOX1d-2AL*.*2*, *TaAOX1d-2DL*, *TuAOX1d*.*1*, *AetAOX1a*, *AetAOX1d*, and *AetAOX1d-like*) (Tables 6 and 7). Another positive regulator WRKY DNA-Binding Protein 63 (AtWRKY63, At1g66600) [91] was more common in the *AOX1d* clade (*TaAOX1a-like-2DL*, *TaAOX1d-2AL*.*2*, *TaAOX1d-2DL*, *TuAOX1d*.*1* and *AetAOX1d*) (Tables 6 and 7). Motifs for the negative regulator ABA Insensitive 4 (ABI4, At2g40220) [92] were found in all the promoters except *TaAOX1c-6AL*, *TaAOX1c-6BL* and *AetAOX1e* (Tables 6, 7, S3 and S4). The binding sites for another negative regulator WRKY DNA-Binding Protein 40 (ATWRKY40, At1g80840) [90] existed only in the *TaAOX1c-6BL* promoter (Tables 6, 7, S3 and S4).

**Table 6:** Occurrence of conserved motifs for known positive and negative AOX regulators in the promoters of the *TaAOX* gene family.

Locus	*TaAOX1a-2AL*	*TaAOX1a-2BL*	*TaAOX1a-2DL*	*TaAOX1a-like-2DL*	*regTaAOX-4BL*	*put*.*regTaAOX-3B*	*TaAOX1c-6AL*	*TaAOX1c-6BL*	*TaAOX1c-6DL*	*regTaAOX-3B*	*put*.*TaAOX1e-3DS*	*TaAOX1d-2AL*.*1*	*TaAOX1d-2AL*.*2*	*TaAOX1d-2DL*	*put*.*TaAOX1d-like-4AS*
At1g32870	3	2	3	1									1	1	
At1g34190	2	2	2	1									1	1	
At1g66600				1									3	1	
At3g10500	2	2	2	1									1	1	
At5g04410	1			2	1							1	1		
At1g80840*								1							
At2g40220*	4	2	4	6	8	10			1	1	6	2	2	4	2
CTTGNNNNNCAMG	2	2	2	2									2	2	
YTTGNNNNNVAMV	8	5	6	4	2	3	4	1	1			1	4	3	

The promoter for *put*.*regTaAOX-6BL* was not analyzed due to the majority of the promoter sequence being unresolved.

*Denotes negative regulators.

**Table 7:** Occurrence of conserved motifs for known positive and negative AOX regulators in the promoters of the diploid *AOX* gene families.

Locus	*TuAOX1a*	*TuAOX1c*	*TuAOX1d*.*1*	*TuAOX1d*.*2*	*AetAOX1a*	*AetAOX1e*	*AetAOX1d*	*AetAOX1d-like*
At1g32870	1		1		3		1	2
At1g34190			1		2		1	2
At1g66600			2				1	
At3g10500			1		2		1	1
At5g04410								1
At2g40220*	2	1	2	1	5		3	4
CTTGNNNNNCAMG			2		2			2
YTTGNNNNNVAMV	2	3	3	4	3	2	3	6

*Denotes negative regulators.

Previous researchers analyzed *A*. *thaliana* transcriptome data obtained via induction with mitochondrial regulation perturbation reagents. Further analysis on the promoters of the highly responsive and upregulated genes showed the presence of the cis-regulatory element CTTGNNNNNCAMG [93] labeled the mitochondrial dysfunction motif (MDM). All the genes with the MDM motif which were upregulated during disruption of mitochondrial retrograde regulation were thereafter referred to as the *MITOCHONDRIAL DYSFUNCTION STIMULON* (*MDS*) genes which include *AOX*. The protein ANAC013 (At1g32870) controls the *MDS* genes by direct interaction with the MDM motif CTTGNNNNNCAMG or the alternative YTTGNNNNNVAMV (sequence variation in orthologs) (Tables 6, 7, S3 and S4). [93]. In the current study, a search was conducted for the MDM motif in the wheat *AOX* gene family promoter regions. The stringent motif, CTTGNNNNNCAMG, was found in all promoters except *regTaAOX-4BL*, *put*.*regTaAOX-3B*, *put*.*regTaAOX-6BL*, *TaAOX1c-6AL*, *TaAOX1c-6BL*, *regTaAOX-3B*, *put*.*TaAOX1e-3DS*, *TaAOX1d-2AL*.*1*, *put*.*TaAOX1d-like-4AS*, *TuAOX1a*, *TuAOX1c*, *TuAOX1d*.*2*, *AetAOX1d* and *AetAOX1e*. The alternative MDM motif (YTTGNNNNNVAMV) was found in all promoters except *regTaAOX-3B* and *put*.*regTaAOX-6BL* (Tables 6, 7, S3 and S4). Furthermore, the promoters for *TaAOX1a-2AL*, *TaAOX1a-2DL*, *TaAOX1a-like-2DL*, *TaAOX1c-6AL*, *TaAOX1d-2AL*.*2*, *TuAOX1a*, *TuAOX1d*.*2*, and *AetAOX1e* contained the YTTGNNNNNVAMV motif but with only one nucleotide deviation from the stringent motif (S4 Table). This single nucleotide deviation was also found amongst some of the 24 MDM motif genes identified in a previous study [93]. This could indicate that for *TaAOX1c-6AL* and *TuAOX1a*, which lacked the stringent motif, they could still be controlled by ANAC013. The results found using the YTTGNNNNNVAMV motif could therefore be false positives as in *A*. *thaliana* there are other genes containing this MDM motif that have regulators other than ANAC013. They could also indicate that although not directly controlled by ANAC013, they may still be involved in the network of mitochondrial retrograde regulation [93]. If valid, this motif distribution allows for greater levels of control in how this gene family is expressed and may provide clues as to how to induce the expression of some or all of the non-expressed wheat *AOX* genes.

It must also be noted that the pattern or mode of expression could differ between the hexaploids and the diploids. Given that the promoter regions of the diploid species are still being sequenced, it is entirely possible that additional motifs of other aforementioned positive regulators could be discovered. This would further our knowledge of the regulation of *AOX* in the wild ancestors of bread wheat. Motifs for the hypoxia responsive promoter element [94] as well as known positive and negative regulators of *A*. *thaliana* AOX, At5g13610, At5g63610, At5g12290, At5g07690 and At1g32230 [95–99] were missing from both the hexaploid and diploid promoters (Tables 6, 7, S3 and S4). It is possible that these factors are restricted to dicots and have evolved a different form of control or have significantly diverged in grasses or monocots.

We also examined the CpG islands in the *AOX* promoters (S4 Fig). In the promoter regions, the largest distribution of CpG islands was in the genes of the *AOX1a* clade followed by the *AOX1d* clade. The lowest CpG distribution was in the *AOX1c* and *AOX1e* clades (Table 5). Similar to the *AOX* gene sequences, there was no obvious relationship between the number or length of CpG islands and the regulation of the transcripts from these promoters. Again, it is entirely possible that variations may emerge under new experimental conditions such as longer-term abiotic stresses and exposure to phytohormones.

### *AOX* expression is diverse among all family members over tissue types and developmental stages

**Fig 7:**
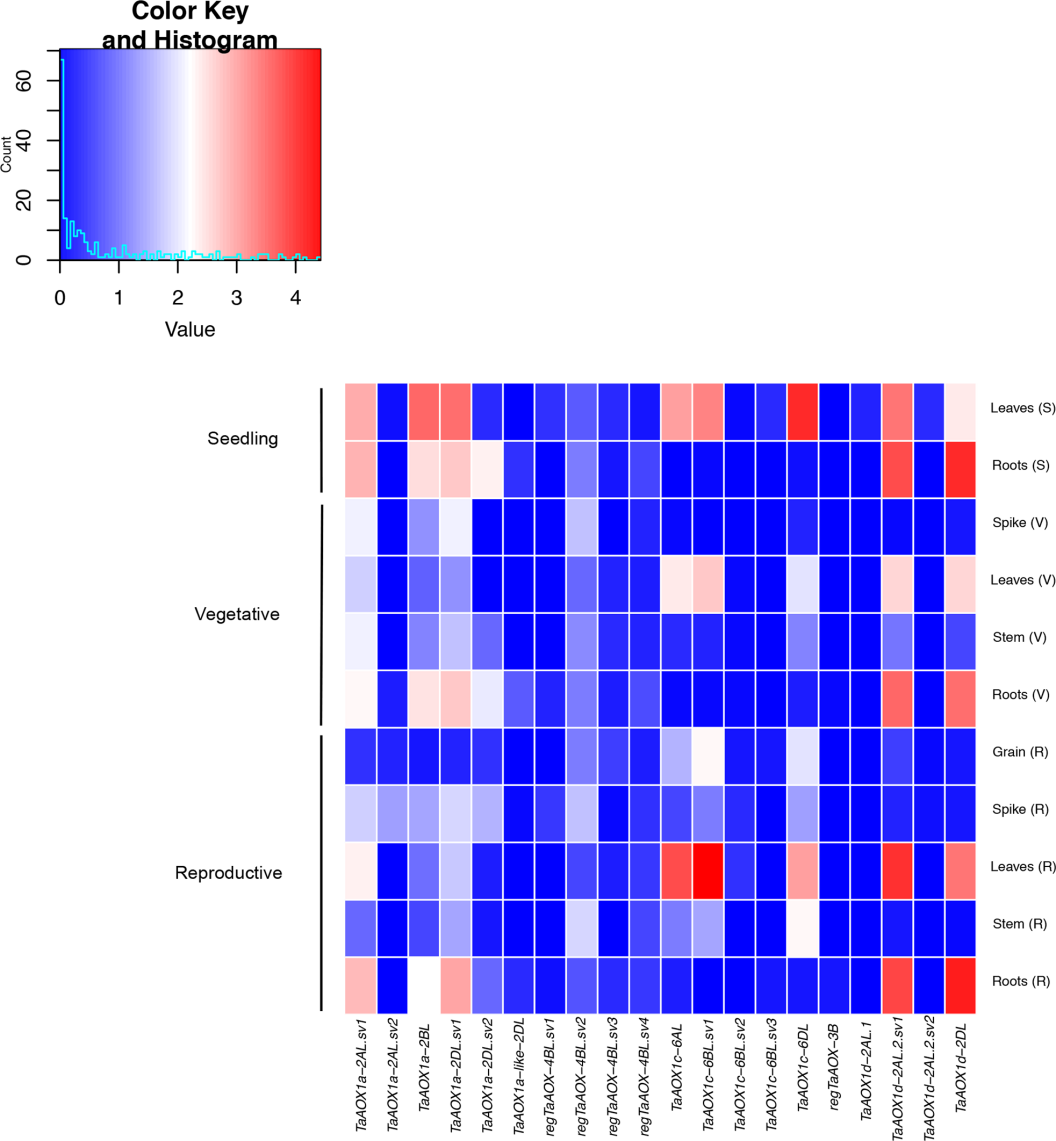
Heat map of expression profiles for high-confidence *TaAOX* genes at different developmental stages.

To provide further insight into wheat *AOX* function, RNA-seq data were obtained from studies deposited into the wheat database expVIP [55]. Data for the high-confidence *AOX* genes was obtained and expression was found in multiple tissues at various developmental stages as well as over various environmental conditions. Ten out of 20 possible transcripts were expressed in all tissues examined at all three developmental stages (seedling, vegetative and reproductive) (Fig 7 and S5 Table). These were, *TaAOX1a-2AL*.*sv1*, *TaAOX1a-2BL*, *TaAOX1a-2DL*.*sv1*, *regTaAOX-4BL*.*sv2*, *regTaAOX-4BL*.*sv4*, *TaAOX1c-6AL*, *TaAOX1c-6BL*.*sv1*, *TaAOX1c-6DL*, *TaAOX1d-2AL*.*2*.*sv1* and *TaAOX1d-2DL*. There was a low-level of expression for *TaAOX1a-2AL*.*sv2*, *TaAOX1a-like-2DL*, *regTaAOX-4BL*.*sv1*, *regTaAOX-4BL*.*sv3*, *regTaAOX-4BL*.*sv4*, *TaAOX1c-6BL*.*sv2*, *TaAOX1c-6BL*.*sv3*, *regTaAOX-3B*, *TaAOX1d-2AL*.*1* and *TaAOX1d-2AL*.*2sv2* in all tissue and developmental stages tested. Of note were *TaAOX1a-2AL*.*sv1*, *TaAOX1a-2BL*, *TaAOX1a-2DL*.*sv1*, *TaAOX1d-2AL*.*2*.*sv1* and *TaAOX1d-2DL* which had higher expression in the root at all three developmental stages (Fig 7 and S5 Table). There were a few transcripts with high root expression in particular stages. *TaAOX1a-2DL*.*sv2* and had higher root expression at the seedling and vegetative stages (Fig 7 and S5 Table).

**Fig 8:**
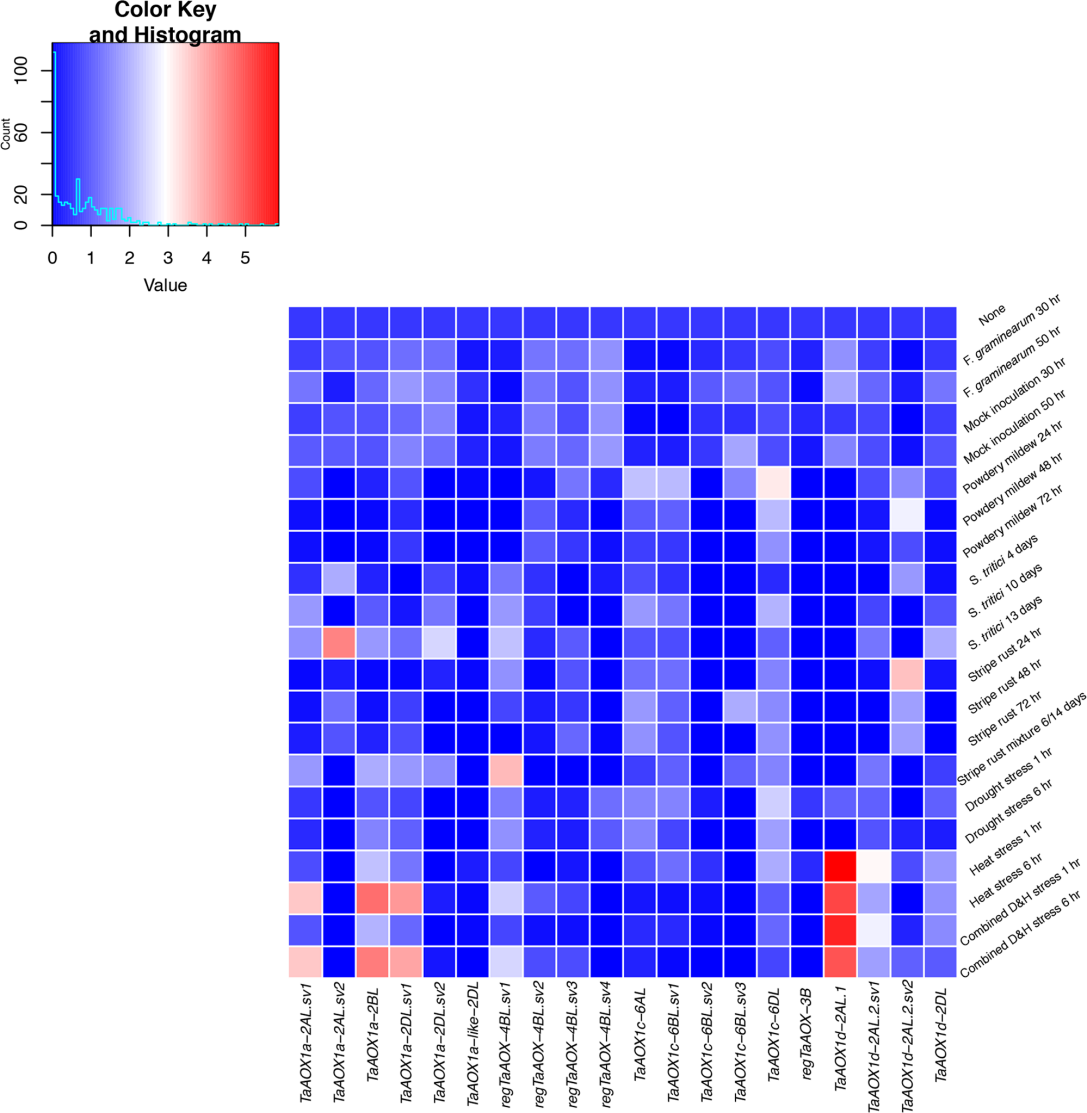
Heat map of expression profiles for high-confidence *TaAOX* genes under biotic and abiotic stresses.

*TaAOX1a-2BL*, *TaAOX1a-2DL*.*sv1*, *regTaAOX-4BL*.*sv1*, *TaAOX1c-6AL*, TaAOX1c*-6BL*.*sv1*, *TaAOX1c-6DL*, *TaAOX1d-2AL*.*1*, *TaAOX1d-2AL*.*2*.*sv1* and *TaAOX1d-2DL* were upregulated under drought stress (Fig 8 and S5 Table). Under heat stress, the highest level of transcript expression was notably *TaAOX1a-2BL*, *TaAOX1a-2DL*.*sv1* and *TaAOX1d-2AL*.*1*. In addition, *TaAOX1a-2AL*.*sv1*, *regTaAOX-4BL*.*sv1*, *regTaAOX-4BL*.*sv2*, *TaAOX1c-6AL*, *TaAOX1c-6BL*.*sv1*, *TaAOX1c-6DL*, *TaAOX1d-2AL*.*2*.*sv1*, *TaAOX1d-2AL*.*2*.*sv2* and *TaAOX1d-2DL* were expressed under heat but to a lesser extent. In contrast, *TaAOX1a-2AL*.*sv2* and *TaAOX1c-6BL*.*sv3* showed no expression under heat or drought stress (Fig 8 and S5 Table). Overall, heat stress had a higher impact on expression levels than drought stress. Under various forms of biotic stress (*Fusarium graminearum*, powdery mildew, stripe rust, *Septoria tritici*), *TaAOX1a-2AL*.*sv2*, *TaAOX1a-2DL*.*sv2*, *regTaAOX-4BL*.*sv1*, *TaAOX1c-6DL* and *TaAOX1d-2AL*.*2*.*sv2* showed high expression levels (Fig 8 and S5 Table). *TaAOX1a-2AL*.*sv1*, *TaAOX1a-2BL*, *TaAOX1a-2DL*.*sv1*, *TaAOX1a-2DL*.*sv2*, *regTaAOX-4BL*.*sv3*, *TaAOX1c-6AL*, *TaAOX1c-6BL*.*sv1*, *TaAOX1c-6BL*.*sv3*, *TaAOX1d-2AL*.*2*.*sv1* and *TaAOX1d-2DL* were also upregulated during biotic stress (Fig 8 and S5 Table). *RegTaAOX-3B* was mostly dormant during biotic stress. The mosaic pattern of results indicates diversity in the level of expression between gene family members and between splice variants of the same gene. This allows for nuance and complexity in function over numerous environmental and physiological conditions. In contrast to previous research, there was no clear relationship between the number of exons and the level of transcript expression [81–84]. It may be that unique physiology, polyploidization and the alternative splicing machinery have given rise to alternate forms of transcriptional regulation in wheat. There was no expression data available for the diploid ancestors. However, polyploidization can lead to neofunctionalization [100, 101] and therefore it is possible that the expression patterns and subsequent protein activities may differ between the hexaploid and diploid wheat species. This highlights the need for expression data in the diploid progenitor species as well.

With regard to the Chinese Spring reference transcriptome, the expVIP database which integrates the most reliable gene models from Ensembl Plants, the most consistent and reliable information that has been supported by expression data from other species [102]. The transcriptome data generated by this database relied on genomic resources such as nullitetrasomic lines as well as the latest RNA-seq software in the analysis [55] to pinpoint the chromosomal localizations and ensure accurate placement on the physical map. In addition, this database contains information that has been shown by other researchers to be validated by alternate sources [77, 103]. A comparison of the transcript sequences shows that there are enough differences for further validation via future qPCR experiments (S6 Table). The current study provides information which can be used in future biological experiments over the same or different conditions used in this study. Different varieties may express these *AOX* genes differently and therefore drawing broad conclusions may not apply to other wheat cultivars or landraces or diploid species. It is critical that elite cultivars being used by researchers be utilized in further studies to further validate the sequences and gene structures shown in the present study. Future experiments may lead to the discovery of new alleles which may show different expression patterns than those revealed in the present study. Subsequent research may also show the occurrence of gene copy number variations, a phenomenon known to occur in the polyploid wheat and which has been shown to cause a spectrum of phenotypic differences that may depend on the geographic region of the cultivars in question [104, 105]. Researchers may therefore find that their respective cultivars have more *AOX* copies which may provide adaptation advantages nonexistent the Chinese Spring cultivar. The possibilities described may require alternate ways of triggering the induction of the newly discovered gene copies as well as the low-confidence and the non-expressed *AOX* genes in order to validate the transcripts and ensure that qPCR primers designed would be gene-specific. For example, hormone elicitors such as jasmonic acid (JA) could trigger expression as JA-responsive elements are found in some wheat *AOX* promoters (Fig 6). Alternatively, reagents that disrupt mitochondrial regulation could be used as in previous studies [93]. Regardless, these data lay the groundwork and generates new hypotheses concerning *AOX* gene expression and function given the isoforms discovered.

Determining expression profiles of *AOX* genes in the diploid ancestors could lead to a better understanding of how these genes evolved in the polyploid species as has been shown in wild relatives of rice [106, 107]. This process could also lead to potential germplasm sources which can be introgressed to improve marketable wheat varieties [108]. The results for short term abiotic stress (1 hour and 6 hours) indicate expression levels under temporary heat and drought stress, but more experimentation is need to determine expression under more sustained levels of stress. Variance in *AOX* expression and or copy number in wheat varieties with contrasting levels of resilience or tolerance to biotic or abiotic stress in a general or tissue-specific manner will facilitate the discovery of germplasm to create more marketable varieties. Importantly, *AOX* can have a strong effect on root morphology and may be upregulated roots under stress [109, 110]. Investigating the level of expression under infection by root pathogens and symbiotic soil microbes may aid in the elucidation of mechanism of susceptibility or resistance as well as symbiosis.

### Comparative analysis of wheat AOX proteins shows potential role of protein properties in functional diversity

The corresponding proteins obtained from the *AOX* transcripts in both the hexaploid and diploid wheat species were used in phylogenetic analysis in order to determine their classification to the clades AOX1a, AOX1c, AOX1e and AOX1d. Ten high-confidence hexaploid isoforms were classified as AOX1a, five as AOX1c, one as AOX1e and four as AOX1d. An additional two low-confidence hexaploid isoforms were in the AOX1a clade and one each in the AOX1e and AOX1d clades respectively (Figs 1 and S1). For the diploid *T*. *urartu*, one protein each was identified in the AOX1a and AOX1c clades and two in the AOX1d clades. In *A*. *tauschii*, one protein each was identified in the AOX1a and AOX1e clades and two in the AOX1d clades (Figs 1 and S1). Overall, the protein lengths ranged from 82 amino acids to 457 amino acids (Table 8). Generally, the theoretical isoelectric points of the AOX1d and AOX1e proteins were the lowest while that of the AOX1c proteins was the highest. In some cases, splice variant isoforms were shown to have distinct isoelectric points (TaAOX1a-2AL.sv1 and TaAOX1a-2AL.sv2; TaAOX1a-2DL.sv1 and TaAOX1a-2DL.sv2; regTaAOX-4BL.sv1, regTaAOX-4BL.sv2 and regTaAOX-4BL.sv3) (Table 8). Plants are able to alter their gene expression in response to external pH [111, 112]. The presence of protein isoforms with varying isoelectric points may therefore help with adaptability to external acidity or alkalinity. It has been experimentally shown that AOX may functionally substitute for the plastid terminal oxidase (PTOX) [113] and it is possible that in the polyploid and ancestral wheat genomes, some of the AOX proteins could perform non-canonical functions or play support roles for other organellar proteins. Different compartments have different acidic or basic environments levels [114] and if this flexibility in functionality works in the AOX proteins in wheat, protein isoforms with efficiencies in a pH range will aid in functionality in multiple cell organelles or compartments. In addition, the isoelectric point can affect protein localization [115, 116]. It is therefore plausible that the range of isoelectric points play a role in the determination of subcellular localization and functionality.

**Table 8:** Features of AOX proteins in the wheat genomes.

Protein Name	Protein Length (Amino Acids)	Molecular Weight (KDa)	Theoretical Isoelectric Point	Export Probability to Mitochondria
TaAOX1a-2AL.sv1	328	36.7	7.90	0.97
TaAOX1a-2AL.sv2	320	36.0	7.51	0.97
TaAOX1a-2BL	457	50.5	9.60	0.12
TaAOX1a-2DL.sv1	336	37.6	8.40	0.94
TaAOX1a-2DL.sv2	294	33.4	7.30	0.82
TaAOX1a-like-2DL	164	18.8	6.50	0.76
regTaAOX-4BL.sv1	108	12.7	10.90	0.93
regTaAOX-4BL.sv2	82	9.9	9.90	0.88
regTaAOX-4BL.sv3	88	10.6	10.10	0.91
regTaAOX-4BL.sv4	108	12.7	10.90	0.93
put.regTaAOX-3B	96	11.1	4.66	0.20
put.regTaAOX-6BL	146	15.5	8.58	0.79
TaAOX1c-6AL	397	43.7	9.80	0.64
TaAOX1c-6BL.sv1	431	47.5	10.20	0.29
TaAOX1c-6BL.sv2	412	45.5	10.30	0.28
TaAOX1c-6BL.sv3	431	47.6	10.20	0.29
TaAOX1c-6DL	395	43.7	9.70	0.57
regTaAOX-3B	107	12.1	6.70	0.77
put.TaAOX1e-3DS	262	30.0	6.97	0.08
TaAOX1d-2AL.1	294	33.5	7.20	0.17
TaAOX1d-2AL.2.sv1	330	37.1	7.20	0.81
TaAOX1d-2AL.2.sv2	330	37.1	7.20	0.81
TaAOX1d-2DL	326	36.7	7.20	0.85
put.TaAOX1d-like-4AS	183	21.0	7.79	0.25
TuAOX1a*	204	23.5	6.90	0.49
TuAOX1c*	434	49.8	5.90	0.15
TuAOX1d.1*	295	33.6	6.80	0.24
TuAOX1d.2*	403	44.6	9.90	0.21
AetAOX1a*	204	23.5	6.90	0.49
AetAOX1e*	365	40.8	7.40	0.49
AetAOX1d*	295	33.6	6.80	0.24
AetAOX1d-like*	289	32.6	8.60	0.58

The theoretical isoelectric points were obtained with the SnapGene Program and the mitochondrial localization probabilities were obtained with TargetP. A high value in the last column indicates a greater likelihood of localization to the mitochondria.

*Denotes diploid wheat AOX proteins.

Using the subcellular localization program TargetP, it was observed that there was a range of mitochondrial localization probabilities for AOX proteins. Generally, the hexaploid AOX1c clade had very low probability of export to the mitochondria and this trend continued with all the diploid isoforms (Table 8). Most prediction software focus on the N-terminal region of the protein in order to determine the subcellular localization. However an internal localization signal may exist as in the parasite *Trypanosoma brucei* (TbAOX) and in other nuclear-encoded plant proteins with no clear N-terminal signals [117, 118]. The region in TbAOX that contains this internal signal (residues 115 to 146) has some sequence similarity to the wheat isoforms but there are also clear differences, making it impossible to extrapolate outcomes from one species to another (S9 Fig). The charge of the amino acids in the sequence can also indicate the final protein destination and this may be further complicated by various protein modifications *in vivo* [119–121]. Given all the alternatives, it is clear that there is potential for a substantial amount of functional diversity and complexity, which could manifest as tissue or subcellular specificity as well as functional redundancy, some of which may require reporter fusions to dissect [122, 123]. This is plausible as it has been shown that AOX has both developmental and physiological functionality some of which may suggest species-specific or clade-specific functionalities [10, 31, 124].

Protein modifications such as phosphorylation, acetylation and glycosylation have been shown to be critical for multiple cellular processes in plants and it is possible that this may be the case with AOX as well [12, 71, 125–130]. Using the Plant Protein Phosphorylation Database and the Musite prediction program with a cut-off score of 0.5 as a baseline [68, 69], we found 14 predicted phosphorylation sites in TuAOX1a and two predicted phosphorylation sites in TuAOX1c (Table 9 and S7). There were two and one predicted acetylation sites in TaAOX1a-2DL.sv1 and TaAOX1d-2AL.1 respectively. There was one predicted glycosylation site each in TaAOX1a-2AL.sv1, TaAOX1a-2AL.sv2, TaAOX1a-2DL.sv1, put.regTaAOX-3B, TaAOX1c-6DL and TaAOX1d-2DL, and 28 sites in TaAOX1a-2DL.sv2. The dramatic difference in the number of predicted glycosylation sites between two splice variant isoforms (TaAOX1a-2DL.sv1 and TaAOX1a-2DL.sv2) (Tables 9 and S7) introduces the possibility of variable regulation and functionality which needs to be studied further.

**Table 9:** Putative post-translational modification sites in wheat AOX proteins.

**Protein**	**Phosphorylation Sites**
TuAOX1a*	T_9_, T_17_, T_34_, T_78_, T_131_, T_162_, T_168_, T_197_, S_12_, S_51_, S_58_, S_111_, S_129,_ S_185_,
TuAOX1c*	Y_145_, Y_214_
	**Acetylation Sites**
TaAOX1d-2AL.1	K_222_
TaAOX1a-2DL.sv1	K_59_, K_67_
TuAOX1d.2*	K_4_
AetAOX1d-like*	K_110_
	**Glycosylation**
TaAOX1a-2AL.sv1	S_113_
TaAOX1a-2AL.sv2	S_105_
TaAOX1a-2DL.sv1	S_121_
TaAOX1a-2DL.sv2	T_24,_ T_25_, T_61_, T_73,_ T_75,_ T_78,_ T_99,_ T_107,_ T_124,_ T_168,_ T_221,_ T_252,_ T_258,_ T_287,_ S_2,_ S_8,_ S_19,_ S_27,_ S_44,_ S_45,_ S_52,_ S_66,_ S_79,_ S_102,_ S_141,_ S_148,_ S_201,_ S_219_
put.regTaAOX-3B	S_11_
TaAOX1c-6DL	T_73_
TaAOX1d-2DL	S_111_
TuAOX1d.2*	S_188_
AetAOX1d*	S_80_

The predictions were obtained using Musite from the Plant Protein Phosphorylation Database.

*Indicates diploid isoforms.

### Molecular modeling depicts conservation of diiron center residues and isoform variance in transmembrane topology

**Fig 9:**
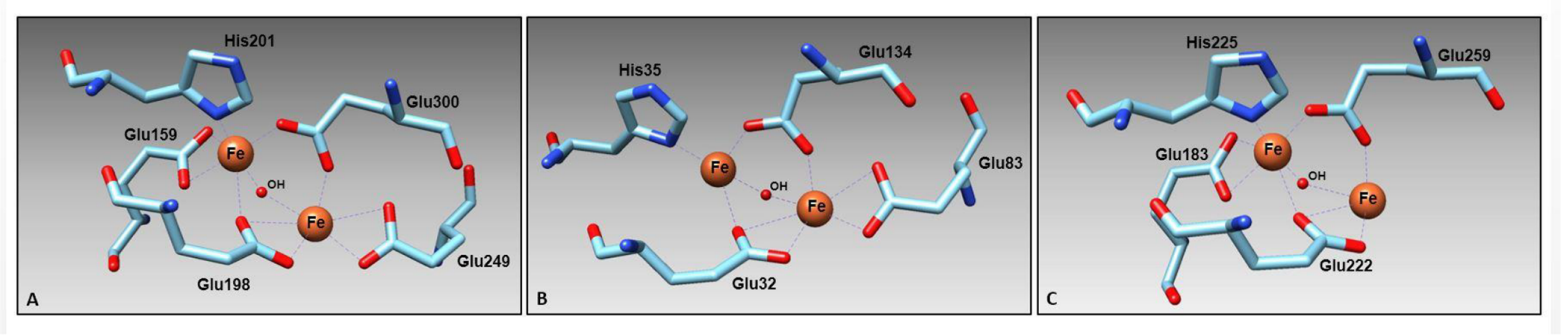
Proposed representative 3-D structure of the residues in the active site diiron center. (A) 3-D structural representation of TaAOX diiron center residues. (B) 3-D structural representation of TaAOX1a-like-2DL diiron center residues. (C) 3-D structural representation of AetAOX1d-like diiron center residues.

In order to determine the three-dimensional structure of the wheat AOX isomers, homology models of wheat AOX proteins were made using the crystal structure of TbAOX as a reference (model 3vvaD in Phyre2). With the exception of the low-confidence protein put.regTaAOX-6BL, all other proteins modeled to the TbAOX with over 95% confidence, sequence identity between 34 to 46% and coverage ranging from 20% to 96% depending on the isoform (range: 82 to 457 amino acids). These results offer a preliminary understanding of the structure of these proteins in wheat (Fig 9 and S8 and S9 Tables and S1 Appendix). The proteins which had most or all the motifs required for the diiron center were modeled with a similar global conformation and active site configuration indicating a likely similarity in three-dimensional structural conformation (Fig 9 and S10 Table and S1 Appendix).

**Fig 10:**
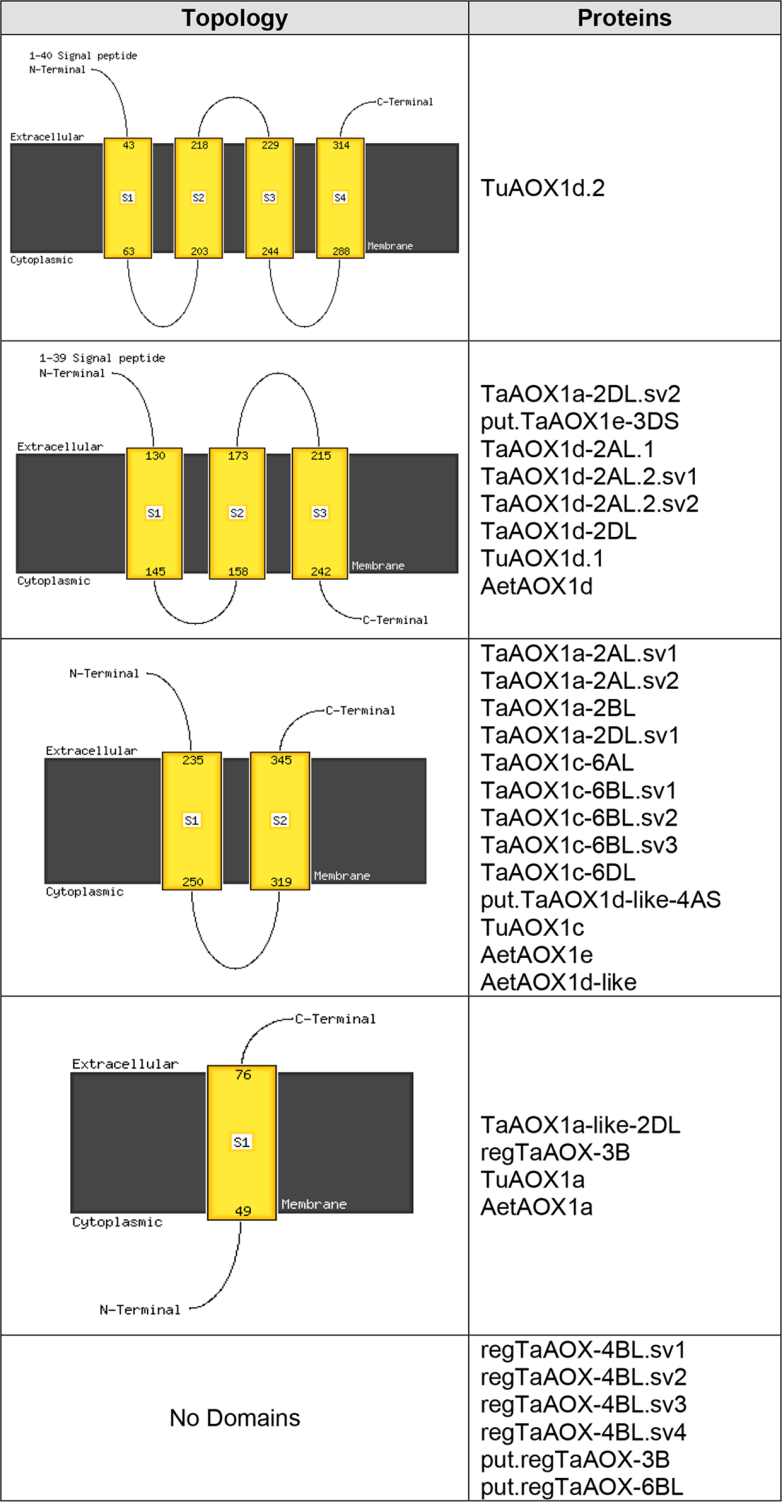
Transmembrane topologies of wheat AOX proteins predicted by Phyre2.

Regardless of clade, most of the modeled wheat AOX proteins had one to three transmembrane domains except regTaAOX-4BL.sv1 to sv4, put.regTaAOX-3B and put.regTaAOX-6BL which had none. Notably, TuAOX1d.2 had four transmembrane domains, the highest of all the proteins analyzed (Fig 10 and S8 and S9 Tables). TaAOX1a-like-2DL, regTaAOX-3B and TuAOX1a had the smallest number of transmembrane domains (Fig 10 and S8 and S9 Tables). Mirroring the observation of heterogeneity in protein properties between splice variant isoforms earlier observed, TaAOX1a-2DL.sv1 and TaAOX1a-2DL.sv2 had two and three transmembrane domains respectively, an example of the phenotypic diversity resulting from alternative splicing which could suggest functional diversification. Transmembrane domains have been shown to be key in the determination of protein localization in plants [131, 132] and the differences observed in wheat may facilitate the functional characterization of these proteins in the future.

### Comparative analysis of wheat AOX proteins and identification of key functional residues in critical domains

**Fig 11:**
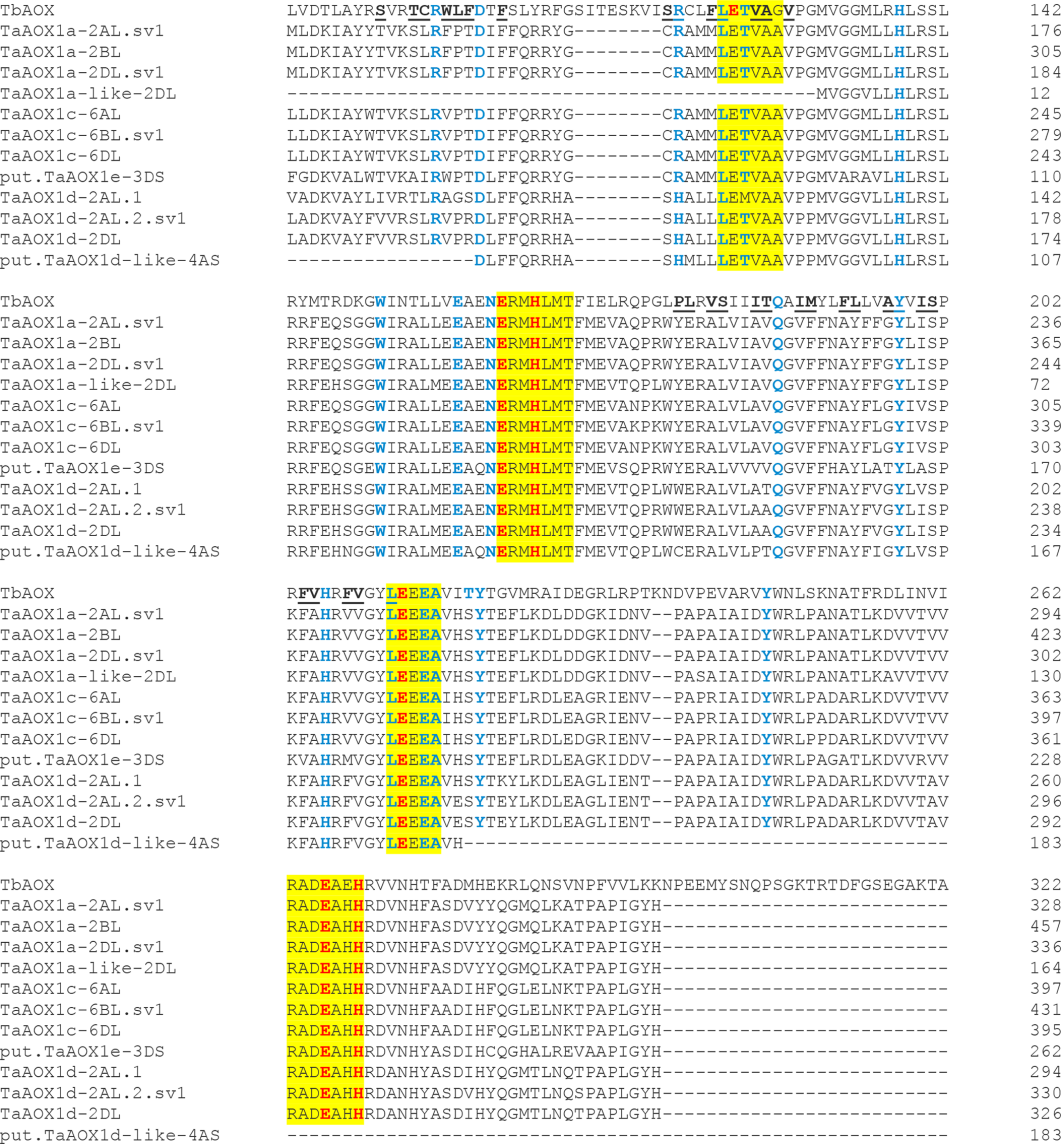
Alignment of select TaAOX (hexaploid wheat) proteins with TbAOX (*T*. *brucei*). Yellow highlights indicate conserved motifs. Red font indicates residues proposed to coordinate the diiron center of the active site. Blue font indicates residues experimentally tested for loss of activity by previous researchers. Underlined residues are involved in the TbAOX hydrophobic cavity. Splice variants were identical for the protein region analyzed. The “reg” proteins were not analyzed due to the absence of the conserved motifs.

**Fig 12:**
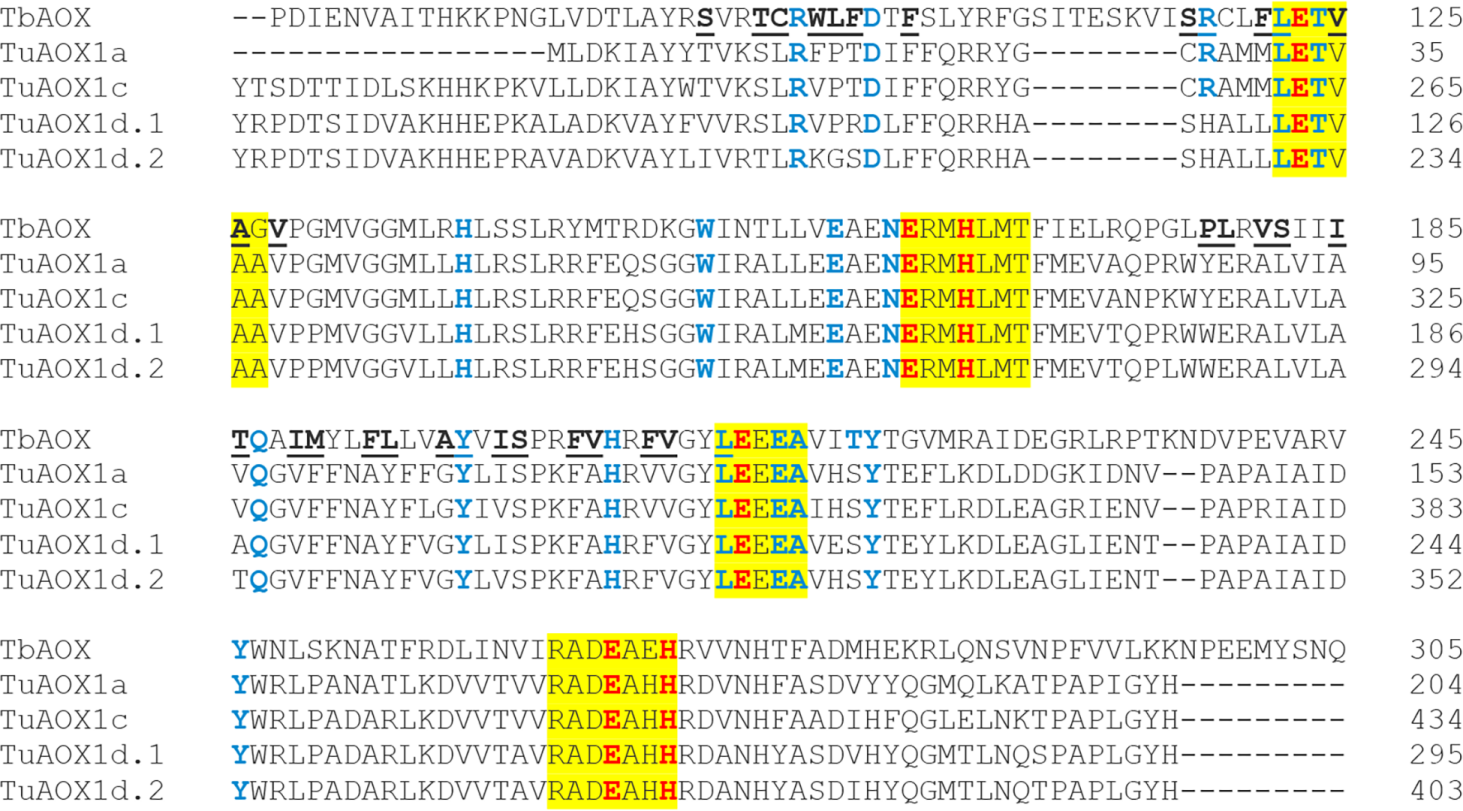
Alignment of TuAOX (*T*. *urartu*) proteins with TbAOX (*T*. *brucei*). Yellow highlights indicate conserved motifs. Red font indicates residues proposed to coordinate the diiron center of the active site. Blue font indicates residues experimentally tested for loss of activity by previous researchers. Underlined residues are involved in the TbAOX hydrophobic cavity.

**Fig 13:**
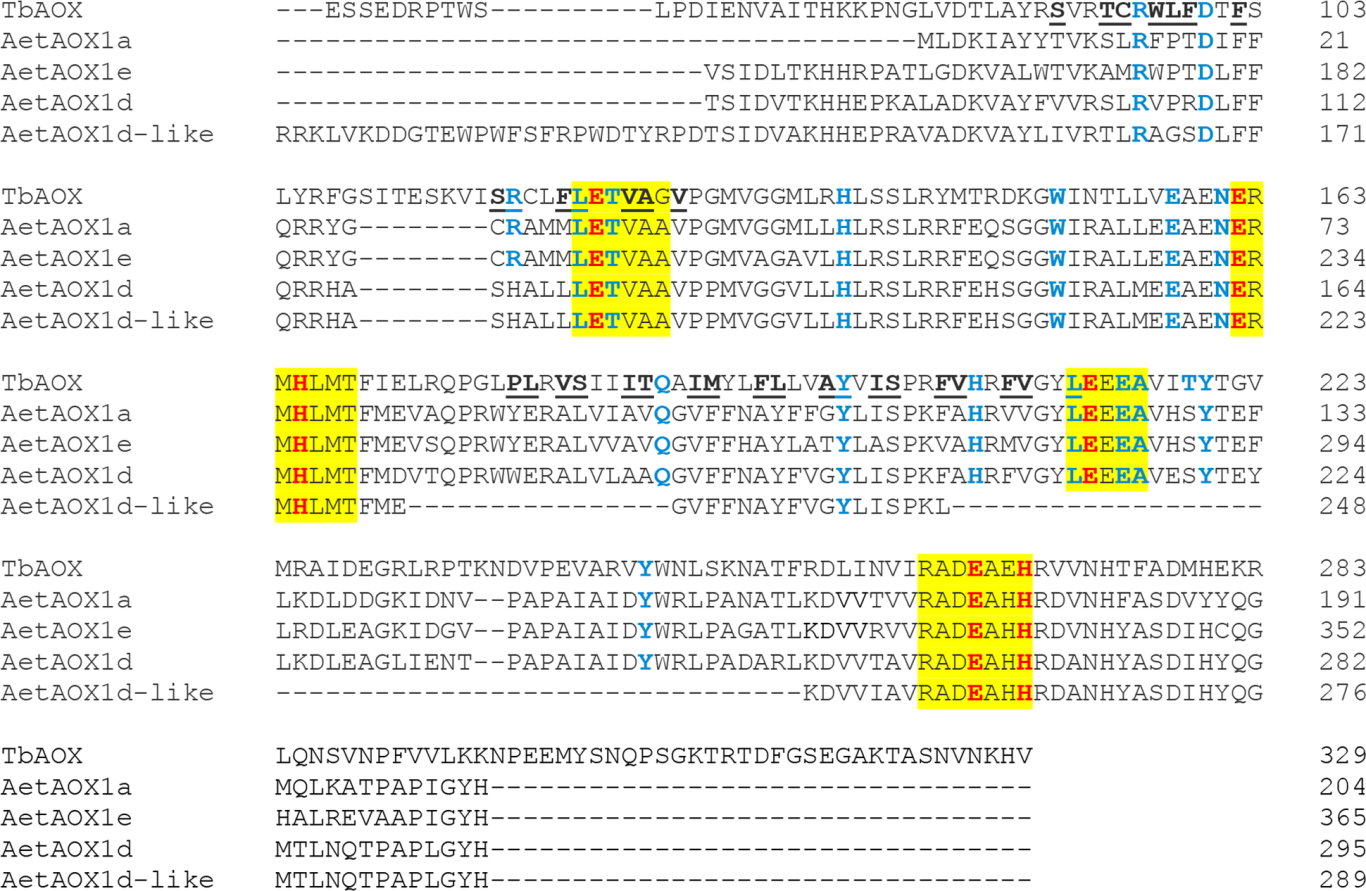
Alignment of AetAOX (*A*. *tauschii*) proteins with TbAOX (*T*. *brucei*). Yellow highlights indicate conserved motifs. Red font indicates residues proposed to coordinate the diiron center of the active site. Blue font indicates residues experimentally tested for loss of activity by previous researchers. Underlined residues are involved in the TbAOX hydrophobic cavity.

To identify conserved amino acid residues required for AOX function, the corresponding proteins of all wheat *AOX* transcripts and splice variants were aligned with the AOX sequence from the parasite *Trypanosoma brucei* (TbAOX) [133] (Figs 11–13; S2 Table; S2 Fig). Signature motifs required for AOX functionality via the diiron center, LETVAA, ERMHLMT, LEEEA and RADEAHH, were found in 22 out of 32 protein sequences (S2 Table). The protein TaAOX1a-like-2DL, lacked the first motif LETVAA, another put.TaAOX1d-like-4AS lacked the motif RADEAHH and a third AetAOX1d-like lacked the motif LEEEA. The protein TaAOX1d-2AL.1, had a slight modification LEMVAA which nevertheless conserved the glutamate critical for the diiron center. All the regulatory proteins lacked all four of these trademark motifs (Figs 11–13; S2 Table; S2 Fig). Previous research shows that mutations in any of the residues needed for coordinating the diiron center in the active site cause a partial or complete attenuation of AOX activity (Figs 11–13 and S11 Table) [12, 133–136]. This could suggest low or abolished activity for TaAOX1a-like-2DL, put.TaAOX1d-like-4AS and AetAOX1d-like which lack the motifs LETVAA, RADEAHH and LEEEA respectively, and consequently the critical glutamate residues needed to coordinate the diiron center (Figs 11 and 13; S11 and S12 Tables). The highly conserved threonine residue on other AOX proteins is a methionine in TaAOX1d-2AL.1 (TbAOX number scheme T124) (Fig 11). The difference in polarity between threonine (polar) and methionine (nonpolar) may have implications for enzyme activity and functionality. In the recombinant *S*. *guttatum* AOX protein (rSgAOX) that was tested, T179A substituted mutant (TbAOX number scheme T124) had severely reduced activity [134]. It is plausible that the same reduced enzyme activity could be observed in TaAOX1d-2AL.1. However, it must be noted that even though the hydrophobicity of the substitution in wheat mirrors that of rSgAOX T179A, the effect of the conformational change on AOX efficiency needs to be experimentally established in order to confirm an identical reduction in function.

A T219V mutation, which leads to a significant change in side chain chemistry and configuration, causes an almost complete loss of function in recombinant *T*. *brucei* AOX (rTbAOX) (S11 Table) [133]. In wheat, there is a T219S (TbAOX number scheme) substitution conserved in all the diploid and hexaploid AOX except regTaAOX-4BL, regTaAOX-3B, put.TaAOX1d-like-4AS and AetAOX1d-like where this residue is nonexistent (Figs 11 and 13; S12 Table). The substitution maintains side chain properties but the effect of the lost methyl group on the enzymatic outcome remains to be determined. The proteins TaAOX1a-like-2DL, put.TaAOX1d-like-4AS and AetAOX1d-like are missing residues which have been experimentally shown to greatly reduce or abolish activity (S12 Table). Where nonexistent, it may imply an alternate protein configuration in that region which may change the enzyme efficiency or allow for high efficiency in a distinct role.

The crystal structure of TbAOX shows that this protein exists as a homodimer. At the dimer interface in TbAOX, there are six completely conserved residues and 12 highly conserved residues [133] some of which show significant loss of activity when mutated (H138, Q187) (S11 Table). Excluding AetAOX1d-like, these six residues are completely conserved in wheat AOX proteins as well (Table 10). With regards to the 12 highly conserved residues for the dimer interface, six are identical to TbAOX (M131, L139, S141, A159, M167, R180) except in the case of AetAOX1d-like (Tables 10 and S13). This high level of conservation across species emphasizes the importance of these residues in this functional capacity. There were three substitutions that were peculiar to the AOX1d clade and the AOX1a-like proteins (M135V, R147H, L156M). There were also one substitutions that were conserved in all wheat clades (M145F) (Tables 10 and S13). Two other substitutions (I183V, D148S) are also conserved in all the wheat clades with the exception of put.TaAOX1d-like-4AS (D148N) and AetAOX1d-like (I183V is nonexistent) (Tables 10 and S13). None of these conserved substitutions match any of the substitutions thought to support an AOX Type AOX1d classification done by other researchers [16] and thus may indicate a divergence peculiar to this AOX clade in wheat which could inform function. These substitutions need further characterization in order to test their effect on AOX dimerization and efficiency in wheat.

**Table 10:** Comparison of residues at dimerization interface between the TbAOX and wheat AOX proteins.

TbAOX Residue Numbers																		
	Completely Conserved in TbAOX						Highly Conserved in TbAOX											
	H138◆	L142	R143	R163	L166	Q187◆	M131	M135	L139	S141	M145	R147	D148	L156	A159	M167	R180	I183
TaAOX1a-2AL.sv1	H	L	R	R	L	Q	M	M	L	S	F	Q	S	L	A	M	R	V
TaAOX1a-2AL.sv2	H	L	R	R	L	Q	M	M	L	S	F	Q	S	L	A	M	R	V
TaAOX1a-2BL	H	L	R	R	L	Q	M	M	L	S	F	Q	S	L	A	M	R	V
TaAOX1a-2DL.sv1	H	L	R	R	L	Q	M	M	L	S	F	Q	S	L	A	M	R	V
TaAOX1a-2DL.sv2	H	L	R	R	L	Q	M	M	L	S	F	Q	S	L	A	M	R	V
TaAOX1a-like-2DL	H	L	R	R	L	Q	M	V	L	S	F	H	S	M	A	M	R	V
TaAOX1c-6AL	H	L	R	R	L	Q	M	M	L	S	F	Q	S	L	A	M	R	V
TaAOX1c-6BL.sv1	H	L	R	R	L	Q	M	M	L	S	F	Q	S	L	A	M	R	V
TaAOX1c-6BL.sv2	H	L	R	R	L	Q	M	M	L	S	F	Q	S	L	A	M	R	V
TaAOX1c-6BL.sv3	H	L	R	R	L	Q	M	M	L	S	F	Q	S	L	A	M	R	V
TaAOX1c-6DL	H	L	R	R	L	Q	M	M	L	S	F	Q	S	L	A	M	R	V
put.TaAOX1e-3DS	H	L	R	R	L	Q	M	A	L	S	F	Q	S	L	A	M	R	V
TaAOX1d-2AL.1	H	L	R	R	L	Q	M	V	L	S	F	H	S	M	A	M	R	V
TaAOX1d-2AL.2.sv1	H	L	R	R	L	Q	M	V	L	S	F	H	S	M	A	M	R	V
TaAOX1d-2AL.2.sv2	H	L	R	R	L	Q	M	V	L	S	F	H	S	M	A	M	R	V
TaAOX1d-2DL	H	L	R	R	L	Q	M	V	L	S	F	H	S	M	A	M	R	V
put.TaAOX1d-like-4AS	H	L	R	R	L	Q	M	V	L	S	F	H	N	M	A	M	R	V
TuAOX1a*	H	L	R	R	L	Q	M	M	L	S	F	Q	S	L	A	M	R	V
TuAOX1c*	H	L	R	R	L	Q	M	M	L	S	F	Q	S	L	A	M	R	V
TuAOX1d.1*	H	L	R	R	L	Q	M	V	L	S	F	H	S	M	A	M	R	V
TuAOX1d.2*	H	L	R	R	L	Q	M	V	L	S	F	H	S	M	A	M	R	V
AetAOX1a*	H	L	R	R	L	Q	M	M	L	S	F	Q	S	L	A	M	R	V
AetAOX1e*	H	L	R	R	L	Q	M	A	L	S	F	Q	S	L	A	M	R	V
AetAOX1d*	H	L	R	R	L	Q	M	V	L	S	F	H	S	M	A	M	R	V
AetAOX1d-like*	H	L	R	R	L	-	M	V	L	S	F	H	S	M	A	M	-	-

Completely conserved residues (yellow), highly conserved (green), and semi-conserved (white) in the wheat AOX family are shown. The “reg” proteins were not analyzed due to the absence of the functionally necessary motifs.

*Denotes diploid wheat AOX proteins.

**◆**Denotes residues which have been experimentally tested.

Another highly essential domain is the hydrophobic cavity consisting of 33 residues known to facilitate quinol-binding in the TbAOX active site (Table 11) [135]. Except in the cases of TaAOX1a-like-2DL, put.TaAOX1d-like-4AS and AetAOX1d-like, nine out of the 33 residues show complete conservation with TbAOX (F102, L122, V125, A126, V128, Y198, S201, V209, L212) emphasizing the crucial nature of these residues in active site efficiency (Tables 11 and S14). With the exception of put.TaAOX1d-like-4AS and AetAOX1d-like, there are eight substitutions which are conserved in all the wheat residues in this hydrophobic cavity (L179E, V181A, S182L, I189V, M190F, F193A, L194Y, V205A) (Tables 11 and S14). The AOX1e clade also has distinct residues or substitutions (T94A, C95M or C95I, W97, A197T, I200A, F204V, F208M) (Tables 11 and S14). With the exception of AetAOX1d-like, there are five residues or substitutions conserved in all wheat AOX1d proteins (S117, R118H, F121L, P178W, F208) (Tables 11 and S14). In addition, there are residues or substitutions peculiar to AOX1d group 1 (S91I, T94, L98G, F99S, T186) or AOX1d group 2 (S91V, F99R, T186A) efficiency (Fig 1; Tables 11 and S14). Substitutions of some of these residues have been shown to cause partial or complete attenuation of AOX activity (R118, L122, Y198 and L212) (Tables 11, S11 and S12). In rTbAOX, R118A and R118Q abolish nearly all AOX function even though some side chain chemistry is conserved for the latter mutation (S11 Table) [133]. The observation that R118H is conserved in one wheat AOX clade suggests that this substitution may be important for how the Type AOX1d isoforms function in wheat and the R118H mutation should be studied in this context.

**Table 11:** Comparison of residues in the hydrophobic cavity of the TbAOX and wheat AOX proteins.

TbAOX Residue Numbers in Hydrophobic Cavity																																	
	**S91**	**T94**	**C95**	**W97**	**L98**	**F99**	**F102**	**S117**	**R118◆**	**F121**	**L122◆**	**V125**	**A126**	**V128**	**P178**	**L179**	**V181**	**S182**	**I185**	**T186**	**I189**	**M190**	**F193**	**L194**	**A197**	**Y198◆**	**I200**	**S201**	**F204**	**V205**	**F208**	**V209**	**L212◆**
TaAOX1a-2AL.sv1	**T**	**S**	**L**	**F**	**P**	**T**	**F**	**C**	**R**	**M**	**L**	**V**	**A**	**V**	**Y**	**E**	**A**	**L**	**A**	**V**	**V**	**F**	**A**	**Y**	**G**	**Y**	**I**	**S**	**F**	**A**	**V**	**V**	**L**
TaAOX1a-2AL.sv2	**T**	**S**	**L**	**F**	**P**	**T**	**F**	**C**	**R**	**M**	**L**	**V**	**A**	**V**	**Y**	**E**	**A**	**L**	**A**	**V**	**V**	**F**	**A**	**Y**	**G**	**Y**	**I**	**S**	**F**	**A**	**V**	**V**	**L**
TaAOX1a-2BL	**T**	**S**	**L**	**F**	**P**	**T**	**F**	**C**	**R**	**M**	**L**	**V**	**A**	**V**	**Y**	**E**	**A**	**L**	**A**	**V**	**V**	**F**	**A**	**Y**	**G**	**Y**	**I**	**S**	**F**	**A**	**V**	**V**	**L**
TaAOX1a-2DL.sv1	**T**	**S**	**L**	**F**	**P**	**T**	**F**	**C**	**R**	**M**	**L**	**V**	**A**	**V**	**Y**	**E**	**A**	**L**	**A**	**V**	**V**	**F**	**A**	**Y**	**G**	**Y**	**I**	**S**	**F**	**A**	**V**	**V**	**L**
TaAOX1a-2DL.sv2	**T**	**S**	**L**	**F**	**P**	**T**	**F**	**C**	**R**	**M**	**L**	**V**	**A**	**V**	**Y**	**E**	**A**	**L**	**A**	**V**	**V**	**F**	**A**	**Y**	**G**	**Y**	**I**	**S**	**F**	**A**	**V**	**V**	**L**
TaAOX1a-like-2DL	**-**	**-**	**-**	**-**	**-**	**-**	**-**	**-**	**-**	**-**	**-**	**-**	**-**	**-**	**Y**	**E**	**A**	**L**	**A**	**V**	**V**	**F**	**A**	**Y**	**G**	**Y**	**V**	**S**	**F**	**A**	**V**	**V**	**L**
TaAOX1c-6AL	**T**	**S**	**L**	**V**	**P**	**T**	**F**	**C**	**R**	**M**	**L**	**V**	**A**	**V**	**Y**	**E**	**A**	**L**	**A**	**V**	**V**	**F**	**A**	**Y**	**G**	**Y**	**I**	**S**	**F**	**A**	**V**	**V**	**L**
TaAOX1c-6BL.sv1	**T**	**S**	**L**	**V**	**P**	**T**	**F**	**C**	**R**	**M**	**L**	**V**	**A**	**V**	**Y**	**E**	**A**	**L**	**A**	**V**	**V**	**F**	**A**	**Y**	**G**	**Y**	**V**	**S**	**F**	**A**	**V**	**V**	**L**
TaAOX1c-6BL.sv2	**T**	**S**	**L**	**V**	**P**	**T**	**F**	**C**	**R**	**M**	**L**	**V**	**A**	**V**	**Y**	**E**	**A**	**L**	**A**	**V**	**V**	**F**	**A**	**Y**	**G**	**Y**	**V**	**S**	**F**	**A**	**V**	**V**	**L**
TaAOX1c-6BL.sv3	**T**	**S**	**L**	**V**	**P**	**T**	**F**	**C**	**R**	**M**	**L**	**V**	**A**	**V**	**Y**	**E**	**A**	**L**	**A**	**V**	**V**	**F**	**A**	**Y**	**G**	**Y**	**V**	**S**	**F**	**A**	**V**	**V**	**L**
TaAOX1c-6DL	**T**	**S**	**L**	**V**	**P**	**T**	**F**	**C**	**R**	**M**	**L**	**V**	**A**	**V**	**Y**	**E**	**A**	**L**	**A**	**V**	**V**	**F**	**A**	**Y**	**G**	**Y**	**V**	**S**	**F**	**A**	**V**	**V**	**L**
Put.TaAOX1e-3DS	**T**	**A**	**I**	**W**	**P**	**T**	**F**	**C**	**R**	**M**	**L**	**V**	**A**	**V**	**Y**	**E**	**A**	**L**	**V**	**V**	**V**	**F**	**A**	**Y**	**T**	**Y**	**A**	**S**	**V**	**A**	**M**	**V**	**L**
TaAOX1d-2AL.1	**I**	**T**	**L**	**A**	**G**	**S**	**F**	**S**	**H**	**L**	**L**	**V**	**A**	**V**	**W**	**E**	**A**	**L**	**A**	**T**	**V**	**F**	**A**	**Y**	**G**	**Y**	**V**	**S**	**F**	**A**	**F**	**V**	**L**
TaAOX1d-2AL.2.sv1	**V**	**S**	**L**	**V**	**P**	**R**	**F**	**S**	**H**	**L**	**L**	**V**	**A**	**V**	**W**	**E**	**A**	**L**	**A**	**A**	**V**	**F**	**A**	**Y**	**G**	**Y**	**I**	**S**	**F**	**A**	**F**	**V**	**L**
TaAOX1d-2AL.2.sv2	**V**	**S**	**L**	**V**	**P**	**R**	**F**	**S**	**H**	**L**	**L**	**V**	**A**	**V**	**W**	**E**	**A**	**L**	**A**	**A**	**V**	**F**	**A**	**Y**	**G**	**Y**	**I**	**S**	**F**	**A**	**F**	**V**	**L**
TaAOX1d-2DL	**V**	**S**	**L**	**V**	**P**	**R**	**F**	**S**	**H**	**L**	**L**	**V**	**A**	**V**	**W**	**E**	**A**	**L**	**A**	**A**	**V**	**F**	**A**	**Y**	**G**	**Y**	**I**	**S**	**F**	**A**	**F**	**V**	**L**
put.TaAOX1d-like-4AS	**-**	**-**	**-**	**-**	**-**	**-**	**F**	**S**	**H**	**L**	**L**	**V**	**A**	**V**	**C**	**E**	**A**	**L**	**P**	**T**	**V**	**F**	**A**	**Y**	**G**	**Y**	**V**	**S**	**F**	**A**	**F**	**V**	**L**
TuAOX1a*	**T**	**S**	**L**	**F**	**P**	**T**	**F**	**C**	**R**	**M**	**L**	**V**	**A**	**V**	**Y**	**E**	**A**	**L**	**A**	**V**	**V**	**F**	**A**	**Y**	**G**	**Y**	**I**	**S**	**F**	**A**	**V**	**V**	**L**
TuAOX1c*	**T**	**S**	**L**	**V**	**P**	**T**	**F**	**C**	**R**	**M**	**L**	**V**	**A**	**V**	**Y**	**E**	**A**	**L**	**A**	**V**	**V**	**F**	**A**	**Y**	**G**	**Y**	**V**	**S**	**F**	**A**	**V**	**V**	**L**
TuAOX1d.1*	**V**	**S**	**L**	**V**	**P**	**R**	**F**	**S**	**H**	**L**	**L**	**V**	**A**	**V**	**W**	**E**	**A**	**L**	**A**	**A**	**V**	**F**	**A**	**Y**	**G**	**Y**	**I**	**S**	**F**	**A**	**F**	**V**	**L**
TuAOX1d.2*	**I**	**T**	**L**	**K**	**G**	**S**	**F**	**S**	**H**	**L**	**L**	**V**	**A**	**V**	**W**	**E**	**A**	**L**	**A**	**T**	**V**	**F**	**A**	**Y**	**G**	**Y**	**V**	**S**	**F**	**A**	**F**	**V**	**L**
AetAOX1a*	**T**	**S**	**L**	**F**	**P**	**T**	**F**	**C**	**R**	**M**	**L**	**V**	**A**	**V**	**Y**	**E**	**A**	**L**	**A**	**V**	**V**	**F**	**A**	**Y**	**G**	**Y**	**I**	**S**	**F**	**A**	**V**	**V**	**L**
AetAOX1e*	**T**	**A**	**M**	**W**	**P**	**T**	**F**	**C**	**R**	**M**	**L**	**V**	**A**	**V**	**Y**	**E**	**A**	**L**	**A**	**V**	**V**	**F**	**A**	**Y**	**T**	**Y**	**A**	**S**	**V**	**A**	**M**	**V**	**L**
AetAOX1d*	**V**	**S**	**L**	**V**	**P**	**R**	**F**	**S**	**H**	**L**	**L**	**V**	**A**	**V**	**W**	**E**	**A**	**L**	**A**	**A**	**V**	**F**	**A**	**Y**	**G**	**Y**	**I**	**S**	**F**	**A**	**F**	**V**	**L**
AetAOX1d-like*	**I**	**T**	**L**	**A**	**G**	**S**	**F**	**S**	**H**	**L**	**L**	**V**	**A**	**V**	**-**	**-**	**-**	**-**	**-**	**-**	**V**	**F**	**A**	**Y**	**G**	**Y**	**I**	**S**	**L**	**-**	**-**	**-**	**-**

Green represents polar residues while red represents hydrophobic residues. Yellow represents residues with cyclic side-chains, and gray represent glycine. The “reg” proteins were not analyzed due to the absence of the functionally necessary motifs.

*Denotes diploid wheat AOX proteins.

**◆**Denotes residues which have been experimentally tested.

The ratios of hydrophobic to polar residues have been shown to be critical in how this cavity binds to substrates in the active site. With the exception of AOX1d group 1 and TaAOX1a-like-2DL, it was very clear that the ratios of sidechain chemistries were conserved in the clades (S15 Table). Functionally, these permutations in the wheat AOX amino acid sequences may suggest a gradient of enzyme function and activity which can be explored in the future. The absence of a significant number of these conserved hydrophobic-cavity residues in the TaAOX1a-like-2DL and AetAOX1d-like proteins (Table 11) may suggest distinct physiological roles for these isoforms such as interaction with other isoforms or proteins to exert control of subcellular localization or processes [137–139]. The situation in wheat may be similar to what has been suggested in other organisms with unique environments and physiologies, where residues critical for the configuration and stability of the active site or hydrophobic cavity in one species may be negligible in another [135, 140].

The information garnered could provide clues on how these proteins function in a polyploid monocot or grass species in general and highlights the urgent need for the biochemical elucidation of more AOX isoforms in diverse plant species. Given the similarities that exist within the wheat AOX isoforms especially those obtained via alternative splicing, it may be helpful to try to dissect how they function in different biological contexts (S16 Table). This can be done by mutation studies to study the effect of the substitutions or deletions, and biochemical methods which detect isoforms as well as posttranslational modifications which may change how and where these isomers function [141, 142].

## Conclusions

Exploiting the structure of AOX holds potential for treating both human and plant diseases [135, 143, 144]. For plants, this is of paramount importance as there is the need to maintain or improve yield of important food and cash crops [145]. Elucidating the structure of AOX proteins in wheat may allow for many possibilities in addition to those already stated in this study. In *A*. *thaliana*, AOX induction has been shown to be a consequence of herbicide toxicity rather than evidence for tolerance [146]; however, the effect may be different in monocots. One could potentially exploit differences in the active site or hydrophobic substrate-binding cavities between hexaploid wheat isoforms and diploid wheat isoforms in order to more effectively design herbicides which affect diploid, wild and weedy ancestral wheat while allowing optimal growth of domesticated wheat which is used for food. The same approach can be used for designing better herbicides against other monocot and dicot weeds which can have a devastating effect on crop yield [147, 148]. On the other hand, the domestication of wheat has caused a loss in alleles which may be beneficial for yield. It may be advantageous to explore AOX structures in wild relatives and select those with the dual advantages of high expression and efficiency [149, 150]. The alleles for these isoforms that then correlate with resistance or tolerance to various forms of biotic and abiotic stress could then be introgressed into marketable wheat varieties or used as a template in order to effect gene-editing in highly profitable hexaploid wheat [151, 152]. In general, the TbAOX has been shown to have the best efficiency [135] but this may change as more plant AOX structures from organisms such as extremophiles and other monocots and dicots with variant ploidy are genetically and biochemically studied. The identification of the AOX gene family in wheat will contribute towards this process.

## Supporting information

S1 Fig Bayesian phylogenetic tree generated for select AOX sequences in order to determine clades. The number of splice variant isomers for a protein are denoted in the dark gray circle when applicable. Colored boxes distinguish the different AOX clades.
(PDF)

S2 Fig Select AOX protein sequences used in this study. (PDF)

S3 Fig Alignment of wheat AOX sequences with *A*. *thaliana* AOX1a in order to determine classification. Blue indicates presence of Type 1 residues. Red indicates a Type 2 residue. Green indicates residues for monocot Type 1(d). Yellow indicates Type 1(a-c/e). Purple represents amino acid residues that did not match either classification. Black represents residues that were absent.
(PDF)

S4 Fig Nucleotide sequences of *AOX* from hexaploid and diploid wheat. (PDF)

S5 Fig Alignment of coding sequence of high-confidence hexaploid *TaAOX1d-2AL*.*2*.*sv1* wheat with the non-expressed coding sequences. (PDF)

S6 Fig Alignment of high-confidence protein sequence of hexaploid TaAOX1d-2AL.2.sv1 protein sequence with the non-expressed protein sequences. (PDF)

S7 Fig Alignment and phylogeny of Waox1a and Waox1c proteins with closest hexaploid wheat relatives used in this study. (PDF)

S8 Fig Distribution of select *TaAOX* genes on the respective chromosomes. Diagram not to scale.
(PDF)

S9 Fig Comparison of TbAOX internal mitochondrial targeting peptide sequence with TaAOX residues in similar regions. The “reg” proteins were not analyzed due to absence of the amino acids within this region.
(PDF)

S1 Table Accessions numbers of AOX proteins used in this study. RC in the wheat sequences refers to sequences which had high sequence similarity in the reverse complement from which the protein was translated.
(XLSX)

S2 Table Conserved AOX motifs in the wheat protein isoforms. The highlighted residues in the motif are critical in the active site diiron center. X indicates presence of motifs. *Indicates diploid wheat isoforms.
(PDF)

S3 Table Regulators and motifs in *AOX* expression. *Denotes negative regulators of *AOX* expression.
(PDF)

S4 Table Sequences of putative wheat AOX regulatory motifs. The nucleotide sequence in bold indicates cases where the motif *YTTGNNNNNVAMV* has a single nucleotide deviation (site of change underlined) from the MDM motif *CTTGNNNNNCAMG*. *Denotes negative regulators of *AOX* expression.
(XLSX)

S5 Table Expression data of high-confidence *TaAOX* gene family over multiple developmental stages, biotic and abiotic stress. (XLSX)

S6 Table Percent identities between the transcripts of all expressed wheat genes identified in this study. (XLSX)

S7 Table Putative post-translational modification sites in wheat AOX proteins. (XLSX)

S8 Table Summary of TaAOX 3-D structures obtained with Phyre2. The model used was c3vvaD. *Models to c3rylB.
(PDF)

S9 Table Summary of diploid AOX 3-D structures (TuAOX and AetAOX) obtained with Phyre2. The model used was c3vvaD.
(PDF)

S10 Table Comparison of active site diiron residues between TbAOX and wheat AOX. *Denotes diploid AOX proteins.
(PDF)

S11 Table Critical TbAOX residues known to cause reduction in activity when mutagenized. The residues that are not highlighted are from Moore *et al*. 2013; red highlights are residues from Shiba *et al*. 2013; green highlights are residues from Young *et al*. 2014 and Crichton *et al*. 2010.
(PDF)

S12 Table Summary of residues of wheat AOX proteins in the context of AOX residues which have been experimentally determined to have reduced activity when mutagenized. The residues that are not highlighted are from Moore *et al*. 2013; red highlights are residues from Shiba *et al*. 2013; green highlights are residues from Young *et al*. 2014 and Crichton *et al*. 2010.
(XLSX)

S13 Table Summary of wheat AOX residues in the dimerization domain. (PDF)

S14 Table Summary of wheat AOX residues in the hydrophobic cavity. (PDF)

S15 Table Summary of ratios of hydrophobic, polar, and cyclic residues in the hydrophobic domain of wheat AOX proteins. (XLSX)

S16 Table Summary of percent identities within the wheat AOX isoforms. (XLSX)

S1 Appendix Wheat protein modeling files generated from Phyre2. (ZIP)
