# Supplementary figures and images for: Genome-wide identification and analysis of the ALTERNATIVE OXIDASE gene family in diploid and hexaploid wheat
Source: PLoS One. 2018 Aug 3;13(8):e0201439. doi: 10.1371/journal.pone.0201439 (PMC6075773; doi:10.1371/journal.pone.0201439)

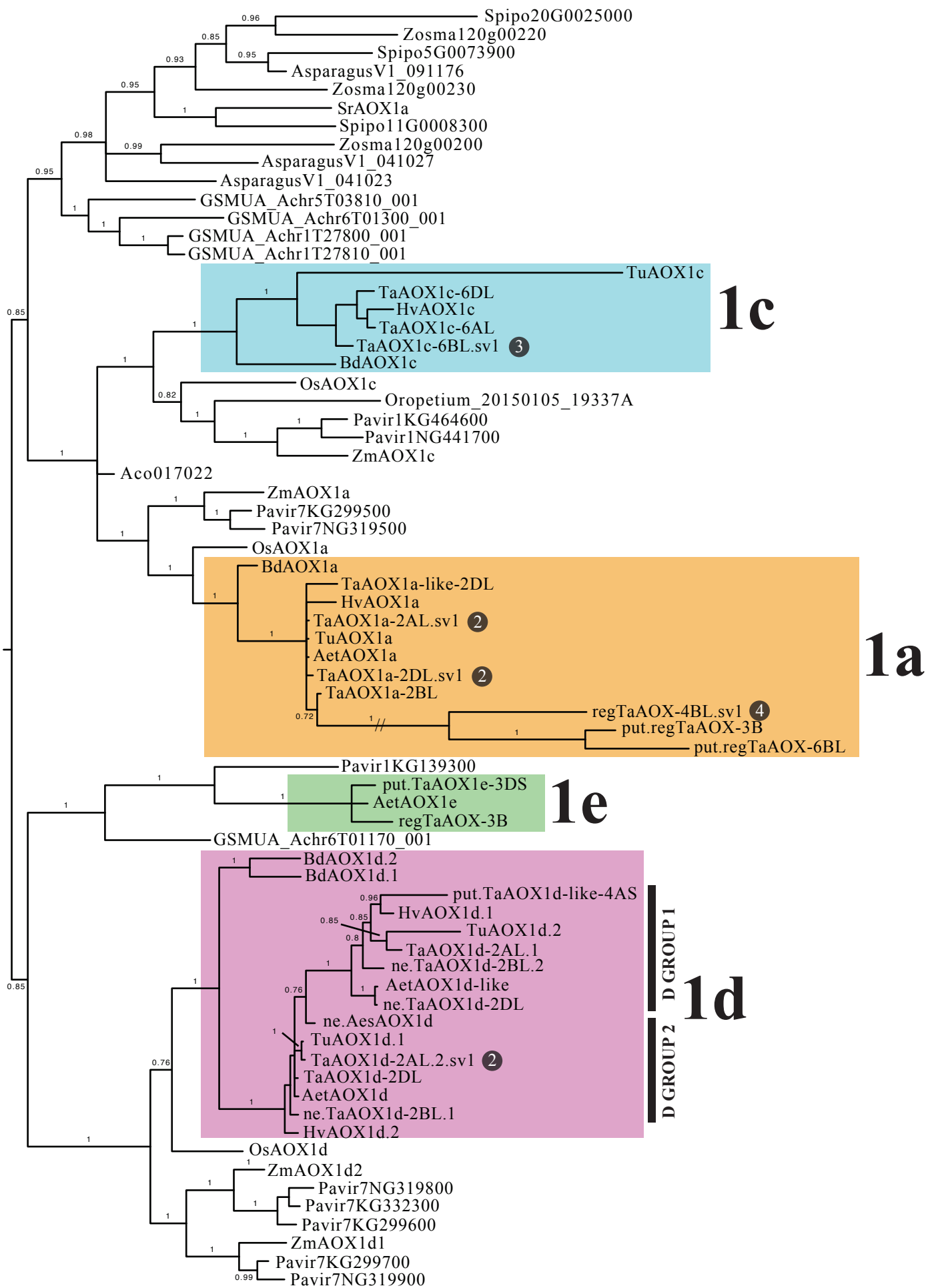

Supplement: S1 Fig — The number of splice variant isomers for a protein are denoted in the dark gray circle when applicable. Colored boxes distinguish the different AOX clades. (PDF) [file pone.0201439.s001.pdf]

**S8 Fig. Distribution of select *TaAOX* genes on the respective chromosomes.** Diagram not to scale.

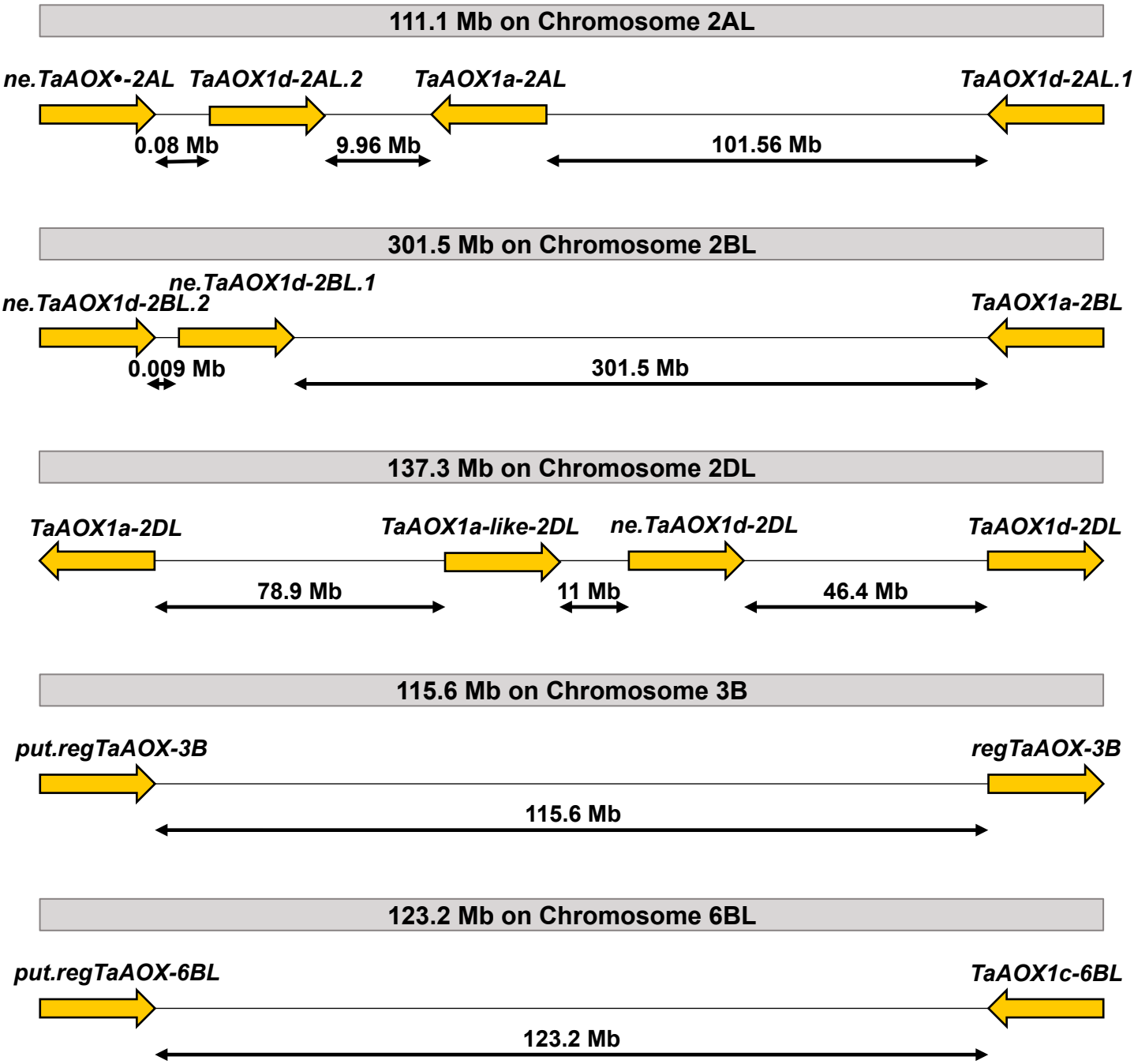

Supplement: S8 Fig — Diagram not to scale. (PDF) [file pone.0201439.s008.pdf]
