# Supplementary material for: Genome-wide identification and analysis of the ALTERNATIVE OXIDASE gene family in diploid and hexaploid wheat
Source: PLoS One. 2018 Aug 3;13(8):e0201439. doi: 10.1371/journal.pone.0201439 (PMC6075773; doi:10.1371/journal.pone.0201439)
Supplement: S2 Fig — (PDF) [file pone.0201439.s002.pdf]

S2 Fig. Select AOX protein sequences used in this study.

WHEAT AOX SEQUENCES:

TaAOX:

>TaAOX1a-2AL.sv1
MSSRMAGSVLLRRAGAGAGRLFATTASPAARTALGGGEGAWVRMMSTSAASQVKDE
AAKGVKAEAAKGDGEKKEVAISSYWGIEQSKKLVREDGTEWKWSCFRPWETYTADTS
IDLTKHHVPNTMLDKIAYYTVKSLRFPTDIFFQRRYGCRAMMLETVAAVPGMVGGMLL
HLRSLRRFEQSGGWIRALLEEAENERMHLMTFMEVAQPRWYERALVIAVQGVFFNAY
FFGYLISPKFAHRVVGYLEEEAVHSYTEFLKDLDDGKIDNVPAPAIAIDYWRLPANATLK
DVVTVVRADEAHHRDVNHFASDVYYQGMQLKATPAPIGYH

>TaAOX1a-2AL.sv2
MSSRMAGSVLLRRAGAGAGRLFATTASPAARTALGGGEGAWVRMMSTSAASQVKDE
AAKGDGEKKEVAISSYWGIEQSKKLVREDGTEWKWSCFRPWETYTADTSIDLTKHHV
PNTMLDKIAYYTVKSLRFPTDIFFQRRYGCRAMMLETVAAVPGMVGGMLLHLRSLRRF
EQSGGWIRALLEEAENERMHLMTFMEVAQPRWYERALVIAVQGVFFNAYFFGYLISPK
FAHRVVGYLEEEAVHSYTEFLKDLDDGKIDNVPAPAIAIDYWRLPANATLKDVVTVVRA
DEAHHRDVNHFASDVYYQGMQLKATPAPIGYH

>TaAOX1a-2BL
MPVVTGRFHAGGKKKEEEENRNDPLMRSIDLEEKKRPPQFLGSPGGRAGSPATDAAA
PPLPSPPPPSILLPNIKSIRGKTLPFANRNPPTPPPPTPTATNAGEGARRRHAHDPSRS
FGVFAEAFPGAQMSSRMAGSVLLRRAGAGASRLFATTPTSPAARTALAGGDGAWVR
MMSTSAASQVKDEAAKAVKAEAAKGDGEKKEVAISSYWGIEQSKKLVREDGTEWKW
SCFRPWETYTADTSIDLTKHHVPNTMLDKIAYYTVKSLRFPTDIFFQRRYGCRAMMLET
VAAVPGMVGGMLLHLRSLRRFEQSGGWIRALLEEAENERMHLMTFMEVAQPRWYER
ALVIAVQGVFFNAYFFGYLISPKFAHRVVGYLEEEAVHSYTEFLKDLDDGKIDNVPAPAI
AIDYWRLPANATLKDVVTVVRADEAHHRDVNHFASDVYYQGMQLKATPAPIGYH

>TaAOX1a-2DL.sv1
MSSRMAGSVLLRRAGAGASRLFATTPSPAARAVLGGGEGAWVRLMSTSAASQVKDE
AAKAVKAEAAKAVKAEAAKGDGEKKEVAISSYWGIEQSKKLVREDGTEWKWSCFRPW
ETYTADTSIDLTKHHVPNTMLDKIAYYTVKSLRFPTDIFFQRRYGCRAMMLETVAAVPG
MVGGMLLHLRSLRRFEQSGGWIRALLEEAENERMHLMTFMEVAQPRWYERALVIAVQ
GVFFNAYFFGYLISPKFAHRVVGYLEEEAVHSYTEFLKDLDDGKIDNVPAPAIAIDYWRL
PANATLKDVVTVVRADEAHHRDVNHFASDVYYQGMQLKATPAPIGYH

>TaAOX1a-2DL.sv2
MSSRMAGSVLLRRAGAGASRLFATTPSPAEAAKGDGEKKEVAISSYWGIEQSKKLVR
EDGTEWKWSCFRPWETYTADTSIDLTKHHVPNTMLDKIAYYTVKSLRFPTDIFFQRRY
GCRAMMLETVAAVPGMVGGMLLHLRSLRRFEQSGGWIRALLEEAENERMHLMTFME
VAQPRWYERALVIAVQGVFFNAYFFGYLISPKFAHRVVGYLEEEAVHSYTEFLKDLDD
GKIDNVPAPAIAIDYWRLPANATLKDVVTVVRADEAHHRDVNHFASDVYYQGMQLKAT
PAPIGYH

>TaAOX1a-like-2DL
MVGGVLLHLRSLRRFEHSGGWIRALMEEAENERMHLMTFMEVTQPLWYERALVIAVQ
GVFFNAYFFGYLISPKFAHRVVGYLEEEAVHSYTEFLKDLDDGKIDNVPASAIAIDYWRL
PANATLKAVVTVVRADEAHHRDVNHFASDVYYQGMQLKATPAPIGYH

>regTaAOX-4BL.sv1
MVRRRRWRSAATWGIEQSKKLVREEGTEWKWSCFRPWEAYSADMSIDLTKHHVPNT
MLDKIAYYTVKSPRFPTDIFFQVRMLPGVAPLLHSRVQASRGREQASSTPTR

>regTaAOX-4BL.sv2
MVRRRRWRSAATWGIEQSKKLVREEGTEWKWSCFRPWEAYSADMSIDLTKHHVPNT
MLDKIAYYTVKSPRFPTDIFFQVLFC

>regTaAOX-4BL.sv3
MVRRRRWRSAATWGIEQSKKLVREEGTEWKWSCFRLVSLRQPWEAYSADMSIDLTK
HHVPNTMLDKIAYYTVKSPRFPTDIFFQVLFC

>regTaAOX-4BL.sv4
MVRRRRWRSAATWGIEQSKKLVREEGTEWKWSCFRPWEAYSADMSIDLTKHHVPNT
MLDKIAYYTVKSPRFPTDIFFQVRMLPGVAPLLHSRVQASRGREQASSTPTR

>TaAOX1c-6AL
MPSWRALARRQRHVIPSPSQSLARPQVLEPATTSFASRAAAHQAGSSSSAMSSRVAG
SVLLRHLGPRVFGPTTPAAQRPLLAGGEGGAVAVAMWARPLSTSAAEAAREEATASK
DNVASTAAATAEAMQAAKADAVQAAKEGKSPAASSYWGIVPAKLVNKDGAEWKWSC
FRPWEAYTSDTTIDLSKHHKPKVLLDKIAYWTVKSLRVPTDIFFQRRYGCRAMMLETVA
AVPGMVGGMLLHLRSLRRFEQSGGWIRALLEEAENERMHLMTFMEVANPKWYERAL
VLAVQGVFFNAYFLGYIVSPKFAHRVVGYLEEEAIHSYTEFLRDLEAGRIENVPAPRIAID
YWRLPADARLKDVVTVVRADEAHHRDVNHFAADIHFQGLELNKTPAPLGYH

>TaAOX1c-6BL.sv1
MDERTQKLSTSQLRARKYSTSQAEPPFIPRLASSARSATPSRRALARRHVVKSPSQSL
ARPQVREPTTTSFASRAAAHQAGSSSSAMSSRVAGSVLLRHLGPRVFGPTTPAAQRP
LLAGGEGGAVVVWARPLSTSAAEAAREEAAASKDNVASTAAATAEAMQAAKAQAVQA
AKEGGKSPVSSYWGIVPAKLVNKDGAEWKWSCFRPWEAYTSDTTIDLTKHHKPKVLL
DKIAYWTVKSLRVPTDIFFQRRYGCRAMMLETVAAVPGMVGGMLLHLRSLRRFEQSG
GWIRALLEEAENERMHLMTFMEVAKPKWYERALVLAVQGVFFNAYFLGYIVSPKFAHR
VVGYLEEEAIHSYTEFLRDLEAGRIENVPAPRIAIDYWRLPADARLKDVVTVVRADEAH
HRDVNHFAADIHFQGLELNKTPAPLGYH

>TaAOX1c-6BL.sv2
MDERTQKLSTSQLRARKYSTSQAEPPFIPRLASSARSATPSRRALARRHVVKSPSQSL
ARPQVREPTTTSFASRAAAHQAGSSSSAMSSRVAGSVLLRHLGPRVFGPTTPAAQRP
LLAGGEGGAAAASKDNVASTAAATAEAMQAAKAQAVQAAKEGGKSPVSSYWGIVPAK
LVNKDGAEWKWSCFRPWEAYTSDTTIDLTKHHKPKVLLDKIAYWTVKSLRVPTDIFFQ
RRYGCRAMMLETVAAVPGMVGGMLLHLRSLRRFEQSGGWIRALLEEAENERMHLMT

FMEVAKPKWYERALVLAVQGVFFNAYFLGYIVSPKFAHRVVGYLEEEAIHSYTEFLRDL
EAGRIENVPAPRIAIDYWRLPADARLKDVVTVVRADEAHHRDVNHFAADIHFQGLELNK
TPAPLGYH

>TaAOX1c-6BL.sv3
MDERTQKLSTSQLRARKYSTSQAEPPFIPRLASSARSATPSRRALARRHVVKSPSQSL
ARPQVREPTTTSFASRAAAHQAGSSSSAMSSRVAGSVLLRHLGPRVFGPTTPAAQRP
LLAGGEGGAVVVWARPLSTSAAEAAREEAAASKDNVASTAAATAEAMQAAKAQAVQA
AKEGGKSPVSSYWGIVPAKLVNKDGAEWKWSCFRPWEAYTSDTTIDLTKHHKPKVLL
DKIAYWTVKSLRVPTDIFFQRRYGCRAMMLETVAAVPGMVGGMLLHLRSLRRFEQSG
GWIRALLEEAENERMHLMTFMEVAKPKWYERALVLAVQGVFFNAYFLGYIVSPKFAHR
VVGYLEEEAIHSYTEFLRDLEAGRIENVPAPRIAIDYWRLPADARLKDVVTVVRADEAH
HRDVNHFAADIHFQGLELNKTPAPLGYH

>TaAOX1c-6DL
MPSWRALARRHRHVIPSPSRSLARPQVLDPATTSFASRAAAHQAGSPSSAMSSRVAG
SVLLRHLGPRVFGPTTQAAQRTLLAGGEGGAVAMWAWPLSTSAAEAAREEAAASKD
NVASTAAATAEAMQAAKAEAVQAAKEGGKSPASSYWGIVPAKLVNKDGAEWKWSCF
RPWEAYTSDTTIDLTKHHKPKVLLDKIAYWTVKSLRVPTDIFFQRRYGCRAMMLETVAA
VPGMVGGMLLHLRSLRRFEQSGGWIRALLEEAENERMHLMTFMEVANPKWYERALV
LAVQGVFFNAYFLGYIVSPKFAHRVVGYLEEEAIHSYTEFLRDLEDGRIENVPAPRIAID
YWRLPPDARLKDVVTVVRADEAHHRDVNHFAADIHFQGLELNKTPAPLGYH

>regTaAOX-3B
MHLMTFMEVSQPRWYERALVVAVQGVFFHAYLATYLASPKVAHRMVGYLEEEAVHSY
TEFLRDLEAGKIDDVPAPTRRTTGTSNHYASDIHCQGHALREVAAPIGYH

>TaAOX1d-2AL.1
MPAAARIFPARMASTEAAAPHAKQEEATEKPQGATTPEHNKKAVVSYWGIEPRKLVKD
DGTEWTWFSFRPWDTYRPDTSIDMAKHHEPRAVADKVAYLIVRTLRAGSDLFFQRRH
ASHALLLEMVAAVPPMVGGVLLHLRSLRRFEHSSGWIRALMEEAENERMHLMTFMEV
TQPLWWERALVLATQGVFFNAYFVGYLVSPKFAHRFVGYLEEEAVHSYTKYLKDLEA
GLIENTPAPAIAIDYWRLPADARLKDVVTAVRADEAHHRDANHYASDIHYQGMTLNQTP
APLGYH

>TaAOX1d-2AL.2.sv1
MSSRMAGATLLRHLGPRLFAAAEPASGLAASARGIMPAAARIFPARMASTEAAAPHAK
QEDDAASPQAAATPEQQNKKPVVSYWGIEPRKLVKDDGTEWPWFCFRPWDTYRPDT
SIEVAKHHEPKALADKVAYFVVRSLRVPRDLFFQRRHASHALLLETVAAVPPMVGGVLL
HLRSLRRFEHSGGWIRALMEEAENERMHLMTFMEVTQPRWWERALVLAAQGVFFNA
YFVGYLISPKFAHRFVGYLEEEAVESYTEYLKDLEAGLIENTPAPAIAIDYWRLPADARL
KDVVTAVRADEAHHRDANHYASDVHYQGMTLNQSPAPLGYH

>TaAOX1d-2AL.2.sv2
MSSRMAGATLLRHLGPRLFAAAEPASGLAASARGIMPAAARIFPARMASTEAAAPHAK
QEDDAASPQAAATPEQQNKKPVVSYWGIEPRKLVKDDGTEWPWFCFRPWDTYRPDT

SIEVAKHHEPKALADKVAYFVVRSLRVPRDLFFQRRHASHALLLETVAAVPPMVGGVLL
HLRSLRRFEHSGGWIRALMEEAENERMHLMTFMEVTQPRWWERALVLAAQGVFFNA
YFVGYLISPKFAHRFVGYLEEEAVESYTEYLKDLEAGLIENTPAPAIAIDYWRLPADARL
KDVVTAVRADEAHHRDANHYASDVHYQGMTLNQSPAPLGYH

>TaAOX1d-2DL
MAGATLLRHLGPRLFAAAEPASGLAASARGIMPAAARIFPARMASTEAAAPHAKQEDD
AASPQAAATPEQQNKKPVVSYWGIEPRKLVKDDGTEWPWFCFRPWDTYRPDTSIDVT
KHHLPKALADKVAYFVVRSLRVPRDLFFQRRHASHALLLETVAAVPPMVGGVLLHLRS
LRRFEHSGGWIRALMEEAENERMHLMTFMEVTQPRWWERALVLAAQGVFFNAYFVG
YLISPKFAHRFVGYLEEEAVESYTEYLKDLEAGLIENTPAPAIAIDYWRLPADARLKDVV
TAVRADEAHHRDANHYASDIHYQGMTLNQTPAPLGYH

>put.regTaAOX-3B
MPWETYTADMSIDLTKHHVPNTMLDKIAYYTVKSLRFPTDIFFQDELQQDNLERKNFE
GKIKENQETITGYLILVAMLRFFGSPLFGPDQLTSVAG

>put.TaAOX1e-3DS
MAATLKKGEEEAASYWGVAPARLVKEDGTEWKWSCFRPWDAYEADVSIVLTKHHRP
ATFGDKVALWTVKAIRWPTDLFFQRRYGCRAMMLETVAAVPGMVARAVLHLRSLRRF
EQSGEWIRALLEEAQNERMHLMTFMEVSQPRWYERALVVVVQGVFFHAYLATYLASP
KVAHRMVGYLEEEAVHSYTEFLRDLEAGKIDDVPAPAIAIDYWRLPAGATLKDVVRVVR
ADEAHHRDVNHYASDIHCQGHALREVAAPIGYH

>put.TaAOX1d-like-4AS
MPTTARIFPARMASTAAGPHAKQEEATGKPQGATTPEQNKKAVPSYWGIKPRKLVED
DGTEWSWFSFRPWDTDLFFQRRHASHMLLLETVAAVPPMVGGVLLHLRSLRRFEHN
GGWIRALMEEAQNERMHLMTFMEVTQPLWCERALVLPTQGVFFNAYFIGYLVSPKFA
HRFVGYLEEEAVH

>put.regTaAOX-6BL
MEAFLGGQMSSRMAGSVLLRRAGAGASRLFSTTTMSPGARTFLAGGKGTWVRMMS
TSAASQVKDEAAKVVKAEAAKGDGNMVTLVQAALAILFEDELQQDNLERKNFEGKIKE
NQETITGYLILAGMLGSFDRPLFGSDQLTFVAG

>ne.TaAOX1d-2BL.1
MSSRMAGATLLRHLGPRLFAAAEPASGLAASARGIMPAAARIFPARMASTEAAAPHAK
QEDDAGTPQAAATPEQQSKKAVVSYWGIEPRKLVKEDGTEWPWFCFRPWDTYRPDT
SIDVTKHHEPKALADKVAYFVVRSLRVPRDLFFQRRHASHALLLETVAAVPPMVGGVLL
HLRSLRRFEHSGGWIRALMEEAENERMHLMTFMEVTQPRWWERALVLAAQGVFFNA
YFVGYLISPKFAHRFVGYLEEEAVESYTEYLKDLEAGLIENTPAPAIAIDYWRLPADARL
KDVVTAVRADEAHHRDANHYASDIHYQGMTLNQTPAPLGYH

>ne.TaAOX1d-2BL.2

MSSRMAGATLLRHLGPRLFAAAEPASGLAASARGIMPAAARIFPARMASTEAAGPRAK
QEEATEKPQGATTPEQNKKAVVSYWGIEPRKLVKDDGTEWPWFSFRPWDTYRPDTSI
DVAKHHEPRAVADKVAYLIVRTLRKGSDLFFQRRHASHALLLETVAAVPPMVGGVLLH
LRSLRRFEHSGGWIRALMEEAENERMHLMTFMEVTQPLWWERALVLATQGVFFNAY
FVGYLISPKFAHRFVGYLEEEAVHSYTEYLKDLEAGLIENTPAPAIAIDYWRLPADARLK
DVVIAVRADEAHHRDANHYASDIHYQGMTLNQTPAPLGYH

>ne.TaAOX1d-2DL
MSSRMAGATLLRHLGPHLFAAAEPASGLAASARGILPAAARIFPARMASTAAGAHAKQ
EGDAEKPESATAPEQNKKPVASYWGIEPRKLVKDDGTEWPWFSFRPWDTYRPDTSID
VAKHHEPRAVADKVAYLIVRTLRAGSDLFFQRRHASHALLLETVAAVPPMVGGVLLHL
RSLRRFEHSGGWIRALMEEAENERMHLMTFMEVTQPLWWERALVLATQGVFFNAYF
VGYLISPKFAHRFVGYLEEEAVHSYTEYLKDLEAGLIENTPAPAIAIDYWRLPADARLKD
VVIAVRADEAHHRDANHYASDIHYQGMTLNQTPAPLGYH

TuAOX:
>TuAOX1a
MLDKIAYYTVKSLRFPTDIFFQRRYGCRAMMLETVAAVPGMVGGMLLHLRSLRRFEQS
GGWIRALLEEAENERMHLMTFMEVAQPRWYERALVIAVQGVFFNAYFFGYLISPKFAH
RVVGYLEEEAVHSYTEFLKDLDDGKIDNVPAPAIAIDYWRLPANATLKDVVTVVRADEA
HHRDVNHFASDVYYQGMQLKATPAPIGYH

>TuAOX1c
MTQSFNNEAYMPTMGVGFNNSHWSQINDMHLDDHEFEVDEDGEGIVDAPKGRGGN
YTNEEDVLLCNTWLQVSRDPSVGDCQKWVAAQMAVDKLNSSGINDEDRRDGMDDLD
MSNKHMQTIDLDEEEEEASSDDGKRSPTPNSVSYSKPKRLDVCKKDAKEKKKRKRDD
ELKNAMKTIVKGRKEANEVRKMARNQDAAAEERKVTLEERKPWEAYTSDTTIDLSKHH
KPKVLLDKIAYWTVKSLRVPTDIFFQRRYGCRAMMLETVAAVPGMVGGMLLHLRSLRR
FEQSGGWIRALLEEAENERMHLMTFMEVANPKWYERALVLAVQGVFFNAYFLGYIVS
PKFAHRVVGYLEEEAIHSYTEFLRDLEAGRIENVPAPRIAIDYWRLPADARLKDVVTVVR
ADEAHHRDVNHFAADIHFQGLELNKTPAPLGYH

>TuAOX1d.1
MPAAARIFPARMASTEAAAPHAKQEDDAASPQAAATPEQQNKKPVVSYWGIEPRKLV
KDDGTEWPWFCFRPWDTYRPDTSIDVAKHHEPKALADKVAYFVVRSLRVPRDLFFQR
RHASHALLLETVAAVPPMVGGVLLHLRSLRRFEHSGGWIRALMEEAENERMHLMTFM
EVTQPRWWERALVLAAQGVFFNAYFVGYLISPKFAHRFVGYLEEEAVESYTEYLKDLE
AGLIENTPAPAIAIDYWRLPADARLKDVVTAVRADEAHHRDANHYASDVHYQGMTLNQ
SPAPLGYH

>TuAOX1d.2
MARKPVGGAAAAAPAPPRAAPGRGADPAERRRRWCGITVRGALVMLFPIAVSFLFSFI
FGIAGLLLGGLSSNASVSMPSTCRILSTANTMSSRMAGATLLRRAASARGIMPAAARVF
PARMASTEAAGPRAKQEEATEKPQGATAPEQNKKAVPSYWGIEPRKLVKDDGTEWP
WFSFRPWDTYRPDTSIDVAKHHEPRAVADKVAYLIVRTLRKGSDLFFQRRHASHALLL

ETVAAVPPMVGGVLLHLRSLRRFEHSGGWIRALMEEAENERMHLMTFMEVTQPLWW
ERALVLATQGVFFNAYFVGYLVSPKFAHRFVGYLEEEAVHSYTEYLKDLEAGLIENTPA
PAIAIDYWRLPADARLKDVVTAVRADEAHHRDANHYASDIHYQGMTLNQTPAPLGYH

AetAOX:
>AetAOX1a
MLDKIAYYTVKSLRFPTDIFFQRRYGCRAMMLETVAAVPGMVGGMLLHLRSLRRFEQS
GGWIRALLEEAENERMHLMTFMEVAQPRWYERALVIAVQGVFFNAYFFGYLISPKFAH
RVVGYLEEEAVHSYTEFLKDLDDGKIDNVPAPAIAIDYWRLPANATLKDVVTVVRADEA
HHRDVNHFASDVYYQGMQLKATPAPIGYH

>AetAOX1e
MAIFLPTKSWRCNSILSQIFIATQSTIRSHAAWHCDELVHEVTSHNLWSTEDASTCMQK
EKQRGSARNGHGAVGSAARREGGGARFFSVAGRSPAALGVGAARTAATLKQGEKEA
ASYWGVAPARLVKEDGTEWKWSCFRPWDAYEADVSIDLTKHHRPATLGDKVALWTV
KAMRWPTDLFFQRRYGCRAMMLETVAAVPGMVAGAVLHLRSLRRFEQSGGWIRALL
EEAENERMHLMTFMEVSQPRWYERALVVAVQGVFFHAYLATYLASPKVAHRMVGYL
EEEAVHSYTEFLRDLEAGKIDGVPAPAIAIDYWRLPAGATLKDVVRVVRADEAHHRDV
NHYASDIHCQGHALREVAAPIGYH

>AetAOX1d
MPAAARIFPARMASTEAAAPHAKQEDDAASPQAAATPEQQNKKPVVSYWGIEPRKLV
KDDGTEWPWFCFRPWDTYRPDTSIDVTKHHEPKALADKVAYFVVRSLRVPRDLFFQR
RHASHALLLETVAAVPPMVGGVLLHLRSLRRFEHSGGWIRALMEEAENERMHLMTFM
DVTQPRWWERALVLAAQGVFFNAYFVGYLISPKFAHRFVGYLEEEAVESYTEYLKDLE
AGLIENTPAPAIAIDYWRLPADARLKDVVTAVRADEAHHRDANHYASDIHYQGMTLNQT
PAPLGYH

>AetAOX1d-like
MSSRMAGATLLRHLGPHLFAAAEPASGLAASARGGAHAKQEGDAEKPESATAPEQNK
KPVASYWGIEPRKLVKDDGTEWPWFSFRPWDTYRPDTSIDVAKHHEPRAVADKPRRK
LVKDDGTEWPWFSFRPWDTYRPDTSIDVAKHHEPRAVADKVAYLIVRTLRAGSDLFFQ
RRHASHALLLETVAAVPPMVGGVLLHLRSLRRFEHSGGWIRALMEEAENERMHLMTF
MEGVFFNAYFVGYLISPKLKDVVIAVRADEAHHRDANHYASDIHYQGMTLNQTPAPLG
YH

AesAOX:
>ne.AesAOX1d
MSSRMAGATLLRHLGPRLFAAAEPASGLAASARGIMPAAARIFPARMASTEAAGPHAK
QESDAEKPESAATPEQQNKKPVVSYWGIEPRKLVKEDGTEWPWFCFRPWDTYRPDT
SIDVTKHHEPKALADKVAYFVVRSLRVPRDLFFQRRHASHALLLETVAAVPPMVGGVLL
HLRSLRRFEHSGGWIRALMEEAENERMHLMTFMEVTQPRWWERALVLAAQGVFFNA
YFVGYLISPKFAHRFVGYLEEEAVESYTEYLKDLEAGLIENTPAPAIAIDYWRLPADARL
KDVVTAVRADEAHHRDANHYASDIHYQGMTLNQTPAPLGYH

SELECTED AOX SEQUENCES:
>HvAOX1a
MSSRMAGSVLLRHAGAGAGRLFATTASPAARTALAGGEGAWARMMSTSAASHAKDE
AAKAAATGDGEKKEVAVNSYWGIEQSKKLVREDGTEWKWSCFRPWETYTADTSIDLT
KHHVPNTMLDKIAYYTVKSLRFPTDIFFQRRYGCRAMMLETVAAVPGMVGGMLLHLRS
LRRFEQSGGWIRALLEEAENERMHLMTFMEVAQPRWYERALVITVQGVFFNAYFFGY
LISPKFAHRVVGYLEEEAVHSYTEFLKDLDDGKIDNVPAPAIAIDYWRLPANATLKDVVT
VVRADEAHHRDVNHFASDVYYQGMELKATPAPIGYH

>HvAOX1c
EAVQAAKGGKSPAVSSYWGIVPAKLVNKDGAEWKWSCFRPWEAYTSDTTIDLTKHHK
PKVLLDKIAYWTVKSLRVPTDIFFQRRYGCRAMMLETVAAVPGMVGGMLLHLRSLRRF
EQSGGWIRALLEEAENERMHLMTFMEVANPKWYERALVLAVQGVFFNAYFVGYILSP
KFAHRVVGYLEEEAIHSYTEFLRDLEAGRIDNVPAPRIAIDYWRLPADARLKDVVTVVR
ADEAHHRDVNHFAADIHFQGLELNKTPAPLGYH

>HvAOX1d1
MSSRMAGATLLRHLGPRLFAAAEPASGLAASARGVMPAATRIFPARMASTAAAPHAK
QEEATEKPQGATTPEQAVSYWGIEPRKLVKDDGTEWPWFCFRPWDTYRPDTSIDVAK
HHEPRALPDKVAYLIVRTLRAGSDLFFQRRHASHALLLETVAAVPPMVGGVLLHLRSLR
RFEHSGGWIRALMEEAENERMHLMTFMEVTQPLWWERALVLATQGVFFNAYFVGYL
VSPKFAHRFVGYLEEEAVHSYTEYLKDLEAGLIENTPAPAIAIDYWRLPADARLKDVVTA
VRADEAHHRDANHYASDIHYQGMTLNQTPAPLGYH

>HvAOX1d2
MSSRMAGATLLRHLGPRLFVAAEPASGLAAGARGIMPAAARIFPARMASTEAAAPHAK
QEDDAKSPQAAATPAQQNKKAVVSYWGIEPRKLVKDDGTEWPWFCFRPWDTYRPDT
SIDVAKHHEPRALPDKVAYFVVRSLRVPRDLFFQRRHASHALLLETVAAVPPMVGGVL
LHLRSLRRFEHSGGWIRALMEEAENERMHLMTFMEVTQPRWWERALVLAAQGVFFN
AYFVGYLISPKFAHRFVGYLEEEAVESYTEYLKDLEAGLIENTPAPAIAIDYWRLPADAR
LKDVVTAVRADEAHHRDANHYASDIHYQGMTLNQTPAPLGYH

>ZmAOX1a
MMSSRAGSILLRHAGSRLFTAAAISPAAASRPLLAGGNGVPAVMLRLMSTSSPAAPTE
AKDEAAKASKVGGDKKAVVINSYWGIEQNNKLARDDGTEWKWTCFRPWETYTADTSI
DLTRHHEPKTLMDKVAYWTVKSLRFPTDIFFQRRYGCRAMMLETVAAVPGMVGGMLL
HLRSLRRFEQSGGWIRALLEEAENERMHLMTFMEVAKPRWYERALVITVQGVFFNAY
FLGYLLSPKFAHRVVGYLEEEAIHSYTEYLKDLEAGKIENVPAPAIAIDYWRLPANATLK
DVVTVVRADEAHHRDVNHFASDIHCQGMQLKQSPAPIGYH

>ZmAOX1c
MSTRTAGSALLRHLGPRVFGPIFSPAVAPPRPLLALAGGGEWGGALVWVRLLSTSAAE
AKEEVAASKGNSGSTAAAKAEAAEAAKEGDGKRDKVVSSYWGVAPSKLMNKDGAEW
RWSCFRRRYGCRAMMLETVAAVPGMVGGMLLHLRSLRRFEHSGGWIRALLEEAENE
RMHLMTFMEVAKPKWYERALVLAVQGVFFNAYFLGYLISPKFAHRVVGYLEEEAIHSY
TEYLKDLEAGKIENIPAPAIAIDYWQLPADATLKDVVVVVRSDEAHHRDVNHFAS

>ZmAOX1d1
MSSRMAGAALLRHLGPRLFAAGPAVSGLTARGGMPAAARLLPARMASTAAEAAREDA
GANKQHGGTEKHEEEAAGGQSKKAVVSYWGIDTPKLVKEDGTEWKWTSFRPWDAY
TSDTSIDIGKHHAPTTLPDKAAYLIVKSLRVPMDLFFQRRHASHALLLETVAAVPGMVG
GMLLHLRSLRRFEHSGGWIRALLEEAENERMHLMTFLEVTQPRWWERALVLTAQGVF
FNAYFVGYLLSPKFAHRVVGYLEEEAVHSYTEYLKDLEAGIIDNTPAPAIAIDYWRLPAD
AKLKDVVTVVRADEAHHRDVNHFASDIHYQGMKLKDTPAPLSYH

>ZmAOX1d2
MSSRMAGAALLRHLGPRLFAAEPVTGLAARGVMPAAARILPARMSSTAAEAAKEAAAP
QQRQKPEAAAAAPEGQDKKAVVSYWGIEPRKLVKEDGTEWRWFCFRPWDTYRADT
SIDMKKHHEPKALPDKLAYWLVKSLVVPKQLFFQRRHASHALLLETVAAVPGMVGGML
LHLRSLRRFEHSGGWIRALLEEAENERMHLMTFLEVAQPKWWERALVLAAQGVYFNA
YFVAYLASPKFAHRFVGYLEEEAVHSYTEYLKDLEAGIIDNTPAPAIAIDYWRLPADARL
KDVVAVVRADEAHHRDVNHFASDIHYQGMKLRDTPAPLGYH

>OsAOX1a
MSSRMAGSAILRHVGGVRLFTASATSPAAAAAAAARPFLAGGEAVPGVWGLRLMSTS
SVASTEAAAKAEAKKADAEKEVVVNSYWGIEQSKKLVREDGTEWKWSCFRPWETYT
ADTSIDLTKHHVPKTLLDKIAYWTVKSLRFPTDIFFQRRYGCRAMMLETVAAVPGMVG
GMLLHLRSLRRFEQSGGWIRTLLEEAENERMHLMTFMEVANPKWYERALVITVQGVF
FNAYFLGYLLSPKFAHRVVGYLEEEAIHSYTEFLKDLEAGKIDNVPAPAIAIDYWRLPAN
ATLKDVVTVVRADEAHHRDVNHFASDIHYQGMELKQTPAPIGYH

>OsAOX1c
MGSRAAGSVLLRHLCPRVSSSTSAAAHAHAQRPPLAGAGGGGVALWARLLSTSAAAA
KEETAASKENTGSTAAAKAEATKAAKEGPASATASPVASSYWGIEASKLASKDGVEW
KWSCFRPWETYSPDTTIDLKKHHEPKVLLDKVAYWTVKALRVPTDIFFQRRYGCRAM
MLETVAAVPGMVGGMLLHLRSLRRFEHSGGWIRALLEEAENERMHLMTFMEVAKPR
WYERALVLAVQGVFFNAYFLGYLLSPKLAHRVVGYLEEEAIHSYTEYLKDIEAGKIENVP
APPIAIDYWRLPAGATLKDVVVVVRADEAHHRDVNHFASDVHFQGMDLKDIPAPLDYH

>OsAOX1d
MSSRMAGATLLRHLGPRLFAAEPVYSGLAASARGVMPAAARIFPARMASTSSAGADV
KEGAAEKLPEPAATAAAAATDPQNKKAVVSYWGIQPPKLVKEDGTEWKWLSFRPWDT
YTSDTSIDVTKHHEPKGLPDKLAYWTVRSLAVPRDLFFQRRHASHALLLETVAGVPGM
VGGMLLHLRSLRRFEQSGGWIRALLEEAENERMHLMTFLEVMQPRWWERALVLAAQ
GVFFNAYFVGYLVSPKFAHRFVGYLEEEAVSSYTEYLKDLEAGKIENTPAPAIAIDYWR
LPADATLKDVVTVIRADEAHHRDLNHFASDIQQQGMKLKDTPAPIGYH
>BdAOX1a
MSSRMAGSVLLRHAGASRLFSATATSPAAAAARPFLAGGESVPGAWVRLMSTSAGS
QAKQEAAKAAAPPKDKEGGEKKEVVVNSYWGIEQAKKLVREDGTEWKWSCFRPWET
YTADTSIDLTKHHVPKTMLDKIAYWTVKSLRFPTDIFFQRRYGCRAMMLETVAAVPGM
VGGMLLHLRSLRRFEQSGGWIRALLEEAENERMHLMTFMEVAQPRWYERALVIAVQG

VFFNAYFFGYLISPKFAHRVVGYLEEEAVHSYTEFLKDLEAGKIDDVPAPSIAIDYWRLP
ANATLKDVVTVVRADEAHHRDVNHFASDVYYQGMELKATPAPIGYH

>BdAOX1c
MPSWQWWHVVARRHVVPLAPEKLARLQGRERAATSSPSRATHRREGSSSSAMSSR
VAGPAVLLRHLGRRIFSSPVSPASPVAAQRPLLSGGEGAVWARLRLLSTSAAEEAAKE
EAAASKENSASTAAAKAEAAQAAKDGDKTVVSSYWGIVPAKLVNKDGAEWKWSCFR
PWEAYTSDTTIDLKKHHEPKVLLDKIAYWTVKSLRVPTDIFFQRRYGCRAMMLETVAAV
PGMVGGMLLHLRSLRRFEHSGGWIRALLEEAENERMHLMTFMEVAGPKWYERALVL
AVQGVFFNAYFLGYLLSPKFAHRVVGYLEEEAVHSYTEFLRDIEAGKIDNVPAPRIAIDY
WRLPPDATLRDVVVVVRADEAHHRDVNHFASDIHFQGLELNKTPAPLGYH

>BdAOX1d.1
MSSRMAGATLLRHLAPRLVAAAEPASGLAARSIMPAAARIFPARMASTAAAPDVQEGA
AGATGKTEGQSKTKAVVSYWGIEPRKLVKADGTEWPWFCFRPWDTYTADTAIDMQK
HHEPKSLPDKIAYYTVKTLGVPKDLFFQRRHASHALLLETVAAVPPMVGGMLLHLRSLR
RFEHSGGWIRALMEEAENERMHLMTFLEVTQPKWWERALVMAVQGVFFNAYFVGYL
VSPKFAHRFVGYLEEEAVKSYTEYLKDLEAGKIENTPAPAIAIDYWRLPADATLKDVVAV
VRADEAHHRDANHYASDIHYQGLTLKETPAPIGYH

>BdAOX1d.2
MSSRMAGATLLRHLGPRLFAAAEPASGLAARSIMAPAAARILPARMASTASAAPDAKE
GASAAAKTDSAATPEQSKTKSVVSYWGIESRKLVKPDGTEWPWFCFTPWDTYRADTS
IDMEKHHKPKSVPDKVAYYAVRSLRVPMDLFFQRRHASHALLLETVAAVPPMVGGMLL
HLRSLRRFEHSGGWIRALMEEAENERMHLMTFLEVTQPNWWERALVMAAQGVFVNA
YFVGYLVSPKFAHRFVGYLEEEAVHSYTEYLKDLEAGKIENTPAPAIAIDYWRLPADATL
KDVVTVIRADEAHHRDANHYASDIHYQGLTLKETPAPIGYH

>TbAOX
MFRNHASRITAAAAPWVLRTACRQKSDAKTPVWGHTQLNRLSFLETVPVVPLRVSDE
SSEDRPTWSLPDIENVAITHKKPNGLVDTLAYRSVRTCRWLFDTFSLYRFGSITESKVIS
RCLFLETVAGVPGMVGGMLRHLSSLRYMTRDKGWINTLLVEAENERMHLMTFIELRQ
PGLPLRVSIIITQAIMYLFLLVAYVISPRFVHRFVGYLEEEAVITYTGVMRAIDEGRLRPTK
NDVPEVARVYWNLSKNATFRDLINVIRADEAEHRVVNHTFADMHEKRLQNSVNPFVVL
KKNPEEMYSNQPSGKTRTDFGSEGAKTASNVNKHV

>SgAOX
MMSSRLVGTALCRQLSHVPVPQYLPALRPTADTASSLLHGCSAAAPAQRAGLWPPS
WFSPPRHASTLSAPAQDGGKEKAAGTAGKVPPGEDGGAEKEAVVSYWAVPPSKVSK
EDGSEWRWTCFRPWETYQADLSIDLHKHHVPTTILDKLALRTVKALRWPTDIFFQRRY
ACRAMMLETVAAVPGMVGGVLLHLKSLRRFEHSGGWIRALLEEAENERMHLMTFMEV
AQPRWYERALVLAVQGVFFNAYFLGYLLSPKFAHRVVGYLEEEAIHSYTEFLKDIDSGA
IQDCPAPAIALDYWRLPQGSTLRDVVTVVRADEAHHRDVNHFASDVHYQDLELKTTPA
PLGYH

>AtAOX1a

MMITRGGAKAAKSLLVAAGPRLFSTVRTVSSHEALSASHILKPGVTSAWIWTRAPTIGG
MRFASTITLGEKTPMKEEDANQKKTENESTGGDAAGGNNKGDKGIASYWGVEPNKIT
KEDGSEWKWNCFRPWETYKADITIDLKKHHVPTTFLDRIAYWTVKSLRWPTDLFFQRR
YGCRAMMLETVAAVPGMVGGMLLHCKSLRRFEQSGGWIKALLEEAENERMHLMTFM
EVAKPKWYERALVITVQGVFFNAYFLGYLISPKFAHRMVGYLEEEAIHSYTEFLKELDK
GNIENVPAPAIAIDYWRLPADATLRDVVMVVRADEAHHRDVNHFASDIHYQGRELKEA
PAPIGYH
