# Supplementary material for: Genome-wide identification and analysis of the ALTERNATIVE OXIDASE gene family in diploid and hexaploid wheat
Source: PLoS One. 2018 Aug 3;13(8):e0201439. doi: 10.1371/journal.pone.0201439 (PMC6075773; doi:10.1371/journal.pone.0201439)
Supplement: S3 Fig — Blue indicates presence of Type 1 residues. Red indicates a Type 2 residue. Green indicates residues for monocot Type 1(d). Yellow indicates Type 1(a-c/e). Purple represents amino acid residues that did not match either classification. Black represents residues that were absent. (PDF) [file pone.0201439.s003.pdf]

**S3 Fig. Alignment of wheat AOX sequences with *A. thaliana* AOX1a in order to determine classification.** Blue indicates presence of Type 1 residues. Red indicates a Type 2 residue. Green indicates residues for monocot Type 1(d). Yellow indicates Type 1(a-c/e). Purple represents amino acid residues that did not match either classification. Black represents residues that were absent.

TaAOX1a-2AL.sv1:

```
AtAOX1a             --------MMITRGGAKAAKSLLVAAGPRLFSTVRTVSSHEALSASHILKPGVTSAWIWT      52
TaAA0283900.1       MSSRMAGSVLLRRAGAGAG---------RLFATTASPAAR-------TALGGGEGAWVRM      44
                            ::: *.** *.         ***:*. : :::           *  .**:

AtAOX1a             RAPTIGGMRFASTITLGEKTPMKEEDANQKKTENESTGGDAAGGNNKGDKGIASYWGVEP      112
TaAA0283900.1       MS-------------TSAASQVKDEAAK------GVKAEAAKGDGEKKEVAISSYWGIEQ      85
                     :              .  : :*:* *:        ..  * *..:* : .*:****:*

AtAOX1a             N-KITKEDGSEWKWNCFRPWETYKADITIDLKKHHVPTTFLDRIAYWTVKSLRWPTDLFF      171
TaAA0283900.1       SKKLVREDGTEWKWSCFRPWETYTADTSIDLTKHHVPNTMLDKIAYYTVKSLRFPTDIFF      145
                    . *:.:***:****.********.** :***.*****.*:**:***:******:***:**

AtAOX1a             QRRYGCRAMMLETVAAVPGMVGGMLLHCKSLRRFEQSGGWIKALLEEAENERMHLMTFME      231
TaAA0283900.1       QRRYGCRAMMLETVAAVPGMVGGMLLHLRSLRRFEQSGGWIRALLEEAENERMHLMTFME      205
                    *************************** :************:******************

AtAOX1a             VAKPKWYERALVITVQGVFFNAYFLGYLISPKFAHRMVGYLEEEAIHSYTEFLKELDKGN      291
TaAA0283900.1       VAQPRWYERALVIAVQGVFFNAYFFGYLISPKFAHRVVGYLEEEAVHSYTEFLKDLDDGK      265
                    **:*:********:**********:***********:********:********:**.*:

AtAOX1a             IENVPAPAIAIDYWRLPADATLRDVVMVVRADEAHHRDVNHFASDIHYQGRELKEAPAPI      351
TaAA0283900.1       IDNVPAPAIAIDYWRLPANATLKDVVTVVRADEAHHRDVNHFASDVYYQGMQLKATPAPI      325
                    *:****************:***:*** ******************::*** :** :****

AtAOX1a             GYH-   354
TaAA0283900.1       GYH*   328
                    ***
```

TaAOX1a-2AL.sv2:

```
AtAOX1a             --------MMITRGGAKAAKSLLVAAGPRLFSTVRTVSSHEALSASHILKPGVTSAWIWT      52
TaAA0283900.2       MSSRMAGSVLLRRAGAGAG---------RLFATTASPAAR-------TALGGGEGAWVRM      44
                            ::: *.** *.         ***:*. : :::           *  .**:

AtAOX1a             RAPTIGGMRFASTITLGEKTPMKEEDANQKKTENESTGGDAAGGNNKGDKGIASYWGVEP      112
TaAA0283900.2       M---------------------STSAAS------QVKDEAAKGDGEKKEVAISSYWGIEQ      77
                                          . . *.      : ..  * *..:* : .*:****:*

AtAOX1a             N-KITKEDGSEWKWNCFRPWETYKADITIDLKKHHVPTTFLDRIAYWTVKSLRWPTDLFF      171
TaAA0283900.2       SKKLVREDGTEWKWSCFRPWETYTADTSIDLTKHHVPNTMLDKIAYYTVKSLRFPTDIFF      137
                    . *:.:***:****.********.** :***.*****.*:**:***:******:***:**

AtAOX1a             QRRYGCRAMMLETVAAVPGMVGGMLLHCKSLRRFEQSGGWIKALLEEAENERMHLMTFME      231
TaAA0283900.2       QRRYGCRAMMLETVAAVPGMVGGMLLHLRSLRRFEQSGGWIRALLEEAENERMHLMTFME      197
                    *************************** :************:******************

AtAOX1a             VAKPKWYERALVITVQGVFFNAYFLGYLISPKFAHRMVGYLEEEAIHSYTEFLKELDKGN      291
TaAA0283900.2       VAQPRWYERALVIAVQGVFFNAYFFGYLISPKFAHRVVGYLEEEAVHSYTEFLKDLDDGK      257
                    **:*:********:**********:***********:********:********:**.*:

AtAOX1a             IENVPAPAIAIDYWRLPADATLRDVVMVVRADEAHHRDVNHFASDIHYQGRELKEAPAPI      351
TaAA0283900.2       IDNVPAPAIAIDYWRLPANATLKDVVTVVRADEAHHRDVNHFASDVYYQGMQLKATPAPI      317
                    *:****************:***:*** ******************::*** :** :****

AtAOX1a             GYH-   354
TaAA0283900.2       GYH*   320
                    ***
```

TaAOX1a-2BL:

```
AtAOX1a          ------------------------------------------------------------      0
TaAA0439680.1    MPVVTGRFHAGGKKKEEEENRNDPLMRSIDLEEKKRPPQFLGSPGGRAGSPATDAAAPPL      60


AtAOX1a          ------------------------------------------------------------      0
TaAA0439680.1    PSPPPPSILLPNIKSIRGKTLPFANRNPPTPPPPTPTATNAGEGARRRHAHDPSRSFGVF      120


AtAOX1a          ----------------MMITRGGAKAAKSLLVAAGPRLFSTVRTVSSHEALSASHILKPG      44
TaAA0439680.1    AEAFPGAQMSSRMAGSVLLRRAGAG---------ASRLFATTPTSP------AARTALAG      165
                                 ::: *.**          . ***:*. *        *::    *

AtAOX1a          VTSAWIWTRAPTIGGMRFASTITLGEKTPMKEEDANQKKTENESTGGDAAGGNNKGDKGI      104
TaAA0439680.1    GDGAWVRMMS-------------TSAASQVKDEAAK------AVKAEAAKGDGEKKEVAI      206
                   .**:   :             .  : :*:* *:         ..  * *..:* : .*

AtAOX1a          ASYWGVEPN-KITKEDGSEWKWNCFRPWETYKADITIDLKKHHVPTTFLDRIAYWTVKSL      163
TaAA0439680.1    SSYWGIEQSKKLVREDGTEWKWSCFRPWETYTADTSIDLTKHHVPNTMLDKIAYYTVKSL      266
                 :****:* . *:.:***:****.********.** :***.*****.*:**:***:*****

AtAOX1a          RWPTDLFFQRRYGCRAMMLETVAAVPGMVGGMLLHCKSLRRFEQSGGWIKALLEEAENER      223
TaAA0439680.1    RFPTDIFFQRRYGCRAMMLETVAAVPGMVGGMLLHLRSLRRFEQSGGWIRALLEEAENER      326
                 *:***:***************************** :************:**********

AtAOX1a          MHLMTFMEVAKPKWYERALVITVQGVFFNAYFLGYLISPKFAHRMVGYLEEEAIHSYTEF      283
TaAA0439680.1    MHLMTFMEVAQPRWYERALVIAVQGVFFNAYFFGYLISPKFAHRVVGYLEEEAVHSYTEF      386
                 **********:*:********:**********:***********:********:******

AtAOX1a          LKELDKGNIENVPAPAIAIDYWRLPADATLRDVVMVVRADEAHHRDVNHFASDIHYQGRE      343
TaAA0439680.1    LKDLDDGKIDNVPAPAIAIDYWRLPANATLKDVVTVVRADEAHHRDVNHFASDVYYQGMQ      446
                 **:**.*:*:***************:***:*** *******************::*** :

AtAOX1a          LKEAPAPIGYH-   354
TaAA0439680.1    LKATPAPIGYH*   457
                 ** :*******
```

TaAOX1a-2DL.sv1:

```
AtAOX1a          --------MMITRGGAKAAKSLLVAAGPRLFSTVRTVSSHEALSASHILKPGVTSAWIWT      52
TaAA0531270.1    MSSRMAGSVLLRRAGAGA---------SRLFATTPTSP-------AARAVLGGGEGAWVRL      44
                         ::: *.** *           ***:*. :        *:: :  *  .**:

AtAOX1a          RAPTIGGMRFASTITLGEKTPMKEEDANQKKT--ENESTGGDAAGGNNKGDKGIASYWGV      110
TaAA0531270.1    MST-------------SAASQVKDEAAKAVKAEAAKAVKAEAAKGDGEKKEVAISSYWGI      91
                  :               .  : :*:* *:   *:    :  ..  * *..:* : .*:****:

AtAOX1a          EPN-KITKEDGSEWKWNCFRPWETYKADITIDLKKHHVPTTFLDRIAYWTVKSLRWPTDL      169
TaAA0531270.1    EQSKKLVREDGTEWKWSCFRPWETYTADTSIDLTKHHVPNTMLDKIAYYTVKSLRFPTDI      151
                 * . *:.:***:****.********.** :***.*****.*:**:***:******:***:

AtAOX1a          FFQRRYGCRAMMLETVAAVPGMVGGMLLHCKSLRRFEQSGGWIKALLEEAENERMHLMTF      229
TaAA0531270.1    FFQRRYGCRAMMLETVAAVPGMVGGMLLHLRSLRRFEQSGGWIRALLEEAENERMHLMTF      211
                 ***************************** :************:****************

AtAOX1a          MEVAKPKWYERALVITVQGVFFNAYFLGYLISPKFAHRMVGYLEEEAIHSYTEFLKELDK      289
TaAA0531270.1    MEVAQPRWYERALVIAVQGVFFNAYFFGYLISPKFAHRVVGYLEEEAVHSYTEFLKDLDD      271
                 ****:*:********:**********:***********:********:********:**.

AtAOX1a          GNIENVPAPAIAIDYWRLPADATLRDVVMVVRADEAHHRDVNHFASDIHYQGRELKEAPA      349
TaAA0531270.1    GKIDNVPAPAIAIDYWRLPANATLKDVVTVVRADEAHHRDVNHFASDVYYQGMQLKATPA      331
                 *:*:***************:***:*** *****************::*** :** :**

AtAOX1a          PIGYH- 354
TaAA0531270.1    PIGYH* 336
                 *****
```

TaAOX1a-2DL.sv2:

```
AtAOX1a            MMITRGGAKAAKSLLVAAGPRLFSTVRTVSSHEALSASHILKPGVTSAWIWTRAPTIGGM      60
TaAA0531270.2      ---------------------------------------------------------MSS      3
                                                                             .

AtAOX1a            RFASTITLGEKTPMKEEDANQKKTENESTGGDAAGGNNKGDKGIASYWGVEPN-KITKED      119
TaAA0531270.2      RMAGSVLLRRA----GAGASRLFATTPSPAEAAKGDGEKKEVAISSYWGIEQSKKLVRED      59
                   *:*.:: * .       .*.:  : . * .  * *..:* : .*:****:* . *:.:**

AtAOX1a            GSEWKWNCFRPWETYKADITIDLKKHHVPTTFLDRIAYWTVKSLRWPTDLFFQRRYGCRA      179
TaAA0531270.2      GTEWKWSCFRPWETYTADTSIDLTKHHVPNTMLDKIAYYTVKSLRFPTDIFFQRRYGCRA      119
                   *:****.********.** :***.*****.*:**:***:******:***:**********

AtAOX1a            MMLETVAAVPGMVGGMLLHCKSLRRFEQSGGWIKALLEEAENERMHLMTFMEVAKPKWYE      239
TaAA0531270.2      MMLETVAAVPGMVGGMLLHLRSLRRFEQSGGWIRALLEEAENERMHLMTFMEVAQPRWYE      179
                   ******************* :************:********************:*:***

AtAOX1a            RALVITVQGVFFNAYFLGYLISPKFAHRMVGYLEEEAIHSYTEFLKELDKGNIENVPAPA      299
TaAA0531270.2      RALVIAVQGVFFNAYFFGYLISPKFAHRVVGYLEEEAVHSYTEFLKDLDDGKIDNVPAPA      239
                   *****:**********:***********:********:********:**.*:*:******

AtAOX1a            IAIDYWRLPADATLRDVVMVVRADEAHHRDVNHFASDIHYQGRELKEAPAPIGYH-  354
TaAA0531270.2      IAIDYWRLPANATLKDVVTVVRADEAHHRDVNHFASDVYYQGMQLKATPAPIGYH*  294
                   **********:***:*** ******************::*** :** :*******
```

TaAOX1a-like-2DL:

```
AtAOX1a            MMITRGGAKAAKSLLVAAGPRLFSTVRTVSSHEALSASHILKPGVTSAWIWTRAPTIGGM      60
TaAA0549780.1      ------------------------------------------------------------      0

AtAOX1a            RFASTITLGEKTPMKEEDANQKKTENESTGGDAAGGNNKGDKGIASYWGVEPNKITKEDG      120
TaAA0549780.1      ------------------------------------------------------------      0

AtAOX1a            SEWKWNCFRPWETYKADITIDLKKHHVPTTFLDRIAYWTVKSLRWPTDLFFQRRYGCRAM      180
TaAA0549780.1      ------------------------------------------------------------      0

AtAOX1a            MLETVAAVPGMVGGMLLHCKSLRRFEQSGGWIKALLEEAENERMHLMTFMEVAKPKWYER      240
TaAA0549780.1      ----------MVGGVLLHLRSLRRFEHSGGWIRALMEEAENERMHLMTFMEVTQPLWYER      50
                             ****:*** :******:*****:**:****************::* ****

AtAOX1a            ALVITVQGVFFNAYFLGYLISPKFAHRMVGYLEEEAIHSYTEFLKELDKGNIENVPAPAI      300
TaAA0549780.1      ALVIAVQGVFFNAYFFGYLISPKFAHRVVGYLEEEAVHSYTEFLKDLDDGKIDNVPASAI      110
                   ****:**********:***********:********:********:**.*:*:**** **

AtAOX1a            AIDYWRLPADATLRDVVMVVRADEAHHRDVNHFASDIHYQGRELKEAPAPIGYH-   354
TaAA0549780.1      AIDYWRLPANATLKAVVTVVRADEAHHRDVNHFASDVYYQGMQLKATPAPIGYH*   164
                   *********:***: ** ******************::*** :** :*******
```

regTaAOX-4BL.sv1:

```
AtAOX1a             MMITRGGAKAAKSLLVAAGPRLFSTVRTVSSHEALSASHILKPGVTSAWIWTRAPTIGGM      60
TaAA1061160.1       ------------------------------------------------------------      0


AtAOX1a             RFASTITLGEKTPMKEEDANQKKTENESTGGDAAGGNNKGDKGIASYWGVEP-NKITKED      119
TaAA1061160.1       -----------------------------------MVRRRRWRSAATWGIEQSKKLVREE      25
                                                         .:     *: **:*  :*:.:*:

AtAOX1a             GSEWKWNCFRPWETYKADITIDLKKHHVPTTFLDRIAYWTVKSLRWPTDLFFQRRYGCRA      179
TaAA1061160.1       GTEWKWSCFRPWEAYSADMSIDLTKHHVPNTMLDKIAYYTVKSPRFPTDIFFQVRMLPGV      85
                    *:****.******:*.**::***.*****.*:**:***:**** *:***:*** *    .

AtAOX1a             --MMLETVAAVPGMVGGMLLHCKSLRRFEQSGGWIKALLEEAENERMHLMTFMEVAKPKW      237
TaAA1061160.1       APLLHSRVQASRGREQAS---STPTR*---------------------------------      108
                      :: . * *  *   .    ..  *

AtAOX1a             YERALVITVQGVFFNAYFLGYLISPKFAHRMVGYLEEEAIHSYTEFLKELDKGNIENVPA      297
TaAA1061160.1       ------------------------------------------------------------      108


AtAOX1a             PAIAIDYWRLPADATLRDVVMVVRADEAHHRDVNHFASDIHYQGRELKEAPAPIGYH 354
TaAA1061160.1       --------------------------------------------------------- 108
```

regTaAOX-4BL.sv2:

```
AtAOX1a             MMITRGGAKAAKSLLVAAGPRLFSTVRTVSSHEALSASHILKPGVTSAWIWTRAPTIGGM      60
TaAA1061160.2       ------------------------------------------------------------      0


AtAOX1a             RFASTITLGEKTPMKEEDANQKKTENESTGGDAAGGNNKGDKGIASYWGVEP-NKITKED      119
TaAA1061160.2       -----------------------------------MVRRRRWRSAATWGIEQSKKLVREE      25
                                                         .:     *: **:*  :*:.:*:

AtAOX1a             GSEWKWNCFRPWETYKADITIDLKKHHVPTTFLDRIAYWTVKSLRWPTDLFFQRRYGCRA      179
TaAA1061160.2       GTEWKWSCFRPWEAYSADMSIDLTKHHVPNTMLDKIAYYTVKSPRFPTDIFFQVLFC*--      82
                    *:****.******:*.**::***.*****.*:**:***:**** *:***:***  :

AtAOX1a             MMLETVAAVPGMVGGMLLHCKSLRRFEQSGGWIKALLEEAENERMHLMTFMEVAKPKWYE      239
TaAA1061160.2       ------------------------------------------------------------      82


AtAOX1a             RALVITVQGVFFNAYFLGYLISPKFAHRMVGYLEEEAIHSYTEFLKELDKGNIENVPAPA      299
TaAA1061160.2       ------------------------------------------------------------      82


AtAOX1a             IAIDYWRLPADATLRDVVMVVRADEAHHRDVNHFASDIHYQGRELKEAPAPIGYH   354
TaAA1061160.2       -------------------------------------------------------   82
```

regTaAOX-4BL.sv3:

```
AtAOX1a          MMITRGGAKAAKSLLVAAGPRLFSTVRTVSSHEALSASHILKPGVTSAWIWTRAPTIGGM    60
TaAA1061160.3    ------------------------------------------------------------    0

AtAOX1a          RFASTITLGEKTPMKEEDANQKKTENESTGGDAAGGNNKGDKGIASYWGVEP-NKITKED    119
TaAA1061160.3    -----------------------------------MVRRRRWRSAATWGIEQSKKLVREE    25
                                                      .:     *: **:*  :*:.:*:

AtAOX1a          GSEWKWNCFR------PWETYKADITIDLKKHHVPTTFLDRIAYWTVKSLRWPTDLFFQR    173
TaAA1061160.3    GTEWKWSCFRLVSLRQPWEAYSADMSIDLTKHHVPNTMLDKIAYYTVKSPRFPTDIFFQV    85
                 *:****.***      ***:*.**::***.*****.*:**:***:**** *:***:***

AtAOX1a          RYGCRAMMLETVAAVPGMVGGMLLHCKSLRRFEQSGGWIKALLEEAENERMHLMTFMEVA    233
TaAA1061160.3    LFC*--------------------------------------------------------    88
                  :

AtAOX1a          KPKWYERALVITVQGVFFNAYFLGYLISPKFAHRMVGYLEEEAIHSYTEFLKELDKGNIE    293
TaAA1061160.3    ------------------------------------------------------------    88

AtAOX1a          NVPAPAIAIDYWRLPADATLRDVVMVVRADEAHHRDVNHFASDIHYQGRELKEAPAPIGY    353
TaAA1061160.3    ------------------------------------------------------------    88

AtAOX1a          H      354
TaAA1061160.3    -      88
```

regTaAOX-4BL.sv4:

```
AtAOX1a          MMITRGGAKAAKSLLVAAGPRLFSTVRTVSSHEALSASHILKPGVTSAWIWTRAPTIGGM    60
TaAA1061160.4    ------------------------------------------------------------    0

AtAOX1a          RFASTITLGEKTPMKEEDANQKKTENESTGGDAAGGNNKGDKGIASYWGVEP-NKITKED    119
TaAA1061160.4    -----------------------------------MVRRRRWRSAATWGIEQSKKLVREE    25
                                                      .:     *: **:*  :*:.:*:

AtAOX1a          GSEWKWNCFRPWETYKADITIDLKKHHVPTTFLDRIAYWTVKSLRWPTDLFFQRRYGCRA    179
TaAA1061160.4    GTEWKWSCFRPWEAYSADMSIDLTKHHVPNTMLDKIAYYTVKSPRFPTDIFFQVRMLPGV    85
                 *:****.******:*.**::***.*****.*:**:***:**** *:***:*** *    .

AtAOX1a          --MMLETVAAVPGMVGGMLLHCKSLRRFEQSGGWIKALLEEAENERMHLMTFMEVAKPKW    237
TaAA1061160.4    APLLHSRVQASRGREQAS---STPTR*---------------------------------    108
                   :: . * *  *   .    ..  *

AtAOX1a          YERALVITVQGVFFNAYFLGYLISPKFAHRMVGYLEEEAIHSYTEFLKELDKGNIENVPA    297
TaAA1061160.4    ------------------------------------------------------------    108

AtAOX1a          PAIAIDYWRLPADATLRDVVMVVRADEAHHRDVNHFASDIHYQGRELKEAPAPIGYH 354
TaAA1061160.4    --------------------------------------------------------- 108
```

put.regTaAOX-3B:

```
AtAOX1a             MMITRGGAKAAKSLLVAAGPRLFSTVRTVSSHEALSASHILKPGVTSAWIWTRAPTIGGM 60
PUT.REGTAAOX-3B     ------------------------------------------------------------ 0

AtAOX1a             RFASTITLGEKTPMKEEDANQKKTENESTGGDAAGGNNKGDKGIASYWGVEPNKITKEDG 120
PUT.REGTAAOX-3B     ------------------------------------------------------------ 0

AtAOX1a             SEWKWNCFRPWETYKADITIDLKKHHVPTTFLDRIAYWTVKSLRWPTDLFFQRRYGCRAM180
PUT.REGTAAOX-3B     --------MPWETYTADMSIDLTKHHVPNTMLDKIAYYTVKSLRFPTDIFFQDELQQLNL 52
                             *****.**::***.*****.*:**:***:******:***:*** .     :

AtAOX1a             MLETVA----AVPGMVGGMLLHCKSLRRFEQSGGWIKALLEEAENERMHLMTFMEVAKPK236
PUT.REGTAAOX-3B     ERKNFEGKIKENQETITGYLILVAMLRFFGSP---------------------------- 84
                      :..           : * *:    ** * .

AtAOX1a             WYERALVITVQGVFFNAYFLGYLISPKFAHRMVGYLEEEAIHSYTEFLKELDKGNIENVP 296
PUT.REGTAAOX-3B     LFGPDQLTSVAG------------------------------------------------ 96
                     :    : :* *

AtAOX1a             APAIAIDYWRLPADATLRDVVMVVRADEAHHRDVNHFASDIHYQGRELKEAPAPIGYH   354
PUT.REGTAAOX-3B     ----------------------------------------------------------   96
```

put.regTaAOX-6BL:

```
AtAOX1a             ----------------MMITRGGAKAAKSLLVAAGPRLFSTVRTVSSHEALSASHILKPG 44
put.regTaAOX-6BL    MEAFLGGQMSSRMAGSVLLRRAGAGA---------SRLFSTTTMSP------GARTFLAG 45
                                    ::: *.** *          *****.          .:: :  *

AtAOX1a             VTSAWIWTRAPTIGGMRFASTITLGEKTPMKEEDANQKKTENESTGGDAAGGNNKGDKGI 104
put.regTaAOX-6BL    GKGTWVRMMSTS-------------AASQVKDEAAKVVKAE--A---------AKGDGNM 81
                     ..:*:   : :                : :*:* *:  *:*  :          *** .:

AtAOX1a             ASYWGVEPNKITKEDGSEWKWNCFRPWETYKADITIDLKKHHVPTTFLDRIAYWTVKSLR 164
put.regTaAOX-6BL    VTLVQA-----------------------------------ALAILFEDELQ-------- 98
                    .:   .                                    :   * *.:

AtAOX1a             WPTDLFFQRRYGCRAMMLETVAAVPGMVGGMLLHCKSLRRFEQSGGWIKALLEEAENERM 224
put.regTaAOX-6BL    --------------------------------------------------------QDNL 102
                                                                            ::.:

AtAOX1a             HLMTFMEVAKPKWYERALVITVQGVFFNAYFLGYLISPKFAHRMVGYLEEEAIHSYTEFL 284
put.regTaAOX-6BL    ERKNFEGKI------KENQETITGYLILAGMLGSFDRPLFGSDQLTFVAG---------- 146
                    .   .*          :    *: * :: * :** :  * *.   : ::

AtAOX1a             KELDKGNIENVPAPAIAIDYWRLPADATLRDVVMVVRADEAHHRDVNHFASDIHYQGREL 344
put.regTaAOX-6BL    ------------------------------------------------------------ 146

AtAOX1a             KEAPAPIGYH    354
put.regTaAOX-6BL    ----------    146
```

TaAOX1c-6AL:

```
AtAOX1a          ----------------------------------MMITR-------------GGAKAAKSL      14
TaAA1505530.1    MPSWRALARRQRHVIPSPSQSLARPQVLEPATTSFASRAAAHQAGSSSSAMSSRVAGSVL      60
                                                  : :*             . .   *.. *

AtAOX1a          LVAAGPRLFSTVRTVSSHEALSASHILKPGVTSAWIWTRAPTIGGMRFASTITLGEKTPM      74
TaAA1505530.1    LRHLGPRVFGPTTPAAQRPLLAGG---EGGAVAVAMWARPLSTSAAEAAREEATASKDNV      117
                 *   ***:*. .  .:.:  *:..   : *..:. :*:*  : .. . *   : ..*  :

AtAOX1a          KEEDANQKKTENESTGGDA-AGGNNKGDKGIASYWGVEPNKITKEDGSEWKWNCFRPWET      133
TaAA1505530.1    ASTAAATAEA-MQAAKADAVQAAKEGKSPAASSYWGIVPAKLVNKDGAEWKWSCFRPWEA      176
                  .  *   ::  ::: .**  ..::  .  . . :****: * *:.::**:****.******:

AtAOX1a          YKADITIDLKKHHVPTTFLDRIAYWTVKSLRWPTDLFFQRRYGCRAMMLETVAAVPGMVG      193
TaAA1505530.1    YTSDTTIDLSKHHKPKVLLDKIAYWTVKSLRVPTDIFFQRRYGCRAMMLETVAAVPGMVG      236
                 *.:* ****.*** *..:**:********** ***:************************

AtAOX1a          GMLLHCKSLRRFEQSGGWIKALLEEAENERMHLMTFMEVAKPKWYERALVITVQGVFFNA      253
TaAA1505530.1    GMLLHLRSLRRFEQSGGWIRALLEEAENERMHLMTFMEVANPKWYERALVLAVQGVFFNA      296
                 *****  :************:*******************:*********::********

AtAOX1a          YFLGYLISPKFAHRMVGYLEEEAIHSYTEFLKELDKGNIENVPAPAIAIDYWRLPADATL      313
TaAA1505530.1    YFLGYIVSPKFAHRVVGYLEEEAIHSYTEFLRDLEAGRIENVPAPRIAIDYWRLPADARL      356
                 *****::*******:****************::*: *.******* ************ *

AtAOX1a          RDVVMVVRADEAHHRDVNHFASDIHYQGRELKEAPAPIGYH-       354
TaAA1505530.1    KDVVTVVRADEAHHRDVNHFAADIHFQGLELNKTPAPLGYH*       397
                 :*** ***************:***:** **:::***:***
```

TaAOX1c-6BL.sv1:

```
AtAOX1a          ------------------------------------------------------------      0
TaAA1593950.1    MDERTQKLSTSQLRARKYSTSQAEPPFIPRLASSARSATPSRRALARRHVVKSPSQSLAR      60


AtAOX1a          ---------MMITR-------------GGAKAAKSLLVAAGPRLFSTVRTVSSHEALSAS      38
TaAA1593950.1    PQVREPTTTSFASRAAAHQAGSSSSAMSSRVAGSVLLRHLGPRVFGPTTPAAQRPLL---      117
                           : :*             ..   *.. **   ***:*. .  .:.:  *

AtAOX1a          HILKPGV-TSAWIWTRAPTIGGMRFASTITLGEKTPMKEEDANQKKTENESTGGDAAGGN      97
TaAA1593950.1    ---AGGEGGAVVVWARPLSTSAAEAAREEAAASKDNVASTAATAEAMQAAKAQAVQAAK      174
                      *   :. :*:*  : .. . *   : ..*  : .  *   :: : :..  . ..:

AtAOX1a          NKGDKGIASYWGVEPNKITKEDGSEWKWNCFRPWETYKADITIDLKKHHVPTTFLDRIAY      157
TaAA1593950.1    EGGKSPVSSYWGIVPAKLVNKDGAEWKWSCFRPWEAYTSDTTIDLTKHHKPKVLLDKIAY      234
                 : *.. ::****: * *:.::**:****.******:*.:* ****.*** *..:**:***

AtAOX1a          WTVKSLRWPTDLFFQRRYGCRAMMLETVAAVPGMVGGMLLHCKSLRRFEQSGGWIKALLE      217
TaAA1593950.1    WTVKSLRVPTDIFFQRRYGCRAMMLETVAAVPGMVGGMLLHLRSLRRFEQSGGWIRALLE      294
                 ******* ***:****************************  :************:****

AtAOX1a          EAENERMHLMTFMEVAKPKWYERALVITVQGVFFNAYFLGYLISPKFAHRMVGYLEEEAI      277
TaAA1593950.1    EAENERMHLMTFMEVAKPKWYERALVLAVQGVFFNAYFLGYIVSPKFAHRVVGYLEEEAI      354
                 **************************::*************::*******:*********

AtAOX1a          HSYTEFLKELDKGNIENVPAPAIAIDYWRLPADATLRDVVMVVRADEAHHRDVNHFASDI      337
TaAA1593950.1    HSYTEFLRDLEAGRIENVPAPRIAIDYWRLPADARLKDVVTVVRADEAHHRDVNHFAADI      414
                 *******::*: *.******* ************ *:*** ****************:**

AtAOX1a          HYQGRELKEAPAPIGYH-      354
TaAA1593950.1    HFQGLELNKTPAPLGYH*      431
                 *:** **:::***:***
```

TaAOX1c-6BL.sv2:

```
AtAOX1a           ------------------------------------------------------------      0
TaAA1593950.2     MDERTQKLSTSQLRARKYSTSQAEPPFIPRLASSARSATPSRRALARRHVVKSPSQSLAR      60


AtAOX1a           ---------MMITRG-------------GAKAAKSLLVAAGPRLFSTVRTVSSHEALSAS      38
TaAA1593950.2     PQVREPTTTSFASRAAAHQAGSSSSAMSSRVAGSVLLRHLGPRVFGPTTPAAQRPLLA--      118
                           : :*.             .  *.. **   ***:*. .  .:.:  *:

AtAOX1a           HILKPGVTSAWIWTRAPTIGGMRFASTITLGEKTPMKEEDANQKKTENESTGGDAAGGNN      98
TaAA1593950.2     ----------------GGEGGAAAASKDNVAST------AAATAEAMQAAKAQAVQAAKE      156
                                    **   **. .:...       *   :: : :..  . ..::

AtAOX1a           KGDKGIASYWGVEPNKITKEDGSEWKWNCFRPWETYKADITIDLKKHHVPTTFLDRIAYW      158
TaAA1593950.2     GGKSPVSSYWGIVPAKLVNKDGAEWKWSCFRPWEAYTSDTTIDLTKHHKPKVLLDKIAYW      216
                   *.. ::****: * *:.::**:****.******:*.:* ****.*** *..:**:****

AtAOX1a           TVKSLRWPTDLFFQRRYGCRAMMLETVAAVPGMVGGMLLHCKSLRRFEQSGGWIKALLEE      218
TaAA1593950.2     TVKSLRVPTDIFFQRRYGCRAMMLETVAAVPGMVGGMLLHLRSLRRFEQSGGWIRALLEE      276
                  ****** ***:***************************** :***********:*****

AtAOX1a           AENERMHLMTFMEVAKPKWYERALVITVQGVFFNAYFLGYLISPKFAHRMVGYLEEEAIH      278
TaAA1593950.2     AENERMHLMTFMEVAKPKWYERALVLAVQGVFFNAYFLGYIVSPKFAHRVVGYLEEEAIH      336
                  *************************::*************::*******:**********

AtAOX1a           SYTEFLKELDKGNIENVPAPAIAIDYWRLPADATLRDVVMVVRADEAHHRDVNHFASDIH      338
TaAA1593950.2     SYTEFLRDLEAGRIENVPAPRIAIDYWRLPADARLKDVVTVVRADEAHHRDVNHFAADIH      396
                  ******::*: *.******* ************ *:*** ****************:***

AtAOX1a           YQGRELKEAPAPIGYH-       354
TaAA1593950.2     FQGLELNKTPAPLGYH*       412
                  :** **:::***:***
```

TaAOX1c-6BL.sv3:

```
AtAOX1a           ------------------------------------------------------------      0
TaAA1593950.3     MDERTQKLSTSQLRARKYSTSQAEPPFIPRLASSARSATPSRRALARRHVVKSPSQSLAR      60


AtAOX1a           ---------MMITR-------------GGAKAAKSLLVAAGPRLFSTVRTVSSHEALSAS      38
TaAA1593950.3     PQVREPTTTSFASRAAAHQAGSSSSAMSSRVAGSVLLRHLGPRVFGPTTPAAQRPLL---      117
                           : :*             ..  *.. **   ***:*. .  .:.:  *

AtAOX1a           HILKPGV-TSAWIWTRAPTIGGMRFASTITLGEKTPMKEEDANQKKTENESTGGDAAGGN      97
TaAA1593950.3     ---AGGEGGAVVVWARPLSTSAAEAAREEAASKDNVASTAAATAEAMQAAKAQAVQAAK      174
                       *   :. :*:*  : .. . *   : ..*  : .  *   :: : :..  . ..:

AtAOX1a           NKGDKGIASYWGVEPNKITKEDGSEWKWNCFRPWETYKADITIDLKKHHVPTTFLDRIAY      157
TaAA1593950.3     EGGKSPVSSYWGIVPAKLVNKDGAEWKWSCFRPWEAYTSDTTIDLTKHHKPKVLLDKIAY      234
                  : *.. ::****: * *:.::**:****.******:*.:* ****.*** *..:**:***

AtAOX1a           WTVKSLRWPTDLFFQRRYGCRAMMLETVAAVPGMVGGMLLHCKSLRRFEQSGGWIKALLE      217
TaAA1593950.3     WTVKSLRVPTDIFFQRRYGCRAMMLETVAAVPGMVGGMLLHLRSLRRFEQSGGWIRALLE      294
                  ******* ***:***************************** :***********:****

AtAOX1a           EAENERMHLMTFMEVAKPKWYERALVITVQGVFFNAYFLGYLISPKFAHRMVGYLEEEAI      277
TaAA1593950.3     EAENERMHLMTFMEVAKPKWYERALVLAVQGVFFNAYFLGYIVSPKFAHRVVGYLEEEAI      354
                  **************************::*************::*******:*********

AtAOX1a           HSYTEFLKELDKGNIENVPAPAIAIDYWRLPADATLRDVVMVVRADEAHHRDVNHFASDI      337
TaAA1593950.3     HSYTEFLRDLEAGRIENVPAPRIAIDYWRLPADARLKDVVTVVRADEAHHRDVNHFAADI      414
                  *******::*: *.******* ************ *:*** ****************:**

AtAOX1a           HYQGRELKEAPAPIGYH-      354
TaAA1593950.3     HFQGLELNKTPAPLGYH*      431
                  *:** **:::***:***
```

TaAOX1c-6DL:

```
AtAOX1a            ---------------------------------MMIT-------------RGGAKAAKSL     14
TaAA1715280.1      MPSWRALARRHRHVIPSPSRSLARPQVLDPATTSFASRAAAHQAGSPSSAMSSRVAGSVL     60
                                                     : :              ..  *.. *

AtAOX1a            LVAAGPRLFSTVRTVSSHEALSASHILKPGVTSAWIWTRAPTIGGMRFASTITLGEKTPM     74
TaAA1715280.1      LRHLGPRVFGPTTQAAQRTLLAG---GEGGAVAMWAWPLSTSAAEAAREEAAA--SKDNV     115
                   *   ***:*. .  .:.:  *:.    : *..: * *  : : .     .: :  .*  :

AtAOX1a            KEEDANQKKTENESTGGDAAGGNNKGDKGIASYWGVEPNKITKEDGSEWKWNCFRPWETY     134
TaAA1715280.1      ASTAAATAEAMQAAKAEAVQAAKEGGKSPASSYWGIVPAKLVNKDGAEWKWSCFRPWEAY     175
                    .  *   :: : :..  . ..:: *..  :****: * *:.::**:****.******:*

AtAOX1a            KADITIDLKKHHVPTTFLDRIAYWTVKSLRWPTDLFFQRRYGCRAMMLETVAAVPGMVGG     194
TaAA1715280.1      TSDTTIDLTKHHKPKVLLDKIAYWTVKSLRVPTDIFFQRRYGCRAMMLETVAAVPGMVGG     235
                   .:* ****.*** *..:**:********** ***:*************************

AtAOX1a            MLLHCKSLRRFEQSGGWIKALLEEAENERMHLMTFMEVAKPKWYERALVITVQGVFFNAY     254
TaAA1715280.1      MLLHLRSLRRFEQSGGWIRALLEEAENERMHLMTFMEVANPKWYERALVLAVQGVFFNAY     295
                   **** :************:********************:*********::*********

AtAOX1a            FLGYLISPKFAHRMVGYLEEEAIHSYTEFLKELDKGNIENVPAPAIAIDYWRLPADATLR     314
TaAA1715280.1      FLGYIVSPKFAHRVVGYLEEEAIHSYTEFLRDLEDGRIENVPAPRIAIDYWRLPPDARLK     355
                   ****::*******:****************::*:.*.******* ********* ** *:

AtAOX1a            DVVMVVRADEAHHRDVNHFASDIHYQGRELKEAPAPIGYH-        354
TaAA1715280.1      DVVTVVRADEAHHRDVNHFAADIHFQGLELNKTPAPLGYH*        395
                   *** ****************:***:** **:::***:***
```

regTaAOX-3B:

```
AtAOX1a            MMITRGGAKAAKSLLVAAGPRLFSTVRTVSSHEALSASHILKPGVTSAWIWTRAPTIGGM     60
TaAA0753740.1      ------------------------------------------------------------     0

AtAOX1a            RFASTITLGEKTPMKEEDANQKKTENESTGGDAAGGNNKGDKGIASYWGVEPNKITKEDG     120
TaAA0753740.1      ------------------------------------------------------------     0

AtAOX1a            SEWKWNCFRPWETYKADITIDLKKHHVPTTFLDRIAYWTVKSLRWPTDLFFQRRYGCRAM     180
TaAA0753740.1      ------------------------------------------------------------     0

AtAOX1a            MLETVAAVPGMVGGMLLHCKSLRRFEQSGGWIKALLEEAENERMHLMTFMEVAKPKWYER     240
TaAA0753740.1      -------------------------------------------MHLMTFMEVSQPRWYER     17
                                                              *********::*:****

AtAOX1a            ALVITVQGVFFNAYFLGYLISPKFAHRMVGYLEEEAIHSYTEFLKELDKGNIENVPAPAI     300
TaAA0753740.1      ALVVAVQGVFFHAYLATYLASPKVAHRMVGYLEEEAVHSYTEFLRDLEAGKIDDVPAPTR     77
                   ***::******:**:  ** ***.************:*******::*: *:*::****:

AtAOX1a            AIDYWRLPADATLRDVVMVVRADEAHHRDVNHFASDIHYQGRELKEAPAPIGYH-   354
TaAA0753740.1      R------------------------TTGTSNHYASDIHCQGHALREVAAPIGYH*   107
                                                 **:***** **: *:*. ******
```

put.TaAOX1e-3DS:

```
AtAOX1a            MMITRGGAKAAKSLLVAAGPRLFSTVRTVSSHEALSASHILKPGVTSAWIWTRAPTIGGM 60
put.TaAOX1e-3DS    ------------------------------------------------------------ 0


AtAOX1a            RFASTITLGEKTPMKEEDANQKKTENESTGGDAAGGNNKGDKGIASYWGVEPNKITKEDG 120
put.TaAOX1e-3DS    --------------------------------MAATLKKGEEEAASYWGVAPARLVKEDG 28
                                                    *.   :**::   ****** *  ::.****

AtAOX1a            SEWKWNCFRPWETYKADITIDLKKHHVPTTFLDRIAYWTVKSLRWPTDLFFQRRYGCRAM 180
put.TaAOX1e-3DS    TEWKWSCFRPWDAYEADVSIVLTKHHRPATFGDKVALWTVKAIRWPTDLFFQRRYGCRAM 88
                   :****.*****::*:**::* *.*** *:** *::* ****::*****************

AtAOX1a            MLETVAAVPGMVGGMLLHCKSLRRFEQSGGWIKALLEEAENERMHLMTFMEVAKPKWYER 240
put.TaAOX1e-3DS    MLETVAAVPGMVARAVLHLRSLRRFEQSGEWIRALLEEAQNERMHLMTFMEVSQPRWYER 148
                   ************.   :**  :********* **:******:************::*:****

AtAOX1a            ALVITVQGVFFNAYFLGYLISPKFAHRMVGYLEEEAIHSYTEFLKELDKGNIENVPAPAI 300
put.TaAOX1e-3DS    ALVVVVQGVFFHAYLATYLASPKVAHRMVGYLEEEAVHSYTEFLRDLEAGKIDDVPAPAI 208
                   ***:.******:**:   ** ***.************:*******::*: *:*::******

AtAOX1a            AIDYWRLPADATLRDVVMVVRADEAHHRDVNHFASDIHYQGRELKEAPAPIGYH354
put.TaAOX1e-3DS    AIDYWRLPAGATLKDVVRVVRADEAHHRDVNHYASDIHCQGHALREVAAPIGYH262
                   *********.***:*** **************:***** **: *:*. ******
```

TaAOX1d-2AL.1:

```
AtAOX1a            MMITRGGAKAAKSLLVAAGPRLFSTVRTVSSHEALSASHILKPGVTSAWIWTRAPTIGGM      60
TaAA0302070.1      -----------------------------------------------------MPAAAR-      6
                                                                        *:  .

AtAOX1a            RFASTITLGEKTPMKEEDANQKKTENESTGGDAAGGNNKGDKGIASYWGVEPNKITKEDG      120
TaAA0302070.1      IFPARMA------STEAAAPHAKQEEATEKPQGATTPEHNKKAVVSYWGIEPRKLVKDDG      60
                    * : ::      .*  * : * *: :   :.*   ::..*.:.****:**.*:.*:**

AtAOX1a            SEWKWNCFRPWETYKADITIDLKKHHVPTTFLDRIAYWTVKSLRWPTDLFFQRRYGCRAM      180
TaAA0302070.1      TEWTWFSFRPWDTYRPDTSIDMAKHHEPRAVADKVAYLIVRTLRAGSDLFFQRRHASHAL      120
                   :**.* .****:**: * :**: *** * :. *::**  *::**  :*******:..:*:

AtAOX1a            MLETVAAVPGMVGGMLLHCKSLRRFEQSGGWIKALLEEAENERMHLMTFMEVAKPKWYER      240
TaAA0302070.1      LLEMVAAVPPMVGGVLLHLRSLRRFEHSSGWIRALMEEAENERMHLMTFMEVTQPLWWER      180
                   :** ***** ****:*** :******:*.***:**:****************::* *:**

AtAOX1a            ALVITVQGVFFNAYFLGYLISPKFAHRMVGYLEEEAIHSYTEFLKELDKGNIENVPAPAI      300
TaAA0302070.1      ALVLATQGVFFNAYFVGYLVSPKFAHRFVGYLEEEAVHSYTKYLKDLEAGLIENTPAPAI      240
                   ***::.*********:***:*******:********:****::**:*: * ***.*****

AtAOX1a            AIDYWRLPADATLRDVVMVVRADEAHHRDVNHFASDIHYQGRELKEAPAPIGYH-   354
TaAA0302070.1      AIDYWRLPADARLKDVVTAVRADEAHHRDANHYASDIHYQGMTLNQTPAPLGYH*   294
                   *********** *:***  .**********.**:********  *:::***:***
```

TaAOX1d-2AL.2.sv1:

```
AtAOX1a              MMITRGGAKAAKSLLVAAGPRLFSTVRTVSSHEALSASHILKPGVTSAWIWTRAPTIGGM      60
TaAA0282360.1        ----MSSRMAGATLLRHLGPRLFAAAEPASGLAASA-RGIM-PA---------AARIFPA      45
                         ..  *. :**   *****::.. .*.  * :   *: *.         *  *

AtAOX1a              RFASTITLGEKTPMKEEDANQ-KKTENESTGGDAAGGNNKGDKGIASYWGVEPNKITKED      119
TaAA0282360.1        RMAST----------EAAAPHAKQEDDAASPQAAATPEQQNKKPVVSYWGIEPRKLVKDD      95
                     *:***          *  * : *: :: ::   **  :::..* :.****:**.*:.*:*

AtAOX1a              GSEWKWNCFRPWETYKADITIDLKKHHVPTTFLDRIAYWTVKSLRWPTDLFFQRRYGCRA      179
TaAA0282360.1        GTEWPWFCFRPWDTYRPDTSIEVAKHHEPKALADKVAYFVVRSLRVPRDLFFQRRHASHA      155
                     *:** * *****:**: * :*:: *** *.:: *::**:.*:*** * *******:..:*

AtAOX1a              MMLETVAAVPGMVGGMLLHCKSLRRFEQSGGWIKALLEEAENERMHLMTFMEVAKPKWYE      239
TaAA0282360.1        LLLETVAAVPPMVGGVLLHLRSLRRFEHSGGWIRALMEEAENERMHLMTFMEVTQPRWWE      215
                     ::******** ****:*** :******:*****:**:****************::*:*:*

AtAOX1a              RALVITVQGVFFNAYFLGYLISPKFAHRMVGYLEEEAIHSYTEFLKELDKGNIENVPAPA      299
TaAA0282360.1        RALVLAAQGVFFNAYFVGYLISPKFAHRFVGYLEEEAVESYTEYLKDLEAGLIENTPAPA      275
                     ****::.*********:***********:********:.****:**:*: * ***.****

AtAOX1a              IAIDYWRLPADATLRDVVMVVRADEAHHRDVNHFASDIHYQGRELKEAPAPIGYH-  354
TaAA0282360.1        IAIDYWRLPADARLKDVVTAVRADEAHHRDANHYASDVHYQGMTLNQSPAPLGYH*  330
                     ************ *:*** .**********.**:***:****  *:::***:***
```

TaAOX1d-2AL.2.sv2:

```
AtAOX1a              MMITRGGAKAAKSLLVAAGPRLFSTVRTVSSHEALSASHILKPGVTSAWIWTRAPTIGGM      60
TaAA0282360.2        ----MSSRMAGATLLRHLGPRLFAAAEPASGLAASA-RGIM-PA---------AARIFPA      45
                         ..  *. :**   *****::.. .*.  * :   *: *.         *  *

AtAOX1a              RFASTITLGEKTPMKEEDANQ-KKTENESTGGDAAGGNNKGDKGIASYWGVEPNKITKED      119
TaAA0282360.2        RMAST----------EAAAPHAKQEDDAASPQAAATPEQQNKKPVVSYWGIEPRKLVKDD      95
                     *:***          *  * : *: :: ::   **  :::..* :.****:**.*:.*:*

AtAOX1a              GSEWKWNCFRPWETYKADITIDLKKHHVPTTFLDRIAYWTVKSLRWPTDLFFQRRYGCRA      179
TaAA0282360.2        GTEWPWFCFRPWDTYRPDTSIEVAKHHEPKALADKVAYFVVRSLRVPRDLFFQRRHASHA      155
                     *:** * *****:**: * :*:: *** *.:: *::**:.*:*** * *******:..:*

AtAOX1a              MMLETVAAVPGMVGGMLLHCKSLRRFEQSGGWIKALLEEAENERMHLMTFMEVAKPKWYE      239
TaAA0282360.2        LLLETVAAVPPMVGGVLLHLRSLRRFEHSGGWIRALMEEAENERMHLMTFMEVTQPRWWE      215
                     ::******** ****:*** :******:*****:**:****************::*:*:*

AtAOX1a              RALVITVQGVFFNAYFLGYLISPKFAHRMVGYLEEEAIHSYTEFLKELDKGNIENVPAPA      299
TaAA0282360.2        RALVLAAQGVFFNAYFVGYLISPKFAHRFVGYLEEEAVESYTEYLKDLEAGLIENTPAPA      275
                     ****::.*********:***********:********:.****:**:*: * ***.****

AtAOX1a              IAIDYWRLPADATLRDVVMVVRADEAHHRDVNHFASDIHYQGRELKEAPAPIGYH-  354
TaAA0282360.2        IAIDYWRLPADARLKDVVTAVRADEAHHRDANHYASDVHYQGMTLNQSPAPLGYH*  330
                     ************ *:*** .**********.**:***:****  *:::***:***
```

TaAOX1d-2DL:

```
AtAOX1a            MMITRGGAKAAKSLLVAAGPRLFSTVRTVSSHEALSASHILKPGVTSAWIWTRAPTIGGM      60
TaAA0562440.1      --------MAGATLLRHLGPRLFAAAEPASGLAASA-RGIM-PA---------AARIFPA      41
                            *. :**   *****::.. .*.  * :   *: *.         *  *

AtAOX1a            RFASTITLGEKTPMKEEDANQ-KKTENESTGGDAAGGNNKGDKGIASYWGVEPNKITKED      119
TaAA0562440.1      RMAST----------EAAAPHAKQEDDAASPQAAATPEQQNKKPVVSYWGIEPRKLVKDD      91
                   *:***          *  * : *: :: ::   **  :::..* :.****:**.*:.*:*

AtAOX1a            GSEWKWNCFRPWETYKADITIDLKKHHVPTTFLDRIAYWTVKSLRWPTDLFFQRRYGCRA      179
TaAA0562440.1      GTEWPWFCFRPWDTYRPDTSIDVTKHHLPKALADKVAYFVVRSLRVPRDLFFQRRHASHA      151
                   *:** * *****:**: * :**:.***:*.:: *::**:.*:*** * *******:..:*

AtAOX1a            MMLETVAAVPGMVGGMLLHCKSLRRFEQSGGWIKALLEEAENERMHLMTFMEVAKPKWYE      239
TaAA0562440.1      LLLETVAAVPPMVGGVLLHLRSLRRFEHSGGWIRALMEEAENERMHLMTFMEVTQPRWWE      211
                   ::******** ****:*** :******:*****:**:****************::*:*:*

AtAOX1a            RALVITVQGVFFNAYFLGYLISPKFAHRMVGYLEEEAIHSYTEFLKELDKGNIENVPAPA      299
TaAA0562440.1      RALVLAAQGVFFNAYFVGYLISPKFAHRFVGYLEEEAVESYTEYLKDLEAGLIENTPAPA      271
                   ****::.*********:***********:********:.****:**:*: * ***.****

AtAOX1a            IAIDYWRLPADATLRDVVMVVRADEAHHRDVNHFASDIHYQGRELKEAPAPIGYH-  354
TaAA0562440.1      IAIDYWRLPADARLKDVVTAVRADEAHHRDANHYASDIHYQGMTLNQTPAPLGYH*  326
                   ************ *:*** .**********.**:********  *:::***:***
```

put.TaAOX1d-like-4AS:

```
AtAOX1a              MMITRGGAKAAKSLLVAAGPRLFSTVRTVSSHEALSASHILKPGVTSAWIWTRAPTIGGM      60
putTaAOX1d-like-4AS  -----------------------------------------------------MPTTARI      7
                                                                           ** . :

AtAOX1a              RFASTITLGEKTPMKEEDANQKKTENESTG-GDAAGGNNKGDKGIASYWGVEPNKITKED      119
putTaAOX1d-like-4AS  -FPAR--------MASTAAGPHAKQEEATGKPQGATTPEQNKKAVPSYWGIKPRKLVEDD      58
                      * :         * .  *. : .::*:**  :.*   ::..*.: ****::*.*:.::*

AtAOX1a              GSEWKWNCFRPWETYKADITIDLKKHHVPTTFLDRIAYWTVKSLRWPTDLFFQRRYGCRA      179
putTaAOX1d-like-4AS  GTEWSWFSFRPWD----------------------------------TDLFFQRRHASHM      84
                     *:**.* .****:                                  ********:..:

AtAOX1a              MMLETVAAVPGMVGGMLLHCKSLRRFEQSGGWIKALLEEAENERMHLMTFMEVAKPKWYE      239
putTaAOX1d-like-4AS  LLLETVAAVPPMVGGVLLHLRSLRRFEHNGGWIRALMEEAQNERMHLMTFMEVTQPLWCE      144
                     ::******** ****:*** :******:.****:**:***:************::* * *

AtAOX1a              RALVITVQGVFFNAYFLGYLISPKFAHRMVGYLEEEAIHSYTEFLKELDKGNIENVPAPA      299
putTaAOX1d-like-4AS  RALVLPTQGVFFNAYFIGYLVSPKFAHRFVGYLEEEAVH----------------[redacted]----      183
                     ****: .*********:***:*******:********:*

AtAOX1a              IAIDYWRLPADATLRDVVMVVRADEAHHRDVNHFASDIHYQGRELKEAPAPIGYH    354
putTaAOX1d-like-4AS  -------------------------------------------------------    183
```

**Diploids:**

TuAOX1a:

```
AtAOX1a         MMITRGGAKAAKSLLVAAGPRLFSTVRTVSSHEALSASHILKPGVTSAWIWTRAPTIGGM      60
TRIUR3_10307    ------------------------------------------------------------      0

AtAOX1a         RFASTITLGEKTPMKEEDANQKKTENESTGGDAAGGNNKGDKGIASYWGVEPNKITKEDG      120
TRIUR3_10307    ------------------------------------------------------------      0

AtAOX1a         SEWKWNCFRPWETYKADITIDLKKHHVPTTFLDRIAYWTVKSLRWPTDLFFQRRYGCRAM      180
TRIUR3_10307    ------------------------------MLDKIAYYTVKSLRFPTDIFFQRRYGCRAM      30
                                              :**:***:******:***:**********

AtAOX1a         MLETVAAVPGMVGGMLLHCKSLRRFEQSGGWIKALLEEAENERMHLMTFMEVAKPKWYER      240
TRIUR3_10307    MLETVAAVPGMVGGMLLHLRSLRRFEQSGGWIRALLEEAENERMHLMTFMEVAQPRWYER      90
                ****************** :***********:*******************:*:****

AtAOX1a         ALVITVQGVFFNAYFLGYLISPKFAHRMVGYLEEEAIHSYTEFLKELDKGNIENVPAPAI      300
TRIUR3_10307    ALVIAVQGVFFNAYFFGYLISPKFAHRVVGYLEEEAVHSYTEFLKDLDDGKIDNVPAPAI      150
                ****:**********:***********:********:********:**.*:*:*******

AtAOX1a         AIDYWRLPADATLRDVVMVVRADEAHHRDVNHFASDIHYQGRELKEAPAPIGYH     354
TRIUR3_10307    AIDYWRLPANATLKDVVTVVRADEAHHRDVNHFASDVYYQGMQLKATPAPIGYH     204
                *********:***:*** *****************::*** :** :*******
```

TuAOX1c:

```
AtAOX1a         ----------------------------------------------MMITRGGAKAAKS      13
TRIUR3_08189    MTQSFNNEAYMPTMGVGFNNSHWSQINDMHLDDHEFEVDEDGEGIVDAPKGRGGNYTNEE      60
                                                                ***  : :.

AtAOX1a         LLVAAGPRLFSTVRTVSSHEALSASHILKPGVTSAWIWT---------------------      52
TRIUR3_08189    DVLLCN-----T--------WLQVSRDRPSVGDCQKWVAAQMAVDKLNSSGINDEDRRDGM      107
                 :: ..     *          *..*:  . *  . *: :

AtAOX1a         ----------------------------RAPTIGGMRFASTITLGEKTPMKEEDANQKKT      84
TRIUR3_08189    DDLDMSNKHMQTIDLDEEEEEASSDDGKRSPTPNSVSYSKPKRLD----VCKKDAKEKKK      163
                                            *:** ..: ::.   *.    : ::**::**.

AtAOX1a         -ENESTGGDAAGGNNKGDKGIASYWGVEPNKITKEDGSEWKWNCFRPWETYKADITIDLK      143
TRIUR3_08189    RKRDDELKNAMKTIVKGRKEANEVRKMARNQDAAAEERKVTLEERKPWEAYTSDTTIDLS      223
                 :.:.   :*     ** *    .    :  *: :  :  : . :  :***:*.:* ****.

AtAOX1a         KHHVPTTFLDRIAYWTVKSLRWPTDLFFQRRYGCRAMMLETVAAVPGMVGGMLLHCKSLR      203
TRIUR3_08189    KHHKPKVLLDKIAYWTVKSLRVPTDIFFQRRYGCRAMMLETVAAVPGMVGGMLLHLRSLR      283
                *** *..:**:********** ***:***************************** :***

AtAOX1a         RFEQSGGWIKALLEEAENERMHLMTFMEVAKPKWYERALVITVQGVFFNAYFLGYLISPK      263
TRIUR3_08189    RFEQSGGWIRALLEEAENERMHLMTFMEVANPKWYERALVLAVQGVFFNAYFLGYIVSPK      343
                *********:********************:*********::*************::***

AtAOX1a         FAHRMVGYLEEEAIHSYTEFLKELDKGNIENVPAPAIAIDYWRLPADATLRDVVMVVRAD      323
TRIUR3_08189    FAHRVVGYLEEEAIHSYTEFLRDLEAGRIENVPAPRIAIDYWRLPADARLKDVVTVVRAD      403
                ****:****************::*: *.******* *********** *:*** *****

AtAOX1a         EAHHRDVNHFASDIHYQGRELKEAPAPIGYH  354
TRIUR3_08189    EAHHRDVNHFAADIHFQGLELNKTPAPLGYH  434
                ***********:***:** **:::***:***
```

## TuAOX1d.1:

```
AtAOX1a         MMITRGGAKAAKSLLVAAGPRLFSTVRTVSSHEALSASHILKPGVTSAWIWTRAPTIGGM      60
TRIUR3_12374    -----------------------------------------------------------M      1
                                                                           *

AtAOX1a         RFASTITLGEKTPMKEEDAN-QKKTENESTGGDAAGGNNKGDKGIASYWGVEPNKITKED      119
TRIUR3_12374    PAAARIFPAR-MASTEAAAPHAKQEDDAASPQAAATPEQQNKKPVVSYWGIEPRKLVKDD      60
                  *: *  ..     .*  *    *: :: ::    **  :::..* :.****:**.*:.*:*

AtAOX1a         GSEWKWNCFRPWETYKADITIDLKKHHVPTTFLDRIAYWTVKSLRWPTDLFFQRRYGCRA      179
TRIUR3_12374    GTEWPWFCFRPWDTYRPDTSIDVAKHHEPKALADKVAYFVVRSLRVPRDLFFQRRHASHA      120
                *:** * *****:**: * :**: *** *.:: *::**:.*:*** * *******:..:*

AtAOX1a         MMLETVAAVPGMVGGMLLHCKSLRRFEQSGGWIKALLEEAENERMHLMTFMEVAKPKWYE      239
TRIUR3_12374    LLLETVAAVPPMVGGVLLHLRSLRRFEHSGGWIRALMEEAENERMHLMTFMEVTQPRWWE      180
                ::******** ****:*** :******:*****:**:****************::*:*:*

AtAOX1a         RALVITVQGVFFNAYFLGYLISPKFAHRMVGYLEEEAIHSYTEFLKELDKGNIENVPAPA      299
TRIUR3_12374    RALVLAAQGVFFNAYFVGYLISPKFAHRFVGYLEEEAVESYTEYLKDLEAGLIENTPAPA      240
                ****::.*********:***********:********:.****:**:*: * ***.****

AtAOX1a         IAIDYWRLPADATLRDVVMVVRADEAHHRDVNHFASDIHYQGRELKEAPAPIGYH    354
TRIUR3_12374    IAIDYWRLPADARLKDVVTAVRADEAHHRDANHYASDVHYQGMTLNQSPAPLGYH    295
                ************ *:*** .**********.**:***:****  *:::***:***
```

## TuAOX1d.2:

```
AtAOX1a         ------------------------------------------------------------      0
TRIUR3_19476    MARKPVGGAAAAAPAPPRAAPGRGADPAERRRRWCGITVRGALVMLFPIAVSFLFSFIFG      60

AtAOX1a         -MMITRGGAKAAKSLLVAAGPRLFSTVRTVSSHEALSASHILKPGVTSAWIWTRAPTIGG      59
TRIUR3_19476    IAGLLLGGLSSNASVSMPSTCRILSTANTMSSR------------MAGATLLRRAASARG      108
                   :  ** .:  *: : :  *::**..*:**:            ::.* :  ** :  *

AtAOX1a         MRFASTITLGEKTPMKEEDANQKKTENESTGGDAAGGNNKGDKGIASYWGVEPNKITKED      119
TRIUR3_19476    IMPAAARVFPARMASTEAAGPRAKQEEATEKPQGATAPEQNKKAVPSYWGIEPRKLVKDD      168
                :  *:: .:  :   .*  . : * *: :   :.* . ::..*.: ****:**.*:.*:*

AtAOX1a         GSEWKWNCFRPWETYKADITIDLKKHHVPTTFLDRIAYWTVKSLRWPTDLFFQRRYGCRA      179
TRIUR3_19476    GTEWPWFSFRPWDTYRPDTSIDVAKHHEPRAVADKVAYLIVRTLRKGSDLFFQRRHASHA      228
                *:** * .****:**: * :**: *** * :. *::**  *::**  :*******:..:*

AtAOX1a         MMLETVAAVPGMVGGMLLHCKSLRRFEQSGGWIKALLEEAENERMHLMTFMEVAKPKWYE      239
TRIUR3_19476    LLLETVAAVPPMVGGVLLHLRSLRRFEHSGGWIRALMEEAENERMHLMTFMEVTQPLWWE      288
                ::******** ****:*** :******:*****:**:****************::* *:*

AtAOX1a         RALVITVQGVFFNAYFLGYLISPKFAHRMVGYLEEEAIHSYTEFLKELDKGNIENVPAPA      299
TRIUR3_19476    RALVLATQGVFFNAYFVGYLVSPKFAHRFVGYLEEEAVHSYTEYLKDLEAGLIENTPAPA      348
                ****::.*********:***:*******:********:*****:**:*: * ***.****

AtAOX1a         IAIDYWRLPADATLRDVVMVVRADEAHHRDVNHFASDIHYQGRELKEAPAPIGYH    354
TRIUR3_19476    IAIDYWRLPADARLKDVVTAVRADEAHHRDANHYASDIHYQGMTLNQTPAPLGYH    403
                ************ *:*** .**********.**:********  *:::***:***
```

## AetAOX1a:

```
AtAOX1a       MMITRGGAKAAKSLLVAAGPRLFSTVRTVSSHEALSASHILKPGVTSAWIWTRAPTIGGM  60
EMT12983      ------------------------------------------------------------  0

AtAOX1a       RFASTITLGEKTPMKEEDANQKKTENESTGGDAAGGNNKGDKGIASYWGVEPNKITKEDG  120
EMT12983      ------------------------------------------------------------  0

AtAOX1a       SEWKWNCFRPWETYKADITIDLKKHHVPTTFLDRIAYWTVKSLRWPTDLFFQRRYGCRAM  180
EMT12983      ------------------------------MLDKIAYYTVKSLRFPTDIFFQRRYGCRAM  30
                                            :**:***:******:***:***********

AtAOX1a       MLETVAAVPGMVGGMLLHCKSLRRFEQSGGWIKALLEEAENERMHLMTFMEVAKPKWYER  240
EMT12983      MLETVAAVPGMVGGMLLHLRSLRRFEQSGGWIRALLEEAENERMHLMTFMEVAQPRWYER  90
              ****************** :************:********************:*:****

AtAOX1a       ALVITVQGVFFNAYFLGYLISPKFAHRMVGYLEEEAIHSYTEFLKELDKGNIENVPAPAI  300
EMT12983      ALVIAVQGVFFNAYFFGYLISPKFAHRVVGYLEEEAVHSYTEFLKDLDDGKIDNVPAPAI  150
              ****:**********:***********:********:********:**.*:*:*******

AtAOX1a       AIDYWRLPADATLRDVVMVVRADEAHHRDVNHFASDIHYQGRELKEAPAPIGYH        354
EMT12983      AIDYWRLPANATLKDVVTVVRADEAHHRDVNHFASDVYYQGMQLKATPAPIGYH        204
              *********:***:*** ******************::*** :** :*******
```

## AetAOX1e:

```
AtAOX1a       --MMITRGGAKAAKSLLVAAGPRLFSTVRTVSSHEALSASHILKPGVTSAWIWTRAPTIG  58
EMT10169      MAIFLPTKSW-RCNSILS---QIFIATQSTIRSHAAWHCDE-LVHEVTSHNLWSTEDAST  55
                :::   .   .:*:*      :::*  *: ** *  ... *   ***  :*:   :

AtAOX1a       GMRFAST--------ITLGEKTPMKEEDA------NQKKTENESTGGDAAGGNNKGDKGI  104
EMT10169      CMQKEKQRGSARNGHGAVGSAARREGGGARFFSVAGRSPAALGVGAARTAATLKQGEKEA  115
               *:  .          ::*. :  :  .*      .:. :     .. :*.  ::*:*

AtAOX1a       ASYWGVEPNKITKEDGSEWKWNCFRPWETYKADITIDLKKHHVPTTFLDRIAYWTVKSLR  164
EMT10169      ASYWGVAPARLVKEDGTEWKWSCFRPWDAYEADVSIDLTKHHRPATLGDKVALWTVKAMR  175
              ****** * ::.****:****.*****::*:**::***.*** *:*: *::* ****::*

AtAOX1a       WPTDLFFQRRYGCRAMMLETVAAVPGMVGGMLLHCKSLRRFEQSGGWIKALLEEAENERM  224
EMT10169      WPTDLFFQRRYGCRAMMLETVAAVPGMVAGAVLHLRSLRRFEQSGGWIRALLEEAENERM  235
              ****************************.* :** :************:***********

AtAOX1a       HLMTFMEVAKPKWYERALVITVQGVFFNAYFLGYLISPKFAHRMVGYLEEEAIHSYTEFL  284
EMT10169      HLMTFMEVSQPRWYERALVVAVQGVFFHAYLATYLASPKVAHRMVGYLEEEAVHSYTEFL  295
              ********::*:*******::******:**:  ** ***.************:*******

AtAOX1a       KELDKGNIENVPAPAIAIDYWRLPADATLRDVVMVVRADEAHHRDVNHFASDIHYQGREL  344
EMT10169      RDLEAGKIDGVPAPAIAIDYWRLPAGATLKDVVRVVRADEAHHRDVNHYASDIHCQGHAL  355
              ::*: *:*:.***************.***:*** **************:***** **: *

AtAOX1a       KEAPAPIGYH  354
EMT10169      REVAAPIGYH  365
              :*. ******
```

AetAOX1d:

```
AtAOX1a       MMITRGGAKAAKSLLVAAGPRLFSTVRTVSSHEALSASHILKPGVTSAWIWTRAPTIGGM   60
EMT02184      -----------------------------------------------------------M   1
                                                                         *

AtAOX1a       RFASTITLGEKTPMKEEDAN-QKKTENESTGGDAAGGNNKGDKGIASYWGVEPNKITKED   119
EMT02184      PAAARIFPAR-MASTEAAAPHAKQEDDAASPQAAATPEQQNKKPVVSYWGIEPRKLVKDD   60
                *: *  ..    .*  *   *: :: ::   **  :::..* :.****:**.*:.*:*

AtAOX1a       GSEWKWNCFRPWETYKADITIDLKKHHVPTTFLDRIAYWTVKSLRWPTDLFFQRRYGCRA   179
EMT02184      GTEWPWFCFRPWDTYRPDTSIDVTKHHEPKALADKVAYFVVRSLRVPRDLFFQRRHASHA   120
              *:** * *****:**: * :**:.*** *.:: *::**:.*:*** * *******:..:*

AtAOX1a       MMLETVAAVPGMVGGMLLHCKSLRRFEQSGGWIKALLEEAENERMHLMTFMEVAKPKWYE   239
EMT02184      LLLETVAAVPPMVGGVLLHLRSLRRFEHSGGWIRALMEEAENERMHLMTFMDVTQPRWWE   180
              ::******** ****:*** :******:*****:**:**************:*::*:*:*

AtAOX1a       RALVITVQGVFFNAYFLGYLISPKFAHRMVGYLEEEAIHSYTEFLKELDKGNIENVPAPA   299
EMT02184      RALVLAAQGVFFNAYFVGYLISPKFAHRFVGYLEEEAVESYTEYLKDLEAGLIENTPAPA   240
              ****::.*********:***********:********:.****:**:*: * ***.****

AtAOX1a       IAIDYWRLPADATLRDVVMVVRADEAHHRDVNHFASDIHYQGRELKEAPAPIGYH        354
EMT02184      IAIDYWRLPADARLKDVVTAVRADEAHHRDANHYASDIHYQGMTLNQTPAPLGYH        295
              ************ *:*** .**********.**:********  *:::***:***
```

AetAOX1d-like:

```
AtAOX1a       MMITRGGAKAAKSLLVAAGPRLFSTVRTVSSHEALSASHILKPGVTSAWIWTRAPTIGGM   60
EMT02460      ----MSSRMAGATLLRHLGPHLFAAAEPAS----------------------------GL   28
                   ..  *. :**   **:**::.. .*                            *:

AtAOX1a       RFASTITLGEKTPMKEEDANQKKTENESTGGDAAGGNNKGDKGIASYWGVEPNKITKEDG   120
EMT02460      AA------------SARGGAHAKQEGDAEKPESATAPEQNKKPVASYWGIEPRKLVKDDG   76
                            . ... : * *.::   ::* . ::..* :*****:**.*:.*:**

AtAOX1a       SEWKWNCFRPWETYKADIT-----------------------------------------   139
EMT02460      TEWPWFSFRPWDTYRPDTSIDVAKHHEPRAVADKPRRKLVKDDGTEWPWFSFRPWDTYRP   136
              :** * .****:**: * :

AtAOX1a       ---IDLKKHHVPTTFLDRIAYWTVKSLRWPTDLFFQRRYGCRAMMLETVAAVPGMVGGML   196
EMT02460      DTSIDVAKHHEPRAVADKVAYLIVRTLRAGSDLFFQRRHASHALLLETVAAVPPMVGGVL   196
                 **: *** * :. *::**  *::**  :*******:..:*::******** ****:*

AtAOX1a       LHCKSLRRFEQSGGWIKALLEEAENERMHLMTFMEVAKPKWYERALVITVQGVFFNAYFL   256
EMT02460      LHLRSLRRFEHSGGWIRALMEEAENERMHLMTFME----------------GVFFNAYFV   240
              ** :******:*****:**:***************                ********:

AtAOX1a       GYLISPKFAHRMVGYLEEEAIHSYTEFLKELDKGNIENVPAPAIAIDYWRLPADATLRDV   316
EMT02460      GYLISPKL-------------------------------------------------KDV   251
              *******:                                                 :**

AtAOX1a       VMVVRADEAHHRDVNHFASDIHYQGRELKEAPAPIGYH       354
EMT02460      VIAVRADEAHHRDANHYASDIHYQGMTLNQTPAPLGYH       289
              *:.**********.**:********  *:::***:***
```

**Non-Expressed:**

## ne.TaAOX1d-2BL.1:

```
AtAOX1a             MMITRGGAKAAKSLLVAAGPRLFSTVRTVSSHEALSASHILKPGVTSAWIWTRAPTIGGM 60
ne.TaAOX1d-2BL.1    ----MSSRMAGATLLRHLGPRLFAAAEPASGLAASA-RGIM-PA---------AARIFPA 45
                         ..  *. :**   *****::.. .*.  * :   *: *.         *  *

AtAOX1a             RFASTITLGEKTPMKEEDANQKKTENESTGGDAAGGNNKGDKGIASYWGVEPNKITKEDG 120
ne.TaAOX1d-2BL.1    RMASTE---------AAAPHAKQEDDAGTPQAAATPEQQSKKAVVSYWGIEPRKLVKEDG 96
                    *:***              : *: :: .*   **  :::..*.:.****:**.*:.****

AtAOX1a             SEWKWNCFRPWETYKADITIDLKKHHVPTTFLDRIAYWTVKSLRWPTDLFFQRRYGCRAM 180
ne.TaAOX1d-2BL.1    TEWPWFCFRPWDTYRPDTSIDVTKHHEPKALADKVAYFVVRSLRVPRDLFFQRRHASHAL 156
                    :** * *****:**: * :**:.*** *.:: *::**:.*:*** * *******:..:*:

AtAOX1a             MLETVAAVPGMVGGMLLHCKSLRRFEQSGGWIKALLEEAENERMHLMTFMEVAKPKWYER 240
ne.TaAOX1d-2BL.1    LLETVAAVPPMVGGVLLHLRSLRRFEHSGGWIRALMEEAENERMHLMTFMEVTQPRWWER 216
                    :******** ****:*** :******:*****:**:****************::*:*:**

AtAOX1a             ALVITVQGVFFNAYFLGYLISPKFAHRMVGYLEEEAIHSYTEFLKELDKGNIENVPAPAI 300
ne.TaAOX1d-2BL.1    ALVLAAQGVFFNAYFVGYLISPKFAHRFVGYLEEEAVESYTEYLKDLEAGLIENTPAPAI 276
                    ***::.*********:***********:********:.****:**:*: * ***.*****

AtAOX1a             AIDYWRLPADATLRDVVMVVRADEAHHRDVNHFASDIHYQGRELKEAPAPIGYH-      354
ne.TaAOX1d-2BL.1    AIDYWRLPADARLKDVVTAVRADEAHHRDANHYASDIHYQGMTLNQTPAPLGYH*      330
                    *********** *:*** .**********.**:********  *:::***:***
```

## ne.TaAOX1d-2BL.2:

```
AtAOX1a             MMITRGGAKAAKSLLVAAGPRLFSTVRTVSSHEALSASHILKPGVTSAWIWTRAPTIGGM 60
ne.TaAOX1d-2BL.2    ----MSSRMAGATLLRHLGPRLFAAAEPASGLAASA-RGIM-P---------AAARIFPA 45
                         ..  *. :**   *****::.. .*.  * :   *: *          *  *

AtAOX1a             RFASTITLGEKTPMKEEDANQKKTENESTGGDAAGGNNKGDKGIASYWGVEPNKITKEDG 120
ne.TaAOX1d-2BL.2    RMASTEAAGPRA----------KQEEATEKPQGATTPEQNKKAVVSYWGIEPRKLVKDDG 95
                    *:*** : * ::          * *: :   :.*   ::..*.:.****:**.*:.*:**

AtAOX1a             SEWKWNCFRPWETYKADITIDLKKHHVPTTFLDRIAYWTVKSLRWPTDLFFQRRYGCRAM 180
ne.TaAOX1d-2BL.2    TEWPWFSFRPWDTYRPDTSIDVAKHHEPRAVADKVAYLIVRTLRKGSDLFFQRRHASHAL 155
                    :** * .****:**: * :**: *** * :. *::**  *::**  :*******:..:*:

AtAOX1a             MLETVAAVPGMVGGMLLHCKSLRRFEQSGGWIKALLEEAENERMHLMTFMEVAKPKWYER 240
ne.TaAOX1d-2BL.2    LLETVAAVPPMVGGVLLHLRSLRRFEHSGGWIRALMEEAENERMHLMTFMEVTQPLWWER 215
                    :******** ****:*** :******:*****:**:****************::* *:**

AtAOX1a             ALVITVQGVFFNAYFLGYLISPKFAHRMVGYLEEEAIHSYTEFLKELDKGNIENVPAPAI 300
ne.TaAOX1d-2BL.2    ALVLATQGVFFNAYFVGYLISPKFAHRFVGYLEEEAVHSYTEYLKDLEAGLIENTPAPAI 275
                    ***::.*********:***********:********:*****:**:*: * ***.*****

AtAOX1a             AIDYWRLPADATLRDVVMVVRADEAHHRDVNHFASDIHYQGRELKEAPAPIGYH-      354
ne.TaAOX1d-2BL.2    AIDYWRLPADARLKDVVIAVRADEAHHRDANHYASDIHYQGMTLNQTPAPLGYH*      329
                    *********** *:***:.**********.**:********  *:::***:***
```

## ne.TaAOX1d-2DL:

```
AtAOX1a             MMITRGGAKAAKSLLVAAGPRLFSTVRTVSSHEALSASHILKPGVTSAWIWTRAPTIGGM 60
ne.TaAOX1d-2DL      ----MSSRMAGATLLRHLGPHLFAAAEPASGLAAS-ARGIL-PA---------AARIFPA 45
                         ..  *. :**   **:**::.. .*.  *  *  ** *.         *  *
```

```
AtAOX1a             RFASTITLGEKTPMKEEDANQKKTENESTGGDAAGGNNKGDKGIASYWGVEPNKITKEDG 120
ne.TaAOX1d-2DL      RMASTA------------AGAHAKQEGDAEKPESATAPEQNKKPVASYWGIEPRKLVKDDG 94
                    *:***             . : * *.::   ::* . ::..* :*****:**.*:.*:**

AtAOX1a             SEWKWNCFRPWETYKADITIDLKKHHVPTTFLDRIAYWTVKSLRWPTDLFFQRRYGCRAM 180
ne.TaAOX1d-2DL      TEWPWFSFRPWDTYRPDTSIDVAKHHEPRAVADKVAYLIVRTLRAGSDLFFQRRHASHAL 154
                    :** * .****:**: * :**: *** * :. *::**  *::**  :*******:..:*:

AtAOX1a             MLETVAAVPGMVGGMLLHCKSLRRFEQSGGWIKALLEEAENERMHLMTFMEVAKPKWYER 240
ne.TaAOX1d-2DL      LLETVAAVPPMVGGVLLHLRSLRRFEHSGGWIRALMEEAENERMHLMTFMEVTQPLWWER 214
                    :******** ****:*** :******:*****:**:****************::* *:**

AtAOX1a             ALVITVQGVFFNAYFLGYLISPKFAHRMVGYLEEEAIHSYTEFLKELDKGNIENVPAPAI 300
ne.TaAOX1d-2DL      ALVLATQGVFFNAYFVGYLISPKFAHRFVGYLEEEAVHSYTEYLKDLEAGLIENTPAPAI 274
                    ***::.*********:***********:********:*****:**:*: * ***.*****

AtAOX1a             AIDYWRLPADATLRDVVMVVRADEAHHRDVNHFASDIHYQGRELKEAPAPIGYH-      354
ne.TaAOX1d-2DL      AIDYWRLPADARLKDVVIAVRADEAHHRDANHYASDIHYQGMTLNQTPAPLGYH*      328
                    *********** *:***:.**********.**:********  *:::***:***
```

ne.AesAOX1d:

```
AtAOX1a             MMITRGGAKAAKSLLVAAGPRLFSTVRTVSSHEALSASHILKPGVTSAWIWTRAPTIGGM 60
ne.AesAOX1d         ----MSSRMAGATLLRHLGPRLFAAAEPASGLAASA-RGIM-P---------AAARIFPA 45
                         ..  *. :**   *****::.. .*.  * :   *: *          *  *

AtAOX1a             RFASTITLGEKTPMKEEDANQKKTENESTGGDAAGGNNKGDKGIASYWGVEPNKITKEDG 120
ne.AesAOX1d         RMASTEAAGPH---AK------QESDAEKPESAATPEQQNKKPVVSYWGIEPRKLVKEDG 96
                    *:*** : * :    :      : .: ..  .**  ::...* :.****:**.*:.****

AtAOX1a             SEWKWNCFRPWETYKADITIDLKKHHVPTTFLDRIAYWTVKSLRWPTDLFFQRRYGCRAM 180
ne.AesAOX1d         TEWPWFCFRPWDTYRPDTSIDVTKHHEPKALADKVAYFVVRSLRVPRDLFFQRRHASHAL 156
                    :** * *****:**: * :**:.*** *.:: *::**:.*:*** * *******:..:*:

AtAOX1a             MLETVAAVPGMVGGMLLHCKSLRRFEQSGGWIKALLEEAENERMHLMTFMEVAKPKWYER 240
ne.AesAOX1d         LLETVAAVPPMVGGVLLHLRSLRRFEHSGGWIRALMEEAENERMHLMTFMEVTQPRWWER 216
                    :******** ****:*** :******:*****:**:****************::*:*:**

AtAOX1a             ALVITVQGVFFNAYFLGYLISPKFAHRMVGYLEEEAIHSYTEFLKELDKGNIENVPAPAI 300
ne.AesAOX1d         ALVLAAQGVFFNAYFVGYLISPKFAHRFVGYLEEEAVESYTEYLKDLEAGLIENTPAPAI 276
                    ***::.*********:***********:********:.****:**:*: * ***.*****

AtAOX1a             AIDYWRLPADATLRDVVMVVRADEAHHRDVNHFASDIHYQGRELKEAPAPIGYH-      354
ne.AesAOX1d         AIDYWRLPADARLKDVVTAVRADEAHHRDANHYASDIHYQGMTLNQTPAPLGYH*      330
                    *********** *:*** .**********.**:********  *:::***:***
```

**Color Key:**

| Pos | Consensus | | Pos | Consensus | |
|---|---|---|---|---|---|
| | **AOX1a-c/1e** | **AOX2a-c** | | **AOX1a-c/1e** | **AOX1d Consensus (Monocots)** |
| 112 | Pqasgt | Rknt | 167 | T | KMSr |
| 124 | Krp | Pta | 175 | Yf | H |
| 229 | F | M | 178 | R | H |
| 233 | Ast | V | 180 | Mi | Lv |
| 241 | Agv | Lfmiv | 181 | Mv | L |
| | | | 295 | Vcst | Tas |
