# Supplementary material for: Genome-wide identification and analysis of the ALTERNATIVE OXIDASE gene family in diploid and hexaploid wheat
Source: PLoS One. 2018 Aug 3;13(8):e0201439. doi: 10.1371/journal.pone.0201439 (PMC6075773; doi:10.1371/journal.pone.0201439)
Supplement: S4 Fig — (PDF) [file pone.0201439.s004.pdf]

S4 Fig. Nucleotide sequences of *AOX* from hexaploid and diploid wheat.

PROMOTERS

TaAOX:

>*TaAOX1a-2AL* promoter

CGTTGTCGTCGTCGTCTTGAAGGTAGGTTGGTCGCTCGCCGGAGAGGAGACCAGA
GCGTGATGTGATGGGTTGGCATTGGTTGAGTTTGAGATGGAAACCTCGAAGCGGG
GCGCCGATGATCAAATAGTCACAATTTTGTAATCACGGGATTTGTACCGTGCATCT
CAAATTTCACAATCAAACAAAATCCAAAATTTCACTATGCATTTTCCATTTTGCCAAT
CACAAACTGGCTAGCATGGTCGAACGAGTCAAACCACGGTGAAAATAACCGAGAG
AACAAAAAGGAAGCAAGAGCCAACACATCCAACTGATGGCACAAGTGTGTTACAAT
CTTAGAAGAAACACAAGCATGGCAAGCACAAGGCCACCAAAGATGATTGCTATTCC
CAAGCAAAGCAAGATCCCAGATGCTTCTAGCTGCTACAGTAGTTAGCTCACGCAG
GACAGGAGGGCCGTATTTGGTCACGTTCCGGTGCTGTGTTAGCTGTTAGAGATTAT
ATGGATCTATTAATAGCGGTTGCCCCGTTTGACGGGTTCTTTCTTGGTCCACAAGT
AAATATTGGGATGGATGGATGGGCATCTGCCGGAACACATCTCTCATTTCGCTTTG
TTTCCTTTTCGCCAGATATTTGCCAACCGTGATAAAGATAAACGGCTGCACGTACC
CGCCAAGTGTGAAAGGGTGCTAAACAGAATATTCCTCTCCTACGAGAACTACTTCA
GAACCTGATTGTACAGCCAGGCTTCGATGATCCCTTTCCCGGTTTAAAAAAATAAC
AATTAGTTTTTTCAAAAAAAATCGTCGTTCATTGTGCATGACACTGTCTCACATATTA
TATTCTACCACTAACCAAACTCCATGAGTGGAGGCACACCGTCACAAATTGAATAC
GTTCACTTTTGCAAGCTTAAAGTAAAGTAATCCCTTAGCAGCCACTGGCCTAAAAAA
ATACGTACAATATAAAAAAATGCCCGCAAAATAAACAAACTTTAAAATTAAATTAAAT
TGAAGTTAAAACGAGTGCTTACATGGCTAACCACGAGGCGTAATTCGTGTCCCGCC
TAGGCATCACACGCACGCCAGTCCGGTCGCAAAGCCGCGAACACGTGAGCCTCT
CGTTGGATGCCAGTGGTCACAGGCCGTCTCCACGCGGAAGGGGGGAAAAAGGGG
GGGAAGAAAATCGCAACGACCAAAATTAATGCGATCGATCGACTTGGAGGAGAAG
AAAAGGCCTCCTCAGTTTCTGGGATCCCCGGGAGGCAGGCACGGCCCCCCCGCC
ACTGACGCCGCGGCCCCACCCCTGCCCTCGCCGCCTCCGCCATCGCTCCTCCTT
CCCGATATAAAATCCCTCGGCCAAAAGACCCTTCCATTCGCAACCCGCAACCCCC
CAACCCCACCCCAACCACCCACCGCAGCAACAAGGGCAAGCGTGCGTCAGCATC
AAACGACGGCCCGTCCCGGATTGTGATTCGCGGAGGCGTTCCTCGGCGCCCAG

>*TaAOX1a-2BL* promoter

TATGTCACATCTAGATGTGTCCTAGACAGACCCATCCAACAAAGAGCGCGCAGTGA
TCTGATGCAGCCGATGTTCGCCAAGGAGAGACAACACATCAGACAAATGCATGTG
AGTGGTTTCCCAAGAGGATATGTAGAGAATACGATCGTTCCGGACGAGCGTGGGA
CGATCGCGCTTGTTCAACCAAGTATAGCGGCGCCCATGAAGACAGAGGTCACGAA
GTTCGACCAAGTATAGCGGCGCTCATGAAGACAGAGGTCACGAAGTTCCTCATCC
ATGATGAATCGCCATAAGCAATGCCTGGCACAACAATTCAGGCAGTGGTTATTCTT
ACCAACGATAGATGTAATCATGTTGAAGTCACCCCCAAGAGCCAAGGCCCAGCAC
ATGAAGCGCGCACGTCCTGAAGGTCACCGAGGAGGGCCCCTTTCTCGTCGTCGTC
ATCCTGAAGGTTGTACATCCCAGTGATCCACCAAGGTTGGTCGCCCGCTGGAAAT
GAGACCAGAGCAGTGATGTGATTGTTGCCATTGTTGAGTTTGAGATGAAAACCTGA

AGCGGCGCCGGTGATCAAATTGTCACAACCACGCATATCAAATTTCATGACCAAAC
AAAAAACCAGAGTTGTACTATGGGATTTCCGTTTGCGAAACGCAAATTGGACGCCA
TGGGTAACCACGGTGAAAATAACCGAGAGAACAGAAAGGAAGCAAGGGCCAAGAC
ACCCAATTGCCAATTGATGGTACAAGTGTGATACAATCCTAGAAGAAACACAAGCG
TGGCAAGCACAAGGCCATCAAAGATTATTGCTAGTCCTACAAGCAAAGCAAGATCC
CAGATGCTTCTAGCTGCTACAGTAGTTAGTTCACGCAGGACAGGAGGTCCGTATTT
GGTCACGTTCCGGTGCTGTTGGATTAAGGATCTATTAATAGCAAGTTGCCCCGTTT
GACGGGTTCTTTCTTGGTCCACAAGCAAATATTAGGATGGATGGATGGATGGGCAT
CTGCCGGAAGACATCTCCCATTTCGCTTTGTTTCCTTTCCGCCAGATATTTGCCAAC
CGTAACAAAGATATAAACTGCACGTACCCGCCTAGTGTGAAAGGGTGCCAAACAG
AATATTCCCCTTCTACGATAACTACTCCTACTTCAGAACCTGATTGTACAGCTAGGC
TTCGATGATCCTTTTCCCGGTTTAAAAAAATCATTTGTTTTTTCGAACAAATTGTCGT
TCATTGTACAGGACATTGTCTCAAATATTATATTCTACTACTAACCAAACTCCATGAT
TGGAGGCACACTGTCACAAATTGAATACGTTCACTTTTGCAAACATAAAGTAAAGTA
ATCTCTTAGCAGCCACTGGCCTAAAAAATACGAACAATATTTTAAAATATCCCCAGA
AAAAAATCAAAATTAAATTAAATCAACTTAAAACGAGTGCTTATATACATGGCTAATA
AAACGCCGGTCGCAAAGCTGCAAACACGTGAGCCTTTCGTTGG
>*TaAOX1a-2DL* promoter
GACCAGAGCGTGATGTGATGGGTTGGCATTGGTTGAGTTTGAGATGGAAACCTCG
AAGGGGGGCGCCGATGATTAAATAGTCACAATTTTGTAATCACGGGATTTGTACCG
CGCATCTCAAATTTCACAACCAAACAAAATCCAAAATTTCACTATGCATTTTTCATTT
TGCGAATCACAAACTGGCTAGCATGGTGGAATGAGTCAGACCACGGTGAAAATAA
CCGAGAGAACAGTTTCAAAAAAAAAAAAAAATAAGCGAGAGAACAAAAAGGAAGC
AAGAGCCAGCACATCCAACTGATGGCACAAGTGTGATACAATCCTAGAAGAAACAC
AAGCGTGGCAAGCACAAGGCCACCAAAAGATGATTGCTAGTCCCAAGCAAAGCAA
GATCCCAGATGCTTCTAGCTGCTACAGTAGTTAGCTCACGCAGGACAGGAGGGCC
GTATTTGGTCACGTTCCGGTGCTGTTTAGCTGTTGGATTAAGGGAAGGATCTATTG
ATAGCGGTTGCCTCGTTTGACGGGTTCTTTCTTGGTCCACAAGCAAATATTAGGAC
GGATGGATGGATGGGCATCTGCCGGAACACATCTCCCATTTCGCTTTGTTTCCTTT
CCGCCAGATATNNNNNNNNNNNNNNNNNNNNNNNNNNNNNNNNNNNNNNNNNNNNNNNNN
NNNNNNNNNNNNNNNNNNNNNNNNNNNNNNNNNNNNNNNNNNNNNNNNNNNNGAGAACTA
CTTCAGAACCTGATTGTACAGCTCGGCTTCGATGATCCTTTTCCCGGTTTTTAAAAA
AATCATTTGGTTTTTCGAAACAATCGTCGTTCATTGTACAGGACACTGTCTCAAATA
TTATATTCTACGTACTAACCAAACTCCATGATTGGACGCACAATGTCTCAACTTGAA
TACGTTCACTTTTTGCAAGCTTAAAGTTAAAGTAATCCCTTAGCAGCCACGGGCCTA
AAAAAAATACTTACAATACTAAAAAAAATCCCAGCAAAAAAGCAAAATTCGGAATTAA
ATCAAACGAAGTTAAAACGAGTGCTTACATGGCTAACACCGAGACGTAATTCGTGT
CCCGCCTGAGCATCACACGCCGGTCCGGTCGCAAAGCTGCAAACACGTGAGCCTT
TCGTTGGATGCCAGTAGTCACAGGCCGTTTCCACGCGGAAGGAACAAAAAAAAAG
CAAGAAGAAAATCGCAACGACCAAAATTAATGCGATCGATCGACTTGGAGGAGAA
GAAAAGACCCCCTCAGTTCCTGGGATCCCCGGGAGGCAGCGGCTGGCTCCCCCG
CCACTGACGCCGCGGCCCCACCCCTGCCCTCGCCGCCTCCGCCATTGATCCCCC
TTCCCATATAAAATCCCTGCAACGAATGACCCTTCCATTAGCAAACCGCAACCCCC

ACCCCACCCCACCCCACCCCACCCACCACAGCAACGAGGGCAAGCGTGCGTCAA
CATCAGACGACTCGTCCCGGATTGTGATTCGCGGAGGCGTTTCCCGGCGCCCAG
>*TaAOX1a-like-2DL* promoter
AGAGTCCCGGCCGGCTTATAAACAGAATCCGGCTCGGTAACTAGTTAATCTTGCCT
TACAACACAAGTCACGCCATTACGGCGGTTTATCACTACGGGCCTTAAATCACCTA
CGGGCTTTAGGCCCTTTATTGAACCGCCATCTTCAAGCTTGATATTGGGCTTCATAT
GATGAATTGCCATAACGTAACCCGGCCCCTCCTGGGCGGGTTACGCCAGTAGTTA
TATCCCCAACACATCCCGTGCGAGTGCAGCGGAGGGCTGAAAGGGTGGCCCGTT
TTGTTGCCTCCTATCATGCCATCCGACGTCCGGCAGCGCATAATGAACTCAAGAAG
GATCTCATTGAGGAGTGGTGGGCTTGGAATGGCCGGCAAAGAGCATCATGATTTG
TGAGTTTGATGTTGTATTGTTGAACTATTTGTTGTATTTCATGAAAGATACTATTTGT
TTGAGTTGTAATAAATTGAACTATTCATTTTAAACTTGGTTGAATGATGTTTGTGATT
TAAAAATTATATGCCATGTCTTTGTTTTGTGGATGTGTTGTGAGACAAAATGCAACA
AATGTGATGCGCCGGCTGCTCACGCGCGTTGCATTTTAGCGCGGCTGCTGGAGCC
AGCGCTGCGCGCCGCGCCAAATCTGGCGATGGACGCGCCGCAAACCAGATTTTA
GCGCGCCGCGCGTTGACCGCCTGTTGGAGATGCTCTAATACTTACACGCCACCCA
GTGTGTGTCAGATACTTTAGTGATAACAGTACCATGCACCAGGTCCTTTGCGTCGG
ATGTGTACCCCGACGAGCCCACCAAACCGACGTGGACACGGGTATTTCGCAAGTA
TGAATCGATGTATGTCACCTTTTCTCGTTTGAACAAAGATTAATGCCCGGATCACAT
CGCCTGGAGCAGCGGCTGGATATCTGCGTCCACGACGTCCGCTCTCGTAAGTCAC
GATGAGACTTAGCTTCAAAGTCACAGAAGCTGAGTCCAACATACACCCGCAAGCC
GGCGACGACGTTCCTTGTCATGAGCTTTCGTCTAGAAAGACTTATCTAGCTTGTTA
ACCAGACAGACACTTGCACGGCAAGGAATTAGTTAGATTATTAAGCACCGGCGCG
CCGCCGCGCGACGGCTGTGCTGCACCTGCACTGTCCAAGAAGCGCCGCCACCCC
ACATGCCAACCAAGAAAGTGGCGCTGAAAAGCCCGAGAGCGCCTCGACGCCGGA
GCAGAGCAAGAGGGCCGTGGTGAGCTACTGGGGCATCGAACCGCGGAAGCTCGT
CAAGGACGACGGCACGGAGTGGCCGTGGTTCTGCTTCAGGCCGTGGGACACGTA
CAGGCCGGACACGTCCATCGACGTCACCAAGCACCACGAGCCCAAGGCCCTGGC
GGACAAGGTGGCCTACTTCGTGGTTCGGTCGCTGCGTGTGCCCAGGGACCTCTTC
TTCCAGCGCCGGCACGCCAGCCACGCCCTGCTGCTGGAGACGGTCGCGGCGGTG
CCGCCC
>*regTaAOX-4BL* promoter
ATGCTATTCTTTTAAAACCCTGTCTGTGATGTCACCCAGATGTCCGACTGCGGTATT
TCTTCTAAAGCCCTGTCTGTGATGTCGCCCAGATGTCCGACTGTGGTATTTCTTTTA
AATCCCTGTCTGTGACGTCGCCCAGATGTCCGACTGTGGTATTTCTTTTAAAGCCC
TGTCTGTGATGTCGCCTAGATGTTCGACTGTGGTATTTCTTTTAAAGCCCTGTCTGT
GATGTCGCCCAGACANNNNNNNNNNNNNNNNNNNNNNNNNNNNNNNNNNNNNNNN
NNNNNNNNNNNNNNNNNNNNNNNNNNNNNNNNNNNNNNNNNNNNNNNNNNNNNNNN
NNNNNNNTTTATTCATGACGCATTCATGCATTCATTGATTATATCTTTTGTCCGATCT
GTCTTGCGAGTACTTTCAAGTACTCACCTGGCTTGTTGATTTGGCCAGATGCTGAT
GAAGGCGATCTCATGGATGAAGAGTTTGATAGCGAGTCCGACGCCTAGAGGAGTC
CCAGTCAGTCCCGTGCGATCCTGTCTTGGTCATTGTACTATCCGCTTCCGCAACCC
CGAATAAATTCATCGAGCCTCACCTCAACGCTCGATGTACTGCCAGTAGAAGTCAA

GTTATCCACCACCACTTTTCCCTCGAGCTAGTAGCATGCCACCCAACCCCGGTGTC
ATATCCGCCCATATGTAATATATTGGCGAGTCTGTAATAAAATTGTTGAGCAACCTC
AGCTCAACCCTGTAATATATTTGATGCTACTGGTTCTCTGTTTATCAAGCTTTTGTCT
ACCAGAAAGGATGACTTTTCCTATACTGGGATTTAAAGATTGGTTTTCTCAATAAAT
TTTTTTATTGAAAAACCGGTCGTGACACGCCGCTGCCTATCTCTCCCACCCACCCC
GATCCATCCCCTCCCTTTGTCTCTCACATCACCTCCGCACCCATCCCCTCCGCTGG
CGGGATTCGCCGCTCCCCCTTCCTCCATCTCGCTCACACTCACAGCCACCGCCGC
CGCCGCAACCCAAACCCTCTCCCTCCCACCCTGATCCGCTGTCACCCCCACCTTC
TACGACCTAGATCGAGCAAGCGAGGCGGTGCTTCCAGACCCGCGACGGCGGCAG
AGGGACGGGGCCGGGGCGTGTGCCAGATCTGCGCCGACGGCCTGGGCACCACG
CTCGAAGGCAAGGTCTTCAGCGCAACCCCCCAACCCCCCCCCCCCCCCCCCAC
CCACCACAGCAACAAGGGCAAGCGTGCGTCAACATCAGACGTCTCGTCCCGGATT
GTGATTCACGGAGGCGTTTCTCGGCGGCCACATGAGCTCCGGGATGGCCGGATC
GGTCCTCCTCCGCCGCGCCGGCGCTGGCGCCAACCGCCTCTTCGCCACCACCGC
GACGTCCCCGGGGGCCAAGACCTCCCTTGTCGGCGGCAAGGGCGCGTTGCTGCG
GATGATGTCCACTTCCGCGGCCTCGTAGGTGGTCAAGGCGGAGGCGGCCAAGGG
CG

>*TaAOX1c-6AL* promoter

CATACTCGAAGCCAAAACGACCGGATGTGTGCGAGAAAGACGCAAAAGAAAAGAA
GAAGAGGAAAAGAGATGATGAGCTAAAAAATGCTATGAAAACTATTGTGAAGGGAA
GAAAAGAAGCGAACGAGGTGAGGAAGATGGCAAGGAACCAAGATGCCGCGGCCG
AGGAGAGGAAGGTGGCATTGGAGGAGAGGAAGGTGGGCATGGAGGAGCGATCTA
GGTTGTTGTGTTGGATTGGAAGAAGTACTTGTTCTTCATGGACACATCTATCCTCAA
TGAGGCGCAAAAGGAGTATGTCAATCTTGCCCATGAAGAAGTCCTGATCGAAAAAA
GAGCCATGATTCGCGGCATGGGTGGTGGTGGCCTTGGCGGCATGGGTGGCCTTG
GCGGCATGTGAGGCATCGGTGGCTTCGGAGCTACCATGGGTGGCTTTGGAGCTAT
CATGGAGACCATGGGAGGCATGGGTGGCTTCGGAGCACCTCCGGGCGGCATGGA
CTGTATGGGAGGCATGAGTTTTGCGTCTCTCATGGGAGGCATGGGAGCACATCGG
GCGACATGGCGGAATGTCTTCCGAGGTGCCTCACACACCTTCGCATGAAGATGCC
GTTGAAAATCTAGCCAACACCTTCCGAGCTTCACATGATGATGCGGCGTGCGACAA
TGAAGAGGAGGAGGAACAATCGTCTTCGAAGGAGGAGGATGAGTCGGAGGAAGA
TGAGGATGAAGACGAGGACGAGGCTTAATTATTGATGTGCCTTTCGTTGATGTGAA
TTTTTGTGTCATGAACTTGTTTTGGATTTTAAACTTGGCCAGATGATGTTATGGGCA
TGAATTTGAACTTGTGGGCATGAACTTTTATGTCAGCATGAACTTGTTTGTGTGATT
TGAATTATGCCATGTCATTGTTTTGATGTTTGAAATTCATTTTGTGTCCAAAATGCAA
CATATGGCAAGCGTCGGCTGCTCCCGCGCGCATGCGGGAGCTGCGCGCGCTAAA
TTTTATCACGGCCGCTGGAGCAAGCGCTCCTCGCCGTGCCAAAGCAGACGATCGA
CACACTGCAAACTGATTTTTAACGTGTCATGGTATTGGGCGGCTGTTGGAGATGCT
CTAAAAACGAGGGGAAAAACAGTAGGCCAGTCTAATTTTCGTGTCAAAACAAGTCC
ACTAAAACTAACAATCTTTCCTCATCCGTTGAAATGGGAACACAAAGATTCTAGGCA
CGCCGACGCACGGCCAAGTTCAGAGTCCACCGGAAGTCCTAGCAGCCACCCCAC
CACATAGCAGCCGCAACGATTTTGACACCCAATCCTGCCTCCTGAAAAGCTTTTTTT
TTCTTTCTTTGAGGGGAGCCTCCTGAAAAGCTGGGGCACACCCGAAACTACAAATC

TCACGGGCGAGAGCACGCAGAAGCTGCCCACCAGTCAATTGCGAGCTCCAGAAA
CACCAGTCAAGCAGAGCCGCCGTTGCTCCCACGTCTCGCGTCATCTGCTCGTAGC
GCC
>*TaAOX1c-6BL* promoter
AAAAGGTAAGGTCCCCAATTCGCACCCAAACAATTGCCTATAAGCCTCTTGTTCCT
CATTGGCTCCTCCAAAACAGAACAATTCGCTTTTGTGAAAGTTAATCTTTAACCCGG
TCAATTGTTCAAATAAACATAACACCAGCTTCATATTTCTCGCTTTTGCCAAGTCATG
CTCCATAAAGATGATCATATCATCGGCGTACTGTAGGATAGACACACCTCCATCAA
CTAGATGAGGCACCAAGCCACCCACCTGACCAGCCTCCTTAGCCCTTTCTATTAGA
ATTGCCAACATATCAACTACAATGTTGAACAAAATAGGAGACATCGGATCCCCTTGT
CTCAGGCCCTTATGTGTCTGAAAATAATGACCTATGTCGTCATTCATTTTAATTCCA
ACACTCCCTTTTTGCATGAACGTACCTGGCGTCGCCAGGCCTCATCAAACCTTTGA
CTTTATCGCACGCTTTCTCAAAAATCACCTTAAAAACAACTCCATCTAGTTTTTTCAT
GTGGATTTCATGGAGCGTTTCATGAAGGACCACAACCCCTTCTAGGATGTTTCTGT
CCGGCATGAAACCAGTTTGGAAATGCTGCACCACAGAATGCGCAATCTGCGTGAG
CCTATTAGTCCCAACCTTGATGAAAATTTTGAAACTAACATTGAGAAGACAGATCGG
CCTGAACTGCTCAATTCTCACAGCCTCTGTTTTCTTAGGAAGCAATGTTACTGTTTC
AAAATTCAAGTGAAATAACTGAACCTGTCCAGAGAATAAATCTTGGAACAATGGTAG
CAAATCCCCCTTAATAATATGCCAACACTTTTTATAGAACTCCGCCGGAAATCCATC
AGCTTTACTATAATCGATAGGGTGGCATTCTTTTCTCCCTATTTTCTCTCGATGTTTG
TGTCGAGACAATTTAGATGCCAAGGCGCTTGAATGTCTAATGTCTATTTTTAATTTC
TTCATTTTTTGAAGCTGATGATGACTCCAATTGTACAAATTGTTTATTCAGTCTTCCT
TTCCTTAACAACACTAAATAAAAAGGAGATACAAAAATAATTTTTAATGTGTATATTT
CCATAATGGCAGGAGTAATACGAGTTTTTTTGCTTTGTTCAAACTCTCGACAAAATT
GCTTCATAACTATAATGAACGAGGAATTTTCTTCCCGCCGTACACGGGTACGGTTG
AAACTCACCTGGTTGATTGCCTTTGATGTGTGATATACTCATATGGATTTATTTTTAA
AAAGAAGTTTATCTACTAATGATGAAGGGAGAAATTCCTTTGTAAGGTTATTCATGC
ATTGCACGTGCATGCTTGCTAGTCAGTTGAAATGGGAACACAAAGATTCTAGGCAC
GTAGACGCACGGCCAAGTTCAGAGTCCACTGGAAGTCCTAGCAGCCACCCCCCAC
CACATAGCAGCCGCAACGATTTTGACACCCAATCCTGCTGCCTCCTGAAAAGCTG
GGGCACATCCGAAACTACAAATCTC
>*TaAOX1c-6DL* promoter
CTGAAATGCAGAAGAAACCCAAATATATGATGTTGGTGAATAAGACCACCAGTTTC
AAAAAGAGAAGCAAAGCAAAGAAGGCCAAGGGAGCTAGTAAGACCGGCGCCCAG
GAAAAGCCTAATGGTGATGCCACTAAGAACATTTGATGAGGTTATCTCGGTACGCC
CATGAAGACTCAAGCCCACATACTATGTATACCAAGATAACATCATCAATGAAAGA
GTTTTTGTCACTTGACATAGGTCACTCATGTGAAGAATTCCGTCCCTCGGCTCATAA
TGAAAAGGATATACCTTCACTTGGATAGACTTTAGGTGCTCACAAGCGCAACGGTC
AGAAGCCTGCAGTTACAACTAGCAATTAGAGTGTTTGTTGCTGTCAGGCGAGGACA
TGTTAGGGCATATACAATGATGCTATTTTAGGAGTGCCACATAAGATAAATAATGAG
GTGGAGAAAAGACAACTCATAAGAAAAGGCTTGCCTTCTCTTATTTAAGAGAAAAC
AAGAGGTGATCTCTTAGCACAATATGTCTCACCATATTTTTAGGAATAACTAGTTAT
TGAAGATAACGCTAAGAGATGACCCATTGTAGAAAAAAAAATTGTCATCTCTAAATT

ACATTCAAGACTTAAGATAAGACTATCTTATCAACCATTGTACATGCCCTTAATACC
TCTTTATCGGTCCGTGGACGGATGAGGCGCCGTGTTAGATGCCAAAACATGTGTG
GATGGATGCGGGCAGTTTGAAAGTTAGCGTTCGAAATGCCTAAGAGCTATTTGACG
CAGCACCCTTGCCATCAAGCGAACGAAAACTATCTGGTTTGCATGGATGATTCTAG
ACTCACGGGCTGCCTCCAACGGACCTTCGCGGAGTGGCCAACATGCCATCTCCTA
ATTCGCCCCCCCCCCCCCCCCATTTTCACTGTAGGGCCCGCCAACCTTAATTAAAC
CCTTGCCGCTAATACTCCCTCCGTTCCTTTATATAAGGTGTATTTATTTTTTTATAAA
ATTTCAGAATGTAAGATGCGTTTTTTTCTAATTCCTCGTGAATACCCCGTGAGTGTA
CCATTGCCCAGGTTTGCATCTTCTGATTCGCCACACGAAGAGTCTTCATTTCTCGG
GTCTTTCTCTAAGCAAAAACGAGGGGAAAAGCAGTAGGCCACTCCAATTTTTCGTG
CCAAAACAAGTCCACTAAAACTAACAATCTTTCCTTATCCGTTGAAATGGGAACACA
AAGATTCTAGACACGTCCACGCACAGCCAAGTTCAGAGTCCACTGGAAGTCCTAG
CAGCCACCCCACCACATAGCAGCCCCAACGATTTTGACACCCAATCCTGCTGCCT
CCTGAAAAGCCGGGGCACATACGAAACTACAAATCTCACGGGCGAGCGCACGCA
GAAGCTGTCCACCAGTCAATAGCGAGCTCCAGAAATATCAGTCAAGCAGAGCCGC
CATTCCTCTTCCTCCCACGTCTCGCGTCGTCTGCTCGTAGCGCC
>*regTaAOX-3B* promoter
TAGGATCTATTTCTAACATCTTCGTTGTGATGAGCACAGTTTTGTGTCCTAAATAAAT
GTATTTTCATTTGGTTATTATATACTGACAAGTCAGAGCTGTGTTCCCTGAATCCGT
GGGCGCTTGGCAGCCGACGATCTGGAGCGTCCACTGAGTTCAGCTCACCTCGTCA
AACGACCAAACATGCAAGCCGACCTGTTCAGCCCGTGTTTTTTTTGTTTCTTCTTCA
GTTTTTCTTGTTCAATAATGCCTTTGTGACGTTTTATGTTTTTTTTCCTTTTCCTTTTC
AGTTTCTGCAGATCCTCTATCTTTTTTTCTTTGCTATTTTTCTTCCATTATTCTTATTC
TTATTTGTTTTTTTTATCCTTTTGTGTTTCTCTCTTTAAGTATTTTTTCCTTTGATTTAT
AAAAAGTTGGCTAAACTTTTTTTAGCATATGAATATAAATTTTTGCATGCATGAAATA
TTTCTAAATATGTGTTTAAACATTCTATTTCACATACTTAATCATTTTTCTAAATTATTA
ATGTTTCTAAAATTTCATAAAAATTTATACATGATATAGAATATGCGTGATATCTTAA
CGTAACATGATTTTTCTAAAAACTAATATGTAATTGTTTTCTATTGAATCTAATTTTTT
TTATACATGAATAATTTGGTGCTATGTAATAACATTTTATTCATATATATGAACATTTT
TTAGCCGATGATTTTGTTGGAATTTTCTAACTAATTTAATACAAGAATATTATTGTTT
GTATACTTTTTAAAGTATGAAATGAATAATTTTAATCATACTCCCTCCGAAAGAAATA
TAAGAGCATTTAGATCTCCACTTTAGTGATCTAAACGCTCATATATTTCTTTACGGA
GGGAGACATATTAACATTTTTATCTGAATATGTGAACATTTTTATTTTACGCAATATA
ATTTGCCCCATTTTTGAAACTGGTTTTTTGACAACTTTATTTAAATGATTATTTTTCCT
TTTTGCAAATATATAGTACTTCGATTATTTAATCTTTTATTAATATATTTTTCATAAATT
TGTAACTATAAATAATTAAAATTAAGTGCTAAGTGCGTTGGTCTTGTGATGCGTGGA
GTGGGTTTGACCACCTATTTGATGCAACTCTCCGCCGACCAACCTGAATTGTCATA
TCAAATTTGAAGTGAGACGATTAGTACTCATGTTTGTTATGCTACTTGCAGTGGATC
AATGCAAAAAATAGAAATTATGTTCAATTGAAACAATTTGTGTACATTTTTCATCGAC
GAGGACTAGGCTGTAGCCTAGTGGCAAGGGGCGCGGTGGCAAACCCTGCGGCCA
CGGTTCGACTCCTGACGGGAGCGAATTTGTGGCGCCTCACCCGGGTGTGCTTTTC
CTATAAAAATATGTCCAGATCCAGGGTGCTAGTGCCCATGGATAGTCGCTCCGGC

GCTTCGAGCAGAGCGGCGGGTGGATCCGTCCACTGCTAGAGGAGGCCGAGAACG
AGCGC
>*TaAOX1d-2AL.1* promoter
TCTTTTGTTGCTCAGCTGTGAGCGGTTTGGTGCTTGGTGGGGGCTGTAGGCCCTG
AAACTTAGTAACTGAGATATGGTGTCGTCGGGTTTTCGTCCCACGGTGGGTAATCT
CGATGGCGAGTGCTCACAGTGGGTTTCCCGGCGATGCTTGAACTCGCTTTCCCCT
TTTCTTCCTTTCTTTGGACTACTGTATTTCCATTATGTCGCAGTAATGAAAGGGGTT
ATGCCTGGTTTATATTTTACAATGATAGTTACTACGATTAGAAGAATGCGAGGGATT
ACTATTCTACTCCCTCCGTTCCTAAATACTTGTCTTTCTAGAGATTTCAACAAGTGG
CTACGTACGGAGCAAAGTAAGTGAATCTAAACTCTAAAATATGTCTATGTACATCCG
TATATTGTAGTCCATTTAAAATCTTTAGAAAGACAAATATTTAGAAAAGGAGGGAAT
AGCTATTTTACGAGGAATATAATAAAGCCTACTTTGCGGAGCAGAGTGTTGTGCGT
GACTAGCAGTAACCATGTTAATTCCATTACTTTTTCTTGCATTCGGTTTTACCATGAT
GATTAGCGGGTTACACGGAGCTGCGGGACGGAGGTGAGGCGCATGAAGTGGTAA
CAGAAATTAAGTGTATTACCATATTTTCTACTTCTGTTTACTCTGTTAGCAGCCCCTC
ATGCAAAAAATTAAGGAGTATAGTATGTGTAACAACATAAAGGAATATTTTTATTTTG
AACTGAACCACAAAAGAATTGGATCATCCATGACAGTTAGCTGTTGGGCGAGTGCG
CACATGTTTTTTGTTTCTTCTTCTCTATCCAGGGAGAGTGCGCACATGTTGTCTAGT
GACATTTCCGGGAAGCACCGTGCCGTGCCGGCCGAGACACTCCCGACGCGTGGC
CCATCTATAAAACCATCTCCAGCAAGACGTTTCCTGCCATCAATCAGTTTGCCAGAT
CAACACCAACAGAAATCACATCCCAGCCACCGGGACCTTTTTTTTACACAGTACAG
ACGCAAGCGCTCATATAAACGCGTATACACTCACCCTTATAAACGCACACACGTAC
ACTCTATCCCTATGAGTACCTCCGAGAGACTGAGCTGGCATATCATGTTGAGATTT
TACGAAGTCACCGTAGGCGCCTCGTAGTCGACGGGAACGTCTCCTCCCACTGAAA
GCGTATTGCCAGAATTCCTGAAATAAATCCAGAATAAATGCAAGCACCATGATTTAA
ACCTTGATGGGTTGTGGATACCACTGTCCACCGAACCATTTCAACCAGAGTTTGGT
TCGCCCCAGCCACCAAGACTTGCCATTCGATCCGATCACCATAAAATATTATACCC
TCAAACTTTTCGATCGGTTTATTACTTGTCCAATAGCCACCACCATGAGCTCTCGGA
TGGCCGGAGCCACGCTGCTGCGCCACCTGGGTCCCCGCCTCTTCACCGCCGCCG
ACCCGGCGTCCGGGCTCGCTGCGAGCGCGAGGGGCATC
>*TaAOX1d-2AL.2* promoter
GGAATGAAACGAGGGAAGGGAAGCGAATTGAGGAGGCCAACTTGGTGGCTAGGA
GAGTTGCTGCACTCATGGAGGAGTGGAAGAACGCGAAGGGGAGGCCACATCGAT
CGAGCCCATCTACGCAACGGGCAACCTGGGAACCACCGGCGGCGGGCTGGTTAA
AGGCCAACGTGGATGGTGCAATGGCTCGGTCAAGTCAGGGAGGAGGGTGTGGCG
TGGTGTTCAGAGATGAGGTGGGAGCTTTCCGAAGAGCTGATGCGGTGTTTCTTCC
TGGGGTCACATCTGCCGAAGCAGCTGAGTTGCAAGCGTGCAAGCGAGCGGTGGT
TTTAGCTATGCAACGGGCTGTGCCTAAGCTCCATCTGGAGACCGACTGCCAGAAC
GTGGCTAGAATGTTGAAGAGCAAGAGCACAACCTGTCATCGGTGGGAATCATGGT
GAAAGAAATCAAGGGGATGGCCAAAAGCTTGGGGGAGTTTAAAGCTACCTGGGTG
AAGAGGGAGGCAAATAAAGCGGCCCATGAGATTGCCCGATTCGGTTTTTGTAATA
GAATTTCTGTTTCGTGGGAGGTTTCTCCTCCAGACTGTATTCTTAGCATAGTCTCAG
ATGAACTTCCTGGTTTAGTTTAATAAAGTGGGGGAGCAAATTTAAAAAAAAAAGAAA

CCTTTTTTTTAGCGACTTACATATAGCTAAAGTGCTATTTTATGAGGGAGGAGCCAG
AAACGTTTTTTCCTTTGAAATTTTCAGCCGGGCCATGGTCAGCGTCTAGAGATAGT
CGAGCTGATGGAATCTTTCATGGAATATATCCTCGTGAAGAAGACGGCGAACGGT
CAACCCGCCTTATCGGTTGACCTCCAAAGGAGAAGACTAGTACGAGTAGTATGCAT
GCACGGCAGTCCCGTGGCATCTACGCACAGGCACGGCAAGTCAAGCTCGCCGGG
CGTTCGTGTCCGGATCGCACAGCGCAACCTGATGGCAACATACACTGCGCCGGTC
AACCGAGACGTCTCGCGAACTCCCCCGGAAGCCGGCGACGACGTCCCCGTCACG
AGCGTTCGTGTTGGTCCGTTTACAAAAACTTATCTAGCTAGCTAGACAACCAGACA
GACACTTGCACGGCAAGGAATTAGTTGGATTATTCGGCACCAGCGCGCCGCCGCG
CCACGGCTGTGCTGCACTGTCCAAGAAGCAAGAATAAACGGAAAAAAATTGGAAC
GTCGCAGTCGCGGCTGTAGCTGGTGGACGAGTGCGCACATGTCTTCTTTTTCCAG
GGAGAGTGTCCACTGTTGTCTGGTGACATTCCCGGAAGCACCGTGCCGGCCGAG
CCCCCTCCCTATAAAACCATCTCCGGCGAGACGTCGTCAAGCCATCGGTCAGTTA
CCAGATCAACACCAACAGTAAACAGAAATCATTTCCTAGCCCGCCAACAAAAACAG
ACTTCCCATTCGATCCGCTCACAAGCTTTTCGATCGCTTTAGTTGTTCAACAATCAA
CAGCCAACACC
>*TaAOX1d-2DL* promoter
TTTCGTAGTCGACGGGAACGTCTCCTCCTACTGAACGCACATCGCCGAAAGCCAT
GGAATAAATCCAGAAAAATAAGAGCACCAGTGCCATGTCTATGAATCACCGGAAGC
CATGGAATAAATTCTGGAAAATGCGAGCACCAGTGCCAAGTCTATGAATTAAACCC
TGATGGCATAACATTATAAGGATCGTTTTCTAAGCCCCTATTCGCTGATTATGGTGT
CCTTTATGTGAAAAAATATTTCACGCTTAGCCATTAGAGATATAGAAAATGCTACGA
GAAGGGGAACCTACAGCTAAATTTCGACGCCATTGCATAGGATACTTCTTTTCCAC
GGCCGGCCTCCTACTTCAGAGTAGATATTTGACATTTCAAAGTAAATGAAAAAAAA
GAATGTACTATCACCACTGGAGAGGCAAACCATATGTGTTACTAAATGATCCAACT
ATTTTAGCATGTCAATCTTTTGTTTCACATTTGCTTATGATTGCTTGTACTTCACTGG
CTTTTCTTTTCTCATTAGCAAATTTGTACATCTAGCCAATGAAATATAACCCATAATG
TCCATTGTTCTTAATAACAACAATAACAAGTGTATAAGTCAAGGCCACTTCCACGGG
CACGGCGTGTCGCCGCGCCTTCACCCGCTAAGTATAGCTAAAGTTCTATTTTATCA
GGGAGAAGCGAGAAACGTTTTTTCCTTTGAAATTTTCAGCCGGGCCGTGTGGTCAG
CGTCTAGAGAGAGTCGAGCCGATGGAATCTTTCATGGAATATATCCTCGTGAAAGA
AGACGGCAAACGTTCAACTCGCCTTACCGGTTGACCTCCAAAGGAGAAGACTAGT
ATGCATGCACGGCAGTCGGTAGTCCCGTGGCATCTACGCACAGGCACGGCAAGT
CAAGCTCGCAGGGCGTTTGTGTCCGGATCGCACAGCGCACTCACCTCAACCTGAT
GGCAACATACGCTGCGCCGGTCAACCGAGATGTCTCGCGAACTCCCCCGGAAGC
CGGCGAGTCCGACGACGTTCCTGTCATGAGCGTTCGTGTTGGTCCGTTCAGAAAA
ACTTATCTAGCTAGTCAACCTGACAGACACTTGCACGGCAAGGAATTGTTAGATTA
TTAAGCACCAGCGCGCCGCCGCGCCACGGCTGTGCTGCACTGTCCAAGAAGCAA
CAATAAACGGAAAAAATTGGAACGTCGCAGTCGCAGCACTCGCAGCTGTAGCTGG
TGGTCGAGTGCGCACATGTCTTCTTTTTCTAGGGAGAGTGCCCACATGTTGTCTGG
TGACATTCCCGGAAGCACCGTGCCGGCCGAGCCCCCTCCCTATAAAACCATCTCC
GGCGAGACGTCGTCAAGCCATCGGTCAGTTACCAGATCAACACCAACAGTAAACA

GAAATCATTTCCTAGCCCGCCAACAAAAACAGACTTCCCATTCGATCCGCTCACAA
GCTTTTCGATCGGTTTAGTTGTTCAACAATCAACAGCCAACAGCTCCCGG

>*put.regTaAOX-3B* promoter
ACTTTTTTAAAATAATTGCCGTATTACAAGTTTATTATTTTTTCTAGTAACTTGGTCAC
ATATAATGATACAATTCGAAGGTTTTCTAATTTATTGATTTTTTTGAATTTTTTATGCC
CGTTTCAAAATTCAGTCAAAACGGCGGGCATGGCCGTTCCTAGCTAGTGGTTGAAT
ATTGGAAAAGTTTTGGTGTTTCTCTGATTAAATAGATACTTATGTACCTAGAAATGAT
TTTTTAAAAATTAAAGAGCAAGCAATAAGGCAGCTGCAGTTCAAACATGACCCGCTT
CCAGCTGGATCGGCGGAAATTTGTCTTTTTCACCGGAGGTGGGTCGAAACTTTCGA
CACCCAACCATTTGATCAATTGTATATTAAATATGTACTATTATTTTAGAAAATTGAT
GGAGTCCAATTTTGCAACAAATATTTGATAGCTTCTTCACAAAAGAACCCATTTTTG
CCACTCGAAAAAATGATTAAAAATAGCTAAAAAGGTAAAAAAAATGCATACAAATTG
GTGCTCATCCATAAAATGTGGTCTAACTTTAGTGAAAATTTGTGTGGTGCCCTTTTG
CAAAATATTTTTGATAGTTGCTTCACAAAATCCTTCTCTTTTTAGGACATGAAAACTA
TTTTAAATCAATGATTTTTCCAACAAATCAGAACTCTTCTCACGGATCACCCCATTGT
AGCTGATCTGAACCGTCCATATCCAACAGATCCAACACCTCCCACGCCTCTCACCC
CATTGTAGCCCAACCAAAACCCTAGCAGCCCAAGGCCGCCTCTCTCTCCCCCAGA
ACACACCCGCCGCCGCCTCTCTCTCTTCCCCTCTCCCCGATCCAGATCGACAACG
ACGGCCCCCATCGCTGCCCCCTCCTTCCCCATCCCGCCGTCGCCTCTCTCTCCCA
CCCACCCCGATCCATCCCCTTCCTTTGTCTCTCACATCACCTCCACACCCATCCCC
TCCCCTGGCGGGATTCGCCGCTCCCTCTTCCTCCGTCTCGCTCACACACACAGCC
GCTGCCGCCGCTGCAACCCAAACCCTCTCCCTCCCACCCTGATCCGCTGTCACCC
CACCGTCTATGACCCAGATCGAGCAAGCGAGGCGGTGCTTCCAGACACGCGATG
GCGGCAGAGGGACGGGGCCGGGGGCGTGTGCCAGATCTGCACTGACGGCCTAG
GCACCACGCTCGAAGGCAAGTTCTTCAGCGCAACCCCCCAACCCCACCACAGCAA
CAAGGGCAAGCGTGCTTCAACATCAGACGACTCGTCCCGGATTGTGATTCACGGA
GGCGTTTCTCGGCGGCCAGATGAGCTCCCGGATGGCCGGATCGGTCCTCCTCCG
CCGTGTCGGCGCCGGCGCCAACCGCCTCTTCGCCACCACCGCGACGTCCCCGGG
GGCCAGGACCTCCCTCACTGGCGGCAAGGGGGCGTTGGTGCGGATGATGTCCAC
TTCTGCGGCCTCGCAGGTGGTCAAGGCGGAGGCGGCCAAGGGCG

>*put.TaAOX1e-3DS* promoter
TCCGGAGTGCTCTTACAATTGTCATTGCTGATGTCAGCCGCATCGGACGGGAGAC
AATAGGATTAGGCAAGTCATTCCGTACGAGATGCATTTCCACGCAATAACAGCATC
GCAAGCTATATCTTGGATCTGCTAAGAAAAAAGAAACCACGTCTCATTTTCTTTTAA
GAGAGATTCTTGACGTGTTTGCCATGGAGATTTTCTTGCCAACTAAAAGCTGGCGA
TGTAACTCAATTTTATCTCAAATTTTCATCGCAACTCAGAGCACCATTCGATCTCAT
GCAGCCTGGCACTGTGACGAGTTGGTACACGAAGTAACGTTGCACAAGCTCTGGT
CCACAGAGGATCCGAGCACATGCATGCAAAAGGAGAAACAGCGTGGTACACTCGC
TCGCATGCATGCATGCATGCTCTTCAAGTTGCCAACTTGGCTCCCCTCAAAAAAAA
AGTTGCCAACTTGGCATGCATTGCATGCAGGGTCAGCAGCTAGGAAGCGCAAGCG
CAAGTGCCTACGTCTCTTCCTATGAAGCACCGATACGGCAACACTGATACGTCGAT

ACGGGTACGGTGATACGGGATACGGTGATTTCTAGAAACAACAATACGGCGATAC
GGCAAATATATATAAATAATAATAATATGACATGTAACAAAAACTAAGATAATAAAAT
ATGTGAGATGAGATCAACATCAACAAGACAAAAGTAATTTTATCCAACATTAGCATG
TAGCAACAGATCGAAGTACTACTAGATCAACACGTCAACAGAAGCCATCAGCAACA
AGATCAACGCATCAAAGAATTGAGAGAGTACTGTTACTAGTCTACTACCTACAGCA
ACGGATCAACAGAAGCCATCGGCAACCCAGCAGCCAAGCATACATCAACGGAATA
ACCACACATCAGTATAGCATCAAGCGGCAGCGGCGAGCCACTCCAGGCGGCGGA
GGCGACGGCGGCAGCGGCGGCGAGCCACTGCTGGTGGCGGCGGCAGCAGTCGC
CCTGGATCCCGCTGTTATCGTGAGAGAGTGCGAGAGAGGATTTTAGGTTTAGTGG
TCGTGGGCTATTGGGCTCGCTCGGTGCTGCGCTCGGCTGCCTCGGCGTGTCCCA
TACGTGTCACGAGCGTGTCCCAGCCGTATCGGCATTTTTTCCTTATTTTTTTAAATA
AAAAAGAGGATACTTCTAGGATATCCGTATCTAAGCCGTATCTGGCGTATCGACGT
ATCGACGACGTCCTGAACGGCGATACAGAGATTTTTGACGTATCGGTGCTAGTCTT
CTCTCCCCTCTCCGCGTATGTATATAAGACCCATCACCGTACACACCGTTTCTCAT
CTACACCAAAACCCATAGGCCCAAAATATACACCAACACCAGTGTCCTTGTCCAGG
CTCGGCAGGAAATGGCCATGGTGCAGTTACTGGTGCAGCGCGGCGTGAGGTCGG
TGGCGACGGCCCAGCTAGGGACGCGCTTCTTCTCCGTGGCCGGCCGCCGAACGC

>*put.TaAOX1d-like-4AS* promoter
TAGCACACGTTCAGCTCGATGACGATCCCCGGACTCCGATCCAGCAAAGTGTCGG
GGAAGAGTTTCGTCAGCACGACGGCGTGATGATGATCTTGATGTTCTATCGTCGCA
GGGCTTCGCCTAAGCACCGCTACAATATTATCGAGGATTATGGTGGAGGGGGGCA
CCGCACACGGCTAAGAGAACGATCATGAAGATCAAAGGTGCCCCCCTGCCCCCGT
ATATAAAGGAGCAAGGGGGGAGGAGGCCGGCCCTAGGAGAGGCGCTCCAGGTGT
GGAGTCCTACTAGGACTCCCTAGTCCTAGTAGGATTCCTCCTCCCATATGGAATAG
GAAAAGAGGAAGGGAAAAGGAGAAGGAAGGTAGGGGGCGCCCCCCTTCCCTAGT
CCAATTCGGACCAGACTAAGGGGAGGGGTGCAGCCACCCTTGAGGCCCTTTTCCT
TCTTTCCCGTATGGCCCAATAAGGCCCAATACGTATTCCCGTAACTCTCCGGTACT
CCAAAAAATACCCGAATCACTCGGAACCTTTCCGATGTCCGAATATAGTCGTCCAA
TATATCGATCTTTACGTCTCGACCATTTCGAGACTCCTTGTCATGTCCCCGATCTCA
TCCGGGACTCCGAACTCCTTCGGTACATCAAAACTCATAAACTCATAATATAACTGT
CATCGAAACCTTAAGCGTGCGGACCCTACGGGTTCGAGAACAATGTAGACATGAC
CAAGACACGTCTCCGGTCAATAACCAATAGCGGAACCTGGATGCTCATATTGGCTC
CCACATATTCTACGANNNNNNNNNNNNNNNNNNNNNNNNNNNNNNNNNNNNNNNNNNNNN
NNNNNNNNNNNNNNNNNNNNNNNNNNNNNNNNNNNNNNNNNNNNNNNNNNNNNNNNNNNN
NNNNNNNNNNNNNNNNNNNNNNNNNNNNNNNNNNNNNNNNNNNNNNNNNNNNNNNNNNNN
NNNNNNNNNNNNNNNNNNNNNNNNNNNNNNNNNNNNNNNNNNNNNNNNNNNNNNNNNNNN
NNNNNNNNNNNNNNNNNNNNNNNNNNNNNNNNNNNNNNNNNNNNNNNNNNNNNNNNNNNN
NNNNNNNNNNNNNTAGTGACACTCTGTTTGTCTATGTATTCACACATGTATTATGTT
TCCGGTTAATACAATTCTAGCATGAATAATAAACATTTATCATGATATAAGGAAATAA
ATAATAACTTTATTATTGCCTCTAGGACATATTTCCTTCACGAGACGTTCCCGACAC
GTGAGTTCCCCTATAAAACCATCTCCAGCAGGACGTTTCCTGTCATCAGTCAGTTT
ACCAGATCAACACCAACAGAAATCACTTCCCAGCCACCAAGACCTCCCATTCGATC

CGGTCCCAACATAAATACCCCCAAAGCTTTTCGACCAGTTTATTAGTTGTTCAAAAG
CCACCATCATGAGCTCACGGATGGCCGGAGCCAGCTACTGCGCCACCTGGGCCC
CCGCCTCTTCGCCGCCACCGAGCCGGCGTCCAGACTCGCCGCGAGCGCGAGGG
GCATC

>*put.regTaAOX-6BL* promoter
NNNNNNNNNNNNNNNNNNNNNNNNNNNNNNNNNNNNNNNNNNNNNNNNNNNNN
NNNNNNNNNNNNNNNNNNNNNNNNNNNNNNNNNNNNNNNNNNNNNNNNNNNNNN
NNNNNNNNNNNNNNNNNNNNNNNNNNNNNNNNNNNNNNNNNNNNNNNNNNNNNN
NNNNNNNNNNNNNNNNNNNNNNNNNNNNNNNNNNNNNNNNNNNNNNNNNNNNNN
NNNNNNNNNNNNNNNNNNNNNNNNNNNNNNNNNNNNNNNNNNNNNNNNNNNNNN
NNNNNNNNNNNNNNNNNNNNNNNNNNNNNNNNNNNNNNNNNNNNNNNNNNNNNN
NNNNNNNNNNNNNNNNNNNNNNNNNNNNNNNNNNNNNNNNNNNNNNNNNNNNNN
NNNNNNNNNNNNNNNNNNNNNNNNNNNNNNNNNNNNNNNNNNNNNNNNNNNNNN
NNNNNNNNNNNNNNNNNNNNNNNNNNNNNNNNNNNNNNNNNNNNNNNNNNNNNN
NNNNNNNNNNNNNNNNNNNNNNNNNNNNNNNNNNNNNNNNNNNNNNNNNNNNNN
NNNNNNNNNNNNNNNNNNNNNNNNNNNNNNNNNNNNNNNNNNNNNNNNNNNNNN
NNNNNNNNNNNNNNNNNNNNNNNNNNNNNNNNNNNNNNNNNNNNNNNNNNNNNN
NNNNNNNNNNNNNNNNNNNNNNNNNNNNNNNNNNNNNNNNNNNNNNNNNNNNNN
NNNNNNNNNNNNNNNNNNNNNNNNNNNNNNNNNNNNNNNNNNNNNNNNNNNNNN
NNNNNNNNNNNNNNNNNNNNNNNNNNNNNNNNNNNNNNNNNNNNNNNNNNNNNN
NNNNNNNNNNNNNNNNNNNNNNNNNNNNNNNNNNNNNNNNNNNNNNNNNNNNNN
NNNNNNNNNNNNNNNNNNNNNNNNNNNNNNNNNNNNNNNNNNNNNNNNNNNNNN
NNNNNNNNNNNNNNNNNNNNNNNNNNNNNNNNNNNNNNNNNNNNNNNNNNNNNN
NNNNNNNNNNNNNNNNNNNNNNNNNNNNNNNNNNNNNNNNNNNNNNNNNNNNNN
NNNNNNNNNNNNNNNNNNNNNNNNNNNNNNNNNNNNNNNNNNNNNNNNNNNNNN
NNNNNNNNNNNNNNNNNNNNNNNNNNNNNNNNNNNNNNNNNNNNNNNNNNNNNN
NNNNNNNNNNNNNNNNNNNNNNNNNNNNNNNNNNNNNNNNNNNNNNNNNNNNNN
NNNNNNNNNNNNNNNNNNNNNNNNNNNNNNNNNNNNNNNNNNNNNNNNNNNNNN
NNNNNNNNNNNNNNNNNNNNNNNNNNNNNNNNNNNNNNNNNNNNNNNNNNNNNN
NNNNNNNNGGCGGTGCTTCCAGATCCGCGATGGCGGTAGTGGGACGGGGTCGG
GGGCGTGTGCCAGATCTGGGCCGACGGCCTGGGCACCACGCTCAAAGGCAAGGT
CTTCAGTGCAACTCCCCAACCCCACCCCCACCCACCCACCACAACAACAAGGGCA
AGCGTGCGTCAACATCAGACGACTCATCCCGGATTGTGATTC

TuAOX:
>*TuAOX1a* promoter
TTCCCGGTTTAAAAAAATAACAATTAGTTTTTTTCAAACAAATCGTCGTTCATTGTGC
ATGACACTGTCTCACATATTATATTCTACCACTAACCAAACTCCATGAGTGGAGGCA
CACCGTCACAAATTGAATACGTTCACTTTTGCAAGCTTAAAGTAAAGTAATCCCCTA
GCAGCCACTGGCCTAAAAAAATACGTACAATATAAAAAAAATGCCCGCAAAATAAA
CAAACTTCAAAATTAAATTAAATTGAAGTTAAAACGAGTGCTTACATGGCTAACCAC

GAGGCGTAATTCGTGTCCCGCCTAGGCATCACACGCACGCCGGTCCGGTCGCAA
AGCCGCGAACACGTGAGCCTCTCGTTGGATGCCAGTGGTCACAGGCCGTCTCCAC
GCGGAAGGGAAAAAAAAGGGGGGGAAGAAAATCGCAACGACCAAAATTAATGCGA
TCGATCGACTTGGAGGAGAAGAAAAGGCCTCCTCAGTTTCTGGGATCCCCGGGAG
GCAGGNNNNNNNNNNNNNNNNNNNNNNNNNNNNNNNNNNNNNNNNNNNNNNNNNN
NNNNNNNNNNNNNNNNNNNNNNNNNNNNNNNNNNNNNNNNNNNNNNNNNNNNNNN
NNNNNNNNNNNNNNNNNNNNNNNNNNNNNNNNNNNNNNNNNNNNNNNNNNNNNNN
NNNNNNNNNNNNNNNNNNNNNNNNNNNNNNNNNNNNNNNNNNNNNNNNNNNNNNN
NNNNNNNNNNNNNNNNNNNNNNNNNNNNNNNNNNNNNNNNNNNNNNNNNNNNNNN
NNNNNNNNNNNNNNNNNNNNNNNNNNNNNNNNNNNNNNNNNNNNNNNNNNNNNNN
NNNNNNNNNNNNNNNNNNNNNNNNNNNNNNNNNNNNNNNNNNNNNNNNNNNNNNN
NNNNNNNNNNNNNNNNNNNNNNNNNNNNNNNNNNNNNNNNNNNNNNNNNNNNNNN
NNNNNNNNNNNNNNNNNNNNNNNNNNNNNNNNNNNNNNNNNNNNNNNNNNNNNNN
NNNNNNNNNNNNNNNNNNNNNNNNNNNNNNNNNNNNNNNNNNNNNNNNNNNNNNN
NNNNNNNNNNNNNNNNNNNNNNNNNNNNNNNNNNNNNNNNNNNNNNNNNNNNNNN
NNNNNNNNNNNNNNNNNNNNNNNNNNNNNNNNNNNNNNNNNNNNNNNNNNNNNNN
NNNNNNNNNNNNNNNNNNNNNNNNNNNNNNNNNNNNNNNNNNNNNNNNNNNNNNN
NNNNNNNNNNNNNNNNNNNNNNNNNNNNNNNNNNNNNNNNNNNNNNNNNNNNNNN
NNNNNNNNNNNNNNNNNNNNNNNNNNCTCTGCTCTGCTTTTCCCGTTTCGGTTCC
GTCGCCGGCGACGGGTCGCCAGATCGGAGCGTTTGTGCGGTCGACGGGCGCTAT
TATTATTTTATTTCCTTGTTGCGCCGCCGCCGTGTGTGATTGATCGGTTGGTTTTGC
TGCCCGCCGCAGCCATGGGAGACGTACACCGCGGACACGTCGATCGATCTGACC
AAGCACCACGTGCCCAACACG
>*TuAOX1c* promoter
TGGGCAGCGCAGTACACTGAATTTATAGCGCAAAAAACCATGCAAAAAAACGGTG
GTATGGAGAATGAAACTAGCACCGCTACTTCTCTCGTATTGCTACAATAACCACTA
GAGCTTCTCAACGGTAGTAGTGACTAATAGCAAAGCAAATTCTTTTAGAACTAATAG
AGTGCGCTATTTATTACTCAGTCAATTACTATATCACTTAGATTTTTGTGTTATTTGA
AAATAATTTGGAAAAAGATTATCTTTTTATGAAAAATAATTAAATTTTGAAAAAGTTCA
CTAATTTCAAGGAAAATCATCAATTTTTGAAAAAAGTTCATCATTTTTGGAAAAAGTT
CATCGATTTGGAAAAAAAGTTCATCGATTTTGAAAAAAACATCAAATTAAAAAATGCT
TTTTGAAAAAAATAAAGAAAAAACAGAAAAAGAAAGAAAAAACAAAAACAAAAACCG
GTGCAGAAAAACCCAACAACGAAAAAGAAAAAACGGCTAGGGAAAATATGAAAGAA
GAAAAGTAAATGGATTACCATAACATTTTTTATATGAAACACCTTGCATTCCATTAAC
ATGTGGTCGCGACAAGATCGCCGGTAACTGCAAGGGTTACATCAGCAGGCACAGG
AAAATTCGTTATCCACAAAGTATGGTCACACTGGTTTGGTCGCGGGTTCGTTTCTTT
CCAGTGTACATTTTTTTGCATATTCAAAACCAGAAAAAGGAGTGGCATGGGCCGGC
CCAGCGCGCAGAAGGGGGGTGTGCTCCGGTTTGCCCAATAGCCTATAACCGGCC
CCTCAAGCGCTAAATAGGAAGCGCTCCTTGCCATCAAGCAAGCGAAAATTATATGA
TTTGCAGTTGCACCTTCTGATACGCTCGCCGCACGAAGGGTCTTCATTTCTCCGGT
CTATCTCTAAGAGCATCTCCAACAGCCGCACTAAATAAGCGCCGCACCGTAAATTT
GTTTTTTTTAGCGTGCGCAATCGATATAGGGGCTTCAGCGGACGCGCAATAAGCGT
GCGCGGCATATAGAGTTGGGCGCGCGGTCTGAATCACGATCGCGCGCTGTGTATT

TGATGCACCCACTTCCGTGCGCCGCACACTCGAGTGCTCGCGCCGCACTCTCTCT
GCCGCGTCACCTTCGCCCCGCCCGNNNNNNNNNNNNNNNNNNNNNNNNNNNNNN
NNNNNNNNNNNNNNNNNNNNNNNNNNNNNNNNNNNNNNNNNNNNNNNNNNNNNN
NNNNNNNNNNNNNNNNNNNNNNNNNNNNNNNNNNNNNNNNNNNNNNNNNNNNNN
NNNNNNNNNNNNNNNNNNNNNNNNNNNNNNNNNNNNNNNNNNNNNNNNNNNNNN
NNNNNNNNNNNNNNNNNNNNNNNNNNNNNNNNNNNNNNNNNNNNNNNNNNNNNN
NNNNNNNNNNNNNNAGGGGGTGCGACGGGCTCGGCGGCTACTGAAGCGCCGGC
GAGCTCACTTGTTGAGCCGGCGGCCGACGCGCACAAGGTGTTCAAGGAA
>*TuAOX1d.1* promoter
TCCATCTGGAGACCGACTGCCAGAACGTGGCTAGAATGTTGAATGAGCAAGAGCG
CAACCTGTCATCGGTGGGAATCATGGTGAAAGAAATCAAGGGGATGGCCAAAAGC
TTGGGGGAGTTTAAAGCTACCTAGGTGAAGAGGGAGGCAAATAAAGCGGCCCATG
AGATTGCCCGATTCGGTTTTTGTAATAGAATTTCTGTTTCGTGGGAGGTTTCTCCTC
CAGACTGTATTCTTAGTATAGTCTCAGATGAACTTCCTGGTTTAGTTTAATAAAGTG
GGGGAGCAAATTTCAAAAAAAAAAGAAACCTTTTTTTTAGCGACTTACATATAGCTA
AAGTGCTATTTTATGAGGGAGGAGCCAGAAACGTTTTTTCCTTTGAAATTTTCAGCC
GGGCCATGGTCAGCGTCTAGAGATAGTCGAGCTGATGGAATCTTTCATGGAATATA
TCCTCGTGAAGAAGACGGCGAACGGTCAACCCGCCTTATCGGTTGACCTCCAAAG
GAGAAGACTAGTACTGAGTAGTATGCATGCACAGCAGTCCCGTGGCATCTACGCA
CAGGCACGGCAAGTCAAACTCGCCGGGCGTTCGTGTCCGGATCGCACAGCGCAA
CCTGATGGCAACATACACTGCGCCGGTCAACCGAGACGTCTTGCGAACTCCCCCG
GAAGCCGGCGACGACGTCCCCGTCACGAGCGTTCGTGTTGGTCCGTTTACAAAAA
CTTATCTAGCTAGCTAGACAACCAGACAGACACTTGCACGGCAAGGAATTAGTTGG
ATTATTCGGCACCAGCGCGCCGCCGCGCCACGGCTGTGCTGCACTGTCCAAGAA
GCAAGAATAAACGGAAAAAATTTGGAACGTCGCAGTCGCGGCTGTAGCTGGTGGA
CGAGTGCGCACATGTCTTCTTTTTCTAGGGAGAGTGTCCACATGTTGTCTGGTGAC
ATTCCCGGAAGCACCGTGCCGGCCGAGCCCCCTCCCTATAAAACCATCTCCGGCG
AGACGTCGTCAAGCCATCGGTCAGTTACCAGATCAACACCAACAGTAAACAGAAAT
CATTTCCTAGCCCGCCAACAAAAACAGACTTCCCATTCGATCCGCTCACAAGCTTT
TCGATCGCTTTAGTTGTTCAACAATCAACAGCCAACACCATGAGCTCCCGGATGGC
CGNNNNNNNNNNNNNNNNNNNNNNNNNNNNNNNNNNNNNNNNNNNNNNNNNNN
NNNNNNNNNNNNNNNNNNNNNNNNNNNNNNNNNNNNNNNNNNNNNNNNNNNNNN
NNNNNNNNNNNNNNNNNNNNNNNNNNNNNNNNNNNNNNNNNNNNNNNNNNNNNN
NNNNNNNNNNNNNNNNNNNNNNNNNNNNNNNNNNNNNNNNNNNNNNNNNNNNNN
NNNNNNNNNNNNNNNNNNNNNNNNNNNNNNNNNNNNNNNNNNNNNNNNNNNNNN
NNNNNNNNNNNNNNNNNNNNNNNNNNNNNNNNNNNNNNNNNNNNNNNNNNNNC
GAGGGGCATC
>*TuAOX1d.2* promoter
CGACACCCTTCATAAAGATTGCTTCTGAGGGTGTTCAGTTCAGGATCATGCCAACT
CAATAACCATTCCCTCCTTGTGCATCAACAAAAATTTAGCGAACCTAGAACATGACC
AAGAAGGCAAACATCCAAAAACTGACTATTACTGTCCATACAAGGGATTGACCAAT
CAATAGTTTGTTGGGGAACGTAGCAGAAATTCAAAATTTTCTACGCATCACCAAGAT
CAATCTATGGAGTAATCTAGCAACGAGGGGAAGGAGAGTGCATCTACATACCCTTG

TAGATCGCTAAGCGGAAGCGTTCAAGTGAACGGGGTTGATGGAGTCGTACTCGTC
GTGATTCAAATCACCGATGATCAAGTGCCGAACGGACGTCACCTCCGCGTTCAAC
ACACGTACAGCCCGGCGACGTCTCCCACGCCTTGATCCAGCAAGGAGAGAGGGA
GAGGTTGAGGAAGACTCCATCCAGCAGCAGCACAACGGCGTGGTGGTGATGGAG
GAGCGTGGCAATCCTGCAGGGCTTCGCCAAGCACCACGGGAGAGGAGAAGGACT
TAGGAGAGAGGTAGGGGCTGCACCAAAGGCATAAGGGGTGTCTCAAGAGGCCC
TCCTACCCTCACTATATATAGGGAGCCCAAGGGGGGGTGCGCCAGCCCTAGGA
GATCCAATCTCCTAGGGGGCGGCGGCCAGGGGAGGTCCCGATGGCCGAAATAGC
ACTTCCTATATATAATTCTTTACCTCCGGACCATTCCGGAACTCCTCGTGACGTCCG
GTATCTCATCCATGACTCCGAACAACATTCGGTAACCACATACAAACTTCCTTTATA
ACCCTAGCGTCATCGAACCTTAAGTGTGTAGACCCTACGGGTTCGGGAGACACGT
AGACATGACCGAGATGACTCTCCGGCCAATAACCAATAGCGGGATCTGGATACCC
ATGTTGGCTCCCACATGTTCCACGATGATCTCATCGGATGAACCACGATGTCAAGG
ACTTAATCAATCCCGTATACAATTCCCTTTGTCTATCGGTATTGTACTTGCCCGAGA
TTCGATCATCGGTATCCCGATACCTTGTTCAATCTCGTTACCGGCAAGTCTCTTTAC
TCGTTCCGTAACACATCATCCCGTGATCAACTCCTTGATCACATTGTGCACATTATG
ATGATGTCCTACCGAGTGGGCCCAGAGATACCTCTCCGTTTACACGGAGTGACAA
ATCCCAGTCTCGATTCGTGCCAACCCAACAGACACTTTCGGAGATACCTGTAGTGC
ACCTTTATAGTCACCCAGTTACGTTGTGACGTTTGATACACCCAAAGCACTCCTAC
GGTATCCGGGAGTTGCACAATCTCATGGTCTAAGGAAATGATACTTGACATTAGAA
AAGCTTTAGCATACGAACTACATGATCTAGTGCTATACTTAGGATTGGGTCTTGTCC
ATCACATCATTCTCCTAATGATGTGATCCCGTTATCAATGACATCCAATGTCC
AetAOX:
>*AetAOX1a* promoter
GTATTTGGTCACGTTCCGGTGCTGTTTAGCTGTTGGATTAAGGGAAGGATCTATTG
ATAGCGGTTGCCTCGTTTGACGGGTTCTTTCTTGGTCCACAAGCAAATATTAGGAC
GGATGGATGGATGGGCATCTGCCGGAACACATCTCCCATTTCGCTTTGTTTCCTTT
CCGCCAGATATTTGCCAACCGTAACAAAGATAAACTGCACGCACCCGCCAAGTGT
GAAAGGGTGCCCCCCCCCCCCCCCCCCTACGAGAACTACTTCAGAACCTGATTG
TACAGCTCGGCTTCGATGATCCTTTTCCCGGTTTTTAAAAAAATCATTTGGTTTTTC
GAAACAATCGTCGTTCATTGTACAGGACACTGTCTCAAATATTATATTCTACGTACT
AACCAAACTCCATGATTGGACGCACAATGTCTCAACTTGAATACGTTCACTTTTTGC
AAGCTTAAAGTTAAAGTAATCCCTTAGCAGCCACGGGCCTAAAAAAAATACTTACAA
TACTAAAAAAATCCCAGCAAAAAAGCAAAATTCGGAATTAAATCAAACGAAGTTAAA
ACGAGTGCTTACATGGCTAACACCGAGACGTAATTCGTGTCCCGCCTGAGCATCA
CACGCCGGTCCGGTCGCAAAGCTGCAAACACGTGAGCCTTTCGTTGGATGCCAGT
AGTCACAGGCCGTTTCCACGCGGAAGGAACAAAAAAAAAGCAAGAAGAAAATCGC
AACGACCAAAATTAATGCGATCGATCGACTTGGAGGAGAAGAAAAGACCCCCTCA
GTTCCTGGGATCCCCGGGAGGCAGCGGCTGGCTCCCCCGCCACTGACGCCGCG
GCCCCACCCCCTGCCCTCGCCGCCTCCGCCATTGATCCCCCTTCCCATATAAAAT
CCCTGCAACGAATGACCCTTCCATTAGCAAACCGCAACCCCCACCCCACCCCACC
CCACCCACCACAGCAACGAGGGCAAGCGTGCGTCAACATCAGACGACTCGTCCC
GGATTGTGATTCGCGGAGGCGTTTCCCGGCGCCCAGATGAGCTCCCGGATGGCC

GGNNNNNNNNNNNNNNNNNNNNNNNNNNNNNNNNNNNNNNNNNNNNNNNNNNN
NNNNNNNNNNNNNNNNNNNNNNNNNNNNNNNNNNNNNNNNNNNNNNNNNNNNNN
NNNNNNNNNNNNNNNNNNNNNNNNNNNNNNNNNNNNNNNNNNNNNNNNNNNNNN
NNNNNNNNNNNNNNNNNNNNNNNCTTGCTTCAGGGTCCGCTCTTCGCTCGCTTCC
TCCGCTCCGCCCCCGCTCTGCTCTGCTTTTCCCGTTTCGGTTCCGGCACCGGCGA
GGGATCGCCAGATCGGAGCGTGTTGTATGCGGTCGATTGGCGCTATTATTTTATTT
CCTTGTTGCGCCGCCGCCGTATGTGATTGACTGATTGGTTTTGTTGCCCGCCGCA
GCCATGGGAGACGTACACCGCGGACACGTCGATCGATCTGACCAAGCACCACGT
GCCCAACACG
>*AetAOX1e* promoter
CAGGGGTTGTGCAGGGTTAGAGGGATGGCCGAGCCAGAGGTTTAAGGTAGATTG
GGGCGGTGAGGTGTGCACGGAAGCGCTGCCGACTGCGAGAGACGGTAGTAGGT
GGCCCTGCCAACGCTGCGAGAGACGGGGCAGGCAGACCAGCCGGCACTGGTTTG
GGTGGTTGGAGAAGGAAGAAGATGAGAGATATAAGAAGCACAACAATTGTTAGATT
TATTGAAAGTCAACAATGGCAGAATTGACTATTGTGACTTTTTTTAAAAAATGGAGG
CATTTTCTTGCGTACGGCCATGTGAACATATGTGGGCCCCTACTATTGGCCAGTCT
CACAACTCATCTTGAGACGCGACAATTGAGGTTGGTAGTGATGAAAATGATTTCAA
CCTCCGACAAAGTTTCAGATTATGGATGAAAACGAAGATCAACTCGGAAACATGAT
GTAGCCGCGGTGGAAATACTCGAATTGGAGGTGCAAATGGTCTAAAAGCCATGGG
GCACCATAGGCCAAACGGGGCCCCACAGCCTTTAGGTGCCCTCAGCCCTAGGACT
CCTAGGGGCGCCTAAGGAACGTGGGGCCCACCTGTTGTGAGGCCAAGCCTCCAT
GGTCTTTTGGTTCCTTTAAACTTCCAAAACTATTTTTGTTTTATTTTCCTCAGATTTTT
TTTAGGTAATTTTTTTGAAATACAAAAATACCTAAAACAAAAACTAGCACTAGGCACT
GGATTAATATCTTACTTCAATAAAATTTTAAAAAATTATGCATGGAGTGTTTATAATT
GATAGCACTATAATAGAAATTATACTAAAATGTTGATACGTTTGAGACGAATTAGCA
TCCCAAGCTTAATCCATACTCGTCCTCGAGCATGTAGATGATAAAAAGGTAAATTTT
GAAGGATGAATGCTACTAATCATGATATTGATTTAACTGATGTATCACCTACGCTAA
ATATAAAGCAGTAAAATATATTTAGCAAAGGTCTAAATAATATGAAAACTTGAGAGTT
TTGGCAGAAATGTATACACTACTCTCTCTGTCTTATAATATAAGTTTTTGCAATTTAT
TTTAGCTTGCAAAAATGTCGTGTATTATAGGACGCAGGGGTACTAGATAGCCTAGT
GTATGAGATGCGATGTATGCCCCACTGATGGCGAGTACGTCCCACTTTGGCATGC
ATCCACCTAGTGTGAACTTAACTGTGCAACTTATATTCCATTCCGCTTGTTCGCTTT
TTTATGTCTCATGTTGCATTTGGACTTACCATACCAGTACAGAGCCTTATAAAAGAA
ATATATAATTTGTCCGGAGTGCTCTTACAATTGTCATTGCTGATGTCAGCCGCATCG
GACGGGAGACAATAGGATTAGGCAAGTCATTCCGTACGAGATGCATTTCCACGCA
ATAACAGCATCGCAAGCTATATCTTGGATCTGCTAAGAAAAAAGAAACCACGTCTC
ATTTTCTCTTAAGAGATCGAGATTCTTGACGTGTTTGCC
>*AetAOX1d* promoter
GCGAGAAACGTTTTTTCCTTTGAAATTTTCAGCCGGGCCGTGTGGTCAGCGTCTAG
AGAGAGTCGAGCCGATGGAATCTTTCATGGAATATATCCTCGTGAAAGAAGACGG
CGAACGTTCAACTCGCCTTACCGGTTGACCTCCAAAGGAGAAGACTAGTATGCATG
CACGGCAGTCGGTAGTCCCGTGGCATCTACGCACAGGCACGGCAAGTCAAGCTC
GCAGGGCGTTTGTGTCCGGATCGCACAGCGCACTCACCTCAACCTGATGGCAACA

TACGCTGCGCCGGTCAACCGAGATGTCTCGCGAACTCCCCCGGAAGCCGGCGAG
TCCGGCGACGTTCCTGTCATGAGCGTTCGTGTTGGTCCGTTCAGAAAAAAACTTAT
CTAGCTAGTCAACCTGACAGACACTTGCACGGCAAGGAATTGTTAGATTATTAAGC
ACCAGCGCGCCGCCGCGCCACGGCTGTGCTGCACTGTCCAAGAAGCAACAATAA
ACGGAAAAAATTGGAACGTCGCAGTCGCAGCACTCGCAGCTGTAGCTGGTGGTCG
AGTGCGCACATGTCTTCTTTTTCTAGGGAGAGTGCCCACATGTTGTCTGGTGACAT
TCCCGGAAGCACCGTGCCGGCCGAGCCCCCTCCCTATAAAACCATCTCCGGCGA
GACGTCGTCAAGCCATCGGTCAGTTACCAGATCAACACCAACAGTAAACAGAAATC
ATTTCCTAGCCCGCCAACAAAAACAGACTTCCCATTCGATCCGCTCACAAGCTTTT
CGATCGCTTTAGTTGTTCAACAATCAACAGCCAACACCATGAGCACCCGGAGTGTG
CCGCTTGTTACTTCGAATCGCTAGGTTTCACGGATCATCAGCAAGGCAAGTAACAC
TTTGATCATACCTTTCCATACNNNNNNNNNNNNNNNNNNNNNNNNNNNNNNNNNNNNNNN
NNNNNNNNNNNNNNNNNNNNNNNNNNNNNNNNNNNNNNNNNNNNNNNNNNNNNNNNNNNN
NNNNNNNNNNNNNNNNNNNNNNNNNNNNNNNNNNNNNNNNNNNNNNNNNNNNNNNNNNNN
NNNNNNNNNNNNNNNNNNNNNNNNNNNNNNNNNNNNNNNNNNNNNNNNNNNNNNNNNNNN
NNNNNNNNNNNNNNNNNNNNNNNNNNNNNNNNNNNNNNNNNNNNNNNNNNNNNNNNNNNN
NNNNNNNNNNNNNNNNNNNNNNNNNNNNNNNNNNNNNNNNNNNNNNNNNNNNNNNNNNNN
NNNNNNNNNNNNNNNNNNNNNNNNNNNNNNNNNNNNNNNNNNNNNNNNNNNNNNNNNNNN
NNNNNNNNNNNNNNNNNNNNNNNNNNNNNNNNNNNNNNNNNNNNNNNNNNNNNNNNNNNN
NNNNNNNNNNNNNNNNNNNNNNNNNNNNNNNNNNNNNNNNNNNNNNNNNNNNNNNNNNNN
NNNNNNNNNNNNNNNNNNNNNNNNNNNNNNNNNNNNNNNNNNNNNNNNNNNNNNNNNNNN
NNNNNNNNNNNNNNNNNNNNNNNNNNNNNNNNNNNNNNNNNNNNNNNNGCCTCCGGGCTC
GCCGCGAGCGCGAGGGGCATC
>*AetAOX1d-like* promoter
TGGAGCCCTTTAGACACGAACGGCCATCTCCGACTGGACATGACCAAGTCTCGGA
CTATTCCACTCAGCACTCAAGCTCAACACCTGCTTGGCCTCGTCTTCCATTTTGATG
CGCCCACCCACCATGTCAAACGCTCCCGTTCTCCTGCGCGTGCGTCGGTGCGTGC
ACGGGACCCATGGAATCTGTCGTCCCCATCATCCCGTCCGCGATGAAGAATGGTC
AAGTCGCCTCCCCGGTTGACCCCCAAAGGCCAAGACTAGTATGAAGCAGGTGGCG
CGAGCACGGCCGTCCCGTGCGTGGCATCCACGGCACCAGCAGCACGGCAAGTCA
AGCTCGGCTCGCAAGGTGGCTGTGTCCGGATCGCGCAGCGCAACCGATGGATGG
CTCGCGAACTCCCCCGGAAGCCGGCGACGACGTTCCCCGTCACGAGCTCCTCGG
CCACTCCGTTAGGAATCCTTTACTAGTCAACCAGACAGAGACTTGCACGGCAAGCA
GTTGATCCCGTTTAGAATATTTTTTAGCAAAGATTCCGTTTAGATTAGACAACGGCT
GTGCTGCGCTGCGCTGCCGCTCTGCCCAGGAAGCAGCGCGCCGGCCGAGCCGC
CGCACTACTGCATATAATTTGTGTGGTAGGTAGTAGGTACAACTGTCTCAGGTCAA
ATATGTCCACCTTTCTAAATTTCAGTAGTCTGTCTTTTTGCGGGGCAAATTAAGTAC
TCTGTTAGCAGCCCCTCATGCACAAATACTAGTGTACTATTGTGCATATTTTTTTTT
CAAAAAAACTTCCAATCTATTCATCTTCAATCATGGCAGTACAACGAACACCAGAAA
TAAAAATTACATCCAGATCCGTAGACCACCTAGCGAAGATTACAAGCACCGAAGCG
AGCCGAAGGCGCGCCGCCGTCAACGCCCCTCCATCACCGGAGCCAGGCACAACT
TGTTGTAATAGATGGTCGGAAAATTGTCGTGCTAAGGCCCCATAGGACCAGCCCC
CCAGAATAGCAACCGCCGTCGATGAAAAATAACGTAGATCGGAAGGATCCAAACC

GAAAACACACGAACGTAGACGAACAACGACGAGATCCGAGCAAATCCACCAAAGA
TATAACAACATAGAGTAAGGATGAAACCATAAAATAATAACTTATTTTGAACTGAAG
CACACAAGAATTGGATCATCGATTGCAGTTAGCTGTTGGGCGAGTGCGCACATGT
CTTTTTTTTTTTACTTCTTCTCTATCCAGGGGAGAGTGCGCACATGTTGTGTTTAGT
GGCATTTCTGGGAAGCACCGTGCCGTGCCGGCCGAGACGTTCCCGACACGCGAC
CCCCCTATAAAACCATCTCCAGCAAGACGTCTCTTGCCATCAGTCAGCTTACCAGA
TAAACACCAACAGAAATCACTTCCCAGCCACCAAAGACTTCCCATTCGATCCGATC
ACCACAAAAAACCTCAAGCTTCTCGACCGGTTTATTAGTTGTTCAACAGCCAACAC
C

<u>GENE BODIES</u>

TaAOX:

>*TaAOX1a-2AL.sv1* gene
ATGAGCTCCCGGATGGCCGGATCGGTCCTCCTCCGCCGCGCCGGCGCCGGCGC
CGGCCGCCTCTTCGCCACCACCGCGTCCCCGGCGGCCAGGACCGCCCTCGGTG
GAGGTGAGGGCGCGTGGGTGCGGATGATGTCCACCTCCGCGGCCTCGCAGGTCA
AGGATGAGGCGGCCAAGGGGGTCAAGGCGGAGGCGGCCAAGGGCGACGGGGA
GAAGAAGGAGGTGGCCATCAGCAGCTACTGGGGGATCGAGCAGTCGAAGAAGCT
GGTGCGCGAGGACGGCACCGAGTGGAAGTGGTCTTGCTTCAGGGTACGCTCTTC
CCTTGCTTCCTCTGCTCTGCTTTTCCCGTTTCGGTTCCGTCGCCGGCGACGGGTG
CCAGATCGGAGCGCGTACGCGGTCGATTGGCGCTGTTATTTTATTTCCTTGCTGCG
CCGCCGCCGTCTGTGATTGATTGGTTGGTTTTGTTGCCCGCCGCAGCCATGGGAG
ACGTACACCGCTGACACGTCGATCGATCTGACCAAGCACCACGTGCCCAACACGA
TGCTCGACAAGATCGCCTACTACACCGTCAAGTCCCTGCGCTTCCCCACCGACAT
CTTCTTCCAGGTACGCGCGCCTCACCGGCCGCCACAACCGCCGGATGGTTCGTTC
CGTGTTTTTTAGTCCAACCCGTTTCGCCGAAGACAGCACGATCGGCGGTGTCTGC
GTCTTTGCGTGTCATGGCCTCGTGGGTGACGGATGTAGCATGGTCCCTGTCACGC
TTTTGATTCTGTTCCCCCCTGCTTTCCGGGGAGACGGCGTTTGGACTTGCTATTTA
GAACGCGGACCCAAGCCGTCATCCCGAGTATTTTTTACCTAATCGTTCGTTCGTGG
GGATGGGTGTTTTATATTCTGCATCAGATTACTGTGGATAATCGCGTGCTGGAGAA
ATCCGCTGACAGTGGATGGAAGTATCGACAGATTATAGACTGTCAGCAAACATTAG
GAACTGGAAACACAAATAACATGGCGACCTTGACATGCTACTACATCGGACATTTT
ATTATTGATTAAAGCACCAGAGAGGATGCCATTTGTTTTGCCATGTACTTTACTTTAT
TAAAGCATCTGTTTATCATGATTTTTGCACAAGCTGTATCATCTTGATCTCCACTCTG
TATTTTGAAGTTCATCATGATCTGAAGCTGACCCTTCAAATTGGATGCAGAGGAGG
TACGGCTGCCGCGCAATGATGCTGGAGACTGTTGCCGCCGTGCCGGGGATGGTG
GGCGGCATGCTCCTGCACCTGCGCTCCCTCCGGCGCTTCGAGCAGAGCGGCGGC
TGGATCCGCGCGCTGCTGGAGGAGGCCGAGAACGAGCGCATGCACCTCATGACC
TTCATGGAGGTGGCGCAGCCGAGGTGGTACGAGCGCGCCCTCGTCATCGCCGTC
CAGGGCGTCTTCTTCAACGCCTATTTCTTCGGCTACCTCATCTCGCCCAAGTTCGC
ACACCGCGTCGTCGGGTACCTCGAGGAGGAGGCCGTCCACTCCTACACCGAGTT
CCTCAAGGACCTCGACGACGGCAAGATCGACAACGTCCCCGCCCCTGCCATCGC
CATCGACTACTGGCGCCTCCCTGCCAACGCCACCCTCAAGGACGTGGTCACCGTG

GTCCGCGCCGACGAGGCTCACCACCGCGACGTCAACCACTTCGCATCGGTACGC
ATCCTTCCAAATCCCACAAGATCAGCAAGTCAATCCTGACCCCATTTCCTGGCCAG
TCCATGCTCATCTGTTTATGTGGATTGGTTCTGAACTTCTGATGATGCATGCTGATT
TGGTCTTGTCGATTGTGCCGTGCAGGACGTGTACTACCAGGGTATGCAGCTGAAG
GCCACCCCGGCGCCGATCGGATACCACTGA
>*TaAOX1a-2AL.sv2* gene
ATGAGCTCCCGGATGGCCGGATCGGTCCTCCTCCGCCGCGCCGGCGCCGGCGC
CGGCCGCCTCTTCGCCACCACCGCGTCCCCGGCGGCCAGGACCGCCCTCGGTG
GAGGTGAGGGCGCGTGGGTGCGGATGATGTCCACCTCCGCGGCCTCGCAGGTCA
AGGATGAGGCGGCCAAGGGGGTCAAGGCGGAGGCGGCCAAGGGCGACGGGGA
GAAGAAGGAGGTGGCCATCAGCAGCTACTGGGGGATCGAGCAGTCGAAGAAGCT
GGTGCGCGAGGACGGCACCGAGTGGAAGTGGTCTTGCTTCAGGGTACGCTCTTC
CCTTGCTTCCTCTGCTCTGCTTTTCCCGTTTCGGTTCCGTCGCCGGCGACGGGTG
CCAGATCGGAGCGCGTACGCGGTCGATTGGCGCTGTTATTTATTTCCTTGCTGCG
CCGCCGCCGTCTGTGATTGATTGGTTGGTTTTGTTGCCCGCCGCAGCCATGGGAG
ACGTACACCGCTGACACGTCGATCGATCTGACCAAGCACCACGTGCCCAACACGA
TGCTCGACAAGATCGCCTACTACACCGTCAAGTCCCTGCGCTTCCCCACCGACAT
CTTCTTCCAGGTACGCGCGCCTCACCGGCCGCCACAACCGCCGGATGGTTCGTTC
CGTGTTTTTTAGTCCAACCCGTTTCGCCGAAGACAGCACGATCGGCGGTGTCTGC
GTCTTTGCGTGTCATGGCCTCGTGGGTGACGGATGTAGCATGGTCCCTGTCACGC
TTTTGATTCTGTTCCCCCCTGCTTTCCGGGGAGACGGCGTTTGGACTTGCTATTTA
GAACGCGGACCCAAGCCGTCATCCCGAGTATTTTTTACCTAATCGTTCGTTCGTGG
GGATGGGTGTTTTATATTCTGCATCAGATTACTGTGGATAATCGCGTGCTGGAGAA
ATCCGCTGACAGTGGATGGAAGTATCGACAGATTATAGACTGTCAGCAAACATTAG
GAACTGGAAACACAAATAACATGGCGACCTTGACATGCTACTACATCGGACATTTT
ATTATTGATTAAAGCACCAGAGAGGATGCCATTTGTTTTGCCATGTACTTTACTTTAT
TAAAGCATCTGTTTATCATGATTTTTGCACAAGCTGTATCATCTTGATCTCCACTCTG
TATTTTGAAGTTCATCATGATCTGAAGCTGACCCTTCAAATTGGATGCAGAGGAGG
TACGGCTGCCGCGCAATGATGCTGGAGACTGTTGCCGCCGTGCCGGGGATGGTG
GGCGGCATGCTCCTGCACCTGCGCTCCCTCCGGCGCTTCGAGCAGAGCGGCGGC
TGGATCCGCGCGCTGCTGGAGGAGGCCGAGAACGAGCGCATGCACCTCATGACC
TTCATGGAGGTGGCGCAGCCGAGGTGGTACGAGCGCGCCCTCGTCATCGCCGTC
CAGGGCGTCTTCTTCAACGCCTATTTCTTCGGCTACCTCATCTCGCCCAAGTTCGC
ACACCGCGTCGTCGGGTACCTCGAGGAGGAGGCCGTCCACTCCTACACCGAGTT
CCTCAAGGACCTCGACGACGGCAAGATCGACAACGTCCCCGCCCCTGCCATCGC
CATCGACTACTGGCGCCTCCCTGCCAACGCCACCCTCAAGGACGTGGTCACCGTG
GTCCGCGCCGACGAGGCTCACCACCGCGACGTCAACCACTTCGCATCGGTACGC
ATCCTTCCAAATCCCACAAGATCAGCAAGTCAATCCTGACCCCATTTCCTGGCCAG
TCCATGCTCATCTGTTTATGTGGATTGGTTCTGAACTTCTGATGATGCATGCTGATT
TGGTCTTGTCGATTGTGCCGTGCAGGACGTGTACTACCAGGGTATGCAGCTGAAG
GCCACCCCGGCGCCGATCGGATACCACTGA
>*TaAOX1a-2BL* gene

ATGCCAGTAGTCACAGGCCGTTTCCACGCGGGAGGAAAAAAAAAAGAGGAGGAAG
AAAATCGCAACGACCCATTAATGCGATCGATCGACTTGGAGGAGAAGAAAAGACC
CCCTCAGTTCCTGGGATCCCCGGGAGGCAGGGCCGGCTCCCCCGCCACTGACGC
CGCGGCCCCACCCCTGCCCTCGCCGCCTCCGCCATCGATCCTCCTTCCCAATATA
AAATCCATCCGCGGAAAGACCCTTCCATTCGCAAACCGCAACCCCCCAACCCCAC
CCCCACCCACCCCCACAGCAACCAACGCAGGCGAAGGCGCGCGTCGACGTCACG
CCCACGACCCGTCCCGGAGTTTTGGGGTGTTCGCGGAGGCGTTTCCCGGCGCCC
AGATGAGCTCCCGGATGGCCGGATCGGTCCTCCTCCGCCGCGCCGGCGCTGGCG
CCAGCCGCCTCTTCGCCACCACCCCGACGTCCCCGGCGGCCAGGACCGCCCTCG
CCGGCGGCGACGGCGCGTGGGTGCGGATGATGTCCACCTCCGCGGCCTCGCAG
GTCAAGGACGAGGCGGCTAAGGCGGTCAAGGCGGAGGCGGCCAAGGGCGACGG
GGAGAAGAAGGAGGTGGCGATCAGCAGCTACTGGGGGATCGAGCAGTCGAAGAA
GCTGGTGCGCGAGGACGGCACCGAGTGGAAGTGGTCTTGCTTCAGGGTACGCTC
TCCGCTTGCTCTGCTCTTCTCTGCTTCTCCCGTTTCGATTCCGACACCGGCGACGG
GACGCCAGATCGGAGCGTGTATGCGATGGATTGGCGCTATTATTTTATTTCCTTGT
TGCGCCGCCGCCGTGTGTGATTGATTGGTTGCTTTCGCTGCCGCAGCCATGGGAG
ACGTACACCGCGGACACGTCGATCGATCTGACCAAGCACCACGTGCCCAACACGA
TGCTCGACAAGATCGCCTACTACACCGTCAAGTCCCTGCGCTTCCCCACCGACAT
CTTCTTCCAGGTACGCGCGCCTCCCCCCTCCCCTCCCCTAGTCCCCTCGCCGGCC
GCCACAACCGCCGGATAGTTCGTTCCGTGTGTTTTTAGTCCAACCCGTTTCGCCGA
AGACAGCACGAACGGCGGTGTCTGCATCTTTGCGTGTCATGGCCTCCTGACTGAC
GGAAGTAGCATGGTCCCTATCACGCTTTTGATTCTGTTTTCCCCCTGCTTTCCGGG
GAGACGGCGTTTGGACTTTCTATTTAGAACGCGGACCCAAGCGGTCAAGCGGAGT
ATTTTTACCTAATCGTTCGTGGAGATGGGTGTTTTATATTCAGTTTTGCATCAGATTA
CTGTGGATAATCGTACTGACATTTCCGTGCTGATTATTTCTATCTGGATACAAGTAG
GCGACAGTGGATAGATGTATCCACAGATTATTTACTGTCAGCAAACATTACGAATT
GAAAACACAAATAACATGGTGACCTTGACATGACCTTAACATAGGATATTTTATATT
GTTGTAAAGCACCAGAGAGGATTCCATTTGTTTTGCCATGTACTGTACTTTATTAAA
GCATCTGTTTATCATGATTTTTGCACAAGTTGTATCATCTTGATCTCCTCTCTGTGTT
TCGAAGTTTATCATGTTCTAAAGCCGATCCTTCAAATTGGTTGCAGAGGAGGTATG
GCTGCCGCGCAATGATGCTGGAGACTGTTGCCGCAGTGCCGGGGATGGTGGGCG
GCATGCTCCTCCACCTGCGCTCGCTCCGGCGCTTTGAGCAGAGCGGTGGTTGGAT
CCGCGCGCTGCTGGAGGAGGCCGAGAACGAGCGCATGCACCTCATGACCTTCAT
GGAGGTGGCGCAGCCGAGGTGGTACGAGCGCGCCCTCGTCATCGCCGTCCAGG
GCGTCTTCTTCAACGCCTACTTCTTCGGCTACCTCATCTCGCCCAAGTTCGCGCAC
CGCGTCGTCGGGTACCTCGAGGAGGAAGCCGTCCACTCCTACACCGAGTTCCTCA
AGGACCTTGACGACGGCAAGATCGACAACGTCCCCGCCCCGGCCATCGCCATCG
ACTACTGGCGCCTCCCTGCCAACGCCACCCTCAAGGACGTGGTCACCGTGGTGC
GCGCCGACGAGGCTCACCACCGCGACGTCAACCACTTCGCATCGGTACGCATCCT
TCCAAATCCCACAAGATCAGCAAGGCAATCTTGACCCCATTTCCTGCCCAGCCCAT
GTGCATCTGTTTATGTGGATTGGTTCGGAACTTCTGATGATGTATATGCTGATTTGG
TCTGTTGCATGTGCCGTGCAGGACGTGTACTACCAGGGTATGCAGCTGAAGGCCA
CCCCAGCGCCGATCGGATACCACTGA

>*TaAOX1a-2DL.sv1* gene
ATGAGCTCACGGATGGCCGGATCGGTCCTCCTCCGCCGCGCCGGCGCCGGCGCC
AGCCGCCTCTTCGCCACCACCCCGTCTCCGGCGGCCAGGGCCGTCCTCGGTGGA
GGTGAGGGCGCGTGGGTGCGGCTGATGTCCACCTCCGCGGCCTCGCAGGTCAAG
GACGAGGCGGCCAAGGCGGTCAAGGCGGAGGCGGCCAAGGCGGTCAAGGCGGA
GGCGGCCAAGGGCGACGGGGAGAAGAAGGAGGTGGCCATCAGCAGCTACTGGG
GGATCGAGCAGTCGAAGAAGCTGGTGCGCGAGGACGGCACCGAGTGGAAGTGGT
CTTGCTTCAGGGTACGCTCTTCGCTCGCTTCCTCCGCTCCGCCCCCGCTCTGCTC
TGCTTTTCCCGTTTCGGTTCCGGCACCGGCGAGGGATCGCCAGATCGGAGCGTGT
TGTATGCGGTCGATTGGCGCTATTATTTTATTTCCTTGTTGCGCCGCCGCCGTATG
TGATTGACTGATTGGTTTTGTTGCCCGCCGCAGCCATGGGAGACGTACACCGCGG
ACACGTCGATCGATCTGACCAAGCACCACGTGCCCAACACGATGCTCGACAAGAT
CGCCTACTACACCGTCAAGTCCCTGCGCTTCCCCACCGACATCTTCTTCCAGGTAT
GCGCGCCTCCCCCCTCCCTTCCCCTCGCCGGCCTTCACGACCGCCGGTTGATCCT
CCGGATGGTTCGTTCCGTGTTTTTTTAGTCCAACCCGTTTCGCCGAAGACGGCACG
AACGGCGGTGTCTGCATCTTTGCGTGTCATGGCCTCATGGGTGACGGAAGTAGCA
TGGTCCCTGTCGCGCTTTTGATTCTGTTTCCCCCTGCTTTCCGGGGAGACGGCGTT
TGGACTTGCTATTTAGAACGCCGACCCAAGCGGTCTAGCGGATTATTTTTTACCTA
ATCGTTCGTGGGAATGGGTGTTTTATATTCTACTGTATCAGATTACTGTGGATAATC
GCGTGCTGGAGAAATCCGCTGATTATTTCTATCTGGATACAAGTAGGCGACAGTAT
CGACAGATTATAGACTGTCAGCAGACATTAGGAATTGAAAACACAAATAACATGGT
GACCTTGACATCGCTACTGCATAGGATATTTTATTGATTGTAAAGCACCAGAGAGG
ATTCCATTTGTTTTGCCATGTACTTTACTTTATTAAAGCATCTGTTTATCATGATTTTT
GCACAAGTTGTATCATCTTGATCTCCTCTCTGTATTTCGAAGTTTATCATGATCTGA
AGCTGACCCTTCAAATTGGATGCAGAGGAGGTATGGCTGCCGCGCAATGATGCTG
GAGACTGTTGCCGCAGTGCCGGGGATGGTGGGCGGCATGCTCCTCCACCTGCGC
TCGCTCCGGCGCTTCGAGCAGAGCGGCGGCTGGATCCGCGCGCTGCTGGAGGA
GGCCGAGAACGAGCGCATGCACCTCATGACCTTCATGGAGGTGGCGCAGCCCAG
GTGGTACGAGCGCGCCCTCGTCATCGCCGTCCAGGGCGTCTTCTTCAACGCCTAC
TTCTTCGGCTACCTCATCTCGCCCAAGTTCGCGCACCGCGTCGTCGGGTACCTCG
AGGAGGAGGCCGTCCACTCCTACACGGAGTTCCTCAAGGACCTCGACGACGGCA
AGATCGACAACGTCCCCGCCCCGGCCATCGCCATCGACTACTGGCGCCTCCCTGC
CAACGCCACCCTCAAGGACGTGGTCACCGTGGTGCGCGCCGACGAGGCTCACCA
CCGCGACGTCAACCACTTCGCATCGGTACGAATCCCACATCCTTTCTTGACCCCAT
TTCCTGACCAGTCCATGTGCATCTGTTTTGTGTGGATTGGTTCTGAATTTTCTGATG
ATTTTGATCTGTCGCATGTGTCGTGCAGGACGTGTACTACCAGGGTATGCAGCTGA
AGGCCACCCCGGCCCCGATCGGATACCACTGA
>*TaAOX1a-2DL.sv2* gene
ATGAGCTCACGGATGGCCGGATCGGTCCTCCTCCGCCGCGCCGGCGCCGGCGCC
AGCCGCCTCTTCGCCACCACCCCGTCTCCGGCGGCCAGGGCCGTCCTCGGTGGA
GGTGAGGGCGCGTGGGTGCGGCTGATGTCCACCTCCGCGGCCTCGCAGGTCAAG
GACGAGGCGGCCAAGGCGGTCAAGGCGGAGGCGGCCAAGGCGGTCAAGGCGGA
GGCGGCCAAGGGCGACGGGGAGAAGAAGGAGGTGGCCATCAGCAGCTACTGGG

GGATCGAGCAGTCGAAGAAGCTGGTGCGCGAGGACGGCACCGAGTGGAAGTGGT
CTTGCTTCAGGGTACGCTCTTCGCTCGCTTCCTCCGCTCCGCCCCCGCTCTGCTC
TGCTTTTCCCGTTTCGGTTCCGGCACCGGCGAGGGATCGCCAGATCGGAGCGTGT
TGTATGCGGTCGATTGGCGCTATTATTTATTTCCTTGTTGCGCCGCCGCCGTATG
TGATTGACTGATTGGTTTTGTTGCCCGCCGCAGCCATGGGAGACGTACACCGCGG
ACACGTCGATCGATCTGACCAAGCACCACGTGCCCAACACGATGCTCGACAAGAT
CGCCTACTACACCGTCAAGTCCCTGCGCTTCCCCACCGACATCTTCTTCCAGGTAT
GCGCGCCTCCCCCCTCCCTTCCCCTCGCCGGCCTTCACGACCGCCGGTTGATCCT
CCGGATGGTTCGTTCCGTGTTTTTTTAGTCCAACCCGTTTCGCCGAAGACGGCACG
AACGGCGGTGTCTGCATCTTTGCGTGTCATGGCCTCATGGGTGACGGAAGTAGCA
TGGTCCCTGTCGCGCTTTTGATTCTGTTTCCCCCTGCTTTCCGGGGAGACGGCGTT
TGGACTTGCTATTTAGAACGCCGACCCAAGCGGTCTAGCGGATTATTTTTTACCTA
ATCGTTCGTGGGAATGGGTGTTTTATATTCTACTGTATCAGATTACTGTGGATAATC
GCGTGCTGGAGAAATCCGCTGATTATTTCTATCTGGATACAAGTAGGCGACAGTAT
CGACAGATTATAGACTGTCAGCAGACATTAGGAATTGAAAACACAAATAACATGGT
GACCTTGACATCGCTACTGCATAGGATATTTTATTGATTGTAAAGCACCAGAGAGG
ATTCCATTTGTTTTGCCATGTACTTTACTTTATTAAAGCATCTGTTTATCATGATTTTT
GCACAAGTTGTATCATCTTGATCTCCTCTCTGTATTTCGAAGTTTATCATGATCTGA
AGCTGACCCTTCAAATTGGATGCAGAGGAGGTATGGCTGCCGCGCAATGATGCTG
GAGACTGTTGCCGCAGTGCCGGGGATGGTGGGCGGCATGCTCCTCCACCTGCGC
TCGCTCCGGCGCTTCGAGCAGAGCGGCGGCTGGATCCGCGCGCTGCTGGAGGA
GGCCGAGAACGAGCGCATGCACCTCATGACCTTCATGGAGGTGGCGCAGCCCAG
GTGGTACGAGCGCGCCCTCGTCATCGCCGTCCAGGGCGTCTTCTTCAACGCCTAC
TTCTTCGGCTACCTCATCTCGCCCAAGTTCGCGCACCGCGTCGTCGGGTACCTCG
AGGAGGAGGCCGTCCACTCCTACACGGAGTTCCTCAAGGACCTCGACGACGGCA
AGATCGACAACGTCCCCGCCCCGGCCATCGCCATCGACTACTGGCGCCTCCCTGC
CAACGCCACCCTCAAGGACGTGGTCACCGTGGTGCGCGCCGACGAGGCTCACCA
CCGCGACGTCAACCACTTCGCATCGGTACGAATCCCACATCCTTTCTTGACCCCAT
TTCCTGACCAGTCCATGTGCATCTGTTTTGTGTGGATTGGTTCTGAATTTTCTGATG
ATTTTGATCTGTCGCATGTGTCGTGCAGGACGTGTACTACCAGGGTATGCAGCTGA
AGGCCACCCCGGCCCCGATCGGATACCACTGA
>*TaAOX1a-like-2DL* gene
ATGGTGGGCGGCGTGCTGCTGCACCTGCGCTCGCTCCGCCGCTTCGAGCACAGC
GGCGGCTGGATCCGCGCGCTCATGGAGGAGGCCGAGAACGAGCGCATGCACCTC
ATGACCTTCATGGAGGTGACCCAGCCCCTGTGGTACGAGCGCGCCCTCGTCATCG
CCGTCCAGGGCGTCTTCTTCAACGCCTACTTCTTCGGCTACCTCATTTCCCCCAAG
TTCGCGCACCGCGTCGTCGGCTACCTCGAGGAGGAGGCCGTCCACTCCTACACC
GAGTTCCTCAAGGACCTCGACGACGGCAAGATCGACAACGTCCCCGCCTCGGCC
ATCGCCATCGACTACTGGCGCCTCCCTGCCAACGCCACCCTCAAGGCCGTGGTCA
CCGTGGTGCGCGCCGACGAGGCTCACCACCGCGACGTCAACCACTTCGCATCGG
TATGAATCTTTCCAAATCCCACAAGATCAGCAAGTCAATCTTTGACCCCCATTTCCT
GACCCCATGCTCATCTGTTTTATGCGGATTGGTTCTGAACTTCTGATGATGTATGCT

GATTTGGTCTGTCGATTGTGTCGTGCAGGACGTGTACTACCAGGGTATGCAGCTG
AAGGCCACCCCCGCGCCGATCGGATACCACTGA
>*regTaAOX-4BL.sv1* gene
ATGGTTAGAAGAAGGAGGTGGCGATCAGCAGCTACTTGGGGGATCGAGCAGTCG
AAGAAGCTGGTGCGTGAGGAAGGCACCGAGTGGAAGTGGTCTTGCTTCAGGGTA
CGCTCTTCCCTTGCTTCCTCTGCTCTACTTTTCCTGTTTCGGTTCCGTCGCCGGCG
ACGGGTCGCCAGATGGGAGCGCGCGTGCGGTCGATGTGCGCTGTAATTTTATTTA
CTTGTTGCGCCGCTGTTGTTTGTGATTCATTAGTTGGTTTCACTGCGGCAGCCATG
GGAGGCGTACAGCGCAGACATGTCGATCGATCTGACCAAGCACCATGTGCCCAAC
ACGATGCTCGACAAGATCGCCTACTACACCGTCAAGTCCCCGCGCTTCCCCACCG
ACATCTTCTTCCAGGTACGCATGCTTCCTGGTGTGGCGCCCCTGCTACATTCACGA
GTGCAAGCGTCACGTGGCCGTGAGCAGGCCAGTTCCACGCCAACAAGATGA
>*regTaAOX-4BL.sv2* gene
ATGGTTAGAAGAAGGAGGTGGCGATCAGCAGCTACTTGGGGGATCGAGCAGTCG
AAGAAGCTGGTGCGTGAGGAAGGCACCGAGTGGAAGTGGTCTTGCTTCAGGGTA
CGCTCTTCCCTTGCTTCCTCTGCTCTACTTTTCCTGTTTCGGTTCCGTCGCCGGCG
ACGGGTCGCCAGATGGGAGCGCGCGTGCGGTCGATGTGCGCTGTAATTTTATTTA
CTTGTTGCGCCGCTGTTGTTTGTGATTCATTAGTTGGTTTCACTGCGGCAGCCATG
GGAGGCGTACAGCGCAGACATGTCGATCGATCTGACCAAGCACCATGTGCCCAAC
ACGATGCTCGACAAGATCGCCTACTACACCGTCAAGTCCCCGCGCTTCCCCACCG
ACATCTTCTTCCAGGTACGCATGCTTCCTGGTGTGGCGCCCCTGCTACATTCACGA
GTGCAAGCGTCACGTGGCCGTGAGCAGGCCAGTTCCACGCCAACAAGATGAACCT
GGGCACTGCAACTACCTAAGTGAACGCACAATTCTTCTTCCTGATTCGATTTGGTG
TTGATTTGATTGATTCCTAACGCATCTAATTCTTCTTCTCAGGTACTCTTCTGTTAA
>*regTaAOX-4BL.sv3* gene
ATGGTTAGAAGAAGGAGGTGGCGATCAGCAGCTACTTGGGGGATCGAGCAGTCG
AAGAAGCTGGTGCGTGAGGAAGGCACCGAGTGGAAGTGGTCTTGCTTCAGGGTA
CGCTCTTCCCTTGCTTCCTCTGCTCTACTTTTCCTGTTTCGGTTCCGTCGCCGGCG
ACGGGTCGCCAGATGGGAGCGCGCGTGCGGTCGATGTGCGCTGTAATTTTATTTA
CTTGTTGCGCCGCTGTTGTTTGTGATTCATTAGTTGGTTTCACTGCGGCAGCCATG
GGAGGCGTACAGCGCAGACATGTCGATCGATCTGACCAAGCACCATGTGCCCAAC
ACGATGCTCGACAAGATCGCCTACTACACCGTCAAGTCCCCGCGCTTCCCCACCG
ACATCTTCTTCCAGGTACGCATGCTTCCTGGTGTGGCGCCCCTGCTACATTCACGA
GTGCAAGCGTCACGTGGCCGTGAGCAGGCCAGTTCCACGCCAACAAGATGAACCT
GGGCACTGCAACTACCTAAGTGAACGCACAATTCTTCTTCCTGATTCGATTTGGTG
TTGATTTGATTGATTCCTAACGCATCTAATTCTTCTTCTCAGGTACTCTTCTGTTAA
>*regTaAOX-4BL.sv4* gene
ATGGTTAGAAGAAGGAGGTGGCGATCAGCAGCTACTTGGGGGATCGAGCAGTCG
AAGAAGCTGGTGCGTGAGGAAGGCACCGAGTGGAAGTGGTCTTGCTTCAGGGTA
CGCTCTTCCCTTGCTTCCTCTGCTCTACTTTTCCTGTTTCGGTTCCGTCGCCGGCG
ACGGGTCGCCAGATGGGAGCGCGCGTGCGGTCGATGTGCGCTGTAATTTTATTTA
CTTGTTGCGCCGCTGTTGTTTGTGATTCATTAGTTGGTTTCACTGCGGCAGCCATG
GGAGGCGTACAGCGCAGACATGTCGATCGATCTGACCAAGCACCATGTGCCCAAC

ACGATGCTCGACAAGATCGCCTACTACACCGTCAAGTCCCCGCGCTTCCCCACCG
ACATCTTCTTCCAGGTACGCATGCTTCCTGGTGTGGCGCCCCTGCTACATTCACGA
GTGCAAGCGTCACGTGGCCGTGAGCAGGCCAGTTCCACGCCAACAAGATGA

>*TaAOX1c-6AL* gene
ATGCCATCGTGGCGCGCGCTGGCTCGGCGACAGCGACACGTCATCCCGTCACCC
TCTCAGAGCTTGGCACGTCCGCAGGTTCTCGAGCCTGCGACCACGAGTTTCGCGA
GCAGAGCGGCAGCCCACCAAGCAGGCTCGTCATCTTCGGCGATGAGCTCCCGCG
TCGCCGGATCCGTCCTCCTCCGCCACCTGGGCCCGCGCGTCTTCGGGCCGACCA
CTCCGGCCGCGCAGAGGCCCCTGCTTGCCGGAGGGGAAGGGGGCGCCGTGGCC
GTGGCCATGTGGGCGCGGCCGCTGTCCACCTCCGCCGCCGAGGCGGCGAGGGA
GGAGGCGACCGCGTCCAAGGACAACGTGGCGAGCACCGCCGCCGCGACGGCCG
AGGCGATGCAGGCCGCCAAGGCCGACGCTGTGCAGGCCGCGAAGGAGGGCAAG
AGCCCCGCGGCGAGCAGCTACTGGGGCATCGTGCCTGCCAAGCTGGTGAACAAG
GACGGCGCCGAGTGGAAGTGGTCTTGCTTCAGGGTAATGAAATCGTCTGCATATT
CTCCTTGTCGTTGACAGTGCACGTAGCCGTGGTGACCGTGTGATTTCGCAGCCGT
GGGAGGCGTACACGTCGGACACGACCATCGATCTCTCCAAGCACCACAAGCCCAA
GGTGCTGCTCGACAAGATCGCCTACTGGACCGTCAAGTCGCTGCGCGTGCCCACC
GACATCTTCTTCCAGGTACGGCACGGGGGTCAGATATATGTGTTCGTCCGACACT
GACATGACACGGCGGGTCGACTTGGGCCTACGCTATGCAGCGGAGGTACGGGTG
CCGGGCGATGATGCTGGAGACGGTGGCGGCGGTGCCGGGGATGGTGGGCGGGA
TGCTGCTGCACCTGCGGTCGCTGCGGCGGTTCGAGCAGAGCGGCGGGTGGATCC
GGGCGCTGCTGGAGGAGGCGGAGAACGAGCGGATGCACCTGATGACCTTCATGG
AGGTGGCCAACCCCAAGTGGTACGAGCGCGCGCTGGTGCTGGCGGTGCAGGGC
GTCTTCTTCAACGCCTACTTCCTGGGGTACATCGTGTCCCCCAAGTTCGCGCACCG
CGTCGTGGGCTACCTGGAGGAGGAGGCCATCCACTCCTACACCGAGTTCCTCCGC
GACCTGGAGGCCGGCAGGATCGAGAACGTCCCCGCCCCGCGCATCGCCATCGAC
TACTGGCGCCTCCCCGCCGACGCCAGGCTCAAGGACGTCGTCACCGTCGTGCGC
GCCGACGAGGCGCACCACCGCGATGTCAACCACTTCGCCGCGGTACGCTCATCT
CCCATGCATTAGTCCCCGATGTCTCGACGTCTGAACTCTGCTCTGCTAGCTTCTCC
TGATGTGATGTCTGGACTCTGAACTGTTTTTTTGATTCTGGTGAATGATGATGGGCA
TGCAGGACATCCATTTCCAGGGGCTGGAGCTCAACAAGACGCCTGCCCCGCTAGG
ATATCACTGA
>*TaAOX1c-6BL.sv1* gene
ATGGACGAGCGCACGCAGAAGCTGTCCACCAGTCAATTGCGAGCTCGTAAATACT
CTACCAGCCAAGCAGAGCCGCCGTTCATCCCACGTCTCGCGTCGTCTGCTCGTAG
CGCCACGCCATCGCGGCGCGCGCTGGCTCGGCGACACGTCGTCAAGTCACCCTC
TCAGAGCTTGGCACGTCCGCAGGTTCGCGAGCCTACGACCACGAGTTTCGCGAG
CAGAGCGGCAGCCCACCAAGCAGGCTCGTCATCTTCGGCGATGAGTTCCCGCGT
CGCCGGATCCGTCCTCCTCCGCCACCTGGGCCCGCGCGTCTTCGGGCCGACCAC
TCCTGCTGCGCAGAGGCCCCTGCTTGCCGGAGGAGAAGGGGGCGCCGTGGTCGT
GTGGGCGCGGCCGCTGTCCACCTCCGCCGCAGAGGCGGCGAGGGAGGAGGCGG

CCGCGTCCAAGGACAACGTGGCGAGCACCGCCGCCGCGACGGCCGAGGCGATG
CAGGCCGCGAAGGCCCAGGCCGTGCAGGCCGCCAAGGAGGGGGGCAAGAGCCC
AGTGAGCAGCTACTGGGGCATCGTGCCTGCCAAGCTGGTGAACAAGGACGGCGC
CGAGTGGAAGTGGTCTTGCTTCAGGGTACTGATCAACTACAAGTTCATGCGTTCTT
GTCGTTGACAGTGCACGTAATCGTGGTGACCGTGTGATTTTGCAGCCGTGGGAGG
CGTACACGTCGGACACGACGATCGATCTCACCAAGCACCACAAGCCCAAGGTGCT
GCTCGACAAGATCGCCTACTGGACCGTCAAGTCGCTGCGCGTGCCCACCGACATC
TTCTTCCAGGTATGGTACGGTGGTCAGATATATGTGTTCGTCAGACACTGACATGA
CACGGCGAGCCGACTTGGGCCTATGCTATGCAGAGGAGGTACGGGTGCCGGGCG
ATGATGCTGGAGACGGTGGCGGCGGTGCCGGGGATGGTGGGCGGATGCTGCT
CCACCTGCGGTCGCTGCGGCGGTTCGAGCAGAGCGGCGGGTGGATCCGGGCGC
TGCTGGAGGAGGCAGAGAACGAGCGGATGCACCTGATGACCTTCATGGAGGTGG
CCAAACCCAAGTGGTACGAGCGCGCGCTGGTGCTGGCGGTGCAGGGCGTCTTCT
TCAACGCCTACTTCCTGGGCTACATCGTGTCCCCCAAGTTTGCGCACCGCGTCGT
CGGCTACCTCGAGGAGGAGGCCATCCACTCCTACACCGAGTTCCTCCGCGACCTC
GAGGCCGGCAGGATCGAGAACGTCCCCGCCCCGCGCATCGCCATCGACTACTGG
CGCCTCCCCGCCGACGCCAGGCTCAAGGACGTGGTCACCGTCGTGCGCGCCGAC
GAGGCGCACCACCGCGACGTCAACCACTTCGCCGCGGTACGCCCATCTCTCTGAT
GCCTGAACTCTGCTCTGCTAGCTTCTCCTGATGTGATGTCTGGACTCTGAACTGTT
TTGATTCTGATGAATGATGATGGGCTGGGCATGCAGGACATCCATTTCCAGGGGCT
GGAGCTCAACAAGACGCCTGCCCCGCTAGGATATCACTGA
>*TaAOX1c-6BL.sv2* gene
ATGGACGAGCGCACGCAGAAGCTGTCCACCAGTCAATTGCGAGCTCGTAAATACT
CTACCAGCCAAGCAGAGCCGCCGTTCATCCCACGTCTCGCGTCGTCTGCTCGTAG
CGCCACGCCATCGCGGCGCGCGCTGGCTCGGCGACACGTCGTCAAGTCACCCTC
TCAGAGCTTGGCACGTCCGCAGGTTCGCGAGCCTACGACCACGAGTTTCGCGAG
CAGAGCGGCAGCCCACCAAGCAGGCTCGTCATCTTCGGCGATGAGTTCCCGCGT
CGCCGGATCCGTCCTCCTCCGCCACCTGGGCCCGCGCGTCTTCGGGCCGACCAC
TCCTGCTGCGCAGAGGCCCCTGCTTGCCGGAGGAGAAGGGGGCGCCGTGGTCGT
GTGGGCGCGGCCGCTGTCCACCTCCGCCGCAGAGGCGGCGAGGGAGGAGGCGG
CCGCGTCCAAGGACAACGTGGCGAGCACCGCCGCCGCGACGGCCGAGGCGATG
CAGGCCGCGAAGGCCCAGGCCGTGCAGGCCGCCAAGGAGGGGGGCAAGAGCCC
AGTGAGCAGCTACTGGGGCATCGTGCCTGCCAAGCTGGTGAACAAGGACGGCGC
CGAGTGGAAGTGGTCTTGCTTCAGGGTACTGATCAACTACAAGTTCATGCGTTCTT
GTCGTTGACAGTGCACGTAATCGTGGTGACCGTGTGATTTTGCAGCCGTGGGAGG
CGTACACGTCGGACACGACGATCGATCTCACCAAGCACCACAAGCCCAAGGTGCT
GCTCGACAAGATCGCCTACTGGACCGTCAAGTCGCTGCGCGTGCCCACCGACATC
TTCTTCCAGGTATGGTACGGTGGTCAGATATATGTGTTCGTCAGACACTGACATGA
CACGGCGAGCCGACTTGGGCCTATGCTATGCAGAGGAGGTACGGGTGCCGGGCG
ATGATGCTGGAGACGGTGGCGGCGGTGCCGGGGATGGTGGGCGGATGCTGCT
CCACCTGCGGTCGCTGCGGCGGTTCGAGCAGAGCGGCGGGTGGATCCGGGCGC
TGCTGGAGGAGGCAGAGAACGAGCGGATGCACCTGATGACCTTCATGGAGGTGG
CCAAACCCAAGTGGTACGAGCGCGCGCTGGTGCTGGCGGTGCAGGGCGTCTTCT

TCAACGCCTACTTCCTGGGCTACATCGTGTCCCCCAAGTTTGCGCACCGCGTCGT
CGGCTACCTCGAGGAGGAGGCCATCCACTCCTACACCGAGTTCCTCCGCGACCTC
GAGGCCGGCAGGATCGAGAACGTCCCCGCCCCGCGCATCGCCATCGACTACTGG
CGCCTCCCCGCCGACGCCAGGCTCAAGGACGTGGTCACCGTCGTGCGCGCCGAC
GAGGCGCACCACCGCGACGTCAACCACTTCGCCGCGGTACGCCCATCTCTCTGAT
GCCTGAACTCTGCTCTGCTAGCTTCTCCTGATGTGATGTCTGGACTCTGAACTGTT
TTGATTCTGATGAATGATGATGGGCTGGGCATGCAGGACATCCATTTCCAGGGGCT
GGAGCTCAACAAGACGCCTGCCCCGCTAGGATATCACTGA
>*TaAOX1c-6BL.sv3* gene
ATGGACGAGCGCACGCAGAAGCTGTCCACCAGTCAATTGCGAGCTCGTAAATACT
CTACCAGCCAAGCAGAGCCGCCGTTCATCCCACGTCTCGCGTCGTCTGCTCGTAG
CGCCACGCCATCGCGGCGCGCGCTGGCTCGGCGACACGTCGTCAAGTCACCCTC
TCAGAGCTTGGCACGTCCGCAGGTTCGCGAGCCTACGACCACGAGTTTCGCGAG
CAGAGCGGCAGCCCACCAAGCAGGCTCGTCATCTTCGGCGATGAGTTCCCGCGT
CGCCGGATCCGTCCTCCTCCGCCACCTGGGCCCGCGCGTCTTCGGGCCGACCAC
TCCTGCTGCGCAGAGGCCCCTGCTTGCCGGAGGAGAAGGGGGCGCCGTGGTCGT
GTGGGCGCGGCCGCTGTCCACCTCCGCCGCAGAGGCGGCGAGGGAGGAGGCGG
CCGCGTCCAAGGACAACGTGGCGAGCACCGCCGCCGCGACGGCCGAGGCGATG
CAGGCCGCGAAGGCCCAGGCCGTGCAGGCCGCCAAGGAGGGGGGCAAGAGCCC
AGTGAGCAGCTACTGGGGCATCGTGCCTGCCAAGCTGGTGAACAAGGACGGCGC
CGAGTGGAAGTGGTCTTGCTTCAGGGTACTGATCAACTACAAGTTCATGCGTTCTT
GTCGTTGACAGTGCACGTAATCGTGGTGACCGTGTGATTTTGCAGCCGTGGGAGG
CGTACACGTCGGACACGACGATCGATCTCACCAAGCACCACAAGCCCAAGGTGCT
GCTCGACAAGATCGCCTACTGGACCGTCAAGTCGCTGCGCGTGCCCACCGACATC
TTCTTCCAGGTATGGTACGGTGGTCAGATATATGTGTTCGTCAGACACTGACATGA
CACGGCGAGCCGACTTGGGCCTATGCTATGCAGAGGAGGTACGGGTGCCGGGCG
ATGATGCTGGAGACGGTGGCGGCGGTGCCGGGGATGGTGGGCGGGATGCTGCT
CCACCTGCGGTCGCTGCGGCGGTTCGAGCAGAGCGGCGGGTGGATCCGGGCGC
TGCTGGAGGAGGCAGAGAACGAGCGGATGCACCTGATGACCTTCATGGAGGTGG
CCAAACCCAAGTGGTACGAGCGCGCGCTGGTGCTGGCGGTGCAGGGCGTCTTCT
TCAACGCCTACTTCCTGGGCTACATCGTGTCCCCCAAGTTTGCGCACCGCGTCGT
CGGCTACCTCGAGGAGGAGGCCATCCACTCCTACACCGAGTTCCTCCGCGACCTC
GAGGCCGGCAGGATCGAGAACGTCCCCGCCCCGCGCATCGCCATCGACTACTGG
CGCCTCCCCGCCGACGCCAGGCTCAAGGACGTGGTCACCGTCGTGCGCGCCGAC
GAGGCGCACCACCGCGACGTCAACCACTTCGCCGCGGTACGCCCATCTCTCTGAT
GCCTGAACTCTGCTCTGCTAGCTTCTCCTGATGTGATGTCTGGACTCTGAACTGTT
TTGATTCTGATGAATGATGATGGGCTGGGCATGCAGGACATCCATTTCCAGGGGCT
GGAGCTCAACAAGACGCCTGCCCCGCTAGGATATCACTGA
>*TaAOX1c-6DL* gene
ATGCCATCGTGGCGCGCGCTAGCTCGGCGACACCGACACGTCATCCCGTCACCCT
CTCGGAGCTTGGCACGTCCACAGGTTCTCGATCCTGCGACCACGAGTTTCGCGAG
CAGAGCGGCAGCTCACCAAGCAGGCTCGCCATCTTCGGCGATGAGTTCCCGCGT
CGCCGGATCCGTCCTCCTCCGCCACCTGGGCCCGCGCGTCTTCGGGCCGACCAC

TCAGGCTGCGCAGAGGACCCTGCTTGCCGGAGGGGAAGGGGGCGCCGTGGCCA
TGTGGGCGTGGCCGCTGTCCACCTCCGCCGCCGAGGCGGCGAGGGAGGAGGCG
GCCGCGTCCAAGGACAACGTGGCGAGCACCGCCGCCGCGACGGCCGAGGCGAT
GCAGGCCGCGAAGGCCGAGGCGGTGCAGGCCGCCAAGGAGGGGGGCAAGAGC
CCGGCGAGCAGCTACTGGGGCATCGTGCCTGCCAAGCTGGTGAACAAGGACGGC
GCCGAGTGGAAGTGGTCTTGCTTCAGGGTAATGAAATCCTCTGCATATTCTCCTTG
TCGTTGACAGTGCACGTAGCCGTGGTGACCGTGTGATTTCGCAGCCGTGGGAGGC
GTACACGTCGGACACGACGATCGATCTCACCAAGCACCACAAGCCCAAGGTGCTG
CTCGACAAGATCGCCTACTGGACCGTCAAGTCGCTGCGCGTGCCCACCGACATCT
TCTTCCAGGTACGGCAAGGTGACCGGTCAAGCAAAGTAGATTTCTGCTGGTGTCC
AGGTGCTGACCATGGCACGGCGTGCAGAGGAGGTACGGGTGCCGGGCGATGATG
CTGGAGACGGTGGCGGCGGTGCCGGGGATGGTGGGCGGGATGCTGCTGCACCT
GCGGTCGCTGCGGCGGTTCGAGCAGAGCGGCGGCTGGATCCGGGCGCTGCTGG
AGGAGGCGGAGAACGAGCGGATGCACCTGATGACCTTCATGGAGGTGGCCAACC
CCAAGTGGTACGAGCGCGCGCTGGTGCTGGCGGTGCAGGGCGTCTTCTTCAACG
CCTACTTCCTGGGCTACATCGTGTCCCCCAAGTTCGCGCACCGCGTCGTCGGCTA
CCTGGAGGAGGAGGCCATCCACTCCTACACCGAGTTCCTCCGCGACCTGGAGGA
CGGCAGGATCGAGAACGTCCCCGCCCCGCGTATCGCCATCGACTACTGGCGCCT
CCCGCCCGACGCCAGGCTCAAGGACGTCGTCACCGTCGTGCGCGCCGACGAGG
CGCACCACCGCGACGTCAACCACTTCGCCGCGGTACGCTCATCTCCCATACATGG
TCCCCGATGTATCGACGTCTGAACTCTGCTCTGCTCAGCTTCTCCTGATGTGATGT
CTGGACTCTCAACTGTTTGGATTCTGATGAGTGATGATGGGCATGCAGGACATCCA
TTTCCAGGGGCTGGAGCTCAACAAGACGCCTGCCCCGCTAGGATATCACTGA
>*regTaAOX-3B* gene
ATGCACCTCATGACCTTCATGGAGGTGTCCCAGCCGCGGTGGTACGAGCGCGCG
CTCGTGGTCGCCGTCCAGGGCGTCTTCTTCCACGCCTACCTCGCCACCTACCTCG
CCTCCCCAAAGGTCGCGCACCGCATGGTGGGGTACCTGGAGGAGGAGGCCGTGC
ACTCCTACACCGAGTTCCTTCGTGACCTCGAGGCCGGCAAGATCGACGACGTGCC
CGCGCCGACGAGGCGCACCACCGGGACGTCAAACCACTACGCCTCCGTAAGCGT
CGCCTCCGTCAAATTTGCCAAGCTGATATCAATTGCAGTCTTCTTTGCACTTAAAAT
GCGTGCATCACATGTGTGCAGGACATACATTGCCAGGGGCATGCACTGCGAGAGG
TAGCTGCGCCGATCGGCTACCACTGA
>*TaAOX1d-2AL.1* gene
ATGCCCGCCGCCGCGAGGATCTTCCCCGCGCGGATGGCCAGCACCGAGGCCGC
CGCCCCGCATGCCAAACAAGAAGAAGCCACCGAAAAGCCCCAGGGCGCAACAAC
GCCGGAGCACAACAAGAAGGCCGTGGTGAGCTACTGGGGCATCGAGCCGCGGAA
GCTCGTGAAGGACGACGGCACGGAGTGGACGTGGTTCTCCTTCAGGCCGTGGGA
CACCTACCGCCCGGACACGTCCATCGACATGGCCAAGCACCACGAGCCCAGGGC
GGTGGCGGACAAGGTGGCGTACCTCATCGTGCGGACGCTGCGCGCGGGCAGCG
ACCTCTTCTTCCAGCGCCGGCACGCCAGCCACGCGCTGCTGCTGGAGATGGTGG
CGGCGGTGCCGCCCATGGTGGGCGGCGTGCTGCTGCACCTGCGCTCGCTCCGC
CGCTTCGAGCACAGCAGCGGCTGGATCCGCGCGCTCATGGAGGAGGCCGAGAAC
GAGCGCATGCACCTCATGACCTTCATGGAGGTGACGCAGCCGCTGTGGTGGGAG

CGCGCGCTCGTGCTCGCCACTCAGGGCGTCTTCTTCNCATGGAGGAGGCCGAGA
ACGAGCGCATGCACCTCATGACCTTCATGGAGGTGACGCAGCCGCTGTGGTGGG
AGCGCGCGCTCGTGCTCGCCACTCAGGGCGTCTTCTTCAACGCCTACTTCGTCGG
CTACCTCGTCTCCCCCAAGTTCGCGCACCGCTTCGTTGGCTACCTCGAGGAGGAG
GCCGTCCACTCCTACACCAAATACCTCAAGGACCTCGAGGCCGGCTTGATCGAGA
ACACGCCCGCGCCGGCCATCGCCATAGATTACTGGCGCCTCCCCGCCGACGCCA
GGCTCAAGGACGTCGTCACCGCCGTGCGCGCCGACGAGGCGCATCACCGTGACG
CCAACCACTACGCATCGGACATCCATTACCAGGGAATGACGCTGAATCAGACGCC
TGCGCCACTCGGCTACCACTGA
>*TaAOX1d-2AL.2.sv1* gene
ATGAGCTCCCGGATGGCCGGAGCCACGCTTCTGCGCCACCTGGGCCCCCGCCTC
TTCGCCGCCGCCGAGCCAGCCTCCGGGCTCGCCGCGAGCGCGAGGGGCATCAT
GCCCGCCGCCGCGAGGATCTTCCCCGCGCGGATGGCCAGCACCGAGGCCGCCG
CCCCGCATGCCAAACAAGAAGATGATGCCGCGAGCCCCCAGGCGGCCGCGACTC
CAGAGCAGCAGAACAAGAAGCCCGTGGTGAGCTACTGGGGCATCGAGCCTCGGA
AGCTCGTCAAGGATGACGGCACGGAGTGGCCATGGTTCTGCTTCAGGCCGTGGG
ACACGTACCGGCCGGACACGTCCATCGAAGTGGCCAAGCACCACGAGCCCAAGG
CCCTGGCGGACAAGGTGGCCTACTTCGTGGTTCGGTCGCTGCGCGTGCCCCGGG
ACCTCTTCTTCCAGCGCCGGCACGCCAGCCATGCTCTGCTACTGGAAACGGTGGC
GGCGGTGCCTCCCATGGTGGGCGGCGTGCTGCTGCACCTGCGCTCGCTCCGCCG
CTTCGAGCACAGCGGCGGCTGGATCCGGGCGCTCATGGAGGAGGCCGAGAACGA
GCGCATGCACCTCATGACCTTCATGGAGGTGACGCAGCCGCGGTGGTGGGAGCG
CGCGCTCGTGCTCGCCGCGCAGGGCGTCTTCTTCAACGCCTACTTCGTCGGGTAC
CTCATTTCCCCCAAGTTCGCGCACCGCTTCGTCGGGTACCTCGAGGAGGAGGCCG
TGGAGTCTTATACTGAGTATCTCAAGGACCTTGAGGCCGGATTGATCGAGAACACG
CCCGCGCCGGCCATCGCCATCGACTACTGGCGCCTCCCCGCCGACGCCAGGCTC
AAGGACGTCGTCACCGCCGTGCGCGCCGACGAGGCGCATCACCGCGACGCCAAC
CACTACGCATCGGACGTCCATTACCAGGGAATGACGCTGAATCAATCGCCTGCGC
CGCTCGGGTACCACTGA
>*TaAOX1d-2AL.2.sv2* gene
ATGAGCTCCCGGATGGCCGGAGCCACGCTTCTGCGCCACCTGGGCCCCCGCCTC
TTCGCCGCCGCCGAGCCAGCCTCCGGGCTCGCCGCGAGCGCGAGGGGCATCAT
GCCCGCCGCCGCGAGGATCTTCCCCGCGCGGATGGCCAGCACCGAGGCCGCCG
CCCCGCATGCCAAACAAGAAGATGATGCCGCGAGCCCCCAGGCGGCCGCGACTC
CAGAGCAGCAGAACAAGAAGCCCGTGGTGAGCTACTGGGGCATCGAGCCTCGGA
AGCTCGTCAAGGATGACGGCACGGAGTGGCCATGGTTCTGCTTCAGGCCGTGGG
ACACGTACCGGCCGGACACGTCCATCGAAGTGGCCAAGCACCACGAGCCCAAGG
CCCTGGCGGACAAGGTGGCCTACTTCGTGGTTCGGTCGCTGCGCGTGCCCCGGG
ACCTCTTCTTCCAGCGCCGGCACGCCAGCCATGCTCTGCTACTGGAAACGGTGGC
GGCGGTGCCTCCCATGGTGGGCGGCGTGCTGCTGCACCTGCGCTCGCTCCGCCG
CTTCGAGCACAGCGGCGGCTGGATCCGGGCGCTCATGGAGGAGGCCGAGAACGA
GCGCATGCACCTCATGACCTTCATGGAGGTGACGCAGCCGCGGTGGTGGGAGCG
CGCGCTCGTGCTCGCCGCGCAGGGCGTCTTCTTCAACGCCTACTTCGTCGGGTAC

CTCATTTCCCCCAAGTTCGCGCACCGCTTCGTCGGGTACCTCGAGGAGGAGGCCG
TGGAGTCTTATACTGAGTATCTCAAGGACCTTGAGGCCGGATTGATCGAGAACACG
CCCGCGCCGGCCATCGCCATCGACTACTGGCGCCTCCCCGCCGACGCCAGGCTC
AAGGACGTCGTCACCGCCGTGCGCGCCGACGAGGCGCATCACCGCGACGCCAAC
CACTACGCATCGGACGTCCATTACCAGGGAATGACGCTGAATCAATCGCCTGCGC
CGCTCGGGTACCACTGA
>*TaAOX1d-2DL* gene
ATGGCCGGAGCCACGCTTCTGCGCCACCTGGGCCCCCGCCTCTTCGCCGCCGCC
GAGCCAGCCTCCGGGCTCGCCGCGAGCGCGAGGGGCATCATGCCCGCCGCCGC
GAGGATCTTCCCCGCGCGGATGGCCAGCACCGAGGCCGCCGCCCCGCATGCCAA
ACAAGAAGATGATGCCGCGAGCCCCCAGGCGGCCGCGACTCCAGAGCAGCAGAA
CAAGAAGCCCGTGGTGAGCTATTGGGGCATCGAGCCTCGGAAGCTCGTCAAGGAT
GACGGCACGGAGTGGCCGTGGTTCTGCTTCAGGCCGTGGGACACGTACCGGCCG
GACACGTCCATCGACGTGACCAAGCACCACTTGCCCAAGGCCCTGGCGGACAAG
GTGGCGTACTTCGTTGTCCGATCGCTGCGCGTGCCCCGGGACCTCTTCTTCCAGC
GCCGGCACGCCAGCCACGCGCTGCTGCTGGAGACGGTGGCGGCGGTGCCGCCC
ATGGTGGGCGGCGTGCTACTTCACCTGCGCTCGCTCCGCCGCTTCGAGCACAGC
GGCGGCTGGATCCGGGCGCTCATGGAGGAGGCCGAGAACGAACGCATGCACCTC
ATGACCTTCATGGAGGTGACGCAGCCCCGGTGGTGGGAACGCGCGCTCGTGCTC
GCCGCGCAGGGCGTCTTCTTCAACGCCTACTTCGTCGGCTACCTCATCTCCCCCA
AGTTCGCGCACCGCTTCGTCGGGTACCTCGAGGAGGAGGCCGTGGAGTCCTATA
CTGAGTACCTCAAGGACCTCGAGGCCGGCTTGATCGAGAACACGCCCGCCCCGG
CCATCGCCATCGACTACTGGCGCCTCCCCGCCGACGCCAGGCTCAAGGACGTCG
TCACCGCCGTGCGCGCCGACGAGGCGCATCACCGCGACGCCAACCACTACGCAT
CGGACATCCATTACCAGGGAATGACGCTGAATCAGACGCCTGCGCCGCTCGGGTA
CCACTGA
>*put.regTaAOX-3B* gene
ATGGTTAGAAGAAGGAGGTGGCGATCAGCAGCTACTTGGGGATCGAGCAGTCGAA
GAAGCTGGTGAGTGAGGAAGGCACCGAGTGGAAGTGGTCTTGCTTCAAGGTACG
CTCTTCCCTTGCTTCCTCTGCTCTGCTTTTCCCATTTCGGTTCCATCGCCGGCGAC
GGGTCTCCAGATGGGAGCACGCGTGCTGTCGATGTGCGCTGTAATTTGATTTACTT
GTTGCACCGCCGCTGTGTGTAATTCATTAGTTGGTTTTGCTGCCGCAGCCATGGGA
GACGTACACCGCGGACATGTCGATCGATCTGACCAAGCACCACGTGCCCAACACG
ATGCTCGACAAGATCGCCTACTACACCGTCAAGTCCCTGCGCTTCCCCACCGACA
TCTTCTTCCAGGTACGCGCGCTTCCTGGTCTGGCGCCCCTGCTACATGTACGAGT
GCAAGCGTCACGTGGCCGTGAGTAGGCCAGTTCCACGCCAACAAGATGAACCTG
GGCACTGCAACTACCTATGTGAACACGTGATTCTTCTTCCTGATTTGATTTGGTGTT
GATTTGATTGATTCCTGGCGCATCTAATTCTTCTTCTCAGGTACTCTTCTGTTGATG
AACTTCCTCCACACCTGCAGTGACAAGAAAACACTAAAGAAGTGGTTCTTCATCGA
CAAGACAGTTGGTTAAGAAAATCCAGCTGAAAGAGAGACAACCATCATACCTGCCT
GAGAAAGATCCTGCCTACAGTGCATGGATGGTAAGGTTTGTTTCATGATTCAGTTTT
GGGTTATGTAGTAAGCAGTAGTCTAGTTACAATGCCTCTTCTGAAGAAAGACATGC
AGATATCCGTCTTGTGCTGCATGCCTATGAAGCGTATCTGTTGTCAATGCTATCCG

TCTTGGCACCTGTCTTCTCGCCTGGCCCTGCATGTGGTATGCTTGCTAGTTCCTTC
TACTGGCATCCCCATCTCTCACCACCATTGTGTAGATAGATTAGCAGAATCAGCTC
ACTGGAGACTACTAGAGAGCATACTGAGCTCACATTTTCAGATTTGATGTGTGCAT
AAACAGATTTTACTTAAAAGCTCCCTGTAACCTTTTGTTTCATTGGTCAATCACGATA
GCAGCGATGCCGGGGGAGCTCCCCTACACACCTATGCATTCTAATGGTCACATAC
ATGTATCTAGTTCTCCTCATAAATTTTTTGCATGCATGACAAAGCTCTAACGCGTAC
ACTGTCATGCTTACTTAGATTAGAAGCATTCTGTACAAGACAAACTACTATATGCTA
GTCTTACTTACACTCTGTGTGGGGCACTCTCATTTAATTTTCTAGCATGTTAAATTTA
ACATTCTAATATATTTCTTTGAACCAATAGTCTAGCATAACTTATTAACTTCATTTTTA
TAAAATAGATAATATATATTGAATTATTCATATTAATCTAAAATAATAACCAAACAACT
ACAGGACTGTGCATTAGTAGCATGCATGTGTGACTTTGTTTTTGGTGTGTTCTGCTT
GCAGGTCATTGTTGTGAATGCCCCACAGGAAGGCGTGGGGGATTTGGCACAAGG
CCGGTGATCGAGTCTATGCTCTCTCCTCCATGGAAAGACATATGTTCAGCTTGCTT
GAATGAACAGTGTGTGTGGAACAGATTCTTCTCTGAATTCTCTTCTTGTGGGAGTAT
GAACTTGCTTGTAGTATCTTGGAGTATCAACTTGGAGCATCTGGTTAGAGTAGAAT
CTTGCAACACATTCGATTACTCCAGTACTGATAGTTACAATAGCATCTTGAAAAATG
GAGTAGTTAGAGTAGCATCTTTGATTAACTAAATTGATGATTACTGCTGTTAAATTG
GGACTTGCAAGCAGTAGGTCCCTCTTCTTATCTCTTCTCTCTTGCAGTAGTTGTTGT
AGTAGTAGTTTCTTGCAGTAGTTTAGTGTACGATTTGTCTTTGGTGGAGTATTGCTT
GAACTGTGTTGTATTTCCTATGACTAAATAGTGAATCTAGTTACAACAAAGCATGCC
AGTACTTGTCTTTGGTGGAGTAGTTTGACAGTGAGGATCAGTGATGTTCCCAATTA
ATTAGGTGTATTTTTGATAGTTAGAGTAGCATGTTGGAGTAGGGCAGTCCACATTTG
TCTACATTAGACCAGATTATTCTGATTAGTCTACATTTCATATTATTCTTGGGGCACT
TTACTATAGCAGCAGTACTGTACTGTAGCAGCAACATGAGTAAACTAAATACTCCTT
CATATGCTTGGTTCCTTCTTATTCTTCCTCAAATTGTGAAAATGGATTCATTCCTCCT
TATGCACTGCACTGTACTGTAGTATAGGTCATCTTTGAATTAATACTCCAAAATTTAT
CAGTATTGGAGGATTGATTGTTTCCAAATCAATACTCCTGGGCATCATTCATGGCTG
TCTAGTAGTCGACATACTTCTGACTCCATTTAGTGTATGCAGGTAGCAACAACTTAA
GTGTAATGGAGTTGTTTTGTTACTCTATGCCTACTTCGTTAGAGTTGGCATCATCTC
CTTGTCGTTTATAGCATAAACGCCTTCACTATTCTTGCTCTGAACCTCTGATCCTGA
TGCTGATTGTATTTGTCCTGATGGAATTACATGCAACATGGTGACTCTAGTGCAGG
CAGCATTGGCCATCCTCTTCGAGGCTGTGTTTAGGTGATAACTCGGGTAGCCATGA
CACTACTCTAGCTGATTAGTACCCAGTGGCATGTTTGTAATATTTATATGCATTTTAC
TTGTAGAAATAATGCTCATGGTATATTTATAAGTCCTGCAGCGCATGCTCTTTTTCG
GTCTGTACATGATCTTGATATGGTGTTTTGGCACCTGTGATATTAGATTGCACTTCT
TTATGTGATTTACTCATGTTGGTACTACTTGTTAAGTGATTCCTCCGTAACTTGTCTT
TTTCGGAACAGTTTGCTTGGTCTTTCATATCTGACTATGTAATGTAGAAGCTCTACT
TTATCCTAATAGTTGCATGTAGCATTGTGCTACTATCTGTGTGATGCTAAACAATAT
GCTCTTAATCTGTTGATATATTCTCAAGTGTTGTTCACCATTTACATTGTTTTTACAT
TTGCTAGTAGTGTCTGGTCTAGATAAGCAAACAAGGTATGATCTATGCAGTTGTTCA
TCTGAATTATTTCTTTCTAGGCGGTGTAGGACGAGCTGCAGCAGGACAACCTGGA
GCGGAAGAACTTCGAGGGCAAGATCAAGGAGAACCAGGAGACGATCACCGGTTA

CCTCATCCTCGTCGCCATGCTTCGCTTCTTTGGCAGTCCCCTCTTTGGCCCCGACC
AACTTACTTCTGTCGCTGGCTAG
>*put.regTaAOX-6BL* gene
ATGGAGGCGTTTCTCGGCGGCCAGATGAGCTCTCGGATGGCCGGATCGGTCCTC
CTCCGCCGCGCCGGCGCTGGCGCTAGCCGCCTCTTCTCCACCACCACGATGTCC
CCAGGGGCCAGGACCTTCCTCGCCGGCGGCAAGGGCACGTGGGTGCGGATGAT
GTCCACTTCTGCGGCCTCGCAGGTCAAGGACGAGGCGGCTAAGGTGGTCAAGGC
GGAGGCGGCCAAGGGCGATGGTTAGAAGAAGAAGGTGGCGATCAGCAGCTACTA
GGGGATCGAGCAGTCGAAGAAGCTGGTGCGTGAGGACGGCACCTAGTGGAAGTG
GTCTTGCTTCAGGGTATGCTCTTCCCTTTCTTCCTCTGCTCTGCTTTTCCCGTTTCG
GTTCCGTCGCTGGCGACGGGTCGCTAGATCAGAGCGCGCATGCAGTCGATGTGC
GCTGTTATTTTATTTACTTGTTGTGCCGCTGCTGTGTGTGATTCATTAGTTGGTTTC
GCTACCGCGGCCATGGGAGACGTACACCATGGACATGTCGATCGATCTGACCAAG
CACCACGTGCCCAACACGATGCTTGACAAGATAGCCTACTACACCGACAAGTCCC
TGCACTTCCCCACCGACATCTTCTTCCAGGTACGTGCGCTTCCTGGTCTGGCGCC
CCTGCTACGTGCAGGAGTGCAAGCGTCACGTGGCCGTGAGCAGGCCAGTTCCAC
GCCAACAAGATGAACCTGGGCATTGCAACTACCTATGTGAACACGCGATTCTTCTT
CCTGATGCATCTAATTCTTCTTCTCATGTACTCTTCTGTTGATGAACTTCCTCCACA
GCTGCAGTGACAAGTAAACACTGAAGAAGTGGTTCTTCATCGACAAGACAGTTGGC
TAAGAAAATCCAGCTGAAAGAAAGTCAACCATCGTACCTGCCTGAGAAAGATCCTG
CCTACAGTGCATGATGGTAAGATTTGTTTCATGATTTAGTTTTGGGTTACGTAGTAA
TCAATAGTCTAGTTACAATGCCTCTTCTGAAGAAAGACATGCAGATATCCGTCTTGT
GTTGCATGCCTATTGAAGTGTATCTGTTGTCAATGCTATCCGTCTTGGCACTTGTCT
TCTCGCCTGGCCCTGCATGTGGTATGTTTGCCAGTTCCTTCTACTGGCATCCCCAT
CTCTCACCACCATTGCGTAGATAGATTAGCAGGATCAGCTCACTGGAGACTAGTAG
AAAGCATATTGAGCTCACATTTTTAGATATGCTGTGTGCATAAACAGATTTTACTTAA
AAGCTCCCTGTAACCTTTTGTTTCATTGGTCAATCATGATAGCAGCGATGCCGGGG
GAGCTCCCCCTACACACCTATGCATTCTAATGGTCACATACATGTAACTAGTTCTCC
TCATAAAATATTTGCATGGATGACAAAGCTCTAACGCGTACACTGTCATGCTTACTT
AGATTAGAAGCATTTTGTACAAGACAAACTACTATATGCTAGTCTTACTTACACTCT
GTGTGGGGCACTCTCCTTTAATTTTCTAGCATGTTAAATTTAACATTCTAATATATTT
CTTTGAAACAACATCTAGCATAACTTATTAACTTCATTTTTTAAACAGATAATACATA
TTGAATTATTCCTATTAATCTAAAATAATAACCAAACAACTACAGGACTGTGCATTAG
TAGCATGCGTGTGTGACATCGTTTTTGGTGTGTTCTGCTTGCAGGTCATTGTTGTG
AAGGCCCCACAGGAAGGCGTGGGGGATTTGGCACAAGGCCGGTGATCGAGTCTA
TGCTCTCTCCTCCATGGAAAGACCTAAGGTCAGCTTGCTTGAATGAACAGTGTGTG
TGGAACAGATTCTTGTCTGAACTCTCTTCTTGTGGGAGTATGAACTTGCTTGCAGTA
TCTTGGAGTATCAACTTGGAGCATCTGGTTAGAGTAGCATCTTGCAACACATTCAAT
TACTCCAGTACTGATAGTTATAGTAGAATCTTGCAAAATGGAGTAGTTAGAGTAGCA
TCTTCGATTAACTAAATTGATGATTACTGCTGTTAAGTTGGGACTTGCAAGCAGTAG
TTCCCTCTTCTTATCTCTTCTCTCTTGCAGTAGTTGTTGTAGTAGTAGTTGCTTGCA
GTAGTTTAGTGTATGATTTGTCTTTGGTGGAGTATTTCTTGAACTATGTTGTATTTCC
TGTGACTAAATAGTGAATCTAGTTACAACAAAGCATGCCAGTACTTGTCTTTGGTGG

AGTAGTTTGACAGTGAGGATCAGTGATGTTCCCATTTGTCTACATTAGACGAGATTA
TTCCGATTTGTCCACATTTCATATTATTCCTGGAGCATTGTACTATAGCAGCAGTAC
TGTACTGTAGCAGCAACATGAGTAAACTGAATACTCTTTCATATGCTTGGTTCCTTC
TTATTCTTCCTCAGATTGTGAAAATGGATTCATTCCTTCTTATACACTGCACTGTACT
ATAGTATAGGTCATCTTCGAATTAATACTCCAAAATTTATCAGTATTGGAGGATTGA
TGTTTCCAAATCAATACTCCTGGGCATCATTCATGGCTGTCTAGTAGTCGACATACT
TCTGGTTCCATTTAATGTATGCAGGTAGCAGCAGCTTAAGTGTAATGGAGTTGTTTT
GTTACTCTATGCCTACTTCGTTAGAGTTAGCATCATCTCCTTGTCGTTTATAGCACA
AACGCCTTCACTATTCTTGCTCTGAACCTCTGATCCTGATACTGATTGTATTTGTCT
TGATGGAATTATAGGCAACATGGTGACTCTAGTGCAGGCAGCGTTGGCCATCCTCT
TCGAGGTTGTGTTTAGGTGATTACTCGGGTAGCCATGACACTACTCTGGCTGATTA
GTACCCAGTGGCATGTTTGTAATATTTATAGGCATTTTACTTGTAGAAATAATGCTC
ATGGTATATTTATATGTCCTGCAGCACATGCTCTTTTTCAATCTGTACGGAATCTTG
ATATGGTGTTTTGGTAGCTGTGATATTAGATTGCACTTCTTTATGTGATTTACCCATC
TTGGTACTGCTTGTTAAGTGATTATCTTTATGCCCTTGCTACTAGTAGAATGGTATA
TACATGATTGCTCTGTAACTTGTCTTTTTCGGAACAGTTTGCTTGGTCTTTCGTATCT
GATTATGTAATGTAGAAGCTCTACTTTATCCTAACAGTTGCATGTAGCATTGTGCTA
CTATCTGTGTGATGCTAAACAATATGCTCTTAATCTATTGATATATTCTCAAGTGTTG
TTTACCATTTACATTGTTTTTACATTTGCTATATTCACAAATAGTAGGATTGTCTAGT
CTAAATAAGCAAACAAGGTATGATCTATGCAATTGTTCATCTGAATTGTTTGTTTCTA
GGCGGTGTAGGACGAGCTGCAGCAGGACAACCTGGAGCGGAAGAACTTCGAGGG
CAAGATCAAGGAGAACCAGGAGACGATCACCGGTTACCTCATCCTCGCCGGCATG
CTTGGCTCCTTTGACAGACCCCTCTTTGGCTCCGACCAACTTACTTTTGTCGCTGG
CTAG
>*put.TaAOX1e-3DS* gene
ATGGCTGCCACGTTAAAGAAAGGGGAGGAGGAGGCGGCGAGCTACTGGGGCGTG
GCGCCGGCGAGGCTCGTCAAGGAGGACGGCACCGAGTGGAAGTGGTCGTGCTTC
AGGGTACGTACTCGCCCGTGAGCTTGTTGCCTAATTATTCATGACGTCACTTATTAT
GGCTTTGAGCTCAAACTCCACGTGAGCTTGTCGACTGAGCTGCATGCATGCATGC
ATGCATGGGTGAATGGCGCATCTGCAGCCGTGGGATGCGTACGAGGCCGACGTG
TCCATCGTTCTGACGAAGCACCACCGGCCGGCCACGTTCGGGGACAAGGTGGCC
TTGTGGACGGTCAAGGCGATACGCTGGCCCACGGACCTCTTCTTCCAGGTAGTAC
TTCCTCATTCTTCGTTGTGATTTTTTTTTTGACAATCACAAGACTTTATTTCTCAGATA
ATAGCCAAGTCTGTTACAACGAGTTGAGAAATAAAATCAGGAGGGGTAGCATCCCA
CATAGAACTAAACTTTGAATTAAAACTGGATTTAGCCAGACAATCTGCTATGGCGTT
TGCTTCACGTGTGTAGTTGTCGAAAAAAAGAGAAGCATAGTTTGCCTTCAATTCATT
GCACTCCATGACAATCGCTGCTTCCTGGCCCAGATAACTTTCAAATTTTTCCACCG
CTTCTAGCGCCAATTGGCTACCAGACTCCACAATTACCTTGGAGCATCCAATATGG
GCAGCTAAGTATAAACCATTCCGCATAGCAAGAATCTCTGCTGAAACAACACTGTT
AATATGTGGAAGAAACCATGATGCAGCAGCCAAGAACTGGCCACTATCATCCCTTG
CAATGGCACCCGTCGCCCCTGAGAGAATATCCTCGTGAAAAGAGGCATCCACATT
GATCTTCACCTTCCCCCTGGTTGGTTTCTTCCACATTTGATCTCTCTTTCTAACTGG
TTGGTTGGGTGTTGCTGCTCGTACATAGTTCGTCGCTAGGATCTTAATTGAAATAG

CTGAATGGTGTGCATCTGAAATCGTTTCCCCCTTTGTAAACTGGCGACGTTGCCAC
CAAATATACCATGCAGCCACCGTAATCAACTCAGCCGCTGGAAGATCACCAATTGA
TGAGTGGTTTCGCAATAAAATCTCCATGGTAACTGATCCCGAGCGGTCCTCCATTG
CTGCTTGGTTGATCACCTTATTCATGCCTAATTCTTTCCACACTTCCATAGCACGGC
AACACAAGAATAAGCAATGCTGAATATCTTCAAACCCAACTTTGCAAACCGAGCATT
GACCAGATACCGGGATATGACGTTTCGCGAGGACTCCACAGCATGGCAGAACGCC
CCTGAGCACCTTCCAAGCGAAATGCTTAATCTTGCCAGGAACTTAGAGTTTCCACG
AATGTTTCCAAATGCCATTTACTACAGACCCTCCTTGTGCATCAGCAGGAGAAGCG
CGCGAGCCAAACTGATGATTGAACTCTGTATGGTATGCTGAACGGACTGAGAAGG
TTCCTGACCGTGTAAACTGCCATGCCACAAAATCGTCAACAATACTCACATTAAGA
GGGATCCTTAGTATCCTTTCCATATCGACTGGATTAAACACGGACTGCAATAAGGC
TTCATCCCATGTGCCCGGGTGCGGGTCTATGAGCTCTTCAACTTTGGACAACATAA
TCTGCCCCCTCGGTGTTGGGGAATGTAGTAATTTCAAAAAAATTCCTACGCACACA
CAGGATCATGGTGATGCATAGCAACGAGAGGGGAGAGTGTGTCCACGTACCCTCG
TAGACCGAAAGCGGAAGCGTTAGCACAACGCGGTTGATGTAGTTGTACGTCTTCA
CGATCCGACCGATCCAAGTATCGAACGCACGGCACCTCCGAGTTCAGCACACGTT
CAGCTCGATGACGTCCCACGAACTCCGATCCAGTAGAGCTTCGAGGGAGAGTTCT
GTCAGCACGACGGGCGTGATGACGGTGATGATGATGCTACCGACGCAGGGCTTC
GCCTAAGCACCGCTACGATATGATCGAGGTGGATTATGGTGGAGGGGGGCACCG
CACACGGCTGGGAGAGATCAACAGATCAACTTGTGTGTCTGGAGGTGCCCCCTTG
CCCCCGTATATAAAGAATGGAGGAGGGGAGGCCGGCCAGCCCCTAGGGCGCGCC
AGAAGTGTGGAGTCCTACTAGGACTCCCTAGTCCTAGTAGGATTCCTCCTCCCACA
TGGAATAGGAAAGAGGGAAGGGAAAAGGAGAAGGAAGGAAGGGGGTGCCCCCCT
CCCTAGTCCAATTCGGACCAGACCATGGGGAGGGGTGCGGCCACCTTTTGAATCC
TTTCTCTCCTTTCCCGTATGGCCCATTAAGGCCCAATACGAATTCCCGTAACTCTCC
GGTACTCCGAAAAATACCCGAATCACTCGGAACCTTTCCGATGTCCGAATATAGTC
GTCCAATATATCGATCTTTACGTCTCGACAATTTTGAGACTCCTCGTCATGCCCCCG
ATCTCATCCGGGACTCCGAACTACCTTCGGTACATCAAAACACAAAAACTCATAATA
CAATCATCATCGAACTTTAAGCGTGCGGACCCTATGGGTTCGAGAACTATGTAGAC
ATGACCGAGACACATCTCCGGTCAATAACCAATAGCGGAACCTGGATGCTCATATT
GGCTCCTACATATTCTACGAAGATCTTTATCGGTCAAACCGCATAACAACATACGTT
GTTCCCTTTGTCATCGGTATGTTACTTGCCCGAGATTTGATCGTCGGTATCTCAATA
CCCAGTTCAATCTCATTACCGGCAAGTCTCTTTACTCATTCCGTAATGCATCATCTC
GCAACTAACTCATTAGTCACATTGCTTGCAAGGCTTATAGTGATGTGCATTACCGA
GAGGGCCCAGAGATACCTCTCCAACAATCGGAGTGACAAATCCTAATCTCGAAATA
CGCCAACTCAACAAGTACCTTCGGAGACACCTGTAGAGCACCTTTATAATCACCCA
GTTACGTTGTGATGTTTGGTAGCACACAAAGTGTTCCTCCGGTAAACGGGAGTTGC
ATAATCTCATAGTCATAGGAACATGTATAAGTCATGAAGAAAGCAATAGTAACATAC
TAAACGATCAAGTGCTAAGCTAACGGAATGGGTCAAGTCAATCACATCATTCTCCT
AATGATGTGATCCCATTAATCAAATGACAACTCATGTCTATGGCTAGGAAACTCAAC
CATCTTTGNNNNNNNNNNNNNNNNNNNNNNNNNNNNNNNNNNNNNNNNNNNNNNNN
NNNNNNNNNNNNNNNNNNNNNNNNNNNNNNNNNNNNNNNNNNNNNNNNNNNNNNNNNNNN
NNNNNNNNNNNNNNNNNNNNNNNNNNNNNNNNNNNNNNNNNNNNNNNNNNNNNNNNNNNN

NNNNNNNNNNNNNNNNNNNNNNNNNNNNNNNNNNNNNNNNNNNNNNNNNNNNNN
NNNNNNNNNNNNNNNNNNNNNNNNNNNNNNNNNNNNNNNNNNNNNNNNNNNNNN
NNNNNNNNNNNNNNNNNNNNNNNNNNNNNNNNNNNNNNNNNNNNNNNNNNNNNN
NNNNNNNNNNNNNNNNNNNNNNNNNNNNNNNNNNNNNNNNNNNNNNNNNNNNNN
NNNNNNNNNNNNNNNNNNNNNNNNNNNNNNNNNNNNNNNNNNNNNNNNNNNNNN
NNNNNNNNNNNNNNNNNNNNNNNNNNNNNNNNNNNNNNNNNNNNNNNNNNNNNN
NNNNNNNNNNNNNNNNNNNNNNNNNNNNNNNNNNNNNNNNNNNNNNNNNNNNNN
NNNNNNNNNNNNNNNNNNNNNNNNNNNNNNNNNNNNNNNNNNNNNNNNNNNNNN
NNNNNNNNNNNNNNNNNNNNNNNNNNNNNNNNNNNNNNNNNNNNNNNNNNNNNN
NNNNNNNNNNNNNNNNNNNNNNNNNNNNNNNNNNNNNNNNNNNNNNNNNNNNNN
NNNNNNNNNNNNNNNNNNNNNNNNNNNNNNNNNNNNNNNNNNNNNNNNNNNNNN
NNNNNNNNNNNNNNNNNNNNNNNNNNNNNNNNNNNNNNNNNNNNNNNNNNNNNN
NNNNNNNNNNNNNNNNNNNNNNNNNNNNNNNNNNNNNNNNNNNNNNNNNNNNNN
NNNNNNNNNNNNNNNNNNNNNNNNNNNNNNNNNNNNNNNNNNNNNNNNNNNNNN
NNNNNNNNNNNNNNNNNNNNNNNNNNNNNNNNNNNNNNNNNNNNNNNNNNNNNN
NNNNNNNNNNNNNNNNNNNNNNNNNNNNNNNNNNNNNNNNNNNNNNNNNNNNNN
NNNNNNNNNNNNNNNNNNNNNNNNNNNNNNNNNNNNNNNNNNNNNNNNNNNNNN
NNNNNNNNNNNNNNNNNNNNNNNNNNNNNNNNNNNNNNNNNNNNNNNNNNNNNN
NNNNNNNNNNNNNNNNNNNNNNNNNNNNNNNNNNNNNNNNNNNNNNNNNNNNNN
NNNNNNNNNNNNNNNNNNNNNNNNNNNNNNNNNNNNNNNNNNNNNNNNNNNNNN
NNNNNNNNNNNNNNNNNNNNNNNNNNNNNNNNNNNNNNNNNNNNNNNNNNNNNN
NNNNNNNNNNNNNNNNNNNNNNNNNNNNNNNNNNNNNNNNNNNNNNNNNNNNNN
NNNNNNNNNNNNNNNNNNNNNNNNNNNNNNNNNNNNNNNNNNNNNNNNNNNNNN
NNNNNNNNNNNNNNNNNNNNNNNNNNNNNNNNNNNNNNNNNNNNNNNNNNNNNN
NNNNNNNNNNNNNNNNNNNNNNNNNNNNNNNNNNNNNNNNNNNNNNNNNNNNNN
NNNNNNNNNNNNNNNNNNNNNNNNNNNNNNNNNNNNNNNNNNNNNNNNNNNNNN
NNNNNAATCACATCATTCTCCTAATGATGTGATCCCATTAATCAAATGACAACTCAT
GTCTATGGCTAGGAAACTCAACCATCTTTGATCAACGAGCTAGTCAAGTAGAGGCA
TACTAGTGACACTATGTTTGTCTATGTATTCACACATGTATCAAGTTTCCGGTTAATA
CAATTCTAGCATGAATAATAAACATTTATCATGGAATAAGGAAATAAATAATGACTTT
ATTATTGCCTCTAGGGCATATTTCCTTCAGTCTCCCACTTGCACTAGAGTCAATAAT
CTAGTCCACATCGCCATGTGATTTAACACCAATAGTTCACATCACCATGTGGTTAAC
ACCCATAGTTCACATCGATATGTGACCAACACCCAAAGGGTTTACTAGAGTCAATA
ATCTAGTTCACATATTAACACCCATAGAGTACTATGGTCTGATCATGTTTTGCTTGT
GAGATAATTTTAGTCAACGGGTCTGTCACATTCAGATCCGTAAGTATTTTGCAAATT
TCTATGTCAATAATGCTCTGCACGGAGCTACTCTAGCTAATTGCTCGCGCTTTTAAT
ATGTATCCAGATTGAGACTTAGAGTATGAATAACTGTATTGCAATAAACATGATCAA
ATCATATTCATGCTCAACGCAAACACGAAATAACACTTATTTAGGTTCAACACTAAT
CCCGAAAGTATAGGGAGTGTGCGATGATGATCATATCAATCTTGGAACCACTTCCA
ACACACATCCTCACTTCACCTTTAACTAGTCTCTGTTCATTCTGCAACTCCCGTTTT
GAGTTACTACTTTTAGCAACTGAACCAGTATCAAATACCGAGGGGTTGCTATAAAC
ACTAGTAAAGTACACATCAATATAACATGTATATCAAATATACTTATGTTCACTTTGC
CATCCTTCTTATCCGCCAATTACTTGGGGTAGTTCCGCTTCCAGTGACCAGTCCCT

TTGCAGTAGATGCACTTAGTCTCAGGCTTAGGTCCAGACTTGGGCTTCTTCACTCG
AGCAGCAACTTGCTTGCCGTTTTTCTTGAAGTTCCCCTTCTTCCCTTTGCCCCTTTT
CTTGAAACTAGTGGTCTTGTTAATCATCAACACTTGATGCTCTTTCTTAATTTCTACC
TTCATCGATTTCATTATCATGAAAAGCTCGGGAATCGTTTTCGTCATCCCTTGCATA
CTATAGTTCATCACGAAGTTCTACTAACTTGGTGATGGTGACTAGAGAATTCTGTCA
ATCACTATTNNNNNNNNNNNNNNNNNNNNNNNNNNNNNNNNNNNNNNNNNNNNNNNNNN
NNNNNNNNNNNNNNNNNNNNNNNNNNNNNNNNNNNNNNNNNNNNNNNNNNNNNNNNNNNN
NNNNNNNNNNNNNNNNNNNNNNNNNNNNNNNNNNNNNNNNNNNNNNNNNNNNNNNNNNNN
NNNNNNNNNNNNNNNNNNNNNNNNNNNNNNNNNNNNNNNNNNNNNNNNNNNNNNNNNNNN
NNNNNNNNNNNNNNNNNNNNNNNNNNNNNNNNNNNNNNNNNNNNNNNNNNNNNNNNNNNN
NNNNNNNNNNNNNNNNNNNNNNNNNNNNNNNNNNNNNNNNNNNNNNNNNNNNNNNNNNNN
NNNNNNNNNNNNNNNNNNNNNNNNNNNNNNNNNNNNNNNNNNNNNNNNNNNNNNNNNNNN
NNNNNNNNNNNNNNNNNNNNNNNNNNNNNNNNNNNNNNNNNNNNNNNNNNNNNNNNNNNN
NNNNNNNNNNNNNNNNNNNNNNNNNNNNNNNNNNNNNNNNNNNNNNNNNNNNNNNNNNNN
NNNNNNNNNNNNNNNNNNNNNNNNNNNNNNNNNNNNNNNNNNNNNNNNNNNNNNNNNNNN
NNNNNNNNNNNNNNNNNNNNNNNNNNNNNNNNNNNNNNNNNNNNNNNNNNNNNNNNNNNN
NNNNNNNNNNNNNNNNNNNNNNNNNNNNNNNNNNNNNNNNNNNNNNNNNNNNNNNNNNNN
NNNNNNNNNNNNNNNNNNNNNNNNNNNNNNNNNNNNNNNNNNNNNNNNNNNNNNNNNNNN
NNNNNNNNNNNNNNNNNNNNNNNNNNNNNNNNNNNNNNNNNNNNNNNNNNNNNNNNNNNN
NNNNNNNNNNNNNNNNNNNNNNNNNNNNNNNNNNNNNNNNNNNNNNNNNNATCGCCGCCT
CGCCCCCGCCACTTGACGCGCTCGCCGGTGCCGCCCCTCCCGGACTCCTCACCC
TCTTCCTCAACAGCCACCTCGTCGCCGCGCCCGTCGCCGGACCGCCTCCCCGTCA
CCGTCGTCATCTACCTCGTCCTCCTCCTCGAGCACACTGCCGTTCCCCTACATCGC
AAGTGAGGCCCGGCCTCTCCTCCTCTCCCCCCTGTCTCCCGAGCTCGCTGGAGCT
CGTAACCCCCACCGCTGCATGCACTCCCTCGCACCACCGCGCGCGCCCCGGCGC
CCCTGAACGCGCCCACGGTTGCCCCCCTGCTACCCGTCTCCCTCGCTCACCCCGC
ACGCCGCGATCCCTGCCCCTGTTTTCCCCTGCTCTGCTCCGCCTCCGGCCGAGAC
CAAGTCCGCATGGCCCGCCTCCCCTGCTCCTGTGGACGCCGAGCCTCCCCACCG
GCCCCCGCGTGAGCCCTCGCTGTCGCTGCGCCACCGCAACCGCCGTGCGCACGC
ACACCCGCACTTCGTCGCCCACCGCTACCTTCCCCCTACCACGTCGCGCTCGTCG
GCGTCCCTGGTGTTGGCGCCGTCGTCCCGTGCCCCGCCTTCGCCTCTGGCCCCG
CAGGCCACCGCCACGTCGACGGCCAGTTCCGGCACCCCGCGGCGCCCCGGGTC
TGCCCTTCGGGTGCAAAAGCCCGGGTGCCCGCTGCCCGTTTCGCCCGCTAGGCC
CCCAGGGGCCTATGACAAAGGGGCCCCGCGCCCCAGAACGGTTTAATAAAAAAGA
ATTAAATAAAAATAAATAATAATAATATGATTAATCAATTAAGATAATTAATTAAATTA
ATTAATCCCTTTAATTAATCTAAATTAAGCCTAATTAATTAACCTAATCACTTTAATTA
ATTTAACTAAACCTGTTTAGTTAACTAAGTCAATGACCAGTGGGACCCACACGTCA
GTGACCCAGTCAACACCTCTGTTGACTGCTGTGTCATGCTACCGTCATGATGACGT
CAGCAAATACTATTCTGGATAATGTTGAATTAAATTAATTAAACAAATTCTAAAAATG
ATTTAAAACTTTAGTAAATCATATAAAATAATCCGTAACTCGGATGAAAATACTTTCT
ACATGAAAGTTGCTCAGAACGACGAGACAAATCCGGATATGCAGCCCGTTCGTCC
GCCACACATCCCTAACATATCGAACTCGCAACTTTCCCCCTCCGGTTCATCTGTCC
GAAAACGCGAAACACCGGGGATACTTTCCCGGATGTTTTCCCCCTTCGCCTGTATC

ACCTCTTACCGCGTTAGGGCACACCTGACACCGCTGTTTGCTTTGTCATGCATCGT
TATGCATTTGTTTGCATTGTATTCATTGTTTCTTCCCCCTCTTCTCTCCGGTAGACTA
TGAGACCGACGCTGCTACTGGTGCCCCGACCGACTACGGTGTTGACGACCCCTCC
TTCTCGGCTGAGCTTCCAGGCAAGCCCCCCCTTGATCACCAGATATCGTCTATTCT
TCTCTATACTGCTTGCATTAGAGTAGTGTAGCATGTTACTGCTTTCAGTTAATCCTA
TTCTGATGCATAGCCTGTCATTGTTGCTACAGTTGTTACCCTTACCTGCTATCCTAC
TGCTTAGTATATGATGCTAGTGTTCCATCAGTGGCCCTACACTCTTGTCCATCTGCC
ATGCTATACTACTGGGCCGTGATCACTTCGGGAGGTGATCACGGGTATATACTATA
TACTTTATATACATGACACATGTGGTGACTAAAGTCGGGTCAGCTCGTTGAGTACC
CGCAAGTGATTTTGATGAGGGGGCTGAAAGGACAGGTGGCTCCNNNNNNNNNNN
NNNNNNNNNNNNNNNNNNNNNNNNNNNNNNNNNNNNNNNNNNNNNNNNNNNNNNNNNNNN
NNNNNNNNNNNNNNNNNNNNNNNNNNNNNNNNNNNNNNNNNNNNNNNNNNNNNNNNNNNN
NNNNNNNNNNNNNNNNNNNNNNNNNNNNNNNNNNNNNNNNNNNNNNNNNNNNNNNNNNNN
NNNNNNNNNNNNNNNNNNNNNNNNNNNNNNNNNNNNNNNNNNNNNNNNNNNNNNNNNNNN
NNNNNNNNNNNNNNNNNNNNNNNNNNNNNNNNNNNNNNNNNNNNNNNNNNNNNNNNNNNN
NNNNNNNNNNNNNNNNNNNNNNNNNNNNNNNNNNNNNNNNNNNNNNNNNNNNNNNNNNNN
NNNNNNNNNNNNNNNNNNNNNNNNNNNNNNNNNNNNNNNNNNNNNNNNNNNNNNNNNNNN
NNNNNNNNNNNNNNNNNNNNNNNNNNNNNNNNNNNNNNNNNNNNNNNNNNNNNNNNNNNN
NNNNNNNNNNNNNNNNNNNNNNNNNNNNNNNNNNNNNNNNNNNNNNNNNNNNNNNNNNNN
NNNNNNNNNNNNNNNNNNNNNNNNNNNNNNNNNNNNNNNNNNNNNNNNNNNNNNNNNNNN
NNNNNNNNNNNNNNNNNNNNNNNNNNNNNNNNNNNNNNNNNNNNNNNNNNNNNNNNNNNN
NNNNNNNNNNNNNNNNNNNNNNNNNNNNNNNNNNNNNNNNNNNNNNNNNNNNNNNNNNNN
NNNNNNNNNNNNNNNNNNNNNNNNNNNNNNNNNNNNNNNNNNNNNNNNNNNTGCGATGC
TACTTGGTGAACTTACCATCTACTCTCTTCTACATGCTGCAAGATGGAGGTGGCCA
GAAGCGTAGTCTTCGACAGGATTAGCTATCCCCCTCTTATTCTGGCATTCTGCAGT
TCAGTCCATCGATATGGCCCTTTACACATATACCCATGCATATGTAGTGTAGCTCCT
TGCTTGCGAGTACTTTGGATGACTAGTCACGGTTGCTTTTCTCCCTCTTTTCCCCTT
TCTATACCTGGTTGTGGCAACCAGCTGCTGGAGTCCAGGAGCTAGAGATCCCGAG
GATGATTCTACATGGAGTTCGGCTTCGAGGAGTAGTTAGGAGGTCCCAGGCAGGA
GGCCTTGCCTTTTCGATCGTTGCTACTTTTGTGCTAGCCTTCTTGAGGCAAACTTGT
TTAACTTATGTCTGTACTCAGATATTGTTGCTTCCGCTGACTCGTCTATGATCGAGC
ACTTGTATTCGAGCCCTCGAGGCCCCTGGCTTGTATTATGATGCTTGTATGACTTAT
TTATGTTTTAGAGTTGTGTTGTGATATCTTCCCGTGAGTCCCTGATCTTGATGTTAC
ACGTTTGCGTGCATGATTAGTGTACGTTTGAATCGGGGGCGTCACAGCTACCGCC
GAACTAAGCAAAATAAGATGTATAAAAGATAAACATCACATGCAATCATAATATGTG
ACATGATATGACCATCATCATCTTGTGCCTTTGATCTCCATCTCCAAAGCATCGTCA
TGATCTCCATCGTCACCGGCATGACACCATGATCTCCATCATCTTGATCTATATCAA
TGTGTCGTCGCATGGTCGTCTCGCCAACTATTGCTCTTGCAACTATTGCTATCGCA
TAGCGATAAAGTAAAGCAATTATTTGGCGCTTGCATCTTATGCAATAGAGAGACAA
CCATAAGGCTTTTGCAGTTGCCGATAACTTCAACAAAACATGATAATCTCATACAAC
AACTTATACCTCATCACGTCTTGACCATATCACATCACAACATGCCCTGCAAAAACA
AGTTAGACGTCCTCTACTTTGTTGTTGCAAGTTTTACGTGGCTGCTACGGGCTGGG
CAAGAACCGTTCTTACCTACGCATCAAAACCACAACGATAGTTCGTCAAGTTAGTG

TTGTTTTAACCTTCTCAAGGACCGGGCATAGCCACACTCGGTTCAACTAAAGTTGG
AGAAACTGACACCCACCAGCCACCTGTATGCAAAGCACGTCGGTAGAACCAGTCT
CGCGTAAGCGTACGCGTAATGTCGGTCCGGGCCGCTTCATCCAACAATACCGCCG
AACCAAAGTATGACATGCTGGTAAGCAGTATGACTTGTATCGCCCACAACTCACTT
GTGTTCTACTCGTGCATATAACATCTACGCATAAAACCGGGCTCGGATGCCACTGT
TGGGGAACGTAGTAATTTCAAAAAAATTCCTACGCACACACACAGGATCATGGTGA
TGCATAGCAACGAGAGGGGAGAGTGTGTCCACGTACCCTCGTAGACCGAAAGTGG
AAGCGTTAGCACAACGCGGTTGATGTAGTCGTACGTCTTCACGATCCGACCGATC
CAAGTACCGAACGCACGGCACCTCCGAGTTCAGCACACGTTCAGCTCGATGACGT
CCCACGAACTCCGATCCAGTAGAGCATCGAGGGAGAGTTCCGTCAGCACGACGG
CGTGATGACGGTGATGATGATGCTACCGACGCAGGGCTTCGCCTAAGCACCGCTA
CGATATGATCGAGGTGGATTATGGTGGAGGGGGGCACCGCACACGGCTGGGAGA
GATCAACAGATCAACTTGTGTGTCTAGAGGTGCCCCCTTGCCCCCGTATATAGAGG
ATGGAGGAGGGGAGGCCGGCCAGCCCCTAGGGCGCGCCAGAAGTGTGGAGTCC
TACTAGGACTCCCTAGTCCTAGTAGGATTCCTCCTCCCACATGGAATAGGAAAGAG
GGAAGGGAAAAGGAGAAGGAAGGAAGGGGGCGCCCCCCTCCCTAGTCCAATTCG
GACCAGACCATGGGGAGGGGTGCGGCCACCTTTTGAGGCCTTTCTCTCCTTTCCC
GTATGGCCCATTAAGGCCCAATACGAATTCCCGTAACTCTCCGGTACTCCGAAAAA
TACCCGAATCACTCGGAATCTTTCCGATGTCTGAATATAGTCGTCCAATATATCGAT
CTTTACGTCTCGACAATTTCAAGAATCCTCGTCATGTCCCCGATCTCATCCGGACT
CCGAACTACCTTCGGTACATCAAAACACAAAAACTCATAATACAATCATCATCGAAC
TTTAAGCGTGCGGACCCTACGGGTTCGAGAACTATGTAGACATGACCGAGACACA
TCTCCGGTCAATAACCAATAACGGAACCTGGATGCTCATAATGGCTCCTACGTATT
CTACGAAGATCTTTATCGGTCAAACCGCATAACAACATACGTTGTTCCCTTTGTCAT
CGGTATATTACTTGCCCGAGATTTGATCGTCGGTATCTCAATACCTAGTTCAATCTC
GTTACCGGCAAGTCTCTTTACTCGTTCCGTAATGCATCATCTCGCAACTAACTCATT
AGTCACATTGCTTGCAAGGCTTATAGTGATGTGCATTACCGAGAGGGCCCAGAGAT
ACCTCTCCGACAATCGGAGTGACAAATCCTAATCTCAAAATACGCCAACTCAACAA
GTACCTTCGGAGACACCTGTAGAGCACCTTTATAATCACCCAGTTACGTTGTGACG
TTTGGGAGCACACAAAGTGTTCCTCCGGTAAACGGGAGTTGCATAATCTCATAGTC
ATAGGAACATATATAAGTCATGAAGAAAGCAATAGCAACATACTAAACGATCAAGTG
CTAAGCTAACGGAATGGGTCAAGTCAATCACATCATTCTCCTAATGATGTGATCCC
GTTAATCAAATGACAACTCATGTCTATGGCTAGGAAATTCAACCATCTTTGATCAAC
AAGCTAGTCAAGTAGAGGCATACTAGTGACACTATGTTTGTCTATGTATTCACACAT
GTATCAAGTTTCCGGTTAATACAATTCTAGCATGAATAATAAACATTTATCATGGAAT
AAGGAAATAAATAATAACTTTATTATTGCCTCTAGGGCATATTTCCTTCACTCGGCG
TTATAACTTTGCGCGAGGCACTTGACGGTATCCATGGGTCCAGCCAAATATTGATA
TTAGAACCTGTCCCAACTCTCCAAATAAACTCCCTCTTAAATGCTTGAATTCCTGAT
ACTATACTCTACCAAGTATAAGAAGAGCCTTTCTTAGGGCCAGCATTAAGAATGTTA
CCATGGGGGTAATACTTTGCACTGAGAACACGCGCACACAAAGAGTCCGGATCTT
GGATCAGTCGCCAACACTGCTTTGCTAACATAGCGAGATTAAAGTTGTGTAAGTCT
CTGAATCCCATTCCACCTTCCTTATTCGGGATGGACATTTTCCACCATGCATACCAA
TGCATTTTTTTCCTATCTTCATCATCACCCCACCAAAAGCTTGACATTTCATCTGTTA

TTGACTTGCATATACTCTTTGGTAGCTTGAACACAGACATGGCATAGGATGGTATTG
CTTGAGCTACTGATTTTAAGAGAATCTCTTTGCCTTGCATAGACAAAACATTTTCTTT
CCACCCTTTCAGTCTCTGACACACTCGATCATTGAGGTGTTGGAAGCAATCGCTGC
GATCCACCCCAAGCATAGTTGGGAGACCAAGATATGTGTCTGAAAGGGCCTCCGT
TAATATATCAAGTTCTCCACAAATACCTTCTCTGACAAGGACACTAGTATTAGGGCT
GAAGAAAATACTCGACTTGGGAGTACTTACCAATTGTCCAGAGCTACTACAATAAG
TATCAAGCACCCTCTTCAGGGTCACCGCATTCTGAAGATCAGCTCTCATAAGGATG
AGAGAATCATCTGCAAAGAGTAAATGGGATATTGATGGGGCATTCCTACACACTTT
CACTCCCTCTATACCTCCTGTTTCCTCTTCATGGGCTAGCATAATTGACAATCCCTC
CGAGCAAAGTAAGAACAGGTAGGGGGACAAGGGGTCTCCCTGTCTTAGTCCACGG
GAAGGGAGGAACTCATCAGTTTTAGTATTATTAAATCTGACTCTGTAGCTTACCGAA
GAAACACATTCCATGATGAGCTCAATCCACTGCGCGTGGAAACCCATCTTTTTCAT
AATCTCTCTCAGAAATTCCCATTCAACTCTGTCGTAAGCTTTCACCATGTCCAGCTT
TACTGCGCAATAACCATTAGTGCCATGAGTTTTCTTCTTGATGGCGTGAAAGCACT
CATAGGCAACCAACACATTGTCAGTTATTAGACGCCCAGGCACAAACGCACTCTAT
GTTGGGGCTATAATGTCAGGAAGAATCTTTTTCAGCCTATTAGCAATCATCATGGA
GATGATTTTATACACCACATTGCACAGGCTGATTGGTCTATATTGTGTAATTACCTC
AGGATTTTGTACTTTGGAATCAACACGATGTTTGTTGCATTCTACCCCTCTGGGATT
TTCTTGCTGTCTATTGCTTCCAGTACTTGCTTGGTTAGTTCATCACCCAGGATGTGC
CAGAATCTCTTAAAGAAAATTGCATGTAGACCATCTGGCCCTGGGGCCTTCATATC
ACCGATCTGAAATAGAGCTTTGCGCACATCTTCCTTGGTATAGGGAGCAACTAGTA
AACCATTCATATCATTAGTTACTACAGGCTTTACCGATGCAAGAAGATTAACATCTA
CCTGTTCTGCACCAGACTGGAAAATATTTTTGAAGTAATCCGAGATAAGAGGCCTG
AGATTATCATTCCCCTCAACCCATCCATTCTCATTCACACGCAGTTTTTTGATTAGAT
TTCTCCTTCTTCTAGCCGAGGCAAAATGACTAAAGTATTTGGTGTTTCGCTCACCAT
GGCGAAGCCAGTTAACTCTTCCACGTTGTGACCAATAGATTTCATCTTGCTCAAGC
AGATTTTCAATCAACACCGACATTTCTTTCTGCTTAGCACCCACATCCGGGGTAAA
GGGAATATTCATCAATCGTTCCAACTCTCTTTGGGCCTTTTTTAGTCTCGCGCGAG
GCGCCTTGAGAATCTTCCTGTCCCAATCGTGTAGATCGTTATGGACATCAGCCAAC
TTCTCTGCAGCCGTAGGACAGAGCCCTTGGTTCACTGCCTTCAGCCAGGCAGTTTT
AACTATTTCTTCCACAGTTTCCTCCGTGAGCCACCTCGCCTCGAACTTACGTGGGG
CAATTACAGTAGGTTCTTCCACCCCTTCTAGATAATCTGTATCAAGGCAAATCGGC
GTATGGTCCGACTTACCCATCTCCAAATTACTGAGTCCAGCTAACGGATGCATTAA
CAGCCATGTCTCATTAGAGACAGCTCTGTCCAGTCTCTCTCGCAGCCCTCCACGGA
ACCATGTAAACTTGTCTCCGTTGAAGCCTAGATCCTCAAGTGAACAATCTGTTAGG
GCATCCTGGAAGGCCTGGAGGCAAGTCTGGCTGCTGGGAGGCCCACCCTCTTTTT
CATCCGAGTATAGAATCTCGTTGAAGTCCCCTATTACAACCCACGGCAATCTCGAC
TGGGCATGCAAATCCCTCAATAGCTGAAAGGTACGGTGTTTACAACTCCAGCTAGG
CTCGCCGTAGATGCCAGTCAGTCTCCACATATCACCATTAGCATTCTCCACCGTCA
CATCTATGAAATCTAGTGTGCTAGCCCGGAATTAGATCCTAACCTCCTTCTTCCAG
GCCAACAGCAAGCCTCCTTTTCTTCCATCTGAACTTGGTGCAACCAACATCTCGTC
CATCTGCAACTTGATCTTAAGAACTTCAGCCTTGTCATTATCCAAATGTGTTTCCGA
CAGAAAAAAACACATCCGGACTGTGCCGCCTTTGGATCTCCAGAAGCGACCGAAC

TGTCGGGGCACCGAGGATCCCCCGACAATTCCAACTTAGTATTTTCATTGCGGGCA
GCGATCCTCCTCGAAGGAGATCGCCGATGAATTGTTTGTTGTACCCGGACTGCAA
GGACTCGCCAAGGGAACAGCCAGAACTGCAGTAACTAGAACCGGTCGTTGCAAAC
GAACGCCGGGACAGGAAACCGATCACTGCACCAGGCAGCGTCGGGACAGAAAAG
CTTGAGCAACTCCAAGGTTGTCTTCAACTCCAATCAACAACCAAATACCCTTAAGCA
CCATCAGGAAAACTGTACGCAAACGAAGCAGTGGAACCTCATGGCGTACAAAGCC
AAATACACACCAGGCGATCAACTCATGAAGCCTGCACCGGCACGGGACTAGCCGA
AACCTAGCAAGGCCGAAACCCTAGCCAAGAACGAGAGACCAGCCACGGCGCCTC
CGGCGCCGTCCTGGGAAAGAGACGCCAGCACACGCGCCGCCGGCAAAACCCTAA
CGGGTCAACGGGTTTCTGTAGTCTCGCCTCGTACTTGTCTTGGACCTGGGCTTCGT
TGTGATTAGCACAGTTTTCTGTCCTAAATGTATTTCCATTTGGTTATTATATACTGAC
AAGTCAAAGCTGTATTCCCTGAATCCGTGGGCGCTTGGCAGCGGACGATCTGGAC
CGTCCACTGAGTTCAGGTGGACCAGGTCCGACTAACTTTTTTTTTTGAGAACTATA
GCTCCGACTAACTAAGCAGCAAATCCTAGACGATGCTCACCGCGTCAAACGACCA
AACATGCAGGCCGACAGCCCGTGTTTTTTGTCTCTTCTGTATTTTTTCTGGCCTTTG
TGACGTTTTATAATTTTTCTTTTGCTTTTCACTTTCTACAGGTCTTCTATCTTTTTTCT
CTTTTTGTTTCTTTTGTTATTTCTCTTCCATTATTCTTATTCTTATTTGATTTTAATCCT
TTTCTTGTTACTCTCTTTAACTCTTTTTACTTTGATTGATACAAAGTTGGGTAAACTTT
TTTGAGCATATGAATATAACTTTTGCATGGATGAAATATTTCCAAATTTGCGATTAAA
CATTTCATTTCACATACATGATCAGTTTTAAAAATTATTAATGTTTCTAAAAGTACATA
AAAATTATACATGATATAGAGGTTTTTTAATTATGTGATATTTTTTCATATGCATGACA
TATTAACAAAACATGATTTTTTTAAAATTTATATATATATATATATATATGTAATTTCTC
TCTACTGAAACTAATTTTATTTATACATGAATAATTTGGTGCTATGTAATAATATTTTC
AAATAAATGAACATTTTTTAGCCGATGATTTTTCTTGGGAATTTTCTAACTAATTTAAT
ACAAGAATAAAATTGTTTGTATACTTTTTAAAATATGAAATGAATATTTTAGTTCATAC
TCCCTCCGTAAAGAAATATAAGAGCGTTTAGATCACTAAAGTAGAGATCTAATAGCT
CTTATATTTCTTTACGGAGGGAGTACATATTAACATTTTTATCTGAATATGTGAACAT
TTTGTTATTTTACGCAATATAATTTTTCCCCGTTTTTGAAACTTGTTTTTTGATAACAT
TATTTAAATGATTATTTTTCCTTTTTTGCAAATATATAGTACTACCATTATTTAATCTTT
TATTGATATTTTTCATAAATCTGTTAACTATAAAAAATTAAACCTAAGTGGGTTGGTC
TTGTGATGCGTGGAGTGGGTTTGACCACCTATCTGATGCATCTCTCCGCCAACCAA
CCTGAATTTTCATGTATCAAATTTGAAGTGAGTCTATAAAAAAAATTGAAGTGAGTC
GATTAGTACCCATGTTTGTTATGCTACTTGCCATGGATCGATTGCCAAAAAAAATCT
ATCGAAACAATTTGTGTACATTTTCCATGGACTAGTCACAGCTAAAAAAACGCCATA
GCTAAAAAAAAAGAGAATTGTACACTTTACTTGCTCACAGTTCTTCTTTGCTTATGTA
CGCTCGCACGCAGAGGAGGTACGGTTGCCGCGCGATGATGCTGGAGACGGTGGC
CGCAGTCCCCGGCATGGTGGCTCGCGCGGTGCTCCACCTCCGGTCGCTCCGGCG
CTTCGAGCAGAGCGGCGAGTGGATCCGTGCACTGCTGGAGGAGGCGCAGAACGA
GCGCATGCACCTCATGACCTTCATGGAGGTGTCCCAGCCGCGGTGGTACGAGCG
CGCGCTCGTCGTCGTCGTCCAGGGCGTCTTCTTCCACGCCTACCTCGCCACCTAC
CTCGCCTCCCCAAAGGTCGCGCACCGCATGGTGGGGTACCTGGAGGAGGAGGCC
GTGCACTCCTACACCGAGTTCCTCCGTGACCTCGAGGCCGGCAAGATCGACGACG
TGCCCGCGCCGGCCATCGCCATCGACTACTGGCGCCTCCCGGCCGGCGCGACCC

TGAAAGATGTCGTCAGGGTTGTCCGCGCCGACGAGGCGCACCACCGGGACGTCA
ACCACTACGCCTCTGTAAGCGTCGCCTCCGTCAAATTTGCCAAGCTGACATCAATT
GCAGTCTTCTTTGTACTGAAATGAGTGCATGTGTGCAGGACATACATTGCCAGGGG
CATGCACTGCGAGAGGTAGCTGCGCCGATCGGCTACCACTGA
>*put.TaAOX1d-like-4AS* gene
ATGCCCACCACCGCGAGGATCTTCCCCGCGCGGATGGCCAGCACTGCCGCAGGC
CCGCATGCCAAACAAGAAGAAGCCACTGGAAAGCCCCAGGGCGCAACAACGCCG
GAGCAGAACAAGAAGGCCGTGCCGAGCTACTGGGGCATCAAGCCGCGGAAGCTC
GTCGAGGACGACGGCACGGAGTGGTCGTGGTTCTCCTTCAGGCCGTGGGACACG
TACCGGCCGGACACGTCCATGGACGTGGCCAAGCACCACGAGCCGAGGGCGATG
GCGGACAAGGTGGCGTAACTCATCGTGCGGACGCTGTGCGCGGGTAGCGACCTC
TTCTTCCAGCGCCGGCACGCCAGCCACATGCTGCTGCTGGAGACGGTCGCGGCG
GTGCCACCCATGGTGGGCGGCGTGCTGCTGCACCTGCGCTCGCTCCGCCGCTTC
GAGCACAACGGCGGCTGGATCCGCGCGCTCATGGAGGAGGCCCAGAACGAGCG
CATGCATCTCATGACCTTCATGGAGGTGACGCAGCCCTTGTGGTGCGAGCGCGCG
CTCGTGCTCCCCACCCAGGGTGTCTTTTTCAACGCCTACTTCATCGGGTACCTCGT
CTCCCCCAAGTTCGCGCACCGCTTCGTCGGCTACCTCGAGGAGGAGGCCGTACA
CTGA
>*ne.TaAOX1d-2BL.1* gene
ATGAGCTCTCGGATGGCCGGAGCCACGCTGCTGCGCCACCTGGGCCCCCGCCTC
TTTGCCGCCGCCGAGCCGGCCTCCGGGCTCGCCGCCAGCGCGAGGGGCATCAT
GCCCGCCGCCGCGAGGATCTTCCCCGCGCGGATGGCCAGCACCGAGGCCGCCG
CCCCGCATGCCAAACAAGAAGATGATGCCGGAACACCCCAGGCGGCCGCGACTC
CAGAGCAGCAGAGCAAGAAGGCCGTGGTGAGCTACTGGGGCATCGAGCCGCGGA
AGCTCGTCAAGGAGGACGGCACGGAGTGGCCGTGGTTCTGCTTCAGGCCGTGGG
ACACGTACCGGCCGGACACGTCCATCGACGTCACCAAGCACCACGAGCCCAAGG
CCCTGGCGGACAAGGTGGCCTACTTCGTGGTTCGGTCGCTGCGTGTGCCGCGGG
ACCTCTTCTTCCAGCGCCGGCACGCCAGCCACGCGCTGCTGCTGGAGACTGTGG
CGGCGGTGCCGCCCATGGTGGGCGGCGTGCTGCTCCACCTGCGCTCGCTCCGCC
GATTCGAGCACAGCGGCGGCTGGATCCGGGCGCTCATGGAGGAGGCCGAGAAC
GAGCGCATGCACCTCATGACCTTCATGGAGGTGACGCAGCCGCGCTGGTGGGAG
CGCGCGCTCGTGCTCGCCGCGCAGGGCGTCTTCTTCAACGCCTACTTCGTCGGCT
ACCTCATCTCCCCCAAGTTCGCGCACCGCTTCGTCGGCTACCTCGAGGAGGAGGC
CGTGGAGTCTTACACTGAGTACCTCAAGGACCTCGAAGCCGGCTTGATCGAGAAC
ACGCCCGCGCCGGCCATCGCCATCGACTACTGGCGCCTCCCCGCCGACGCCAGG
CTCAAAGACGTCGTCACCGCCGTGCGCGCCGACGAGGCGCATCACCGCGACGCC
AACCACTACGCATCGGACATCCATTACCAGGGAATGACGCTGAATCAGACGCCTG
CGCCGCTCGGGTACCACTGA
>*ne.TaAOX1d-2BL.2* gene
ATGAGCTCTCGGATGGCCGGAGCCACGCTGCTGCGCCACCTGGGCCCCCGCCTC
TTCGCCGCCGCCGAGCCGGCGTCCGGGCTCGCCGCGAGCGCGAGGGGCATCAT
GCCCGCCGCCGCGAGGATCTTCCCCGCGCGGATGGCCAGCACAGAGGCTGCCG
GCCCGCGTGCCAAACAAGAAGAAGCCACTGAAAAGCCCCAGGGCGCAACAACGC

CGGAGCAGAACAAGAAGGCCGTGGTGAGCTACTGGGGCATCGAGCCGCGGAAGC
TCGTCAAGGACGACGGCACGGAGTGGCCGTGGTTCTCCTTCAGGCCGTGGGACA
CGTACCGGCCGGACACGTCCATCGACGTGGCCAAGCACCACGAGCCCAGGGCGG
TGGCGGACAAGGTGGCGTACCTCATCGTGCGGACGCTGCGCAAGGGAAGCGACC
TCTTCTTCCAGCGCCGGCATGCGAGCCACGCCTTGCTGCTGGAGACGGTGGCCG
CGGTGCCGCCCATGGTGGGCGGCGTGCTGCTGCACCTGCGCTCGCTCCGCCGCT
TCGAGCACAGCGGCGGCTGGATCCGCGCGCTCATGGAGGAGGCCGAGAACGAG
CGCATGCACCTCATGACCTTCATGGAGGTGACGCAGCCGCTGTGGTGGGAGCGC
GCGCTCGTGCTCGCCACTCAGGGCGTCTTCTTCAACGCCTACTTCGTCGGCTACC
TCATCTCCCCAAAGTTCGCGCACCGCTTCGTCGGCTACCTCGAGGAGGAGGCCGT
CCACTCCTACACCGAGTACCTCAAGGACCTCGAGGCCGGCTTGATCGAGAACACG
CCCGCGCCGGCCATCGCCATCGACTACTGGCGCCTCCCCGCCGACGCCAGGCTC
AAAGACGTCGTCATCGCCGTGCGCGCCGACGAGGCGCATCACCGCGACGCCAAC
CACTACGCATCGGACATCCATTACCAGGGAATGACGCTGAATCAGACGCCTGCGC
CGCTCGGGTACCACTGA
>*ne.TaAOX1d-2DL* gene
ATGAGCTCTCGGATGGCCGGAGCCACGTTGCTGCGCCACCTGGGCCCCCACCTC
TTCGCCGCCGCCGAGCCGGCGTCCGGGCTCGCCGCGAGCGCGAGGGGTATCCT
GCCCGCCGCCGCGAGGATCTTCCCCGCGCGGATGGCCAGCACCGCCGCCGGCG
CGCATGCCAAACAAGAAGGTGACGCTGAAAAGCCCGAGAGCGCCACAGCGCCGG
AGCAGAACAAGAAGCCCGTGGCGAGCTACTGGGGCATCGAGCCGCGGAAGCTCG
TCAAGGACGACGGCACGGAGTGGCCGTGGTTCTCCTTCAGGCCGTGGGACACGT
ACCGGCCGGACACGTCCATCGACGTGGCCAAGCACCACGAGCCCAGGGCGGTG
GCGGACAAGGTGGCGTACCTCATCGTGCGGACGCTGCGCGCGGGCAGCGACCTC
TTCTTCCAGCGCCGCCACGCTAGCCACGCGCTGCTGCTTGAGACGGTGGCGGCG
GTGCCGCCCATGGTGGGCGGCGTGCTGCTGCACCTGCGCTCGCTCCGCCGATTC
GAGCACAGCGGCGGCTGGATCCGCGCGCTCATGGAGGAGGCCGAGAACGAGCG
CATGCACCTCATGACCTTCATGGAGGTGACGCAGCCCCTGTGGTGGGAGCGCGC
GCTCGTGCTCGCCACGCAGGGCGTCTTCTTCAACGCCTACTTCGTCGGCTACCTC
ATCTCCCCCAAGTTCGCGCACCGCTTCGTCGGCTACCTCGAGGAGGAGGCCGTCC
ACTCCTACACCGAGTACCTCAAGGACCTTGAGGCCGGCTTGATCGAGAACACGCC
CGCGCCGGCCATTGCCATCGACTACTGGCGCCTCCCCGCCGACGCCAGGCTCAA
GGACGTCGTCATCGCCGTGCGCGCCGACGAGGCGCATCACCGCGACGCCAACCA
CTACGCATCGGACATCCATTACCAGGGAATGACGCTGAATCAGACGCCTGCGCCG
CTCGGCTACCACTGA

TuAOX:
>*TuAOX1a* gene
ATGCTCGACAAGATCGCCTACTACACCGTCAAGTCCCTGCGCTTCCCCACCGACA
TCTTCTTCCAGGTACGCGCGCCTCCTCCCTCCCCTCCCCTCACCGGCCGCCACAA
CCGCCGGATGGTTCGTTCCGTGTTTTTAGTCCAACCCGTTTCGCCGAAGACAGCA
CGAACGGCGGTGTCTGCGTCTTTGCGTGTCATGGCCTCGTGGGTGACGGATGTAG

CATGGTCCCTGTCACGCTTTTGATTCTGTTCCCCCCTGCTTTCCGGGGAGACGGC
GTTTGGACTCGCTATTTAGAACGCGGACCCAAGCCGTCATGCCGAGTATTTTTTAC
CTAATCGTTCGTTCGTGGGGATGGGTGTTTTATATTCTGCATCAGATTACTGTGGAT
AATCGCGTGCTGGAGAAATCCGCTGACAGTGGATGGAAGTATCGACAGATTATAG
ACTGTCAGCAAACATTAGGAATTGGAAACACAAATAACATGGCGATCCTTGACATC
GCTACTACATCGGACATTTTATTATTGATTAAAGCACCAGAGAGGATGCCATTTGTT
TTGCCATGTACTTTACTTTACTTTATTAAAGCATCTGTTTATCATGATTTTTGCACAA
GCTGTATCATCTTGATCTCCACTCTGTATTTCGAAGTTCATCATGATCTGAAGCTGA
CCCTTCAAATTGGTTGCAGAGGAGGTATGGCTGCCGCGCAATGATGCTGGAGACT
GTCGCCGCAGTGCCGGGGATGGTGGGCGGCATGCTCCTCCACCTGCGCTCCCTC
CGGCGCTTCGAGCAGAGCGGCGGGTGGATCCGCGCGCTGCTGGAGGAGGCCGA
GAACGAGCGCATGCATCTCATGACCTTCATGGAGGTGGCGCAGCCGAGGTGGTA
CGAGCGCGCCCTCGTCATCGCCGTCCAGGGCGTCTTCTTCAACGCCTACTTCTTC
GGCTACCTCATCTCGCCCAAGTTCGCGCACCGCGTCGTCGGGTACCTGGAGGAG
GAGGCCGTCCACTCCTACACCGAGTTCCTCAAGGACCTCGACGACGGCAAGATCG
ACAACGTCCCCGCCCCGGCCATCGCCATCGACTACTGGCGCCTCCCTGCCAACG
CCACCCTCAAGGACGTGGTCACCGTGGTCCGCGCCGACGAGGCTCACCACCGCG
ACGTCAACCACTTCGCATCGGTATGAATCCTTCCAAATCCCACATGATCGGCATTC
TTTCTTGACCCCATTTCCTGACCAGTCCATGTGCATCTGTTTTGTGTGGATTGGTTC
TGAATTTCTTATGATGTATTCTGATTTTGTCTGTCGATTGTGTCGTGCAGGACGTGT
ACTACCAGGGTATGCAGCTGAAGGCCACCCCGGCGCCGATCGGATACCACTGA
>*TuAOX1c* gene
ATGACCCAAAGGTATAAAATTTCGCCAACTTTTTTGTTCTTGTTATTTTTTGAATGCA
TGCATATAGATAGCTTATTTGCTTCATTGTGTATACTTTGTAGTTTCAACAATGAGGC
ATACATGCCAACTATGGGTGTTGGCTTCAACAATTCGCATTGGTCTCAAATAAATGA
CATGCATCTCGATGACCATGAGTTCGAGGTGGACGAGGATGGTGAGGGCATTGTC
GATGCACCGAAAGGAAGAGGAGGCAACTACACCAATGAAGAAGACGTCTTGCTAT
GCAATACTTGGTTGCAAGTGTCGAGGGATCCATCCGTTGGAGGTGATCAAAGTAG
AGATGCTTATTAGAACCGGATGAAAGAGCATTTTGATCTACACAACAAGAGTGGAA
GTGATCACTCCGACCGATTTCTTCGCTCCCGGTGGTCGACAATCAACAAAGATTGT
CAAAAGTGGGTGGCCGCACAAATGGCGGTTGACAAGTTGAATTCAAGTGGCATTA
ATGATGAAGATAGGGTAAGTGCCATTTCGTCCATGTTCATCATGCTTGTTGTTGGT
GTTTGTAGTGCTAAATTGTTTTGTTTTTATGTAGTTCAATATTGCACAAAACTAGTTC
AAAGGAGAGGAGAAGAAGAGCAAGAAAGGAAGACCATTTACCTTGCCTCATTGCT
ATGAAGCATTGAAGGATGATGAGAAATGAAAAAAGCGTGATGGTATGGATGATTTG
GATATGAGCAACAAACACATGCAAACAATTGATTTGGATGAGGAGGAGGAGGAGG
CATCAAGTGATGACGGCAAGAGAAGCCCCACACCCAACTCGGTTTCATACTCGAA
GCCAAAACGACTGGATGTGTGCAAGAAAGACGCAAAAGAAAAGAAGAAGAGGAAA
AGAGATGATGAGCTAAAAAATGCTATGAAAACTATTGTGAAGGGAAGAAAAGAAGC
GAACGAGGTGAGGAAGATGGCAAGGAACCAAGATGCCGCGGCCGAGGAGAGGAA
GGTGACATTGGAGGAGAGGAAGGTGGGCATGGAGGAGCGATCTAGGTTGTTGTG
TTGGATTGGGAGAAGTACTTGTTCTTCATGGACACATCTATCCTCAATGAGGCGTA
AAAGGAGCATGTCAATCTTGCCCGTGAAGAAGTCCTGATCGAAAAAAAAGCCATGA

TTCGCGGCATGGGTGGTGGTGGCCTTGGCGGCATGGGTGGCCTTGGCGGCATGT
GAGGCATCGGTGGCTTCGGAGCTATCATGGATGGCTTTGGAGCTATCATGGAGAC
CATGGGAGGCATGGGTGGCTTCGGAGCACCTCCGGGCGGCACGGACTGTATGGG
AGGCATGAGTTTTGCGTCTCTCATGGGAGGCATGGAAGCACCTCGGGCGACATGG
CGGAATGTCTTCCGGGGTGCCTCACACACCTTCGCATGAAGATGCCGTTGAAAAT
CTAGCCAACACCTTCCGAGCTTCATATGATGATGCGGCGTGTGACAATGAAGAGG
AGGAGGAACAATCGTCTTCGGAGGAGGAGGATGAGTCGGAGGAAGATGAGGATG
AAGACGAGGACGAGGCTTAATTATTGATGTGCCTTTCGTTGATGTGAACTTTTGTGT
CATGAATTTGTTTTGGATTTTAAACTTGGTCAGATGATGTTATGGGCATGAATTTGA
ATTTGTGGGCATGAACTTTTATGTCAGCATGAACTTGTTTGTGTGATTTGAATTATG
CCATGTCATTGTTTTGATGTTTGAAATTCATTTTGTGTCCAAAATGCAACATATGGCA
AGCGCGGCTGCTCCCCCGCGCATGCGGGAGCTGCGCGCGCTAAATTTTATCACG
GCTGCTGGAGNNNNNNNNNNNNNNNNNNNNNNNNNNNNNNNNNNNNNNNNNNNNNNNNNN
NNNNNNNNNNNNNNNNNNNNNNNNNNNNNNNNNNNNNNNNNNNNNNNNNNNNNNNNNNNN
NNNNNNNNNNNNNNNNNNNNNNNNNNNNNNNNNNNNNNNNNNNNNNNNNNNNNNNNNNNN
NNNNNNNNNNNNNNNNNNNNNNNNNNNNNNNNNNNNNNNNNNNNNNNNNNNNNNNNNNNN
NNNNNNNNNNNNNNNNNNNNNNNNNNNNNNNNNNNNNNNNNNNNNNNNNNNNNNNNNNNN
NNNNNNNNNNNNNNNNNNNNNNNNNNNNNNNNNNNNNNNNNNNNNNNNNNNNNNNNNNNN
NNNNNNNNNNNNNNNNNNNNNNNNNNNNNNNNNNNNNNNNNNNNNNNNNNNNNNNNNNNN
NNNNNNNNNNNNNNNNNNNNNNNNNNNNNNNNNNNNNNNNNNNNNNNNNNNNNNNNNNNN
NNNNNNNNNNNNNNNNNNNNNNNNNNNNNNNNNNNNNNNNNNNNNNNNNNNNNNNNNNNN
NNNNNNNNNNNNNNNNNNNNNNNNNNNNNNNNNNNNNNNNNNNNNNNNNNNNNNNNNNNN
NNNNNNNNNNNNNNNNNNNNNNNNNNNNNNNNNNNNNNNNNNNNNNNNNNNNNNNNNNNN
NNNNNNNNNNNNNNNNNNNNNNNNNNNNNNNNNNNNNNNNNNNNNNNNNNNNNNNNNNNN
NNNNNNNNNNNNNNNNNNNNNNNNNNNNNNNNNNNNNNNNNNNNNNNNNNNNNNNNNNNN
NNNNNNNNNNNGCGCGTTAAATTTTATCACGGCTGCTGGAGCAAGCGCTCCCC
GCCGTGCCAAAGCAGACGGTCGACACACTGCAAACTGATTTTTACCGTGTCACGG
TATTGGGCGGCTGTTGGAAATGCTCTAAGAACGAGGGGAAAAACAGTAGGCCACT
CTAATTTTCGTGTCAAAACAAGTCCACTAAAACTAACAATCTTTCCTCATCCGTTGA
AATGGGAACACAAAGATTCTAGGCACGCCGACGCACGGCCAAGTTCAGAGTCCAC
CGGAAGTCCTAGCAGCCACCCCACCACATAGCAGCCGCAACGATTTTGACACCCA
TTCCTGCCTCCTGAAAAGCTTTTTTTTTTTTTNNNNNNNNNNNNNNNNNNNNNNNN
NNNNNNNNNNNNNNNNNNNNNNNNNNNNNNNNNNNNNNNNNNNNNNNNNNNNNNNNNNNN
NNNNNNNNNNNNNNNNNNNNNNNNNNNNNNNNNNNNNNNNNNNNNNNNNNNNNNNNNNNN
NNNNNNNNNNNNNNNNNNNNNNNNNNNNNNNNNNNNNNNNNNNNNNNNNNNNNNNNNNNN
NNNNNNNNNNNNNNNNNNNNNNNNNNNNNNNNNNNNNNNNNNNNNNNNNNNNNNNNNNNN
NNNNNNNNNNNNNNNNNNNNNNNNNNNNNNNNNNNNNNNNNNNNNNNNNNNNNNNNNNNN
NNNNNNNNNNNNNNNNNNNNNNNNNNNNNNNNNNNNNNNNNNNNNNNNNNNNNNNNNNNN
NNNNNNNNNNNNNNNNNNNNNNNNNNNNNNNNNNNNNNNNNNNNNNNNNNNNNNNNNNNN
NNNNNNNNNNNNNNNNNNNNNNNNNNNNNNNNNNNNNNNNNNNNNNNNNNNNNNNNNNNN
NNNNNNNNNNNNNNNNNNNNNNNNNNNNNNNNNNNNNNNNNNNNNNNNNNNNNNNNNNNN
NNNNNNNNNNNNNNNNNNNNNNNNNNNNNCTGGGGCATCGTGCCTGCCAAGCTG
GTGAACAAGGACGGCGCCGAGTGGAAGTGGTCTTGCTTCAGGGTAATGAAATCGT

CTGCATATTCTCCTTGTCGTTGACAGTGCACGTAGCCGTGGTGACCGTGTGATTTC
GCAGCCGTGGGAGGCGTACACGTCGGACACGACCATCGATCTCTCCAAGCACCA
CAAGCCCAAGGTGCTGCTCGACAAGATCGCCTACTGGACCGTCAAGTCGCTGCGC
GTGCCCACCGACATCTTCTTCCAGGTACGGCACGGGGGTCAGATATATGTGTTCG
TCCGACACTGACATGACACGGCGGGTCGACTTGGGCCTACGCTATGCAGCGGAG
GTACGGGTGCCGGGCGATGATGCTGGAGACGGTGGCGGCGGTGCCGGGGATGG
TGGGCGGGATGCTGCTGCACCTGCGGTCGCTGCGGCGGTTCGAGCAGAGCGGC
GGGTGGATCCGGGCGCTGCTGGAGGAGGCGGAGAACGAGCGGATGCACCTGAT
GACCTTCATGGAGGTGGCCAACCCCAAGTGGTACGAGCGCGCGCTGGTGCTGGC
GGTGCAGGGCGTCTTCTTCAACGCCTACTTCCTGGGGTACATCGTGTCCCCCAAG
TTCGCGCACCGCGTCGTGGGCTACCTGGAGGAGGAGGCCATCCACTCCTACACC
GAGTTCCTCCGCGACCTGGAGGCCGGCAGGATCGAGAACGTCCCCGCCCCGCGC
ATCGCCATCGACTACTGGCGCCTCCCCGCCGACGCCAGGCTCAAGGACGTCGTC
ACCGTCGTGCGCGCCGACGAGGCGCACCACCGCGACGTCAACCACTTCGCCGCG
GTACGCTCATCTCCCATGCATTAGTCCCCGATGTCTCGACGTCTGAACTCTGCTCT
GCTAGCTTCTCCTGATGTGATGTCTGGACTCTGAACTGTTTTTTTGATTCTGGTGAA
TGGTGATGGGCATGCAGGACATCCATTTCCAGGGGCTGGAGCTCAACAAGACGCC
TGCCCCGCTAGGATATCACTGA
>*TuAOX1d.1* gene
ATGCCCGCCGCCGCGAGGATCTTCCCCGCGCGGATGGCCAGCACCGAGGCCGC
CGCCCCGCATGCCAAACAAGAAGATGATGCCGCGAGCCCCCAGGCGGCCGCGAC
TCCAGAGCAGCAGAACAAGAAGCCCGTGGTGAGCTACTGGGGCATCGAGCCTCG
GAAGCTCGTCAAGGATGACGGCACGGAGTGGCCATGGTTCTGCTTCAGGCCGTG
GGACACGTACCGGCCGGACACGTCCATCGACGTGGCCAAGCACCACGAGCCCAA
GGCCCTGGCGGACAAGGTGGCCTACTTCGTGGTTCGGTCGCTGCGCGTGCCCCG
GGACCTCTTCTTCCAGCGCCGGCACGCCAGCCATGCTCTGCTACTGGAAACGGTG
GCGGCGGTGCCTCCCATGGTGGGCGGCGTGCTGCTGCACCTGCGCTCGCTCCGC
CGCTTCGAGCACAGCGGCGGCTGGATCCGGGCGCTCATGGAGGAGGCCGAGAA
CGAGCGCATGCACCTCATGACCTTCATGGAGGTGACGCAGCCGCGGTGGTGGGA
GCGCGCGCTCGTGCTCGCCGCGCAGGGCGTCTTCTTCAACGCCTACTTCGTCGG
GTACCTCATTTCCCCCAAGTTCGCGCACCGCTTCGTCGGGTACCTCGAGGAGGAG
GCCGTGGAGTCTTATACTGAGTATCTCAAGGACCTTGAGGCCGGATTGATCGAGA
ACACGCCCGCGCCGGCCATCGCCATCGACTACTGGCGCCTCCCCGCCGACGCCA
GGCTCAAGGACGTCGTCACCGCCGTGCGCGCCGACGAGGCGCATCACCGCGAC
GCCAACCACTACGCATCGGACGTCCATTACCAGGGAATGACGCTGAATCAATCGC
CTGCGCCGCTCGGGTACCACTGA
>*TuAOX1d.2* gene
ATGGCCAGGAAACCGTAACCATCTATTGATCAACGAGCTAGTCAACTAGAGGCTTA
CTAGGGACATGGTGTTGTCTATGTATCCACACATGTATCTGAGTTTCCTATCAATAC
AATTCTAGCATGGATAATAAACGATTATCATGAACAAGGAAATATAATAATAATAAAT
TTATTATTGCCTCTAGGGCATATTTCCAACATAGTTAACTTTTCGGCCTCGCTAAAT
ATGAACATGCCAATTTTTTTGCATTAGACCAATTATTGCCCCTATAGAGTATGCTTC
AGGTGAGCAAGCAAATGGCGAGCTCGCCCATAGCTGGGGTAGTAGGATGTACCTT

GGAACAAACCTTCGTCGGGATGGTCTACGTTTAATGTGGACGACTGACACGTCGC
TGGCCAAAGCACTGGATGAGTTGGCATGGTGCGGAACAACACACGAGCCATCATT
TTCTCAGCGTGTCATTTCCCTCGCTCATGTGGATTGCCAATGTAGGCGGAAATTGC
AGCGTTTCTCGAGGGTACGTCCTTGGAGTTGTACCGGACGAACATATCGTTGATCA
CTGAACTAGATTGTGGAAGGAGCGGATGCTCTGTCAAATAGGAGTATCAGTAGATC
TTTGTTGGTTGGCATGGTGGAGGAGACGAAGAGACTCCTGCATGGGATCCAACGG
CATTGCTTTCTCTACGCCCGAAGATCGGTGAAATGGTGTTGTACACTGCATGGCTC
AGATAGGACGTTGCTCCCAATGCTCGGTAGTCTGGTTAGGTGCTAGGCCAGATGA
TTTATGTAAAATCTATGAAAATGAGTCTAACATTATTCCTTAATTAATACAATCCTGT
TTTACCGTGCAATTTTTTTTGCCCCCACCTTGTTTCTTAGGACATGTACAATGATCG
ATAAGATTGTCTTATCTTAAGCCTTACACACAATCTGGAGAACCCAGTAAAAATATG
ATGTACAATGATTATTTTATAACCTTATCTCTAATAATTAAGACATCCCTAAAAATATA
ACAAGACATTGTTGGTAAGCGATCACCTCTTCATTAAAGGAAGGCAAGTCTTTTCTT
CTCCTTTCTTTTTTCTCCACCTCAGTAGATATTCTAGTACATGACACTCGTAAGATA
GCATTATTGCATATGCCTTAAAAAACATATGCTCTTAAAAATGCAAAAGATAGACAA
AGTGATCGCCAGAGGTCAAGAAGATCTAACCCATCTTTGCTCGCCATGTGGCGCG
AGAGGCACAGTTGATATCCGGGGGCGTTTTTTTCTTGGTAAAGACGAAAATTCGTT
GACTAAGCTTGACAGGCCTGCACATCGGCCCAAACAGCCTTCGATCGTCCCAGTG
GCACACCACCGAAGCCCAAGAAAAGTATGGTCGCTACGCCCAGCTCCAACCATCG
TTCACCCCCAATTCCACCTTCACCTCTCTCCACCGCCGGCGTCCGGCGACCGCCG
CTTGCCGGCCTACCGGCGATGGACCGCGCATCCACTTGATGGATCCGTCGGCGG
CAGGGGCCGCGGCACCACCGGGNNNNNNNNNNNNNNNNNNNNNNNNNNNNNNNNNNNNNN
NNNNNNNNNNNNNNNNNNNNNNNNNNNNNNNNNNNNNNNNNNNNNNNNNNNNNNNNNNNN
NNNNNNNNNNNNNNNNNNNNNNNNNNNNNNNNNNNNNNNNNNNNNNNNNNNNNNNNNNNN
NNNNNNNNNNNNNNNNNNNNNNNNNNNNNNNNNNNNNNNNNNNNNNNNNNNNNNNNNNCC
TCAGCGTGGGCGGCGCGGCAGCGGCGGCGCCTGCGCCCCCACGGGCCGCCCC
GGGCAGAGGGGCGGACCCAGCGGAGCGGCGCAGGCGGTGGTGCGGGATCACA
GTCCGCGGGGCCCTCGTGATGCTCTTCCCCATCGCCGTGTCATTCCTCTTCTCCTT
TATCTTCGGCATCGCTGGCCTCCTCCTCGGCGGGCTCTCCTCCAACGCGTCCGTC
TCCATGCCCTCCACCTGCCGCATCCTCTCTACCGGTCGGCCACTGTACCTCCCCTT
CCATCTCTTCCCTCCCGATTCACTCCCATGCTGTTCCCAGAGCTTAGGAATCTAAC
CGGGTGCTGAAGTTAGCATGCAAATTAGCTGCTTTGGTGTTTAGCGTGGAGAGCTT
ACTGCAAATTTTGTGATGTATATTCCATTTATGCACTTTACCATCTGCTCCACCAAGT
CTAAGAGCTGCTTACGTATTCCCCACTTGATGTGTGCTGGATGCTGATTACACTGT
GGCTCAGCTAGTGAGCTTAGGATGTGATTAGTTTTCCCTTTTGGTTTGGACCGAGC
TGCGTTGATCTGTAGCCTGATGCGGTTGTTGGCGCATTGTAGGCCTGGACATTCG
GTCATCTAAGGTTTGCGAGCTTGGACTGTTGAACTATAAAGCTAAGCATGTATTTTA
CCCCTCGAGTAATCGAAGATTCCGCTGCCATGATGACTATTACTGGGCTTCGGTTT
TTGAGGTGAGAATTTATATTGTGATTCTACTTTGGTGTTTGTTTCTCTAGTGGAGCA
GCTAAAAATGTTGTTCAAGCTGCTGGTTGGTTGTTTCACCTGCTTTATCTTCTTGTA
CCAGCACAGCGACATTTTATACATCATGTATTAACTACATTTTAGCTATTCTCACATT
CAGCTCCTCTAGATTTGGGCTGTCTAATGTTTTTAACCGATAATGATATATACAGAC
CTTTGATTCTTTCTATGTGCTCTCAACTCCCAAATAAGGCAATAAATGATGAACAAT

TCTAGCTGTAGTTTCTAGTTTGTTGATAAAGGACCTGTAAAAAAAAGGACACGACAT
ATTTACATTTCGAACATCAGCTTGCGGTCATGCACGTCTTTCAGTTTACTATTAGTA
TTCACGAGGATTACTGTATGTGTATTTAATTAATGGGTGGGGATATAGGTATCCAGA
ATTTAGGAGGGGAAAGGCCATGTGTGTGCAAAACAAAATTAAATAAGAAGATCATG
ATACTGAAACGCACAGCCTCTCATTTCTATTCCAAGTTAGTTTCACTTACTGAAGTA
GCTTAACACCTTCCAAATTTATCATATCAAATAAAGAAGGTCATGAACATGTTCAATT
CTCACATACCTCATAAGAAAATGACTTGTCAGCACTCAGCAGCAGACCATCGTTTT
AGGTAGCTAATAAAGAAAGAAAACAGCAGGTGTTAACTCAACAAATCAAAGGATCA
CCTGATAGGACAGACAAGTACTTGTATTGACTGCAAAATACTGCTCAAAGCTAACT
CAACATAGCAACAAATGTGGGTATCATAGGCTTACGTTAGTGCCTAAGAGACTCAG
GAGGCTGATACTCTTTGTTGTAAGCCAGAACATCACAAAATGTACTTCTCAGCACG
GTAGAAAAATTTACGGGCAGAACCTACCATAGGTAACAGAAGCAAATGAGCAAACA
CCTAGTTACTCAGATGAGCAATCAGCACCTTATTGAGCAAGCTGGGGTCCTTGAAG
AGAAGACGGGATTACAGAAGAGCCTTACGAGTGCATTCATCTGATCTTGTGGATCT
GCGATGGCATCACCATGTGTGCCGCAGCCTACGCCGGTCTCACCTTCGCTTCCAT
GTGCACGACGGTTGCGAACTCAACAGTACTGCCACCTCTCCTATCGCTCCCGTGC
GGGACCCTTGTAGAGGGCATGCTGTCGGTGCCTGTAACGCTGATGGCTGGGACG
GCGTGCCGTGCTAGGACAGCGTCTCCGTTGGCGGGTTCGCCGTTGCGTTCTCTGA
TTGGGGCGTTGGGGCGGCTCCTCCCACACCGGTGACCCGAACCACGACATCAGG
NNNNNNNNNNNNNNNNNNNNNNNNNNNNNNNNNNNNNNNNNNNNNNNNGTGGCCG
TGGGGGTGGGGCCGGGAGGCGATTTATGGGGCGTGCCTGCGTGGGATGTGTGG
GTGCGTGGCTGACGGACTGGCGGTTGTTACCCCTTTCCCGGGGTTTTTTCCAGTT
CTTTTCTTCTTTTTGTTTTTCGTGTGCTGGAAGGGGACGGAAGGAGGAGTAACTATT
ACGACGGCAAACTCCCCTTTAATAGTACAGATTTAGGTATTCTCACATTCAGATCCT
CTAGATTTGGGCTGTTTAATGCTTTTACTGATAATGATATATACAGATCTTTGATGCT
TTCTATGTGCTCTCAACTCCCAAATAAGGCAATAAATGATGAACAATTCTAGCTGTA
GTTTCTAGTTTGTTCATGAAGTCCTGTAAATAAAGACACGACATATTTACGTTTCGA
ACATTAGCTTGTGGTCGTGCACTGCACACTGCACAGCCTGCCCGCAGTCTTTCAGT
TTACTATTAGTATTCACGAGTATTACTATATGTGTTTAGTTAGTTTTTCTCACTTGTG
TTGTGGATGGATACTCTAGGTTGAATACACAGAATATTTTTCTGGTCAAACATCTTA
TGCAATGGCAGAAGCTCCAAAAGAGGCTCTTCCTCACAATTGCCGGCCTGATTTTG
GTGCTGTATGGTCAACAACAGCAAAGTTCAAGGTACACTACTATGGAACATCTACT
CCCTCCGTTCCAAAATAGATGACTCAACTTTGTACTAACTTTAGTACAAAGTTAGTA
CAAAATTGGGTCATCTATTTTGGAACGGAGGGAGTAGTTCACATGAGAAAGAACAT
CTAGTTCACATGAGAGTATGAGACATACATAATGTTCTCTCCAGTGGTTTTTTGAAT
CTTCAGTTGTTTGCACGCCCTTTGCGTATATCATGTTTAGTTATCAAGAGGTCATCA
TTTTATTATCGTCTTTTCTTGTAGGTCAATGAATCATACAAGTGCAGGTACACACTG
GGGAGTACCAAAGCTGACATTTATTCAGACAAGCTATTCAATTGCACTGCTGAGGA
TCCTTCAATAATTGAAATGCTGAAAAGGATTTTTGTACTGTGAGTATATATGCCCTC
TAATTTTCTACCTGCTGCATGGGCATCGCCTTACCTTCTCATAATGTGATAACATAT
GTTAATCCACATATCCTTAACCAGAAGTCGATAGCCGAAAGTACTCGTTGCTCTTAT
TTGCACTGGTAATCTGACTAGCTCTAGTGCTTTATGCTTATCAATTGGCTTTGTTTC
TTATACTTGGGTTGCATTGTTGCATCACCACTTAATTTCAAGCTGTAAACTCTAGGA

ACTTAAGAATCTGTGGTTGTTTAAAATGGTTACTTTTTCCCTTCAGTTCTTCAAATGG
TCATTTATCCTTAATATTTCCATTTGCTTCTGTTTAACTTGAGATTGAAACATGACAG
GTTTTCAAAGTTCTGCAAGTCAAAAGATTTCAGTTCGTGGTGGATGCTTGGATATGC
GGCAGCAGGGGTCGTGGCGGGCGTGCTGAGCTCCATACTCATCACAATAGCAGT
AAGAATCCTGCGAGGAGTGTTCCTTGCTGCCGCCAGGCGGGCTGTGAGCAACCAT
AGCATCAGGGTGTTTGCTTACCGTTTCAAGCGGGCGTGCCTTCTTGTTGCGTATAT
ATCTTTCGTTGGCTGGATCACCTTGCAGTACAGCAAAATGGTTGGGCTGAAGGAG
CTTGTGCTGGACTTCAAACTCTTGGAGAGGTTCTTGTAAAATGTAAACGGGTTTCG
GCAATGTATGTCAAATAAGCCGATCCCCTTGTGTGATGTAACCTAGTGTAACTTATC
CTGCCTTGCTTCGATATGCAGCCGCCCATGAACAGGTGGATGAATCATGGGTAGC
TAGTAGCTACAGTGGGCATGGAGTAACTCCCATCTGTTGTAACCTGCAATGCAACA
TGTATCACAATCATCGTAATGACAGTTTGGGGGGCTTGGAAATGTGGTCTAACTTT
CTGGCTCTGAATGCGAAATCACGGTTAGGGGCCTCTTCGATACGCTGGAATTTTTC
TCTGAATCAAAGAGGATAGTTATGAACATTTTTTCAAACTGAATTTTTTGATGGGAT
CAGAGTATACCTGGCCAAACCTCGGGCTTTGGGCCGGGCCTAGCCAAGCCTGAG
ACAAAAAATCCAGGCCCGGGCCTGGCCCAGCCCTGGCATTGGGACTGTTTTTGGG
CCCAAGCCCGGCCCAAACGCGTAAAAGCCCGCCGGGCCTTGGGCCCCTTCAGGA
AAATAGCAAAAATGACAGACAAAATCTAGGCCGGGCCCGGCCTGGAAGCAGTGCC
GGGCCGGGCCGGGTCAGGCTATTTCGGGCTGGGCCGTTCGGGCCGGGCTGCCG
ATGGCCAGGACTAGATCAGAGGTACATGCTAGACAAACTATGCCTAAAGCTTTTCA
GTTAAGATAAGAAAAACTCTGCCTGATAATAAAGCTCTTTTACTCCGGAAGCCTTCT
CTGAGCTTCTTGGAATTCAGAAATTTCCTCTGGAACAACAGTACTTCCAATGTACTC
CCTCCGTCCCATAATTTAAGAGTGTTTTTGACACTGCACTAGTGTAAAAAACGCTCT
TATATTATGGGACAGAGGGAGTAGCTCTTATGATGCTTTAATACTTGGAAAAAAATG
GCAGTGCATAAAAACTGTATGTTGCCACTGCATGGTTGTTTCCTGGAAGAAGAAGA
AAAAATGGAGCCGTATACGTTTCTAGAACCCTTTAGACATGGACAGCCATCTCCAG
CAGGACATAAGCAAATGTATTTGATTTCCAAGTCTCCAACTGTTCCACTGAGCACTC
AAGCTCAGCACCTACTTGGCCTCGTCTTCCATTTTGATGCGCCCGCCCACCATGTC
AAACGCTCCCGTTCTCCTGCGCGTGCGTCGGTGCGTGCACGGACCCATGGAATC
TGTCGTCCCCATCATCCCGGCCGCGGCGAAGAATGGTCAAAGTCGCCTCCCCGG
CTGACCCCCAAAGGCGAAGACCAGTGGTATTTCCCAACCAGGTGGCACGAGCACG
GCCGTCCCGTGCGTGGCATCCACGCCACAAGCAGCACTGCAGGTCAAGTTCGCA
CGGCGGTTGTGTCCGGATCGCGCAGCGCAACCGATGGATGGCTCTCGAACTCCC
CCGGAAGCCGGCGACGACGTTCCCCGTCACGAGCTCCTCGGCCACTCCGTTAGG
AATCTTTTACTCCCTCCGTTTCAAATTACTTGTCTGTCAACCAGACAGAGACTTGCA
CGGCAAGCAGTTGATCCTGTTTAGATTATACTCTCTCCGTTTCATAATGTAAGACGT
TTTTTGACACTACATTAGTGTAAAAAAACGTCTTACATTATGAGACAGAGAGGGAGT
ACTTTTTAGCAAAGATTCCATTTAGATTAGACAACTGCTGTGCTGCGCTGCCGCTC
CGCCCAGGAAGCAGCGCGCGCCGGCCGAGCCCGCCGCACTATGCATTATTGTGT
GGTGGGTACAACTGTGTCAAATTAAATATGCACACCTATTTAAACTAAGTAGTCTGT
TTACATCAAAAATAAATAACTAAATTAAGTAGTACTCTGTTAGCAGCCCTTCATGCA
CGAAAATAGTATAGTATGTGTAACAACATATAAGAATATTTTTGTTTTGAACTGAACC
ACAAAAGAATTGGATCATCCATGACAGTTAGCTGTTGGGCGAGTGCGCACATGTCT

TTTTTTCTCCTTCTCTATCCAGGGAGAATGCGCACATGTTGATGTTGTCTAGTGACA
TTCCGGGAAGCACCGTGCCGTGCCGTTCCCGACACGCGAGTTCCCCTATAAAACC
ATCTCCAGCAAAAACGTTTCTTGCCATCACTCAGTTTACCAGATCAACACCAACAGA
AATCACTTCCCAGCAACCAAGACTTCCCATTCGATCCGATCACAAGAAAATATTATA
TCCTCAAGCTTTTCGACCGGCGTATTTTAGTAGTTCAACAGCCAACACCATGAGCT
CTCGGATGGCCGGAGCCACGCTGCTGCGCCGCGCCGCGAGCGCGAGGGGCATC
ATGCCCGCCGCCGCGAGGGTCTTCCCCGCGCGGATGGCCAGCACCGAGGCCGC
CGGCCCGCGTGCCAAGCAAGAAGAAGCCACTGAAAAGCCCCAGGGCGCAACAGC
GCCGGAGCAGAACAAGAAGGCCGTGCCGAGCTACTGGGGTATCGAGCCGCGGAA
GCTCGTCAAGGACGACGGCACGGAGTGGCCGTGGTTCTCCTTCAGGCCGTGGGA
CACGTACAGGCCGGACACGTCCATCGACGTGGCCAAGCACCACGAGCCCAGGGC
GGTGGCGGACAAGGTGGCGTACCTCATCGTGCGGACGCTGCGCAAGGGCAGCGA
CCTCTTCTTCCAGCGCCGGCACGCCAGCCACGCGCTGCTGCTGGAGACGGTGGC
GGCCGTGCCGCCCATGGTGGGCGGCGTGCTGCTGCACCTGCGCTCGCTCCGCC
GCTTCGAGCACAGCGGCGGCTGGATCCGCGCGCTCATGGAGGAGGCCGAGAAC
GAGCGCATGCACCTCATGACCTTCATGGAGGTGACGCAGCCGCTGTGGTGGGAG
CGCGCGCTCGTGCTCGCCACTCAGGGCGTCTTCTTCAACGCCTACTTCGTCGGCT
ACCTCGTCTCCCCCAAGTTCGCGCACCGCTTCGTTGGCTACCTCGAGGAGGAGGC
CGTCCACTCCTACACCGAATACCTCAAGGACCTCGAGGCCGGCTTGATCGAGAAC
ACGCCCGCGCCGGCCATCGCCATAGATTACTGGCGCCTCCCCGCCGACGCCAGG
CTCAAGGACGTCGTCACCGCCGTGCGCGCCGACGAGGCGCATCACCGTGACGCC
AACCACTACGCATCGGACATCCATTACCAGGGAATGACACTGAATCAGACGCCTG
CGCCACTCGGCTACCACTGA

AetAOX:

>*AetAOX1a* gene
ATGCTCGACAAGATCGCCTACTACACCGTCAAGTCCCTGCGCTTCCCCACCGACA
TCTTCTTCCAGGTATGCGCGCCTCCCCCCTCCCTTCCCCTCGCCGGCCTTCACGA
CCGCCGGTTGATCCTCCGGATGGTTCGTTCCGTGTTTTTTTAGTCCAACCCGTTTC
GCCGAAGACGGCACGAACGGCGGTGTCTGCATCTTTGCGTGTCATGGCCTCATGG
GTGACGGAAGTAGCATGGTCCCTGTCGCGCTTTTGATTCTGTTTCCCCCTGCTTTC
CGGGGAGACGGCGTTTGGACTTGCTATTTAGAACGCCGACCCAAGCGGTCTAGCG
GATTATTTTTTACCTAATCGTTCGTGGGAATGGGTGTTTTATATTCTACTGTATCAGA
TTACTGTGGATAATCGCGTGCTGGAGAAATCCGCTGATTATTTCTATCTGGATACAA
GTAGGCGACAGTATCGACAGATTATAGACTGTCAGCAGACATTAGGAATTGAAAAC
ACAAATAACATGGTGACCTTGACATCGCTACTGCATAGGATATTTTATTGATTGTAA
AGCACCAGAGAGGATTCCATTTGTTTTGCCATGTACTTTACTTTATTAAAGCATCTG
TTTATCATGATTTTTGCACAAGTTGTATCATCTTGATCTCCTCTCTGTATTTCGAAGT
TTATCATGATCTGAAGCTGACCCTTCAAATTGGATGCAGAGGAGGTATGGCTGCCG
CGCAATGATGCTGGAGACTGTTGCCGCAGTGCCGGGGATGGTGGGCGGCATGCT
CCTCCACCTGCGCTCGCTCCGGCGCTTCGAGCAGAGCGGCGGCTGGATCCGCGC
GCTGCTGGAGGAGGCCGAGAACGAGCGCATGCACCTCATGACCTTCATGGAGGT
GGCGCAGCCCAGGTGGTACGAGCGCGCCCTCGTCATCGCCGTCCAGGGCGTCTT

CTTCAACGCCTACTTCTTCGGCTACCTCATCTCGCCCAAGTTCGCGCACCGCGTCG
TCGGGTACCTCGAGGAGGAGGCCGTCCACTCCTACACGGAGTTCCTCAAGGACCT
CGACGACGGCAAGATCGACAACGTCCCCGCCCCGGCCATCGCCATCGACTACTG
GCGCCTCCCTGCCAACGCCACCCTCAAGGACGTGGTCACCGTGGTGCGCGCCGA
CGAGGCTCACCACCGCGACGTCAACCACTTCGCATCGGTACGAATCCCACATCCT
TTCTTGACCCCATTTCCTGACCAGTCCATGTGCATCTGTTTTGTGTGGATTGGTTCT
GAATTTTCTGATGATTTTGATCTGTCGCATGTGTCGTGCAGGACGTGTACTACCAG
GGTATGCAGCTGAAGGCCACCCCGGCCCCGATCGGATACCACTGA
>*AetAOX1e* gene
ATGGCGATTTTCTTGCCAACTAAAAGCTGGCGATGTAACTCAATTTTATCTCAAATT
TTCATCGCAACTCAGAGCACCATTCGATCTCATGCAGCTTGGCACTGTGACGAGTT
GGTACACGAAGTAACATCGCACAATCTCTGGTCCACAGAGGATGCGAGCACATGC
ATGCAAAAGGAGAAACAGCGTGGTACACACTCGCTCGCATGCATGCATGCATGCT
CTTCAAGTTACCTCAAAAAAAAAAAAGTTGCCAACTTGGCATGCACTGCATGCAGG
GTCAGCAGCTAGGAAGCACAAGGGCAACTACCTACGTCTCTTCTCTCCCCTCTCC
GCGTATGTATATAAGACCCATCACCGTACACACCGTTTCTGATCTACACCAAAACC
CATACCCATAGGCCCCAAATATACACCAACACTAGTGTCCTTGTCCAGGCTCGGCA
AGAAATGGCCATGGTGCAGTCGGTAGCGCGGCGCGGCGCGAGGGCGGTGGGGC
GCGCTTCTTCTCCGTGGCCGGCCGCTCACCGGCGGCGCTCGGCGTCGGCGCCG
CGCGCACGGCTGCCACGTTAAAGCAAGGGGAGAAGGAGGCGGCGAGCTACTGG
GGCGTGGCGCCGGCGAGGCTCGTCAAGGAGGACGGCACCGAGTGGAAGTGGTC
GTGCTTCAGGGTGCGTACTCGCCCGTGAGCTTGTTGCCTGATTATTCATGGCGTCA
CTTATTATGGCTTGTCGACTGAGCTGCATGCATGGGTGAATGGCGCATCTGCAGC
CGTGGGATGCGTACGAGGCTGACGTGTCCATCGATCTGACGAAGCACCACCGGC
CGGCCACGCTCGGGGACAAGGTGGCCTTGTGGACGGTCAAGGCGATGCGCTGGC
CCACCGACCTCTTCTTCCAGGTAGTACTTCCTCATTCTTCGTTTTGATTAGCACAGT
TTTCTGTCTTAAATGTATTTCCATTTGGTTATTATATACTGACAAGTCAAAGCTGTAT
TCCCTGAATCCGTGGGCGCTTGGCAGCGGACGATCTGGACCGTCCACTGAGTTGA
GGTGGACGAGTTCCGACTAACTAAGCAGAAAATCCTATACGATGCTCACCGCGTC
AAACGACCAAACATGCAGGCCGACCTACTCAGCTCGTGTTTTTTTGTCTCTTCTTCA
TTTTTTCTGGTTTAATAATAATACCTTTGTGACATTTTATGTTTTTTCCTTTTGCATTT
CAGTTTCTACAGGTCCTCTATCTTTTTTTATTTTTCTTTCTTTGTTATTTTTCTTCCAT
TATTCTTATTCTTATTTGGTTTTTTATCCTTTTTTGTTTCTTTCTTTAAGTCTTTTTCCT
ATGATTTATAGAAAGTTAGCTAAACTTTTTTGAGCATGTAATAATTTTTGCATGCATG
AAACATTTCTAAATATGCGATTAAACATTTCATTTCACATACATGATCAGTTTTAAAA
ATTATTAATGTTTCTAAAATTACATAAAAATTATATAGAGGTTTTTTAAAATATTATTG
CATATGGAGGTTTTTTAATTATGTGATATTTTTTCATATGCAGGACATATTAACAAAA
CATGATTTTTTTTAAAAATATAATTTTTTTCTACTGAAACTAATTTTATTTATACATGAA
TAATTTGGTGCTATGCAATAATATTTTATTCATATAAATGAACATTTTTTAGCCGATG
ATTTTTTTGGGAATTTTATAACTAATTTAATACAAGAATAAAATTGTTTGTATACTTTT
TAAAATATGAAATGAATATTTTAGTTCATACCCCTCCGTAAGAAATATAAGAACGTTT
AGATCACTAAAGTAGGGATCTAAACACTCTTATATTTCTTTACGAAGGGAGTACATA
TTAACATTTTTATCTGAATATGTGAACATTTTGTTATTTTACGCAATATAAGTTTTCCC

CATTTTTGAAACTGGTTTTTTGACACGATTATTTAAATGATAGTTTTTCCTTTTTTGCA
AATATATAGTACTTCCATTATTTAATCTTTTATTAATATTTTTCCATAAATCTGTTAACT
TTAACAAATTAAACCTAAGTGGGTGATCTTGTGATGCGTGGAGCGGGTTTAACCAC
CTATTTGATACAACTCTCCACCGACCAACCTGAATTTTCATATCAAATTCGAAGTGA
GACGATTAGAACTCATGTTTTTACGCTACTTGCAGTGGATCGATTGCAAAAAAAACA
TATGTTCAATCGGAACAATTTGTGTACATTTTCCATCGACTTGCCACAGCTAAAAGA
AAAAAGAATTGTACACTTGACTTGCTCATAGTTCTTCTTTGCTTATGTACGCACGCA
CGCAGAGGAGGTACGGTTGCCGCGCCATGATGCTGGAGACGGTGGCCGCAGTCC
CCGGCATGGTGGCTGGCGCGGTGCTCCACCTCCGGTCGCTCCGGCGCTTCGAAC
AGAGCGGCGGGTGGATCCGTGCACTGCTGGAGGAGGCCGAGAACGAGCGCATG
CACCTGATGACCTTCATGGAGGTGTCCCAGCCGCGGTGGTACGAGCGCGCGCTC
GTCGTCGCCGTCCAGGGCGTCTTCTTCCACGCGTACCTCGCCACCTACCTCGCCT
CCCCAAAGGTCGCGCACCGCATGGTGGGGTACCTGGAGGAGGAGGCCGTGCACT
CCTACACCGAGTTCCTCCGTGACCTCGAGGCCGGCAAGATCGACGGCGTGCCCG
CGCCGGCCATCGCCATCGACTACTGGCGCCTCCCGGCCGGCGCGACCCTGAAAG
ATGTCGTCAGGGTTGTCCGCGCCGACGAGGCGCACCACCGGGACGTCAACCACT
ACGCCTCTGTAAGCGTCGCCTCCGTCAAATTTGCCAAGCTGACATCAATTGCAGTC
TTCTTTGTACTGAAATGAGTGCATGTGTGCAGGACATACATTGCCAGGGGCATGCA
CTGCGAGAGGTAGCTGCGCCGATCGGCTACCACTGA
>*AetAOX1d* gene
ATGCCCGCCGCCGCGAGGATCTTCCCCGCGCGGATGGCCAGCACCGAGGCCGC
CGCCCCGCATGCCAAACAAGAAGATGATGCCGCGAGCCCCCAGGCGGCCGCGAC
TCCAGAGCAGCAGAACAAGAAGCCCGTGGTGAGCTATTGGGGCATCGAGCCTCG
GAAGCTCGTCAAGGATGACGGCACGGAGTGGCCGTGGTTCTGCTTCAGGCCGTG
GGACACGTACCGGCCGGACACGTCCATCGACGTGACCAAGCACCACGAGCCCAA
GGCCCTGGCGGACAAGGTGGCGTACTTCGTTGTCCGATCGCTGCGCGTGCCCCG
GGACCTCTTCTTCCAGCGCCGGCACGCCAGCCACGCGCTGCTGCTGGAGACGGT
GGCGGCGGTGCCGCCCATGGTGGGCGGCGTGCTACTTCACCTGCGCTCGCTCCG
CCGCTTCGAGCACAGCGGCGGCTGGATCCGGGCGCTCATGGAGGAGGCCGAGA
ACGAACGCATGCACCTCATGACCTTCATGGACGTGACGCAGCCCCGGTGGTGGG
AGCGCGCGCTCGTGCTCGCCGCGCAGGGCGTCTTCTTCAACGCCTACTTCGTCG
GCTACCTCATCTCCCCCAAGTTCGCGCACCGCTTCGTCGGGTACCTCGAGGAGGA
GGCCGTGGAGTCCTATACTGAGTACCTCAAGGACCTCGAGGCCGGCTTGATCGAG
AACACGCCCGCCCCGGCCATCGCCATCGACTACTGGCGCCTCCCCGCCGACGCC
AGGCTCAAGGACGTCGTCACCGCCGTGCGCGCCGACGAGGCGCATCACCGCGAC
GCCAACCACTACGCATCGGACATCCATTACCAGGGAATGACGCTGAATCAGACGC
CTGCGCCGCTCGGGTACCACTGA
>*AetAOX1d-like* gene
ATGAGCTCTCGGATGGCCGGAGCCACGTTGCTGCGCCACCTGGGCCCCCACCTC
TTCGCCGCCGCCGAGCCGGCGTCCGGGCTCGCCGCGAGCGCGAGGGGTGGCGC
GCATGCCAAACAAGAAGGTGACGCTGAAAAGCCCGAGAGCGCCACAGCGCCGGA
GCAGAACAAGAAGCCCGTGGCGAGCTACTGGGGCATCGAGCCGCGGAAGCTCGT
CAAGGACGACGGCACGGAGTGGCCGTGGTTCTCCTTCAGGCCGTGGGACACGTA

CCGGCCGGACACGTCCATCGACGTGGCCAAGCACCACGAGCCCAGGGCGGTGG
CGGACAAGGTGGCGTACCTCATCGTGCGGACGCTGCGCGCGGGCAGCGACCTCT
TCTTCCAGCGCCGCCACGCCAGCCACGGCGGAAGCTCGTCAAGGACGACGGCAC
GGAGTGGCCGTGGTTCTCCTTCAGGCCGTGGGACACGTACCGGCCGGACACGTC
CATCGACGTGGCCAAGCACCACGAGCCCAGGGCGGTGGCGGACAAGGTGGCGTA
CCTCATCGTGCGGACGCTGCGCGCGGGCAGCGACCTCTTCTTCCAGCGCCGCCA
CGCCAGCCACGCGCTGCTGCTTGAGACGGTGGCGGCGGTGCCGCCCATGGTGG
GCGGCGTGCTGCTGCACCTGCGCTCGCTCCGCCGATTCGAGCACAGCGGCGGCT
GGATCCGCGCGCTCATGGAGGAGGCCGAGAACGAGCGCATGCACCTCATGACCT
TCATGGAGGTGACGCAGCCCCTGTGGTGGGAGCGCGCGCTCGTGCTCGCCACGC
AGGGCGTCTTCTTCAACGCCTACTTCGTCGGCTACCTCATCTCCCCCAAGTTCGCG
CACCNNNNNNNNNNNNNNNNNNNNNNNNNNNNNNNNNNNNNNNNNNNNNNNNNNN
NNNNNNNNNNNNNNNNNNNNNNNNNNNNNNNNNNNNNNNNNNNNNNNNNNNNNN
NNNNNNNNNNNNNNNNNNNNNNNNNNNNNNNNNNNNNNNNNNNNNNNNNNNNNN
NNCCTTTCGCCTCCGCCTGCGGCCCCCCCCCGCCGACGCCAGGCTCAAGGACG
TCGTCATCGCCGTGCGCGCCGACGAGGCGCATCACCGCGACGCCAACCACTACG
CATCGGACATCCATTACCAGGGAATGACGCTGAATCAGACGCCTGCGCCGCTCGG
CTACCACTGA

AesAOX:

>*ne.AesAOX1d* gene
ATGAGCTCTCGGATGGCCGGAGCCACGCTTCTGCGCCACCTGGGTCCCCGCCTC
TTCGCCGCCGCCGAGCCGGCCTCCGGGCTCGCCGCGAGCGCGAGGGGCATCAT
GCCCGCCGCCGCGAGGATCTTCCCCGCGCGGATGGCCAGCACCGAGGCCGCCG
GGCCGCATGCCAAACAAGAAAGTGACGCTGAAAAGCCCGAGAGCGCCGCGACGC
CGGAGCAGCAGAACAAGAAGCCCGTGGTGAGCTACTGGGGCATCGAGCCGCGGA
AGCTCGTCAAGGAGGACGGCACGGAGTGGCCATGGTTCTGCTTCAGGCCGTGGG
ACACGTACCGGCCGGACACGTCCATCGACGTGACCAAGCACCACGAGCCCAAGG
CCCTGGCGGACAAGGTGGCCTACTTCGTCGTCAGGTCGCTGCGCGTGCCCCGGG
ACCTCTTCTTCCAGCGGCGGCACGCGAGCCACGCGCTGCTGTTGGAGACGGTGG
CGGCCGTGCCCCCGATGGTGGGCGGCGTGTTGCTGCACCTGCGCTCGCTCCGCC
GCTTCGAGCACAGCGGCGGCTGGATCCGGGCGCTTATGGAGGAGGCCGAGAACG
AGCGCATGCACCTCATGACCTTCATGGAGGTGACGCAGCCGCGGTGGTGGGAGC
GCGCGCTCGTGCTCGCCGCGCAGGGCGTCTTCTTCAACGCCTACTTCGTCGGGTA
CCTCATCTCCCCCAAGTTCGCTCACCGCTTCGTCGGCTACCTCGAGGAGGAAGCC
GTGGAGTCTTATACTGAGTACCTCAAGGACCTAGAGGCCGGCTTGATCGAGAACA
CGCCCGCGCCGGCCATCGCCATCGACTACTGGCGCCTCCCCGCCGACGCCAGGC
TCAAGGACGTCGTCACCGCCGTGCGCGCCGACGAGGCGCATCACCGCGACGCCA
ACCACTATGCATCAGACATCCATTACCAGGGAATGACGCTGAATCAGACGCCTGC
GCCGCTCGGGTACCACTGA

CODING SEQUENCES

TaAOX:

>*TaAOX1a-2AL.sv1* CDS
ATGAGCTCCCGGATGGCCGGATCGGTCCTCCTCCGCCGCGCCGGCGCCGGCGC
CGGCCGCCTCTTCGCCACCACCGCGTCCCCGGCGGCCAGGACCGCCCTCGGTG
GAGGTGAGGGCGCGTGGGTGCGGATGATGTCCACCTCCGCGGCCTCGCAGGTCA
AGGATGAGGCGGCCAAGGGGGTCAAGGCGGAGGCGGCCAAGGGCGACGGGGA
GAAGAAGGAGGTGGCCATCAGCAGCTACTGGGGGATCGAGCAGTCGAAGAAGCT
GGTGCGCGAGGACGGCACCGAGTGGAAGTGGTCTTGCTTCAGGCCATGGGAGAC
GTACACCGCTGACACGTCGATCGATCTGACCAAGCACCACGTGCCCAACACGATG
CTCGACAAGATCGCCTACTACACCGTCAAGTCCCTGCGCTTCCCCACCGACATCTT
CTTCCAGAGGAGGTACGGCTGCCGCGCAATGATGCTGGAGACTGTTGCCGCCGT
GCCGGGGATGGTGGGCGGCATGCTCCTGCACCTGCGCTCCCTCCGGCGCTTCGA
GCAGAGCGGCGGCTGGATCCGCGCGCTGCTGGAGGAGGCCGAGAACGAGCGCA
TGCACCTCATGACCTTCATGGAGGTGGCGCAGCCGAGGTGGTACGAGCGCGCCC
TCGTCATCGCCGTCCAGGGCGTCTTCTTCAACGCCTATTTCTTCGGCTACCTCATC
TCGCCCAAGTTCGCACACCGCGTCGTCGGGTACCTCGAGGAGGAGGCCGTCCAC
TCCTACACCGAGTTCCTCAAGGACCTCGACGACGGCAAGATCGACAACGTCCCCG
CCCCTGCCATCGCCATCGACTACTGGCGCCTCCCTGCCAACGCCACCCTCAAGGA
CGTGGTCACCGTGGTCCGCGCCGACGAGGCTCACCACCGCGACGTCAACCACTT
CGCATCGGACGTGTACTACCAGGGTATGCAGCTGAAGGCCACCCCGGCGCCGAT
CGGATACCACTGA
>*TaAOX1a-2AL.sv2* CDS
ATGAGCTCCCGGATGGCCGGATCGGTCCTCCTCCGCCGCGCCGGCGCCGGCGC
CGGCCGCCTCTTCGCCACCACCGCGTCCCCGGCGGCCAGGACCGCCCTCGGTG
GAGGTGAGGGCGCGTGGGTGCGGATGATGTCCACCTCCGCGGCCTCGCAGGTCA
AGGATGAGGCGGCCAAGGGCGACGGGGAGAAGAAGGAGGTGGCCATCAGCAGC
TACTGGGGGATCGAGCAGTCGAAGAAGCTGGTGCGCGAGGACGGCACCGAGTGG
AAGTGGTCTTGCTTCAGGCCATGGGAGACGTACACCGCTGACACGTCGATCGATC
TGACCAAGCACCACGTGCCCAACACGATGCTCGACAAGATCGCCTACTACACCGT
CAAGTCCCTGCGCTTCCCCACCGACATCTTCTTCCAGAGGAGGTACGGCTGCCGC
GCAATGATGCTGGAGACTGTTGCCGCCGTGCCGGGGATGGTGGGCGGCATGCTC
CTGCACCTGCGCTCCCTCCGGCGCTTCGAGCAGAGCGGCGGCTGGATCCGCGCG
CTGCTGGAGGAGGCCGAGAACGAGCGCATGCACCTCATGACCTTCATGGAGGTG
GCGCAGCCGAGGTGGTACGAGCGCGCCCTCGTCATCGCCGTCCAGGGCGTCTTC
TTCAACGCCTATTTCTTCGGCTACCTCATCTCGCCCAAGTTCGCACACCGCGTCGT
CGGGTACCTCGAGGAGGAGGCCGTCCACTCCTACACCGAGTTCCTCAAGGACCTC
GACGACGGCAAGATCGACAACGTCCCCGCCCCTGCCATCGCCATCGACTACTGG
CGCCTCCCTGCCAACGCCACCCTCAAGGACGTGGTCACCGTGGTCCGCGCCGAC
GAGGCTCACCACCGCGACGTCAACCACTTCGCATCGGACGTGTACTACCAGGGTA
TGCAGCTGAAGGCCACCCCGGCGCCGATCGGATACCACTGA
>*TaAOX1a-2BL* CDS
ATGCCAGTAGTCACAGGCCGTTTCCACGCGGGAGGAAAAAAAAAAGAGGAGGAAG
AAAATCGCAACGACCCATTAATGCGATCGATCGACTTGGAGGAGAAGAAAAGACC
CCCTCAGTTCCTGGGATCCCCGGGAGGCAGGGCCGGCTCCCCCGCCACTGACGC

CGCGGCCCCACCCCTGCCCTCGCCGCCTCCGCCATCGATCCTCCTTCCCAATATA
AAATCCATCCGCGGAAAGACCCTTCCATTCGCAAACCGCAACCCCCCAACCCCAC
CCCCACCCACCCCCACAGCAACCAACGCAGGCGAAGGCGCGCGTCGACGTCACG
CCCACGACCCGTCCCGGAGTTTTGGGGTGTTCGCGGAGGCGTTTCCCGGCGCCC
AGATGAGCTCCCGGATGGCCGGATCGGTCCTCCTCCGCCGCGCCGGCGCTGGCG
CCAGCCGCCTCTTCGCCACCACCCCGACGTCCCCGGCGGCCAGGACCGCCCTCG
CCGGCGGCGACGGCGCGTGGGTGCGGATGATGTCCACCTCCGCGGCCTCGCAG
GTCAAGGACGAGGCGGCTAAGGCGGTCAAGGCGGAGGCGGCCAAGGGCGACGG
GGAGAAGAAGGAGGTGGCGATCAGCAGCTACTGGGGGATCGAGCAGTCGAAGAA
GCTGGTGCGCGAGGACGGCACCGAGTGGAAGTGGTCTTGCTTCAGGCCATGGGA
GACGTACACCGCGGACACGTCGATCGATCTGACCAAGCACCACGTGCCCAACACG
ATGCTCGACAAGATCGCCTACTACACCGTCAAGTCCCTGCGCTTCCCCACCGACA
TCTTCTTCCAGAGGAGGTATGGCTGCCGCGCAATGATGCTGGAGACTGTTGCCGC
AGTGCCGGGGATGGTGGGCGGCATGCTCCTCCACCTGCGCTCGCTCCGGCGCTT
TGAGCAGAGCGGTGGTTGGATCCGCGCGCTGCTGGAGGAGGCCGAGAACGAGC
GCATGCACCTCATGACCTTCATGGAGGTGGCGCAGCCGAGGTGGTACGAGCGCG
CCCTCGTCATCGCCGTCCAGGGCGTCTTCTTCAACGCCTACTTCTTCGGCTACCTC
ATCTCGCCCAAGTTCGCGCACCGCGTCGTCGGGTACCTCGAGGAGGAAGCCGTC
CACTCCTACACCGAGTTCCTCAAGGACCTTGACGACGGCAAGATCGACAACGTCC
CCGCCCCGGCCATCGCCATCGACTACTGGCGCCTCCCTGCCAACGCCACCCTCAA
GGACGTGGTCACCGTGGTGCGCGCCGACGAGGCTCACCACCGCGACGTCAACCA
CTTCGCATCGGACGTGTACTACCAGGGTATGCAGCTGAAGGCCACCCCAGCGCC
GATCGGATACCACTGA
>*TaAOX1a-2DL.sv1* CDS
ATGAGCTCACGGATGGCCGGATCGGTCCTCCTCCGCCGCGCCGGCGCCGGCGCC
AGCCGCCTCTTCGCCACCACCCCGTCTCCGGCGGCCAGGGCCGTCCTCGGTGGA
GGTGAGGGCGCGTGGGTGCGGCTGATGTCCACCTCCGCGGCCTCGCAGGTCAAG
GACGAGGCGGCCAAGGCGGTCAAGGCGGAGGCGGCCAAGGCGGTCAAGGCGGA
GGCGGCCAAGGGCGACGGGGAGAAGAAGGAGGTGGCCATCAGCAGCTACTGGG
GGATCGAGCAGTCGAAGAAGCTGGTGCGCGAGGACGGCACCGAGTGGAAGTGGT
CTTGCTTCAGGCCATGGGAGACGTACACCGCGGACACGTCGATCGATCTGACCAA
GCACCACGTGCCCAACACGATGCTCGACAAGATCGCCTACTACACCGTCAAGTCC
CTGCGCTTCCCCACCGACATCTTCTTCCAGAGGAGGTATGGCTGCCGCGCAATGA
TGCTGGAGACTGTTGCCGCAGTGCCGGGGATGGTGGGCGGCATGCTCCTCCACC
TGCGCTCGCTCCGGCGCTTCGAGCAGAGCGGCGGCTGGATCCGCGCGCTGCTGG
AGGAGGCCGAGAACGAGCGCATGCACCTCATGACCTTCATGGAGGTGGCGCAGC
CCAGGTGGTACGAGCGCGCCCTCGTCATCGCCGTCCAGGGCGTCTTCTTCAACGC
CTACTTCTTCGGCTACCTCATCTCGCCCAAGTTCGCGCACCGCGTCGTCGGGTAC
CTCGAGGAGGAGGCCGTCCACTCCTACACGGAGTTCCTCAAGGACCTCGACGAC
GGCAAGATCGACAACGTCCCCGCCCCGGCCATCGCCATCGACTACTGGCGCCTC
CCTGCCAACGCCACCCTCAAGGACGTGGTCACCGTGGTGCGCGCCGACGAGGCT
CACCACCGCGACGTCAACCACTTCGCATCGGACGTGTACTACCAGGGTATGCAGC
TGAAGGCCACCCCGGCCCCGATCGGATACCACTGA

>*TaAOX1a-2DL.sv2* CDS
ATGAGCTCACGGATGGCCGGATCGGTCCTCCTCCGCCGCGCCGGCGCCGGCGCC
AGCCGCCTCTTCGCCACCACCCCGTCTCCGGCGGAGGCGGCCAAGGGCGACGG
GGAGAAGAAGGAGGTGGCCATCAGCAGCTACTGGGGGATCGAGCAGTCGAAGAA
GCTGGTGCGCGAGGACGGCACCGAGTGGAAGTGGTCTTGCTTCAGGCCATGGGA
GACGTACACCGCGGACACGTCGATCGATCTGACCAAGCACCACGTGCCCAACACG
ATGCTCGACAAGATCGCCTACTACACCGTCAAGTCCCTGCGCTTCCCCACCGACA
TCTTCTTCCAGAGGAGGTATGGCTGCCGCGCAATGATGCTGGAGACTGTTGCCGC
AGTGCCGGGGATGGTGGGCGGCATGCTCCTCCACCTGCGCTCGCTCCGGCGCTT
CGAGCAGAGCGGCGGCTGGATCCGCGCGCTGCTGGAGGAGGCCGAGAACGAGC
GCATGCACCTCATGACCTTCATGGAGGTGGCGCAGCCCAGGTGGTACGAGCGCG
CCCTCGTCATCGCCGTCCAGGGCGTCTTCTTCAACGCCTACTTCTTCGGCTACCTC
ATCTCGCCCAAGTTCGCGCACCGCGTCGTCGGGTACCTCGAGGAGGAGGCCGTC
CACTCCTACACGGAGTTCCTCAAGGACCTCGACGACGGCAAGATCGACAACGTCC
CCGCCCCGGCCATCGCCATCGACTACTGGCGCCTCCCTGCCAACGCCACCCTCAA
GGACGTGGTCACCGTGGTGCGCGCCGACGAGGCTCACCACCGCGACGTCAACCA
CTTCGCATCGGACGTGTACTACCAGGGTATGCAGCTGAAGGCCACCCCGGCCCC
GATCGGATACCACTGA
>*TaAOX1a-like-2DL* CDS
ATGGTGGGCGGCGTGCTGCTGCACCTGCGCTCGCTCCGCCGCTTCGAGCACAGC
GGCGGCTGGATCCGCGCGCTCATGGAGGAGGCCGAGAACGAGCGCATGCACCTC
ATGACCTTCATGGAGGTGACCCAGCCCCTGTGGTACGAGCGCGCCCTCGTCATCG
CCGTCCAGGGCGTCTTCTTCAACGCCTACTTCTTCGGCTACCTCATTTCCCCCAAG
TTCGCGCACCGCGTCGTCGGCTACCTCGAGGAGGAGGCCGTCCACTCCTACACC
GAGTTCCTCAAGGACCTCGACGACGGCAAGATCGACAACGTCCCCGCCTCGGCC
ATCGCCATCGACTACTGGCGCCTCCCTGCCAACGCCACCCTCAAGGCCGTGGTCA
CCGTGGTGCGCGCCGACGAGGCTCACCACCGCGACGTCAACCACTTCGCATCGG
ACGTGTACTACCAGGGTATGCAGCTGAAGGCCACCCCCGCGCCGATCGGATACCA
CTGA
>*regTaAOX-4BL.sv1* CDS
ATGGTTAGAAGAAGGAGGTGGCGATCAGCAGCTACTTGGGGGATCGAGCAGTCG
AAGAAGCTGGTGCGTGAGGAAGGCACCGAGTGGAAGTGGTCTTGCTTCAGGCCAT
GGGAGGCGTACAGCGCAGACATGTCGATCGATCTGACCAAGCACCATGTGCCCAA
CACGATGCTCGACAAGATCGCCTACTACACCGTCAAGTCCCCGCGCTTCCCCACC
GACATCTTCTTCCAGGTACGCATGCTTCCTGGTGTGGCGCCCCTGCTACATTCACG
AGTGCAAGCGTCACGTGGCCGTGAGCAGGCCAGTTCCACGCCAACAAGATGA
>*regTaAOX-4BL.sv2* CDS
ATGGTTAGAAGAAGGAGGTGGCGATCAGCAGCTACTTGGGGGATCGAGCAGTCG
AAGAAGCTGGTGCGTGAGGAAGGCACCGAGTGGAAGTGGTCTTGCTTCAGGCCAT
GGGAGGCGTACAGCGCAGACATGTCGATCGATCTGACCAAGCACCATGTGCCCAA
CACGATGCTCGACAAGATCGCCTACTACACCGTCAAGTCCCCGCGCTTCCCCACC
GACATCTTCTTCCAGGTACTCTTCTGTTAA
>*regTaAOX-4BL.sv3* CDS

ATGGTTAGAAGAAGGAGGTGGCGATCAGCAGCTACTTGGGGGATCGAGCAGTCG
AAGAAGCTGGTGCGTGAGGAAGGCACCGAGTGGAAGTGGTCTTGCTTCAGGTTG
GTTTCACTGCGGCAGCCATGGGAGGCGTACAGCGCAGACATGTCGATCGATCTGA
CCAAGCACCATGTGCCCAACACGATGCTCGACAAGATCGCCTACTACACCGTCAA
GTCCCCGCGCTTCCCCACCGACATCTTCTTCCAGGTACTCTTCTGTTAA
>*regTaAOX-4BL.sv4* CDS
ATGGTTAGAAGAAGGAGGTGGCGATCAGCAGCTACTTGGGGGATCGAGCAGTCG
AAGAAGCTGGTGCGTGAGGAAGGCACCGAGTGGAAGTGGTCTTGCTTCAGGCCAT
GGGAGGCGTACAGCGCAGACATGTCGATCGATCTGACCAAGCACCATGTGCCCAA
CACGATGCTCGACAAGATCGCCTACTACACCGTCAAGTCCCCGCGCTTCCCCACC
GACATCTTCTTCCAGGTACGCATGCTTCCTGGTGTGGCGCCCCTGCTACATTCACG
AGTGCAAGCGTCACGTGGCCGTGAGCAGGCCAGTTCCACGCCAACAAGATGA
>*TaAOX1c-6AL* CDS
ATGCCATCGTGGCGCGCGCTGGCTCGGCGACAGCGACACGTCATCCCGTCACCC
TCTCAGAGCTTGGCACGTCCGCAGGTTCTCGAGCCTGCGACCACGAGTTTCGCGA
GCAGAGCGGCAGCCCACCAAGCAGGCTCGTCATCTTCGGCGATGAGCTCCCGCG
TCGCCGGATCCGTCCTCCTCCGCCACCTGGGCCCGCGCGTCTTCGGGCCGACCA
CTCCGGCCGCGCAGAGGCCCCTGCTTGCCGGAGGGGAAGGGGGCGCCGTGGCC
GTGGCCATGTGGGCGCGGCCGCTGTCCACCTCCGCCGCCGAGGCGGCGAGGGA
GGAGGCGACCGCGTCCAAGGACAACGTGGCGAGCACCGCCGCCGCGACGGCCG
AGGCGATGCAGGCCGCCAAGGCCGACGCTGTGCAGGCCGCGAAGGAGGGCAAG
AGCCCCGCGGCGAGCAGCTACTGGGGCATCGTGCCTGCCAAGCTGGTGAACAAG
GACGGCGCCGAGTGGAAGTGGTCTTGCTTCAGGCCGTGGGAGGCGTACACGTCG
GACACGACCATCGATCTCTCCAAGCACCACAAGCCCAAGGTGCTGCTCGACAAGA
TCGCCTACTGGACCGTCAAGTCGCTGCGCGTGCCCACCGACATCTTCTTCCAGCG
GAGGTACGGGTGCCGGGCGATGATGCTGGAGACGGTGGCGGCGGTGCCGGGGA
TGGTGGGCGGGATGCTGCTGCACCTGCGGTCGCTGCGGCGGTTCGAGCAGAGCG
GCGGGTGGATCCGGGCGCTGCTGGAGGAGGCGGAGAACGAGCGGATGCACCTG
ATGACCTTCATGGAGGTGGCCAACCCCAAGTGGTACGAGCGCGCGCTGGTGCTG
GCGGTGCAGGGCGTCTTCTTCAACGCCTACTTCCTGGGGTACATCGTGTCCCCCA
AGTTCGCGCACCGCGTCGTGGGCTACCTGGAGGAGGAGGCCATCCACTCCTACA
CCGAGTTCCTCCGCGACCTGGAGGCCGGCAGGATCGAGAACGTCCCCGCCCCGC
GCATCGCCATCGACTACTGGCGCCTCCCCGCCGACGCCAGGCTCAAGGACGTCG
TCACCGTCGTGCGCGCCGACGAGGCGCACCACCGCGATGTCAACCACTTCGCCG
CGGACATCCATTTCCAGGGGCTGGAGCTCAACAAGACGCCTGCCCCGCTAGGATA
TCACTGA
>*TaAOX1c-6BL.sv1* CDS
ATGGACGAGCGCACGCAGAAGCTGTCCACCAGTCAATTGCGAGCTCGTAAATACT
CTACCAGCCAAGCAGAGCCGCCGTTCATCCCACGTCTCGCGTCGTCTGCTCGTAG
CGCCACGCCATCGCGGCGCGCGCTGGCTCGGCGACACGTCGTCAAGTCACCCTC
TCAGAGCTTGGCACGTCCGCAGGTTCGCGAGCCTACGACCACGAGTTTCGCGAG
CAGAGCGGCAGCCCACCAAGCAGGCTCGTCATCTTCGGCGATGAGTTCCCGCGT
CGCCGGATCCGTCCTCCTCCGCCACCTGGGCCCGCGCGTCTTCGGGCCGACCAC

TCCTGCTGCGCAGAGGCCCCTGCTTGCCGGAGGAGAAGGGGGCGCCGTGGTCGT
GTGGGCGCGGCCGCTGTCCACCTCCGCCGCAGAGGCGGCGAGGGAGGAGGCGG
CCGCGTCCAAGGACAACGTGGCGAGCACCGCCGCCGCGACGGCCGAGGCGATG
CAGGCCGCGAAGGCCCAGGCCGTGCAGGCCGCCAAGGAGGGGGGCAAGAGCCC
AGTGAGCAGCTACTGGGGCATCGTGCCTGCCAAGCTGGTGAACAAGGACGGCGC
CGAGTGGAAGTGGTCTTGCTTCAGGCCGTGGGAGGCGTACACGTCGGACACGAC
GATCGATCTCACCAAGCACCACAAGCCCAAGGTGCTGCTCGACAAGATCGCCTAC
TGGACCGTCAAGTCGCTGCGCGTGCCCACCGACATCTTCTTCCAGAGGAGGTACG
GGTGCCGGGCGATGATGCTGGAGACGGTGGCGGCGGTGCCGGGGATGGTGGGC
GGGATGCTGCTCCACCTGCGGTCGCTGCGGCGGTTCGAGCAGAGCGGCGGGTG
GATCCGGGCGCTGCTGGAGGAGGCAGAGAACGAGCGGATGCACCTGATGACCTT
CATGGAGGTGGCCAAACCCAAGTGGTACGAGCGCGCGCTGGTGCTGGCGGTGCA
GGGCGTCTTCTTCAACGCCTACTTCCTGGGCTACATCGTGTCCCCCAAGTTTGCGC
ACCGCGTCGTCGGCTACCTCGAGGAGGAGGCCATCCACTCCTACACCGAGTTCCT
CCGCGACCTCGAGGCCGGCAGGATCGAGAACGTCCCCGCCCCGCGCATCGCCAT
CGACTACTGGCGCCTCCCCGCCGACGCCAGGCTCAAGGACGTGGTCACCGTCGT
GCGCGCCGACGAGGCGCACCACCGCGACGTCAACCACTTCGCCGCGGACATCCA
TTTCCAGGGGCTGGAGCTCAACAAGACGCCTGCCCCGCTAGGATATCACTGA
>*TaAOX1c-6BL.sv2* CDS
ATGGACGAGCGCACGCAGAAGCTGTCCACCAGTCAATTGCGAGCTCGTAAATACT
CTACCAGCCAAGCAGAGCCGCCGTTCATCCCACGTCTCGCGTCGTCTGCTCGTAG
CGCCACGCCATCGCGGCGCGCGCTGGCTCGGCGACACGTCGTCAAGTCACCCTC
TCAGAGCTTGGCACGTCCGCAGGTTCGCGAGCCTACGACCACGAGTTTCGCGAG
CAGAGCGGCAGCCCACCAAGCAGGCTCGTCATCTTCGGCGATGAGTTCCCGCGT
CGCCGGATCCGTCCTCCTCCGCCACCTGGGCCCGCGCGTCTTCGGGCCGACCAC
TCCTGCTGCGCAGAGGCCCCTGCTTGCCGGAGGAGAAGGGGGCGCCGCGGCCG
CGTCCAAGGACAACGTGGCGAGCACCGCCGCCGCGACGGCCGAGGCGATGCAG
GCCGCGAAGGCCCAGGCCGTGCAGGCCGCCAAGGAGGGGGGCAAGAGCCCAGT
GAGCAGCTACTGGGGCATCGTGCCTGCCAAGCTGGTGAACAAGGACGGCGCCGA
GTGGAAGTGGTCTTGCTTCAGGCCGTGGGAGGCGTACACGTCGGACACGACGAT
CGATCTCACCAAGCACCACAAGCCCAAGGTGCTGCTCGACAAGATCGCCTACTGG
ACCGTCAAGTCGCTGCGCGTGCCCACCGACATCTTCTTCCAGAGGAGGTACGGGT
GCCGGGCGATGATGCTGGAGACGGTGGCGGCGGTGCCGGGGATGGTGGGCGGG
ATGCTGCTCCACCTGCGGTCGCTGCGGCGGTTCGAGCAGAGCGGCGGGTGGATC
CGGGCGCTGCTGGAGGAGGCAGAGAACGAGCGGATGCACCTGATGACCTTCATG
GAGGTGGCCAAACCCAAGTGGTACGAGCGCGCGCTGGTGCTGGCGGTGCAGGG
CGTCTTCTTCAACGCCTACTTCCTGGGCTACATCGTGTCCCCCAAGTTTGCGCACC
GCGTCGTCGGCTACCTCGAGGAGGAGGCCATCCACTCCTACACCGAGTTCCTCCG
CGACCTCGAGGCCGGCAGGATCGAGAACGTCCCCGCCCCGCGCATCGCCATCGA
CTACTGGCGCCTCCCCGCCGACGCCAGGCTCAAGGACGTGGTCACCGTCGTGCG
CGCCGACGAGGCGCACCACCGCGACGTCAACCACTTCGCCGCGGACATCCATTT
CCAGGGGCTGGAGCTCAACAAGACGCCTGCCCCGCTAGGATATCACTGA
>*TaAOX1c-6BL.sv3* CDS

ATGGACGAGCGCACGCAGAAGCTGTCCACCAGTCAATTGCGAGCTCGTAAATACT
CTACCAGCCAAGCAGAGCCGCCGTTCATCCCACGTCTCGCGTCGTCTGCTCGTAG
CGCCACGCCATCGCGGCGCGCGCTGGCTCGGCGACACGTCGTCAAGTCACCCTC
TCAGAGCTTGGCACGTCCGCAGGTTCGCGAGCCTACGACCACGAGTTTCGCGAG
CAGAGCGGCAGCCCACCAAGCAGGCTCGTCATCTTCGGCGATGAGTTCCCGCGT
CGCCGGATCCGTCCTCCTCCGCCACCTGGGCCCGCGCGTCTTCGGGCCGACCAC
TCCTGCTGCGCAGAGGCCCCTGCTTGCCGGAGGAGAAGGGGGCGCCGTGGTCGT
GTGGGCGCGGCCGCTGTCCACCTCCGCCGCAGAGGCGGCGAGGGAGGAGGCGG
CCGCGTCCAAGGACAACGTGGCGAGCACCGCCGCCGCGACGGCCGAGGCGATG
CAGGCCGCGAAGGCCCAGGCCGTGCAGGCCGCCAAGGAGGGGGGCAAGAGCCC
AGTGAGCAGCTACTGGGGCATCGTGCCTGCCAAGCTGGTGAACAAGGACGGCGC
CGAGTGGAAGTGGTCTTGCTTCAGGCCGTGGGAGGCGTACACGTCGGACACGAC
GATCGATCTCACCAAGCACCACAAGCCCAAGGTGCTGCTCGACAAGATCGCCTAC
TGGACCGTCAAGTCGCTGCGCGTGCCCACCGACATCTTCTTCCAGAGGAGGTACG
GGTGCCGGGCGATGATGCTGGAGACGGTGGCGGCGGTGCCGGGGATGGTGGGC
GGGATGCTGCTCCACCTGCGGTCGCTGCGGCGGTTCGAGCAGAGCGGCGGGTG
GATCCGGGCGCTGCTGGAGGAGGCAGAGAACGAGCGGATGCACCTGATGACCTT
CATGGAGGTGGCCAAACCCAAGTGGTACGAGCGCGCGCTGGTGCTGGCGGTGCA
GGGCGTCTTCTTCAACGCCTACTTCCTGGGCTACATCGTGTCCCCCAAGTTTGCGC
ACCGCGTCGTCGGCTACCTCGAGGAGGAGGCCATCCACTCCTACACCGAGTTCCT
CCGCGACCTCGAGGCCGGCAGGATCGAGAACGTCCCCGCCCCGCGCATCGCCAT
CGACTACTGGCGCCTCCCCGCCGACGCCAGGCTCAAGGACGTGGTCACCGTCGT
GCGCGCCGACGAGGCGCACCACCGCGACGTCAACCACTTCGCCGCGGACATCCA
TTTCCAGGGGCTGGAGCTCAACAAGACGCCTGCCCCGCTAGGATATCACTGA
>*TaAOX1c-6DL* CDS
ATGCCATCGTGGCGCGCGCTAGCTCGGCGACACCGACACGTCATCCCGTCACCCT
CTCGGAGCTTGGCACGTCCACAGGTTCTCGATCCTGCGACCACGAGTTTCGCGAG
CAGAGCGGCAGCTCACCAAGCAGGCTCGCCATCTTCGGCGATGAGTTCCCGCGT
CGCCGGATCCGTCCTCCTCCGCCACCTGGGCCCGCGCGTCTTCGGGCCGACCAC
TCAGGCTGCGCAGAGGACCCTGCTTGCCGGAGGGGAAGGGGGCGCCGTGGCCA
TGTGGGCGTGGCCGCTGTCCACCTCCGCCGCCGAGGCGGCGAGGGAGGAGGCG
GCCGCGTCCAAGGACAACGTGGCGAGCACCGCCGCCGCGACGGCCGAGGCGAT
GCAGGCCGCGAAGGCCGAGGCGGTGCAGGCCGCCAAGGAGGGGGGCAAGAGC
CCGGCGAGCAGCTACTGGGGCATCGTGCCTGCCAAGCTGGTGAACAAGGACGGC
GCCGAGTGGAAGTGGTCTTGCTTCAGGCCGTGGGAGGCGTACACGTCGGACACG
ACGATCGATCTCACCAAGCACCACAAGCCCAAGGTGCTGCTCGACAAGATCGCCT
ACTGGACCGTCAAGTCGCTGCGCGTGCCCACCGACATCTTCTTCCAGAGGAGGTA
CGGGTGCCGGGCGATGATGCTGGAGACGGTGGCGGCGGTGCCGGGGATGGTGG
GCGGGATGCTGCTGCACCTGCGGTCGCTGCGGCGGTTCGAGCAGAGCGGCGGC
TGGATCCGGGCGCTGCTGGAGGAGGCGGAGAACGAGCGGATGCACCTGATGACC
TTCATGGAGGTGGCCAACCCCAAGTGGTACGAGCGCGCGCTGGTGCTGGCGGTG
CAGGGCGTCTTCTTCAACGCCTACTTCCTGGGCTACATCGTGTCCCCCAAGTTCGC
GCACCGCGTCGTCGGCTACCTGGAGGAGGAGGCCATCCACTCCTACACCGAGTT

CCTCCGCGACCTGGAGGACGGCAGGATCGAGAACGTCCCCGCCCCGCGTATCGC
CATCGACTACTGGCGCCTCCCGCCCGACGCCAGGCTCAAGGACGTCGTCACCGT
CGTGCGCGCCGACGAGGCGCACCACCGCGACGTCAACCACTTCGCCGCGGACAT
CCATTTCCAGGGGCTGGAGCTCAACAAGACGCCTGCCCCGCTAGGATATCACTGA
>*regTaAOX-3B* CDS
ATGCACCTCATGACCTTCATGGAGGTGTCCCAGCCGCGGTGGTACGAGCGCGCG
CTCGTGGTCGCCGTCCAGGGCGTCTTCTTCCACGCCTACCTCGCCACCTACCTCG
CCTCCCCAAAGGTCGCGCACCGCATGGTGGGGTACCTGGAGGAGGAGGCCGTGC
ACTCCTACACCGAGTTCCTTCGTGACCTCGAGGCCGGCAAGATCGACGACGTGCC
CGCGCCGACGAGGCGCACCACCGGGACGTCAAACCACTACGCCTCCGACATACA
TTGCCAGGGGCATGCACTGCGAGAGGTAGCTGCGCCGATCGGCTACCACTGA
>*TaAOX1d-2AL.1* CDS
ATGCCCGCCGCCGCGAGGATCTTCCCCGCGCGGATGGCCAGCACCGAGGCCGC
CGCCCCGCATGCCAAACAAGAAGAAGCCACCGAAAAGCCCCAGGGCGCAACAAC
GCCGGAGCACAACAAGAAGGCCGTGGTGAGCTACTGGGGCATCGAGCCGCGGAA
GCTCGTGAAGGACGACGGCACGGAGTGGACGTGGTTCTCCTTCAGGCCGTGGGA
CACCTACCGCCCGGACACGTCCATCGACATGGCCAAGCACCACGAGCCCAGGGC
GGTGGCGGACAAGGTGGCGTACCTCATCGTGCGGACGCTGCGCGCGGGCAGCG
ACCTCTTCTTCCAGCGCCGGCACGCCAGCCACGCGCTGCTGCTGGAGATGGTGG
CGGCGGTGCCGCCCATGGTGGGCGGCGTGCTGCTGCACCTGCGCTCGCTCCGC
CGCTTCGAGCACAGCAGCGGCTGGATCCGCGCGCTCATGGAGGAGGCCGAGAAC
GAGCGCATGCACCTCATGACCTTCATGGAGGTGACGCAGCCGCTGTGGTGGGAG
CGCGCGCTCGTGCTCGCCACTCAGGGCGTCTTCTTCAACGCCTACTTCGTCGGCT
ACCTCGTCTCCCCCAAGTTCGCGCACCGCTTCGTTGGCTACCTCGAGGAGGAGGC
CGTCCACTCCTACACCAAATACCTCAAGGACCTCGAGGCCGGCTTGATCGAGAAC
ACGCCCGCGCCGGCCATCGCCATAGATTACTGGCGCCTCCCCGCCGACGCCAGG
CTCAAGGACGTCGTCACCGCCGTGCGCGCCGACGAGGCGCATCACCGTGACGCC
AACCACTACGCATCGGACATCCATTACCAGGGAATGACGCTGAATCAGACGCCTG
CGCCACTCGGCTACCACTGA
>*TaAOX1d-2AL.2.sv1* CDS
ATGAGCTCCCGGATGGCCGGAGCCACGCTTCTGCGCCACCTGGGCCCCCGCCTC
TTCGCCGCCGCCGAGCCAGCCTCCGGGCTCGCCGCGAGCGCGAGGGGCATCAT
GCCCGCCGCCGCGAGGATCTTCCCCGCGCGGATGGCCAGCACCGAGGCCGCCG
CCCCGCATGCCAAACAAGAAGATGATGCCGCGAGCCCCCAGGCGGCCGCGACTC
CAGAGCAGCAGAACAAGAAGCCCGTGGTGAGCTACTGGGGCATCGAGCCTCGGA
AGCTCGTCAAGGATGACGGCACGGAGTGGCCATGGTTCTGCTTCAGGCCGTGGG
ACACGTACCGGCCGGACACGTCCATCGAAGTGGCCAAGCACCACGAGCCCAAGG
CCCTGGCGGACAAGGTGGCCTACTTCGTGGTTCGGTCGCTGCGCGTGCCCCGGG
ACCTCTTCTTCCAGCGCCGGCACGCCAGCCATGCTCTGCTACTGGAAACGGTGGC
GGCGGTGCCTCCCATGGTGGGCGGCGTGCTGCTGCACCTGCGCTCGCTCCGCCG
CTTCGAGCACAGCGGCGGCTGGATCCGGGCGCTCATGGAGGAGGCCGAGAACGA
GCGCATGCACCTCATGACCTTCATGGAGGTGACGCAGCCGCGGTGGTGGGAGCG
CGCGCTCGTGCTCGCCGCGCAGGGCGTCTTCTTCAACGCCTACTTCGTCGGGTAC

CTCATTTCCCCCAAGTTCGCGCACCGCTTCGTCGGGTACCTCGAGGAGGAGGCCG
TGGAGTCTTATACTGAGTATCTCAAGGACCTTGAGGCCGGATTGATCGAGAACACG
CCCGCGCCGGCCATCGCCATCGACTACTGGCGCCTCCCCGCCGACGCCAGGCTC
AAGGACGTCGTCACCGCCGTGCGCGCCGACGAGGCGCATCACCGCGACGCCAAC
CACTACGCATCGGACGTCCATTACCAGGGAATGACGCTGAATCAATCGCCTGCGC
CGCTCGGGTACCACTGA

>*TaAOX1d-2AL.2.sv2* CDS

ATGAGCTCCCGGATGGCCGGAGCCACGCTTCTGCGCCACCTGGGCCCCCGCCTC
TTCGCCGCCGCCGAGCCAGCCTCCGGGCTCGCCGCGAGCGCGAGGGGCATCAT
GCCCGCCGCCGCGAGGATCTTCCCCGCGCGGATGGCCAGCACCGAGGCCGCCG
CCCCGCATGCCAAACAAGAAGATGATGCCGCGAGCCCCCAGGCGGCCGCGACTC
CAGAGCAGCAGAACAAGAAGCCCGTGGTGAGCTACTGGGGCATCGAGCCTCGGA
AGCTCGTCAAGGATGACGGCACGGAGTGGCCATGGTTCTGCTTCAGGCCGTGGG
ACACGTACCGGCCGGACACGTCCATCGAAGTGGCCAAGCACCACGAGCCCAAGG
CCCTGGCGGACAAGGTGGCCTACTTCGTGGTTCGGTCGCTGCGCGTGCCCCGGG
ACCTCTTCTTCCAGCGCCGGCACGCCAGCCATGCTCTGCTACTGGAAACGGTGGC
GGCGGTGCCTCCCATGGTGGGCGGCGTGCTGCTGCACCTGCGCTCGCTCCGCCG
CTTCGAGCACAGCGGCGGCTGGATCCGGGCGCTCATGGAGGAGGCCGAGAACGA
GCGCATGCACCTCATGACCTTCATGGAGGTGACGCAGCCGCGGTGGTGGGAGCG
CGCGCTCGTGCTCGCCGCGCAGGGCGTCTTCTTCAACGCCTACTTCGTCGGGTAC
CTCATTTCCCCCAAGTTCGCGCACCGCTTCGTCGGGTACCTCGAGGAGGAGGCCG
TGGAGTCTTATACTGAGTATCTCAAGGACCTTGAGGCCGGATTGATCGAGAACACG
CCCGCGCCGGCCATCGCCATCGACTACTGGCGCCTCCCCGCCGACGCCAGGCTC
AAGGACGTCGTCACCGCCGTGCGCGCCGACGAGGCGCATCACCGCGACGCCAAC
CACTACGCATCGGACGTCCATTACCAGGGAATGACGCTGAATCAATCGCCTGCGC
CGCTCGGGTACCACTGA

>*TaAOX1d-2DL* CDS

ATGGCCGGAGCCACGCTTCTGCGCCACCTGGGCCCCCGCCTCTTCGCCGCCGCC
GAGCCAGCCTCCGGGCTCGCCGCGAGCGCGAGGGGCATCATGCCCGCCGCCGC
GAGGATCTTCCCCGCGCGGATGGCCAGCACCGAGGCCGCCGCCCCGCATGCCAA
ACAAGAAGATGATGCCGCGAGCCCCCAGGCGGCCGCGACTCCAGAGCAGCAGAA
CAAGAAGCCCGTGGTGAGCTATTGGGGCATCGAGCCTCGGAAGCTCGTCAAGGAT
GACGGCACGGAGTGGCCGTGGTTCTGCTTCAGGCCGTGGGACACGTACCGGCCG
GACACGTCCATCGACGTGACCAAGCACCACTTGCCCAAGGCCCTGGCGGACAAG
GTGGCGTACTTCGTTGTCCGATCGCTGCGCGTGCCCCGGGACCTCTTCTTCCAGC
GCCGGCACGCCAGCCACGCGCTGCTGCTGGAGACGGTGGCGGCGGTGCCGCCC
ATGGTGGGCGGCGTGCTACTTCACCTGCGCTCGCTCCGCCGCTTCGAGCACAGC
GGCGGCTGGATCCGGGCGCTCATGGAGGAGGCCGAGAACGAACGCATGCACCTC
ATGACCTTCATGGAGGTGACGCAGCCCCGGTGGTGGGAACGCGCGCTCGTGCTC
GCCGCGCAGGGCGTCTTCTTCAACGCCTACTTCGTCGGCTACCTCATCTCCCCCA
AGTTCGCGCACCGCTTCGTCGGGTACCTCGAGGAGGAGGCCGTGGAGTCCTATA
CTGAGTACCTCAAGGACCTCGAGGCCGGCTTGATCGAGAACACGCCCGCCCCGG
CCATCGCCATCGACTACTGGCGCCTCCCCGCCGACGCCAGGCTCAAGGACGTCG

TCACCGCCGTGCGCGCCGACGAGGCGCATCACCGCGACGCCAACCACTACGCAT
CGGACATCCATTACCAGGGAATGACGCTGAATCAGACGCCTGCGCCGCTCGGGTA
CCACTGA

>put.regTaAOX-3B
ATGCCATGGGAGACGTACACCGCGGACATGTCGATCGATCTGACCAAGCACCACG
TGCCCAACACGATGCTCGACAAGATCGCCTACTACACCGTCAAGTCCCTGCGCTT
CCCCACCGACATCTTCTTCCAGGACGAGCTGCAGCAGGACAACCTGGAGCGGAA
GAACTTCGAGGGCAAGATCAAGGAGAACCAGGAGACGATCACCGGTTACCTCATC
CTCGTCGCCATGCTTCGCTTCTTTGGCAGTCCCCTCTTTGGCCCCGACCAACTTAC
TTCTGTCGCTGGCTAG
>put.regTaAOX-6BL
ATGGAGGCGTTTCTCGGCGGCCAGATGAGCTCTCGGATGGCCGGATCGGTCCTC
CTCCGCCGCGCCGGCGCTGGCGCTAGCCGCCTCTTCTCCACCACCACGATGTCC
CCAGGGGCCAGGACCTTCCTCGCCGGCGGCAAGGGCACGTGGGTGCGGATGAT
GTCCACTTCTGCGGCCTCGCAGGTCAAGGACGAGGCGGCTAAGGTGGTCAAGGC
GGAGGCGGCCAAGGGCGATGGCAACATGGTGACTCTAGTGCAGGCAGCGTTGGC
CATCCTCTTCGAGGACGAGCTGCAGCAGGACAACCTGGAGCGGAAGAACTTCGAG
GGCAAGATCAAGGAGAACCAGGAGACGATCACCGGTTACCTCATCCTCGCCGGCA
TGCTTGGCTCCTTTGACAGACCCCTCTTTGGCTCCGACCAACTTACTTTTGTCGCT
GGCTAG
>put.TaAOX1e-3DS
ATGGCTGCCACGTTAAAGAAAGGGGAGGAGGAGGCGGCGAGCTACTGGGGCGTG
GCGCCGGCGAGGCTCGTCAAGGAGGACGGCACCGAGTGGAAGTGGTCGTGCTTC
AGGCCGTGGGATGCGTACGAGGCCGACGTGTCCATCGTTCTGACGAAGCACCAC
CGGCCGGCCACGTTCGGGGACAAGGTGGCCTTGTGGACGGTCAAGGCGATACGC
TGGCCCACGGACCTCTTCTTCCAGAGGAGGTACGGTTGCCGCGCGATGATGCTGG
AGACGGTGGCCGCAGTCCCCGGCATGGTGGCTCGCGCGGTGCTCCACCTCCGGT
CGCTCCGGCGCTTCGAGCAGAGCGGCGAGTGGATCCGTGCACTGCTGGAGGAGG
CGCAGAACGAGCGCATGCACCTCATGACCTTCATGGAGGTGTCCCAGCCGCGGT
GGTACGAGCGCGCGCTCGTCGTCGTCGTCCAGGGCGTCTTCTTCCACGCCTACCT
CGCCACCTACCTCGCCTCCCCAAAGGTCGCGCACCGCATGGTGGGGTACCTGGA
GGAGGAGGCCGTGCACTCCTACACCGAGTTCCTCCGTGACCTCGAGGCCGGCAA
GATCGACGACGTGCCCGCGCCGGCCATCGCCATCGACTACTGGCGCCTCCCGGC
CGGCGCGACCCTGAAAGATGTCGTCAGGGTTGTCCGCGCCGACGAGGCGCACCA
CCGGGACGTCAACCACTACGCCTCTGACATACATTGCCAGGGGCATGCACTGCGA
GAGGTAGCTGCGCCGATCGGCTACCACTGA
>put.TaAOX1d-like-4AS
ATGCCCACCACCGCGAGGATCTTCCCCGCGCGGATGGCCAGCACTGCCGCAGGC
CCGCATGCCAAACAAGAAGAAGCCACTGGAAAGCCCCAGGGCGCAACAACGCCG
GAGCAGAACAAGAAGGCCGTGCCGAGCTACTGGGGCATCAAGCCGCGGAAGCTC
GTCGAGGACGACGGCACGGAGTGGTCGTGGTTCTCCTTCAGGCCGTGGGACACC
GACCTCTTCTTCCAGCGCCGGCACGCCAGCCACATGCTGCTGCTGGAGACGGTC

GCGGCGGTGCCACCCATGGTGGGCGGCGTGCTGCTGCACCTGCGCTCGCTCCGC
CGCTTCGAGCACAACGGCGGCTGGATCCGCGCGCTCATGGAGGAGGCCCAGAAC
GAGCGCATGCATCTCATGACCTTCATGGAGGTGACGCAGCCCTTGTGGTGCGAGC
GCGCGCTCGTGCTCCCCACCCAGGGTGTCTTTTTCAACGCCTACTTCATCGGGTA
CCTCGTCTCCCCCAAGTTCGCGCACCGCTTCGTCGGCTACCTCGAGGAGGAGGC
CGTACACTGA

>*ne.TaAOX1d-2DL* CDS

ATGAGCTCTCGGATGGCCGGAGCCACGTTGCTGCGCCACCTGGGCCCCCACCTC
TTCGCCGCCGCCGAGCCGGCGTCCGGGCTCGCCGCGAGCGCGAGGGGTATCCT
GCCCGCCGCCGCGAGGATCTTCCCCGCGCGGATGGCCAGCACCGCCGCCGGCG
CGCATGCCAAACAAGAAGGTGACGCTGAAAAGCCCGAGAGCGCCACAGCGCCGG
AGCAGAACAAGAAGCCCGTGGCGAGCTACTGGGGCATCGAGCCGCGGAAGCTCG
TCAAGGACGACGGCACGGAGTGGCCGTGGTTCTCCTTCAGGCCGTGGGACACGT
ACCGGCCGGACACGTCCATCGACGTGGCCAAGCACCACGAGCCCAGGGCGGTG
GCGGACAAGGTGGCGTACCTCATCGTGCGGACGCTGCGCGCGGGCAGCGACCTC
TTCTTCCAGCGCCGCCACGCTAGCCACGCGCTGCTGCTTGAGACGGTGGCGGCG
GTGCCGCCCATGGTGGGCGGCGTGCTGCTGCACCTGCGCTCGCTCCGCCGATTC
GAGCACAGCGGCGGCTGGATCCGCGCGCTCATGGAGGAGGCCGAGAACGAGCG
CATGCACCTCATGACCTTCATGGAGGTGACGCAGCCCCTGTGGTGGGAGCGCGC
GCTCGTGCTCGCCACGCAGGGCGTCTTCTTCAACGCCTACTTCGTCGGCTACCTC
ATCTCCCCCAAGTTCGCGCACCGCTTCGTCGGCTACCTCGAGGAGGAGGCCGTCC
ACTCCTACACCGAGTACCTCAAGGACCTTGAGGCCGGCTTGATCGAGAACACGCC
CGCGCCGGCCATTGCCATCGACTACTGGCGCCTCCCCGCCGACGCCAGGCTCAA
GGACGTCGTCATCGCCGTGCGCGCCGACGAGGCGCATCACCGCGACGCCAACCA
CTACGCATCGGACATCCATTACCAGGGAATGACGCTGAATCAGACGCCTGCGCCG
CTCGGCTACCACTGA

>*ne.TaAOX1d-2BL.1* CDS

ATGAGCTCTCGGATGGCCGGAGCCACGCTGCTGCGCCACCTGGGCCCCCGCCTC
TTTGCCGCCGCCGAGCCGGCCTCCGGGCTCGCCGCCAGCGCGAGGGGCATCAT
GCCCGCCGCCGCGAGGATCTTCCCCGCGCGGATGGCCAGCACCGAGGCCGCCG
CCCCGCATGCCAAACAAGAAGATGATGCCGGAACACCCCAGGCGGCCGCGACTC
CAGAGCAGCAGAGCAAGAAGGCCGTGGTGAGCTACTGGGGCATCGAGCCGCGGA
AGCTCGTCAAGGAGGACGGCACGGAGTGGCCGTGGTTCTGCTTCAGGCCGTGGG
ACACGTACCGGCCGGACACGTCCATCGACGTCACCAAGCACCACGAGCCCAAGG
CCCTGGCGGACAAGGTGGCCTACTTCGTGGTTCGGTCGCTGCGTGTGCCGCGGG
ACCTCTTCTTCCAGCGCCGGCACGCCAGCCACGCGCTGCTGCTGGAGACTGTGG
CGGCGGTGCCGCCCATGGTGGGCGGCGTGCTGCTCCACCTGCGCTCGCTCCGCC
GATTCGAGCACAGCGGCGGCTGGATCCGGGCGCTCATGGAGGAGGCCGAGAAC
GAGCGCATGCACCTCATGACCTTCATGGAGGTGACGCAGCCGCGCTGGTGGGAG
CGCGCGCTCGTGCTCGCCGCGCAGGGCGTCTTCTTCAACGCCTACTTCGTCGGCT
ACCTCATCTCCCCCAAGTTCGCGCACCGCTTCGTCGGCTACCTCGAGGAGGAGGC
CGTGGAGTCTTACACTGAGTACCTCAAGGACCTCGAAGCCGGCTTGATCGAGAAC

ACGCCCGCGCCGGCCATCGCCATCGACTACTGGCGCCTCCCCGCCGACGCCAGG
CTCAAAGACGTCGTCACCGCCGTGCGCGCCGACGAGGCGCATCACCGCGACGCC
AACCACTACGCATCGGACATCCATTACCAGGGAATGACGCTGAATCAGACGCCTG
CGCCGCTCGGGTACCACTGA

>*ne.TaAOX1d-2BL.2* CDS
ATGAGCTCTCGGATGGCCGGAGCCACGCTGCTGCGCCACCTGGGCCCCCGCCTC
TTCGCCGCCGCCGAGCCGGCGTCCGGGCTCGCCGCGAGCGCGAGGGGCATCAT
GCCCGCCGCCGCGAGGATCTTCCCCGCGCGGATGGCCAGCACAGAGGCTGCCG
GCCCGCGTGCCAAACAAGAAGAAGCCACTGAAAAGCCCCAGGGCGCAACAACGC
CGGAGCAGAACAAGAAGGCCGTGGTGAGCTACTGGGGCATCGAGCCGCGGAAGC
TCGTCAAGGACGACGGCACGGAGTGGCCGTGGTTCTCCTTCAGGCCGTGGGACA
CGTACCGGCCGGACACGTCCATCGACGTGGCCAAGCACCACGAGCCCAGGGCGG
TGGCGGACAAGGTGGCGTACCTCATCGTGCGGACGCTGCGCAAGGGAAGCGACC
TCTTCTTCCAGCGCCGGCATGCGAGCCACGCCTTGCTGCTGGAGACGGTGGCCG
CGGTGCCGCCCATGGTGGGCGGCGTGCTGCTGCACCTGCGCTCGCTCCGCCGCT
TCGAGCACAGCGGCGGCTGGATCCGCGCGCTCATGGAGGAGGCCGAGAACGAG
CGCATGCACCTCATGACCTTCATGGAGGTGACGCAGCCGCTGTGGTGGGAGCGC
GCGCTCGTGCTCGCCACTCAGGGCGTCTTCTTCAACGCCTACTTCGTCGGCTACC
TCATCTCCCCAAAGTTCGCGCACCGCTTCGTCGGCTACCTCGAGGAGGAGGCCGT
CCACTCCTACACCGAGTACCTCAAGGACCTCGAGGCCGGCTTGATCGAGAACACG
CCCGCGCCGGCCATCGCCATCGACTACTGGCGCCTCCCCGCCGACGCCAGGCTC
AAAGACGTCGTCATCGCCGTGCGCGCCGACGAGGCGCATCACCGCGACGCCAAC
CACTACGCATCGGACATCCATTACCAGGGAATGACGCTGAATCAGACGCCTGCGC
CGCTCGGGTACCACTGA

TuAOX:
>*TuAOX1a* CDS
ATGCTCGACAAGATCGCCTACTACACCGTCAAGTCCCTGCGCTTCCCCACCGACA
TCTTCTTCCAGAGGAGGTATGGCTGCCGCGCAATGATGCTGGAGACTGTCGCCGC
AGTGCCGGGGATGGTGGGCGGCATGCTCCTCCACCTGCGCTCCCTCCGGCGCTT
CGAGCAGAGCGGCGGGTGGATCCGCGCGCTGCTGGAGGAGGCCGAGAACGAGC
GCATGCATCTCATGACCTTCATGGAGGTGGCGCAGCCGAGGTGGTACGAGCGCG
CCCTCGTCATCGCCGTCCAGGGCGTCTTCTTCAACGCCTACTTCTTCGGCTACCTC
ATCTCGCCCAAGTTCGCGCACCGCGTCGTCGGGTACCTGGAGGAGGAGGCCGTC
CACTCCTACACCGAGTTCCTCAAGGACCTCGACGACGGCAAGATCGACAACGTCC
CCGCCCCGGCCATCGCCATCGACTACTGGCGCCTCCCTGCCAACGCCACCCTCAA
GGACGTGGTCACCGTGGTCCGCGCCGACGAGGCTCACCACCGCGACGTCAACCA
CTTCGCATCGGACGTGTACTACCAGGGTATGCAGCTGAAGGCCACCCCGGCGCC
GATCGGATACCACTGA
>*TuAOX1c* CDS
ATGACCCAAAGTTTCAACAATGAGGCATACATGCCAACTATGGGTGTTGGCTTCAA
CAATTCGCATTGGTCTCAAATAAATGACATGCATCTCGATGACCATGAGTTCGAGG

TGGACGAGGATGGTGAGGGCATTGTCGATGCACCGAAAGGAAGAGGAGGCAACT
ACACCAATGAAGAAGACGTCTTGCTATGCAATACTTGGTTGCAAGTGTCGAGGGAT
CCATCCGTTGGAGATTGTCAAAAGTGGGTGGCCGCACAAATGGCGGTTGACAAGT
TGAATTCAAGTGGCATTAATGATGAAGATAGGCGTGATGGTATGGATGATTTGGAT
ATGAGCAACAAACACATGCAAACAATTGATTTGGATGAGGAGGAGGAGGAGGCAT
CAAGTGATGACGGCAAGAGAAGCCCCACACCCAACTCGGTTTCATACTCGAAGCC
AAAACGACTGGATGTGTGCAAGAAAGACGCAAAAGAAAAGAAGAAGAGGAAAAGA
GATGATGAGCTAAAAAATGCTATGAAAACTATTGTGAAGGGAAGAAAAGAAGCGAA
CGAGGTGAGGAAGATGGCAAGGAACCAAGATGCCGCGGCCGAGGAGAGGAAGGT
GACATTGGAGGAGAGGAAGCCGTGGGAGGCGTACACGTCGGACACGACCATCGA
TCTCTCCAAGCACCACAAGCCCAAGGTGCTGCTCGACAAGATCGCCTACTGGACC
GTCAAGTCGCTGCGCGTGCCCACCGACATCTTCTTCCAGCGGAGGTACGGGTGCC
GGGCGATGATGCTGGAGACGGTGGCGGCGGTGCCGGGGATGGTGGGCGGGATG
CTGCTGCACCTGCGGTCGCTGCGGCGGTTCGAGCAGAGCGGCGGGTGGATCCG
GGCGCTGCTGGAGGAGGCGGAGAACGAGCGGATGCACCTGATGACCTTCATGGA
GGTGGCCAACCCCAAGTGGTACGAGCGCGCGCTGGTGCTGGCGGTGCAGGGCG
TCTTCTTCAACGCCTACTTCCTGGGGTACATCGTGTCCCCCAAGTTCGCGCACCGC
GTCGTGGGCTACCTGGAGGAGGAGGCCATCCACTCCTACACCGAGTTCCTCCGC
GACCTGGAGGCCGGCAGGATCGAGAACGTCCCCGCCCCGCGCATCGCCATCGAC
TACTGGCGCCTCCCCGCCGACGCCAGGCTCAAGGACGTCGTCACCGTCGTGCGC
GCCGACGAGGCGCACCACCGCGACGTCAACCACTTCGCCGCGGACATCCATTTC
CAGGGGCTGGAGCTCAACAAGACGCCTGCCCCGCTAGGATATCACTGA
>*TuAOX1d.1* CDS
ATGCCCGCCGCCGCGAGGATCTTCCCCGCGCGGATGGCCAGCACCGAGGCCGC
CGCCCCGCATGCCAAACAAGAAGATGATGCCGCGAGCCCCCAGGCGGCCGCGAC
TCCAGAGCAGCAGAACAAGAAGCCCGTGGTGAGCTACTGGGGCATCGAGCCTCG
GAAGCTCGTCAAGGATGACGGCACGGAGTGGCCATGGTTCTGCTTCAGGCCGTG
GGACACGTACCGGCCGGACACGTCCATCGACGTGGCCAAGCACCACGAGCCCAA
GGCCCTGGCGGACAAGGTGGCCTACTTCGTGGTTCGGTCGCTGCGCGTGCCCCG
GGACCTCTTCTTCCAGCGCCGGCACGCCAGCCATGCTCTGCTACTGGAAACGGTG
GCGGCGGTGCCTCCCATGGTGGGCGGCGTGCTGCTGCACCTGCGCTCGCTCCGC
CGCTTCGAGCACAGCGGCGGCTGGATCCGGGCGCTCATGGAGGAGGCCGAGAA
CGAGCGCATGCACCTCATGACCTTCATGGAGGTGACGCAGCCGCGGTGGTGGGA
GCGCGCGCTCGTGCTCGCCGCGCAGGGCGTCTTCTTCAACGCCTACTTCGTCGG
GTACCTCATTTCCCCCAAGTTCGCGCACCGCTTCGTCGGGTACCTCGAGGAGGAG
GCCGTGGAGTCTTATACTGAGTATCTCAAGGACCTTGAGGCCGGATTGATCGAGA
ACACGCCCGCGCCGGCCATCGCCATCGACTACTGGCGCCTCCCCGCCGACGCCA
GGCTCAAGGACGTCGTCACCGCCGTGCGCGCCGACGAGGCGCATCACCGCGAC
GCCAACCACTACGCATCGGACGTCCATTACCAGGGAATGACGCTGAATCAATCGC
CTGCGCCGCTCGGGTACCACTGA
>*TuAOX1d.2* CDS
ATGGCCAGGAAACCCGTGGGCGGCGCGGCAGCGGCGGCGCCTGCGCCCCCACG
GGCCGCCCCGGGCAGAGGGGCGGACCCAGCGGAGCGGCGCAGGCGGTGGTGC

GGGATCACAGTCCGCGGGGCCCTCGTGATGCTCTTCCCCATCGCCGTGTCATTCC
TCTTCTCCTTTATCTTCGGCATCGCTGGCCTCCTCCTCGGCGGGCTCTCCTCCAAC
GCGTCCGTCTCCATGCCCTCCACCTGCCGCATCCTCTCTACCGCCAACACCATGA
GCTCTCGGATGGCCGGAGCCACGCTGCTGCGCCGCGCCGCGAGCGCGAGGGGC
ATCATGCCCGCCGCCGCGAGGGTCTTCCCCGCGCGGATGGCCAGCACCGAGGCC
GCCGGCCCGCGTGCCAAGCAAGAAGAAGCCACTGAAAAGCCCCAGGGCGCAACA
GCGCCGGAGCAGAACAAGAAGGCCGTGCCGAGCTACTGGGGTATCGAGCCGCG
GAAGCTCGTCAAGGACGACGGCACGGAGTGGCCGTGGTTCTCCTTCAGGCCGTG
GGACACGTACAGGCCGGACACGTCCATCGACGTGGCCAAGCACCACGAGCCCAG
GGCGGTGGCGGACAAGGTGGCGTACCTCATCGTGCGGACGCTGCGCAAGGGCA
GCGACCTCTTCTTCCAGCGCCGGCACGCCAGCCACGCGCTGCTGCTGGAGACGG
TGGCGGCCGTGCCGCCCATGGTGGGCGGCGTGCTGCTGCACCTGCGCTCGCTCC
GCCGCTTCGAGCACAGCGGCGGCTGGATCCGCGCGCTCATGGAGGAGGCCGAG
AACGAGCGCATGCACCTCATGACCTTCATGGAGGTGACGCAGCCGCTGTGGTGG
GAGCGCGCGCTCGTGCTCGCCACTCAGGGCGTCTTCTTCAACGCCTACTTCGTCG
GCTACCTCGTCTCCCCCAAGTTCGCGCACCGCTTCGTTGGCTACCTCGAGGAGGA
GGCCGTCCACTCCTACACCGAATACCTCAAGGACCTCGAGGCCGGCTTGATCGAG
AACACGCCCGCGCCGGCCATCGCCATAGATTACTGGCGCCTCCCCGCCGACGCC
AGGCTCAAGGACGTCGTCACCGCCGTGCGCGCCGACGAGGCGCATCACCGTGAC
GCCAACCACTACGCATCGGACATCCATTACCAGGGAATGACACTGAATCAGACGC
CTGCGCCACTCGGCTACCACTGA

AetAOX:
>*AetAOX1a* CDS
ATGCTCGACAAGATCGCCTACTACACCGTCAAGTCCCTGCGCTTCCCCACCGACA
TCTTCTTCCAGAGGAGGTATGGCTGCCGCGCAATGATGCTGGAGACTGTTGCCGC
AGTGCCGGGGATGGTGGGCGGCATGCTCCTCCACCTGCGCTCGCTCCGGCGCTT
CGAGCAGAGCGGCGGCTGGATCCGCGCGCTGCTGGAGGAGGCCGAGAACGAGC
GCATGCACCTCATGACCTTCATGGAGGTGGCGCAGCCCAGGTGGTACGAGCGCG
CCCTCGTCATCGCCGTCCAGGGCGTCTTCTTCAACGCCTACTTCTTCGGCTACCTC
ATCTCGCCCAAGTTCGCGCACCGCGTCGTCGGGTACCTCGAGGAGGAGGCCGTC
CACTCCTACACGGAGTTCCTCAAGGACCTCGACGACGGCAAGATCGACAACGTCC
CCGCCCCGGCCATCGCCATCGACTACTGGCGCCTCCCTGCCAACGCCACCCTCAA
GGACGTGGTCACCGTGGTGCGCGCCGACGAGGCTCACCACCGCGACGTCAACCA
CTTCGCATCGGACGTGTACTACCAGGGTATGCAGCTGAAGGCCACCCCGGCCCC
GATCGGATACCACTGA
>*AetAOX1e* CDS
ATGGCGATTTTCTTGCCAACTAAAAGCTGGCGATGTAACTCAATTTTATCTCAAATT
TTCATCGCAACTCAGAGCACCATTCGATCTCATGCAGCTTGGCACTGTGACGAGTT
GGTACACGAAGTAACATCGCACAATCTCTGGTCCACAGAGGATGCGAGCACATGC
ATGCAAAAGGAGAAACAGCGTGGCTCGGCAAGAAATGGCCATGGTGCAGTCGGTA
GCGCGGCGCGGCGCGAGGGCGGTGGGGCGCGCTTCTTCTCCGTGGCCGGCCGC

TCACCGGCGGCGCTCGGCGTCGGCGCCGCGCGCACGGCTGCCACGTTAAAGCAA
GGGGAGAAGGAGGCGGCGAGCTACTGGGGCGTGGCGCCGGCGAGGCTCGTCAA
GGAGGACGGCACCGAGTGGAAGTGGTCGTGCTTCAGGCCGTGGATGCGTACGA
GGCTGACGTGTCCATCGATCTGACGAAGCACCACCGGCCGGCCACGCTCGGGGA
CAAGGTGGCCTTGTGGACGGTCAAGGCGATGCGCTGGCCCACCGACCTCTTCTTC
CAGAGGAGGTACGGTTGCCGCGCCATGATGCTGGAGACGGTGGCCGCAGTCCCC
GGCATGGTGGCTGGCGCGGTGCTCCACCTCCGGTCGCTCCGGCGCTTCGAACAG
AGCGGCGGGTGGATCCGTGCACTGCTGGAGGAGGCCGAGAACGAGCGCATGCA
CCTGATGACCTTCATGGAGGTGTCCCAGCCGCGGTGGTACGAGCGCGCGCTCGT
CGTCGCCGTCCAGGGCGTCTTCTTCCACGCGTACCTCGCCACCTACCTCGCCTCC
CCAAAGGTCGCGCACCGCATGGTGGGGTACCTGGAGGAGGAGGCCGTGCACTCC
TACACCGAGTTCCTCCGTGACCTCGAGGCCGGCAAGATCGACGGCGTGCCCGCG
CCGGCCATCGCCATCGACTACTGGCGCCTCCCGGCCGGCGCGACCCTGAAAGAT
GTCGTCAGGGTTGTCCGCGCCGACGAGGCGCACCACCGGGACGTCAACCACTAC
GCCTCTGACATACATTGCCAGGGGCATGCACTGCGAGAGGTAGCTGCGCCGATC
GGCTACCACTGA
>*AetAOX1d* CDS
ATGCCCGCCGCCGCGAGGATCTTCCCCGCGCGGATGGCCAGCACCGAGGCCGC
CGCCCCGCATGCCAAACAAGAAGATGATGCCGCGAGCCCCCAGGCGGCCGCGAC
TCCAGAGCAGCAGAACAAGAAGCCCGTGGTGAGCTATTGGGGCATCGAGCCTCG
GAAGCTCGTCAAGGATGACGGCACGGAGTGGCCGTGGTTCTGCTTCAGGCCGTG
GGACACGTACCGGCCGGACACGTCCATCGACGTGACCAAGCACCACGAGCCCAA
GGCCCTGGCGGACAAGGTGGCGTACTTCGTTGTCCGATCGCTGCGCGTGCCCCG
GGACCTCTTCTTCCAGCGCCGGCACGCCAGCCACGCGCTGCTGCTGGAGACGGT
GGCGGCGGTGCCGCCCATGGTGGGCGGCGTGCTACTTCACCTGCGCTCGCTCCG
CCGCTTCGAGCACAGCGGCGGCTGGATCCGGGCGCTCATGGAGGAGGCCGAGA
ACGAACGCATGCACCTCATGACCTTCATGGACGTGACGCAGCCCCGGTGGTGGG
AGCGCGCGCTCGTGCTCGCCGCGCAGGGCGTCTTCTTCAACGCCTACTTCGTCG
GCTACCTCATCTCCCCCAAGTTCGCGCACCGCTTCGTCGGGTACCTCGAGGAGGA
GGCCGTGGAGTCCTATACTGAGTACCTCAAGGACCTCGAGGCCGGCTTGATCGAG
AACACGCCCGCCCCGGCCATCGCCATCGACTACTGGCGCCTCCCCGCCGACGCC
AGGCTCAAGGACGTCGTCACCGCCGTGCGCGCCGACGAGGCGCATCACCGCGAC
GCCAACCACTACGCATCGGACATCCATTACCAGGGAATGACGCTGAATCAGACGC
CTGCGCCGCTCGGGTACCACTGA
>*AetAOX1d-like* CDS
ATGAGCTCTCGGATGGCCGGAGCCACGTTGCTGCGCCACCTGGGCCCCCACCTC
TTCGCCGCCGCCGAGCCGGCGTCCGGGCTCGCCGCGAGCGCGAGGGGTGGCGC
GCATGCCAAACAAGAAGGTGACGCTGAAAAGCCCGAGAGCGCCACAGCGCCGGA
GCAGAACAAGAAGCCCGTGGCGAGCTACTGGGGCATCGAGCCGCGGAAGCTCGT
CAAGGACGACGGCACGGAGTGGCCGTGGTTCTCCTTCAGGCCGTGGGACACGTA
CCGGCCGGACACGTCCATCGACGTGGCCAAGCACCACGAGCCCAGGGCGGTGG
CGGACAAGCCACGGCGGAAGCTCGTCAAGGACGACGGCACGGAGTGGCCGTGG
TTCTCCTTCAGGCCGTGGGACACGTACCGGCCGGACACGTCCATCGACGTGGCCA

AGCACCACGAGCCCAGGGCGGTGGCGGACAAGGTGGCGTACCTCATCGTGCGGA
CGCTGCGCGCGGGCAGCGACCTCTTCTTCCAGCGCCGCCACGCCAGCCACGCGC
TGCTGCTTGAGACGGTGGCGGCGGTGCCGCCCATGGTGGGCGGCGTGCTGCTGC
ACCTGCGCTCGCTCCGCCGATTCGAGCACAGCGGCGGCTGGATCCGCGCGCTCA
TGGAGGAGGCCGAGAACGAGCGCATGCACCTCATGACCTTCATGGAGGGCGTCT
TCTTCAACGCCTACTTCGTCGGCTACCTCATCTCCCCCAAGCTCAAGGACGTCGTC
ATCGCCGTGCGCGCCGACGAGGCGCATCACCGCGACGCCAACCACTACGCATCG
GACATCCATTACCAGGGAATGACGCTGAATCAGACGCCTGCGCCGCTCGGCTACC
ACTGA

AesAOX:
>*ne.AesAOX1d* CDS
ATGAGCTCTCGGATGGCCGGAGCCACGCTTCTGCGCCACCTGGGTCCCCGCCTC
TTCGCCGCCGCCGAGCCGGCCTCCGGGCTCGCCGCGAGCGCGAGGGGCATCAT
GCCCGCCGCCGCGAGGATCTTCCCCGCGCGGATGGCCAGCACCGAGGCCGCCG
GGCCGCATGCCAAACAAGAAAGTGACGCTGAAAAGCCCGAGAGCGCCGCGACGC
CGGAGCAGCAGAACAAGAAGCCCGTGGTGAGCTACTGGGGCATCGAGCCGCGGA
AGCTCGTCAAGGAGGACGGCACGGAGTGGCCATGGTTCTGCTTCAGGCCGTGGG
ACACGTACCGGCCGGACACGTCCATCGACGTGACCAAGCACCACGAGCCCAAGG
CCCTGGCGGACAAGGTGGCCTACTTCGTCGTCAGGTCGCTGCGCGTGCCCCGGG
ACCTCTTCTTCCAGCGGCGGCACGCGAGCCACGCGCTGCTGTTGGAGACGGTGG
CGGCCGTGCCCCCGATGGTGGGCGGCGTGTTGCTGCACCTGCGCTCGCTCCGCC
GCTTCGAGCACAGCGGCGGCTGGATCCGGGCGCTTATGGAGGAGGCCGAGAACG
AGCGCATGCACCTCATGACCTTCATGGAGGTGACGCAGCCGCGGTGGTGGGAGC
GCGCGCTCGTGCTCGCCGCGCAGGGCGTCTTCTTCAACGCCTACTTCGTCGGGTA
CCTCATCTCCCCCAAGTTCGCTCACCGCTTCGTCGGCTACCTCGAGGAGGAAGCC
GTGGAGTCTTATACTGAGTACCTCAAGGACCTAGAGGCCGGCTTGATCGAGAACA
CGCCCGCGCCGGCCATCGCCATCGACTACTGGCGCCTCCCCGCCGACGCCAGGC
TCAAGGACGTCGTCACCGCCGTGCGCGCCGACGAGGCGCATCACCGCGACGCCA
ACCACTATGCATCAGACATCCATTACCAGGGAATGACGCTGAATCAGACGCCTGC
GCCGCTCGGGTACCACTGA
