# Supplementary material for: Genome-wide identification and analysis of the ALTERNATIVE OXIDASE gene family in diploid and hexaploid wheat
Source: PLoS One. 2018 Aug 3;13(8):e0201439. doi: 10.1371/journal.pone.0201439 (PMC6075773; doi:10.1371/journal.pone.0201439)
Supplement: S5 Fig — (PDF) [file pone.0201439.s005.pdf]

S5 Fig. Alignment of coding sequence of high confidence hexaploid *TaAOX1d-2AL.2.sv1* wheat with the non-expressed coding sequences.

```
ne.TaAOX1d-2DL         ATGAGCTCTCGGATGGCCGGAGCCACGTTGCTGCGCCACCTGGGCCCCCACCTCTTCGCC  60
ne.TaAOX1d-2BL.2       ATGAGCTCTCGGATGGCCGGAGCCACGCTGCTGCGCCACCTGGGCCCCCGCCTCTTCGCC  60
ne.AesAOX1d            ATGAGCTCTCGGATGGCCGGAGCCACGCTTCTGCGCCACCTGGGTCCCCGCCTCTTCGCC  60
TaAOX1d-2AL.2.sv1      ATGAGCTCCCGGATGGCCGGAGCCACGCTTCTGCGCCACCTGGGCCCCCGCCTCTTCGCC  60
ne.TaAOX1d-2BL.1       ATGAGCTCTCGGATGGCCGGAGCCACGCTGCTGCGCCACCTGGGCCCCCGCCTCTTTGCC  60
                       ******** ****************** * ************** **** ****** ***

ne.TaAOX1d-2DL         GCCGCCGAGCCGGCGTCCGGGCTCGCCGCGAGCGCGAGGGGTATCCTGCCCGCCGCCGCG  120
ne.TaAOX1d-2BL.2       GCCGCCGAGCCGGCGTCCGGGCTCGCCGCGAGCGCGAGGGGCATCATGCCCGCCGCCGCG  120
ne.AesAOX1d            GCCGCCGAGCCGGCCTCCGGGCTCGCCGCGAGCGCGAGGGGCATCATGCCCGCCGCCGCG  120
TaAOX1d-2AL.2.sv1      GCCGCCGAGCCAGCCTCCGGGCTCGCCGCGAGCGCGAGGGGCATCATGCCCGCCGCCGCG  120
ne.TaAOX1d-2BL.1       GCCGCCGAGCCGGCCTCCGGGCTCGCCGCCAGCGCGAGGGGCATCATGCCCGCCGCCGCG  120
                       *********** ** ************** *********** *** **************

ne.TaAOX1d-2DL         AGGATCTTCCCCGCGCGGATGGCCAGCACCGCCG---CCGGCGCGCATGCCAAACAAGAA  177
ne.TaAOX1d-2BL.2       AGGATCTTCCCCGCGCGGATGGCCAGCACAGAGGCTGCCGGCCCGCGTGCCAAACAAGAA  180
ne.AesAOX1d            AGGATCTTCCCCGCGCGGATGGCCAGCACCGAGGCCGCCGGGCCGCATGCCAAACAAGAA  180
TaAOX1d-2AL.2.sv1      AGGATCTTCCCCGCGCGGATGGCCAGCACCGAGGCCGCCGCCCCGCATGCCAAACAAGAA  180
ne.TaAOX1d-2BL.1       AGGATCTTCCCCGCGCGGATGGCCAGCACCGAGGCCGCCGCCCCGCATGCCAAACAAGAA  180
                       ***************************** *  *   ***   *** *************

ne.TaAOX1d-2DL         GGTGACGCTGAAAAGCCCGAGAGCGCCACAGCGCCG---GAGCAGAACAAGAAGCCCGTG  234
ne.TaAOX1d-2BL.2       GAAGCCACTGAAAAGCCCCAGGGCGCAACAACGCCG---GAGCAGAACAAGAAGGCCGTG  237
ne.AesAOX1d            AGTGACGCTGAAAAGCCCGAGAGCGCCGCGACGCCGGAGCAGCAGAACAAGAAGCCCGTG  240
TaAOX1d-2AL.2.sv1      GATGATGCCGCGAGCCCCCAGGCGGCCGCGACTCCAGAGCAGCAGAACAAGAAGCCCGTG  240
ne.TaAOX1d-2BL.1       GATGATGCCGGAACACCCCAGGCGGCCGCGACTCCAGAGCAGCAGAGCAAGAAGGCCGTG  240
                          *   * *  *  *** **   **  *  * **     ****** ******* *****

ne.TaAOX1d-2DL         GCGAGCTACTGGGGCATCGAGCCGCGGAAGCTCGTCAAGGACGACGGCACGGAGTGGCCG  294
ne.TaAOX1d-2BL.2       GTGAGCTACTGGGGCATCGAGCCGCGGAAGCTCGTCAAGGACGACGGCACGGAGTGGCCG  297
ne.AesAOX1d            GTGAGCTACTGGGGCATCGAGCCGCGGAAGCTCGTCAAGGAGGACGGCACGGAGTGGCCA  300
TaAOX1d-2AL.2.sv1      GTGAGCTACTGGGGCATCGAGCCTCGGAAGCTCGTCAAGGATGACGGCACGGAGTGGCCA  300
ne.TaAOX1d-2BL.1       GTGAGCTACTGGGGCATCGAGCCGCGGAAGCTCGTCAAGGAGGACGGCACGGAGTGGCCG  300
                       * ********************* ***************** *****************

ne.TaAOX1d-2DL         TGGTTCTCCTTCAGGCCGTGGGACACGTACCGGCCGGACACGTCCATCGACGTGGCCAAG  354
ne.TaAOX1d-2BL.2       TGGTTCTCCTTCAGGCCGTGGGACACGTACCGGCCGGACACGTCCATCGACGTGGCCAAG  357
ne.AesAOX1d            TGGTTCTGCTTCAGGCCGTGGGACACGTACCGGCCGGACACGTCCATCGACGTGACCAAG  360
TaAOX1d-2AL.2.sv1      TGGTTCTGCTTCAGGCCGTGGGACACGTACCGGCCGGACACGTCCATCGAAGTGGCCAAG  360
ne.TaAOX1d-2BL.1       TGGTTCTGCTTCAGGCCGTGGGACACGTACCGGCCGGACACGTCCATCGACGTCACCAAG  360
                       ******* ****************************************** **  *****

ne.TaAOX1d-2DL         CACCACGAGCCCAGGGCGGTGGCGGACAAGGTGGCGTACCTCATCGTGCGGACGCTGCGC  414
ne.TaAOX1d-2BL.2       CACCACGAGCCCAGGGCGGTGGCGGACAAGGTGGCGTACCTCATCGTGCGGACGCTGCGC  417
ne.AesAOX1d            CACCACGAGCCCAAGGCCCTGGCGGACAAGGTGGCCTACTTCGTCGTCAGGTCGCTGCGC  420
TaAOX1d-2AL.2.sv1      CACCACGAGCCCAAGGCCCTGGCGGACAAGGTGGCCTACTTCGTGGTTCGGTCGCTGCGC  420
ne.TaAOX1d-2BL.1       CACCACGAGCCCAAGGCCCTGGCGGACAAGGTGGCCTACTTCGTGGTTCGGTCGCTGCGT  420
                       ************* ***  **************** *** ** * **  ** *******

ne.TaAOX1d-2DL         GCGGGCAGCGACCTCTTCTTCCAGCGCCGCCACGCTAGCCACGCGCTGCTGCTTGAGACG  474
ne.TaAOX1d-2BL.2       AAGGGAAGCGACCTCTTCTTCCAGCGCCGGCATGCGAGCCACGCCTTGCTGCTGGAGACG  477
ne.AesAOX1d            GTGCCCCGGGACCTCTTCTTCCAGCGGCGGCACGCGAGCCACGCGCTGCTGTTGGAGACG  480
TaAOX1d-2AL.2.sv1      GTGCCCCGGGACCTCTTCTTCCAGCGCCGGCACGCCAGCCATGCTCTGCTACTGGAAACG  480
ne.TaAOX1d-2BL.1       GTGCCGCGGGACCTCTTCTTCCAGCGCCGGCACGCCAGCCACGCGCTGCTGCTGGAGACT  480
                         *    * ***************** ** ** ** ***** **  ****  * ** **

ne.TaAOX1d-2DL         GTGGCGGCGGTGCCGCCCATGGTGGGCGGCGTGCTGCTGCACCTGCGCTCGCTCCGCCGA  534
ne.TaAOX1d-2BL.2       GTGGCCGCGGTGCCGCCCATGGTGGGCGGCGTGCTGCTGCACCTGCGCTCGCTCCGCCGC  537
ne.AesAOX1d            GTGGCGGCCGTGCCCCCGATGGTGGGCGGCGTGTTGCTGCACCTGCGCTCGCTCCGCCGC  540
TaAOX1d-2AL.2.sv1      GTGGCGGCGGTGCCTCCCATGGTGGGCGGCGTGCTGCTGCACCTGCGCTCGCTCCGCCGC  540
ne.TaAOX1d-2BL.1       GTGGCGGCGGTGCCGCCCATGGTGGGCGGCGTGCTGCTCCACCTGCGCTCGCTCCGCCGA  540
                       ***** ** ***** ** *************** **** ********************
```

```
ne.TaAOX1d-2DL          TTCGAGCACAGCGGCGGCTGGATCCGCGCGCTCATGGAGGAGGCCGAGAACGAGCGCATG  594
ne.TaAOX1d-2BL.2        TTCGAGCACAGCGGCGGCTGGATCCGCGCGCTCATGGAGGAGGCCGAGAACGAGCGCATG  597
ne.AesAOX1d             TTCGAGCACAGCGGCGGCTGGATCCGGGCGCTTATGGAGGAGGCCGAGAACGAGCGCATG  600
TaAOX1d-2AL.2.sv1       TTCGAGCACAGCGGCGGCTGGATCCGGGCGCTCATGGAGGAGGCCGAGAACGAGCGCATG  600
ne.TaAOX1d-2BL.1        TTCGAGCACAGCGGCGGCTGGATCCGGGCGCTCATGGAGGAGGCCGAGAACGAGCGCATG  600
                        ************************** ***** ***************************

ne.TaAOX1d-2DL          CACCTCATGACCTTCATGGAGGTGACGCAGCCCCTGTGGTGGGAGCGCGCGCTCGTGCTC  654
ne.TaAOX1d-2BL.2        CACCTCATGACCTTCATGGAGGTGACGCAGCCGCTGTGGTGGGAGCGCGCGCTCGTGCTC  657
ne.AesAOX1d             CACCTCATGACCTTCATGGAGGTGACGCAGCCGCGGTGGTGGGAGCGCGCGCTCGTGCTC  660
TaAOX1d-2AL.2.sv1       CACCTCATGACCTTCATGGAGGTGACGCAGCCGCGGTGGTGGGAGCGCGCGCTCGTGCTC  660
ne.TaAOX1d-2BL.1        CACCTCATGACCTTCATGGAGGTGACGCAGCCGCGCTGGTGGGAGCGCGCGCTCGTGCTC  660
                        ******************************** *  ************************

ne.TaAOX1d-2DL          GCCACGCAGGGCGTCTTCTTCAACGCCTACTTCGTCGGCTACCTCATCTCCCCCAAGTTC  714
ne.TaAOX1d-2BL.2        GCCACTCAGGGCGTCTTCTTCAACGCCTACTTCGTCGGCTACCTCATCTCCCCAAAGTTC  717
ne.AesAOX1d             GCCGCGCAGGGCGTCTTCTTCAACGCCTACTTCGTCGGGTACCTCATCTCCCCCAAGTTC  720
TaAOX1d-2AL.2.sv1       GCCGCGCAGGGCGTCTTCTTCAACGCCTACTTCGTCGGGTACCTCATTTCCCCCAAGTTC  720
ne.TaAOX1d-2BL.1        GCCGCGCAGGGCGTCTTCTTCAACGCCTACTTCGTCGGCTACCTCATCTCCCCCAAGTTC  720
                        *** * ******************************** ******** ***** ******

ne.TaAOX1d-2DL          GCGCACCGCTTCGTCGGCTACCTCGAGGAGGAGGCCGTCCACTCCTACACCGAGTACCTC  774
ne.TaAOX1d-2BL.2        GCGCACCGCTTCGTCGGCTACCTCGAGGAGGAGGCCGTCCACTCCTACACCGAGTACCTC  777
ne.AesAOX1d             GCTCACCGCTTCGTCGGCTACCTCGAGGAGGAAGCCGTGGAGTCTTATACTGAGTACCTC  780
TaAOX1d-2AL.2.sv1       GCGCACCGCTTCGTCGGGTACCTCGAGGAGGAGGCCGTGGAGTCTTATACTGAGTATCTC  780
ne.TaAOX1d-2BL.1        GCGCACCGCTTCGTCGGCTACCTCGAGGAGGAGGCCGTGGAGTCTTACACTGAGTACCTC  780
                        ** ************** ************** *****  * ** ** ** ***** ***

ne.TaAOX1d-2DL          AAGGACCTTGAGGCCGGCTTGATCGAGAACACGCCCGCGCCGGCCATTGCCATCGACTAC  834
ne.TaAOX1d-2BL.2        AAGGACCTCGAGGCCGGCTTGATCGAGAACACGCCCGCGCCGGCCATCGCCATCGACTAC  837
ne.AesAOX1d             AAGGACCTAGAGGCCGGCTTGATCGAGAACACGCCCGCGCCGGCCATCGCCATCGACTAC  840
TaAOX1d-2AL.2.sv1       AAGGACCTTGAGGCCGGATTGATCGAGAACACGCCCGCGCCGGCCATCGCCATCGACTAC  840
ne.TaAOX1d-2BL.1        AAGGACCTCGAAGCCGGCTTGATCGAGAACACGCCCGCGCCGGCCATCGCCATCGACTAC  840
                        ******** ** ***** ***************************** ************

ne.TaAOX1d-2DL          TGGCGCCTCCCCGCCGACGCCAGGCTCAAGGACGTCGTCATCGCCGTGCGCGCCGACGAG  894
ne.TaAOX1d-2BL.2        TGGCGCCTCCCCGCCGACGCCAGGCTCAAAGACGTCGTCATCGCCGTGCGCGCCGACGAG  897
ne.AesAOX1d             TGGCGCCTCCCCGCCGACGCCAGGCTCAAGGACGTCGTCACCGCCGTGCGCGCCGACGAG  900
TaAOX1d-2AL.2.sv1       TGGCGCCTCCCCGCCGACGCCAGGCTCAAGGACGTCGTCACCGCCGTGCGCGCCGACGAG  900
ne.TaAOX1d-2BL.1        TGGCGCCTCCCCGCCGACGCCAGGCTCAAAGACGTCGTCACCGCCGTGCGCGCCGACGAG  900
                        ***************************** ********** *******************

ne.TaAOX1d-2DL          GCGCATCACCGCGACGCCAACCACTACGCATCGGACATCCATTACCAGGGAATGACGCTG  954
ne.TaAOX1d-2BL.2        GCGCATCACCGCGACGCCAACCACTACGCATCGGACATCCATTACCAGGGAATGACGCTG  957
ne.AesAOX1d             GCGCATCACCGCGACGCCAACCACTATGCATCAGACATCCATTACCAGGGAATGACGCTG  960
TaAOX1d-2AL.2.sv1       GCGCATCACCGCGACGCCAACCACTACGCATCGGACGTCCATTACCAGGGAATGACGCTG  960
ne.TaAOX1d-2BL.1        GCGCATCACCGCGACGCCAACCACTACGCATCGGACATCCATTACCAGGGAATGACGCTG  960
                        ************************** ***** *** ***********************

ne.TaAOX1d-2DL          AATCAGACGCCTGCGCCGCTCGGCTACCACTGA    987
ne.TaAOX1d-2BL.2        AATCAGACGCCTGCGCCGCTCGGGTACCACTGA    990
ne.AesAOX1d             AATCAGACGCCTGCGCCGCTCGGGTACCACTGA    993
TaAOX1d-2AL.2.sv1       AATCAATCGCCTGCGCCGCTCGGGTACCACTGA    993
ne.TaAOX1d-2BL.1        AATCAGACGCCTGCGCCGCTCGGGTACCACTGA    993
                        *****  **************** *********
```
