# Supplementary material for: Genome-wide identification and analysis of the ALTERNATIVE OXIDASE gene family in diploid and hexaploid wheat
Source: PLoS One. 2018 Aug 3;13(8):e0201439. doi: 10.1371/journal.pone.0201439 (PMC6075773; doi:10.1371/journal.pone.0201439)
Supplement: S6 Fig — (PDF) [file pone.0201439.s006.pdf]

S6 Fig. Alignment of high confidence protein sequence of hexaploid TaAOX1d-2AL.2.sv1 protein sequence with the non-expressed protein sequences.

```
TaAOX1d-2AL.2.sv1      MSSRMAGATLLRHLGPRLFAAAEPASGLAASARGIMPAAARIFPARMASTEAAAPHAKQE  60
ne.TaAOX1d-2BL.1       MSSRMAGATLLRHLGPRLFAAAEPASGLAASARGIMPAAARIFPARMASTEAAAPHAKQE  60
ne.AesAOX1d            MSSRMAGATLLRHLGPRLFAAAEPASGLAASARGIMPAAARIFPARMASTEAAGPHAKQE  60
ne.TaAOX1d-2DL         MSSRMAGATLLRHLGPHLFAAAEPASGLAASARGILPAAARIFPARMASTA-AGAHAKQE  59
ne.TaAOX1d-2BL.2       MSSRMAGATLLRHLGPRLFAAAEPASGLAASARGIMPAAARIFPARMASTEAAGPRAKQE  60
                       ****************:******************:**************  *. :****

TaAOX1d-2AL.2.sv1      DDAASPQAAATPEQQNKKPVVSYWGIEPRKLVKDDGTEWPWFCFRPWDTYRPDTSIEVAK  120
ne.TaAOX1d-2BL.1       DDAGTPQAAATPEQQSKKAVVSYWGIEPRKLVKEDGTEWPWFCFRPWDTYRPDTSIDVTK  120
ne.AesAOX1d            SDAEKPESAATPEQQNKKPVVSYWGIEPRKLVKEDGTEWPWFCFRPWDTYRPDTSIDVTK  120
ne.TaAOX1d-2DL         GDAEKPESATAPE-QNKKPVASYWGIEPRKLVKDDGTEWPWFSFRPWDTYRPDTSIDVAK  118
ne.TaAOX1d-2BL.2       EATEKPQGATTPE-QNKKAVVSYWGIEPRKLVKDDGTEWPWFSFRPWDTYRPDTSIDVAK  119
                         : .*:.*::** *.** *.************:********.*************:*:*

TaAOX1d-2AL.2.sv1      HHEPKALADKVAYFVVRSLRVPRDLFFQRRHASHALLLETVAAVPPMVGGVLLHLRSLRR  180
ne.TaAOX1d-2BL.1       HHEPKALADKVAYFVVRSLRVPRDLFFQRRHASHALLLETVAAVPPMVGGVLLHLRSLRR  180
ne.AesAOX1d            HHEPKALADKVAYFVVRSLRVPRDLFFQRRHASHALLLETVAAVPPMVGGVLLHLRSLRR  180
ne.TaAOX1d-2DL         HHEPRAVADKVAYLIVRTLRAGSDLFFQRRHASHALLLETVAAVPPMVGGVLLHLRSLRR  178
ne.TaAOX1d-2BL.2       HHEPRAVADKVAYLIVRTLRKGSDLFFQRRHASHALLLETVAAVPPMVGGVLLHLRSLRR  179
                       ****:*:******::**:**   *************************************

TaAOX1d-2AL.2.sv1      FEHSGGWIRALMEEAENERMHLMTFMEVTQPRWWERALVLAAQGVFFNAYFVGYLISPKF  240
ne.TaAOX1d-2BL.1       FEHSGGWIRALMEEAENERMHLMTFMEVTQPRWWERALVLAAQGVFFNAYFVGYLISPKF  240
ne.AesAOX1d            FEHSGGWIRALMEEAENERMHLMTFMEVTQPRWWERALVLAAQGVFFNAYFVGYLISPKF  240
ne.TaAOX1d-2DL         FEHSGGWIRALMEEAENERMHLMTFMEVTQPLWWERALVLATQGVFFNAYFVGYLISPKF  238
ne.TaAOX1d-2BL.2       FEHSGGWIRALMEEAENERMHLMTFMEVTQPLWWERALVLATQGVFFNAYFVGYLISPKF  239
                       ******************************* *********:******************

TaAOX1d-2AL.2.sv1      AHRFVGYLEEEAVESYTEYLKDLEAGLIENTPAPAIAIDYWRLPADARLKDVVTAVRADE  300
ne.TaAOX1d-2BL.1       AHRFVGYLEEEAVESYTEYLKDLEAGLIENTPAPAIAIDYWRLPADARLKDVVTAVRADE  300
ne.AesAOX1d            AHRFVGYLEEEAVESYTEYLKDLEAGLIENTPAPAIAIDYWRLPADARLKDVVTAVRADE  300
ne.TaAOX1d-2DL         AHRFVGYLEEEAVHSYTEYLKDLEAGLIENTPAPAIAIDYWRLPADARLKDVVIAVRADE  298
ne.TaAOX1d-2BL.2       AHRFVGYLEEEAVHSYTEYLKDLEAGLIENTPAPAIAIDYWRLPADARLKDVVIAVRADE  299
                       *************.*************************************** ******

TaAOX1d-2AL.2.sv1      AHHRDANHYASDVHYQGMTLNQSPAPLGYH      330
ne.TaAOX1d-2BL.1       AHHRDANHYASDIHYQGMTLNQTPAPLGYH      330
ne.AesAOX1d            AHHRDANHYASDIHYQGMTLNQTPAPLGYH      330
ne.TaAOX1d-2DL         AHHRDANHYASDIHYQGMTLNQTPAPLGYH      328
ne.TaAOX1d-2BL.2       AHHRDANHYASDIHYQGMTLNQTPAPLGYH      329
                       ************:*********:*******
```
