# Supplementary material for: Genome-wide identification and analysis of the ALTERNATIVE OXIDASE gene family in diploid and hexaploid wheat
Source: PLoS One. 2018 Aug 3;13(8):e0201439. doi: 10.1371/journal.pone.0201439 (PMC6075773; doi:10.1371/journal.pone.0201439)
Supplement: S7 Fig — (PDF) [file pone.0201439.s007.pdf]

S7 Fig. Alignment and Phylogeny of Waox1a and Waox1c proteins with closest hexaploid wheat relatives used in this study.

```
Waox1a:

TaAOX1a-2AL.sv1  MSSRMAGSVLLRRAGAGAGRLFATTASPAARTALGGGEGAWVRMMSTSAASQVKDEAAKG        60
Waox1a           MSSRMAGSVLLRRAGAGAGRLFATTASPAARTALGGGGGAWVRMMSTSAASQVKDEAAKG        60
                 ************************************* **********************

TaAOX1a-2AL.sv1  VKAEAAKGDGEKKEVAISSYWGIEQSKKLVREDGTEWKWSCFRPWETYTADTSIDLTKHH        120
Waox1a           VKAEAAKGDGEKKEVAISSYWGIDQSKKLVREDGTEWKWSCFRPWETYTADTSIDLTKHH        120
                 ***********************:************************************

TaAOX1a-2AL.sv1  VPNTMLDKIAYYTVKSLRFPTDIFFQRRYGCRAMMLETVAAVPGMVGGMLLHLRSLRRFE        180
Waox1a           VPNTMLDKIAYYTVKSLRFPTDIFFQRRYGCRAMMLETVAAVPGMVGGMLLHLRSLRRFE        180
                 ************************************************************

TaAOX1a-2AL.sv1  QSGGWIRALLEEAENERMHLMTFMEVAQPRWYERALVIAVQGVFFNAYFFGYLISPKFAH        240
Waox1a           QSGGWIRALLEEAENERMHLMTFMEVAQPRWYERALVIAVQGVFFNAYFFGYLISPKFAH        240
                 ************************************************************

TaAOX1a-2AL.sv1  RVVGYLEEEAVHSYTEFLKDLDDGKIDNVPAPAIAIDYWRLPANATLKDVVTVVRADEAH        300
Waox1a           RVVGYLEEEAVHSYTEFLKDLDDGKIDNVPAPAIAIDYWRLPANATLKDVVTVVRADEAH        300
                 ************************************************************

TaAOX1a-2AL.sv1  HRDVNHFASDVYYQGMQLKATPAPIGYH      328
Waox1a           HRDVNHFASDVYYQGMQLKATPAPIGYH      328
                 ****************************


Waox1c:

TaAOX1c-6AL      MPSWRALARRQRHVIPSPSQSLARPQVLEPATTSFASRAAAHQAGSSSSAMSSRVAGSVL        60
Waox1c           --------------------------------------------------MSSRVAGSVL        10
                                                                   **********

TaAOX1c-6AL      LRHLGPRVFGPTTPAAQRPLLAGGEGGAVAVAMWARPLSTSAAEAAREEATASKDNVAST        120
Waox1c           LRHLGPRVFGPTTPAAQRPLLAGGEGGAVAVAMWARPLSTSAAEAAREEATASKDNVAST        70
                 ************************************************************

TaAOX1c-6AL      AAATAEAMQAAKADAVQAAKEGKSPAASSYWGIVPAKLVNKDGAEWKWSCFRPWEAYTSD        180
Waox1c           AAATAEAMQAAKAGAVQAAKEGKSPAASSYWGIVPAKLVNKDGAEWKWSCFRPWEAYTSD        130
                 *************.**********************************************

TaAOX1c-6AL      TTIDLSKHHKPKVLLDKIAYWTVKSLRVPTDIFFQRRYGCRAMMLETVAAVPGMVGGMLL        240
Waox1c           TTIDLSKHHKPKVLLDKIAYWTVKSLRVPTDIFFQRRYGCRAMMLETVAAVPGMVDGMLL        190
                 ********************************************************.***

TaAOX1c-6AL      HLRSLRRFEQSGGWIRALLEEAENERMHLMTFMEVANPKWYERALVLAVQGVFFNAYFLG        300
Waox1c           HLRSLRRFEQSGGWIRALLEEAENERMHLMTFMEVANPKWYERALVLAVQGVFFNAYFLG        250
                 ************************************************************

TaAOX1c-6AL      YIVSPKFAHRVVGYLEEEAIHSYTEFLRDLEAGRIENVPAPRIAIDYWRLPADARLKDVV        360
Waox1c           YIVSPKFAHRVVGYLEEEAIHSYTEFLRDLEAGRIENVPAPRIAIDYWRLPADARLKDVV        310
                 ************************************************************

TaAOX1c-6AL      TVVRADEAHHRDVNHFAADIHFQGLELNKTPAPLGYH      397
Waox1c           TVVRADEAHHRDVNHFAADIHFQGLELNKTPAPLGYH      347
                 *************************************
```

This excerpt is from the full phylogenetic tree*, which was created using the same methodology from the manuscript.

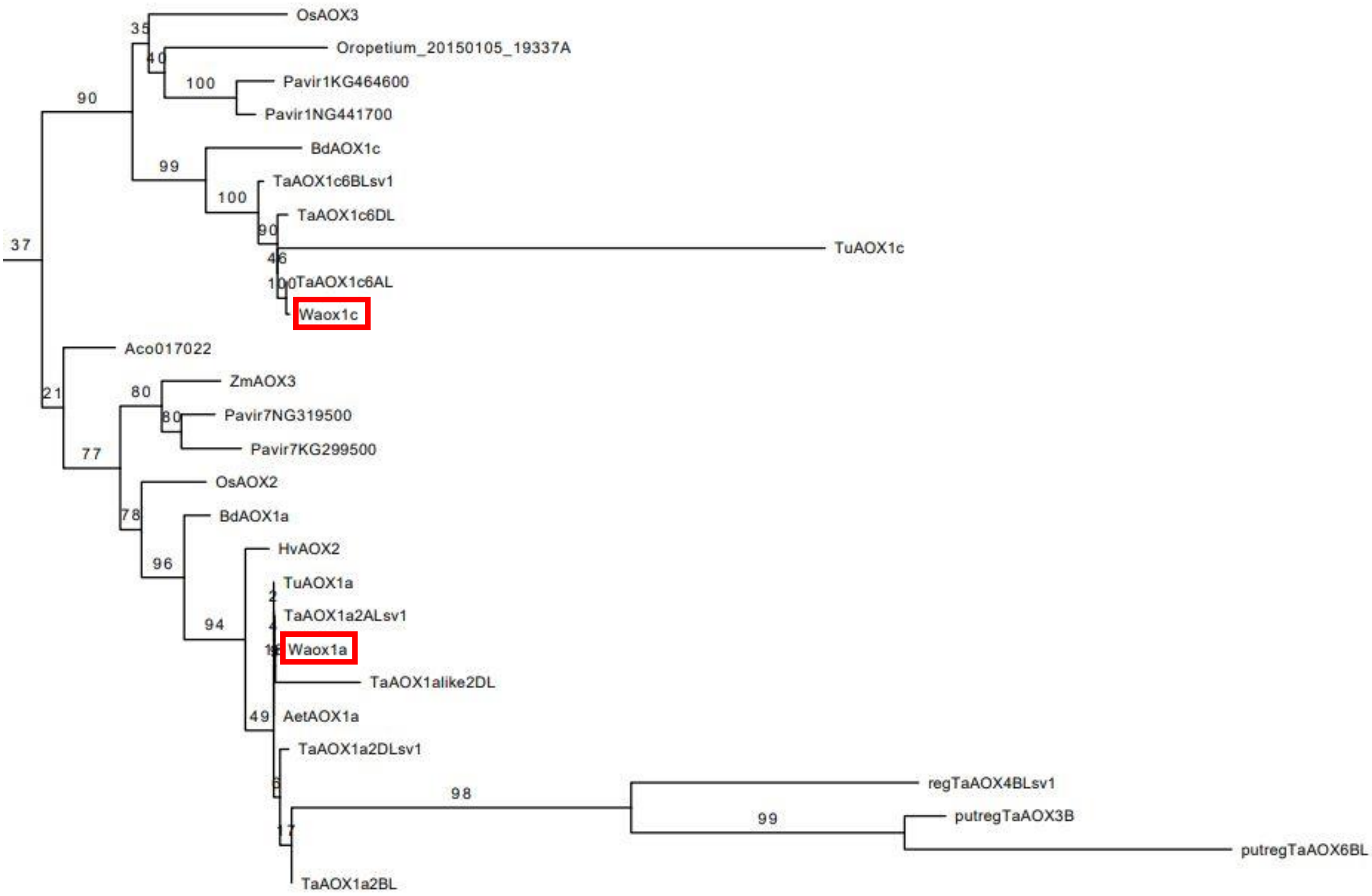

*Full Tree:

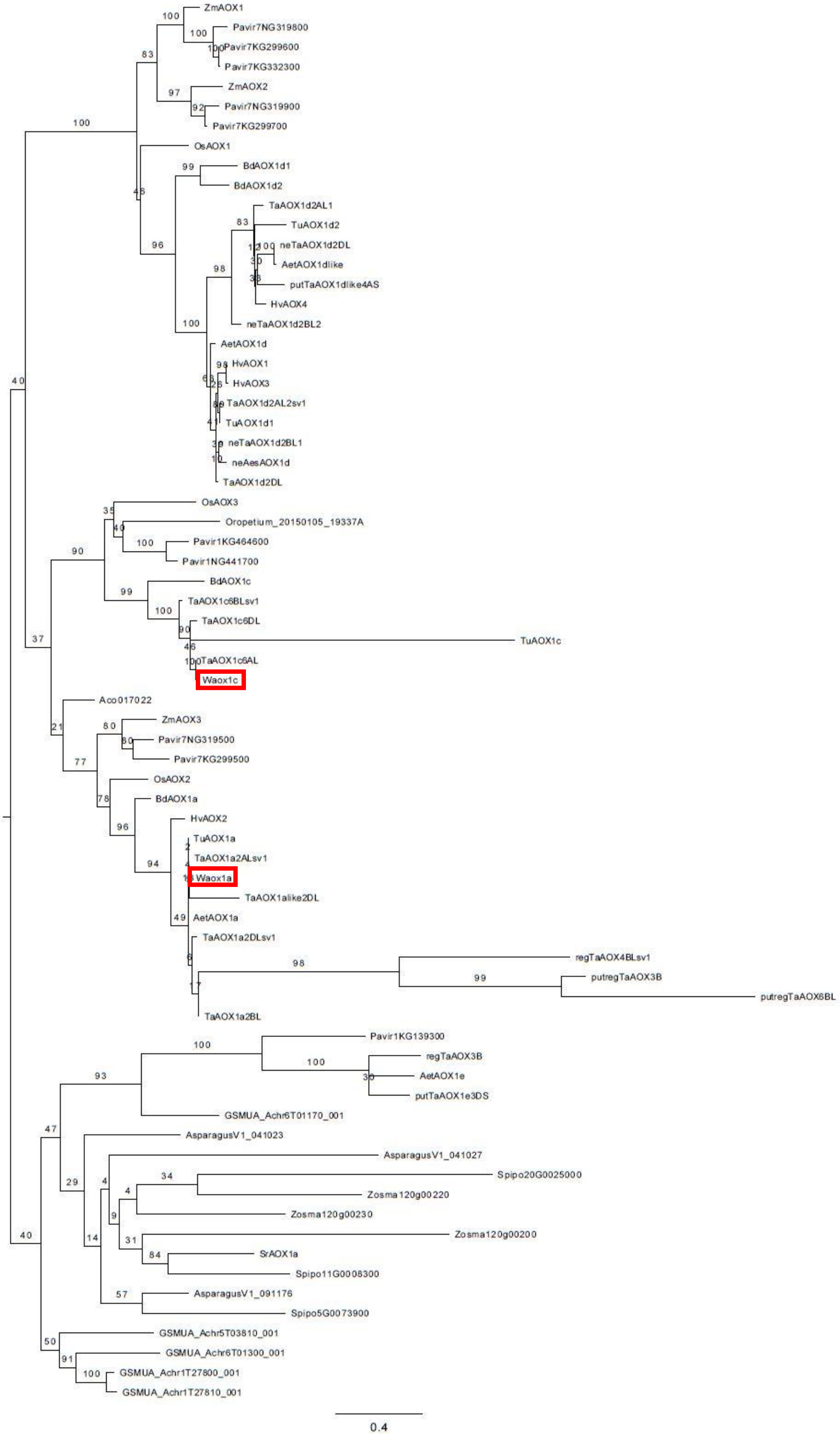
ZmAOX1
Pavir7NG319800
Pavir7KG299600
Pavir7KG332300
ZmAOX2
Pavir7NG319900
Pavir7KG299700
OsAOX1
BdAOX1d1
BdAOX1d2
TaAOX1d2AL1
TuAOX1d2
neTaAOX1d2DL
AetAOX1dlike
putTaAOX1dlike4AS
HvAOX4
neTaAOX1d2BL2
AetAOX1d
HvAOX1
HvAOX3
TaAOX1d2AL2sv1
TuAOX1d1
neTaAOX1d2BL1
neAesAOX1d
TaAOX1d2DL
OsAOX3
Oropetium_20150105_19337A
Pavir1KG464600
Pavir1NG441700
BdAOX1c
TaAOX1c6BLsv1
TaAOX1c6DL
TuAOX1c
TaAOX1c6AL
Waox1c
Aco017022
ZmAOX3
Pavir7NG319500
Pavir7KG299500
OsAOX2
BdAOX1a
HvAOX2
TuAOX1a
TaAOX1a2ALsv1
Waox1a
TaAOX1alike2DL
AetAOX1a
TaAOX1a2DLsv1
regTaAOX4BLsv1
putregTaAOX3B
putregTaAOX6BL
TaAOX1a2BL
Pavir1KG139300
regTaAOX3B
AetAOX1e
putTaAOX1e3DS
GSMUA_Achr6T01170_001
AsparagusV1_041023
AsparagusV1_041027
Spipo20G0025000
Zosma120g00220
Zosma120g00230
Zosma120g00200
SrAOX1a
Spipo11G0008300
AsparagusV1_091176
Spipo5G0073900
GSMUA_Achr5T03810_001
GSMUA_Achr6T01300_001
GSMUA_Achr1T27800_001
GSMUA_Achr1T27810_001
0.4
