# Supplementary material for: Genome-wide identification and analysis of the ALTERNATIVE OXIDASE gene family in diploid and hexaploid wheat
Source: PLoS One. 2018 Aug 3;13(8):e0201439. doi: 10.1371/journal.pone.0201439 (PMC6075773; doi:10.1371/journal.pone.0201439)
Supplement: S9 Fig — The “reg” proteins were not analyzed due to absence of the amino acids within this region. (PDF) [file pone.0201439.s009.pdf]

S9 Fig. Comparison of TbAOX internal mitochondrial targeting peptide sequence with TaAOX residues in similar regions. The “reg” proteins were not analyzed due to absence of the amino acids within this region.

```
>TbAOX
VISRCLFLETVAGVPGMVGGMLRHLSSLRYMT
>TaAOX1a-2AL.sv1
CRAMMLETVAAVPGMVGGMLLHLRSLRRFE
>TaAOX1a-2BL
CRAMMLETVAAVPGMVGGMLLHLRSLRRFE
>TaAOX1a-2DL.sv1
CRAMMLETVAAVPGMVGGMLLHLRSLRRFE
>TaAOX1a-like-2DL
MVGGVLLHLRSLRRFE
>TaAOX1c-6AL
CRAMMLETVAAVPGMVGGMLLHLRSLRRFE
>TaAOX1c-6BL.sv1
CRAMMLETVAAVPGMVGGMLLHLRSLRRFE
>TaAOX1c-6DL
CRAMMLETVAAVPGMVGGMLLHLRSLRRFE
>put.TaAOX1e-3DS
CRAMMLETVAAVPGMVARAVLHLRSLRRFE
>TaAOX1d-2AL.1
SHALLLEMVAAVPPMVGGVLLHLRSLRRFE
>TaAOX1d-2AL.2.sv1
SHALLLETVAAVPPMVGGVLLHLRSLRRFE
>TaAOX1d-2DL
SHALLLETVAAVPPMVGGVLLHLRSLRRFE
>put.TaAOX1d-like-4AS
SHMLLLETVAAVPPMVGGVLLHLRSLRRFE
```

```
TbAOX                     VISRCLFLETVAGVPGMVGGMLRHLSSLRYMT  32
TaAOX1a-2AL.sv1           --CRAMMLETVAAVPGMVGGMLLHLRSLRRFE  30
TaAOX1a-2BL               --CRAMMLETVAAVPGMVGGMLLHLRSLRRFE  30
TaAOX1a-2DL.sv1           --CRAMMLETVAAVPGMVGGMLLHLRSLRRFE  30
TaAOX1a-like-2DL          ----------------MVGGVLLHLRSLRRFE  16
TaAOX1c-6AL               --CRAMMLETVAAVPGMVGGMLLHLRSLRRFE  30
TaAOX1c-6BL.sv1           --CRAMMLETVAAVPGMVGGMLLHLRSLRRFE  30
TaAOX1c-6DL               --CRAMMLETVAAVPGMVGGMLLHLRSLRRFE  30
put.TaAOX1e-3DS           --CRAMMLETVAAVPGMVARAVLHLRSLRRFE  30
TaAOX1d-2AL.1             --SHALLLEMVAAVPPMVGGVLLHLRSLRRFE  30
TaAOX1d-2AL.2.sv1         --SHALLLETVAAVPPMVGGVLLHLRSLRRFE  30
TaAOX1d-2DL               --SHALLLETVAAVPPMVGGVLLHLRSLRRFE  30
put.TaAOX1d-like-4AS      --SHMLLLETVAAVPPMVGGVLLHLRSLRRFE  30
                                          **.  : ** *** :
```
