# Supplementary material for: Genome-wide identification and analysis of the ALTERNATIVE OXIDASE gene family in diploid and hexaploid wheat
Source: PLoS One. 2018 Aug 3;13(8):e0201439. doi: 10.1371/journal.pone.0201439 (PMC6075773; doi:10.1371/journal.pone.0201439)
Supplement: S2 Table — The highlighted residues in the motif are critical in the active site diiron center. X indicates presence of motifs. *Indicates diploid wheat isoforms. (PDF) [file pone.0201439.s011.pdf]

S2 Table. Conserved AOX motifs in the wheat protein isoforms. The highlighted residues in the motif are critical in the active site diiron center. X indicates presence of motifs. *Indicates diploid wheat isoforms.

Sequence	LETVAA	ERMHLMT	LEEEA	RADEAHH
TaAOX1a-2AL.sv1	X	X	X	X
TaAOX1a-2AL.sv2	X	X	X	X
TaAOX1a-2BL	X	X	X	X
TaAOX1a-2DL.sv1	X	X	X	X
TaAOX1a-2DL.sv2	X	X	X	X
TaAOX1a-like-2DL		X	X	X
regTaAOX-4BL.sv1				
regTaAOX-4BL.sv2				
regTaAOX-4BL.sv3				
regTaAOX-4BL.sv4				
put.regTaAOX-3B				
put.regTaAOX-6BL				
TaAOX1c-6AL	X	X	X	X
TaAOX1c-6BL.sv1	X	X	X	X
TaAOX1c-6BL.sv2	X	X	X	X
TaAOX1c-6BL.sv3	X	X	X	X
TaAOX1c-6DL	X	X	X	X
regTaAOX-3B			X	
put.TaAOX1e-3DS	X	X	X	X
TaAOX1d-2AL.2.sv1	X	X	X	X
TaAOX1d-2AL.2.sv2	X	X	X	X
TaAOX1d-2AL.1	X	X	X	X
TaAOX1d-2DL	X	X	X	X
put.TaAOX1d-like-4AS	X	X	X	
TuAOX1c*	X	X	X	X
TuAOX1a*	X	X	X	X
TuAOX1d.1*	X	X	X	X
TuAOX1d.2*	X	X	X	X
AetAOX1d*	X	X	X	X
AetAOX1d-like*	X	X		X
AetAOX1e*	X	X	X	X
AetAOX1a*	X	X	X	X
