# Supplementary material for: Genome-wide identification and analysis of the ALTERNATIVE OXIDASE gene family in diploid and hexaploid wheat
Source: PLoS One. 2018 Aug 3;13(8):e0201439. doi: 10.1371/journal.pone.0201439 (PMC6075773; doi:10.1371/journal.pone.0201439)
Supplement: S3 Table — *Denotes negative regulators of AOX expression. (PDF) [file pone.0201439.s012.pdf]

S3 Table. Regulators and motifs in *AOX* expression. *Denotes negative regulators of *AOX* expression.

Gene Name	Locus	Function	Reference
ANAC013	At1g32870	Activator of *AOX1a*.	De Clercq et al. 2013; Van Aken et al. 2016
ANAC017	At1g34190	Main Positive regulator, epistatic over *ANAC013* and *ANAC053.*	Ng et al. 2013b;Van Aken et al. 2016
AtWRKY63	At1g66600	Activator of *AOX1a.*	Van Aken et al. 2013
ANAC053	At3g10500	Positive regulator.	De Clercq et al. 2013;Van Aken et al. 2016
ANAC078	At5g04410	Positive regulator.	De Clercq et al. 2013;Van Aken et al. 2016
RETARDED ROOT GROWTH-LIKE	At5g13610	Activator of *AOX1a* under ABA induction. Located in mitochondria.	Yao et al. 2015
CDKE1/RAO1	At5g63610	Activator of *AOX1a*.	Ng et al. 2013a
*RCD1**	At1g32230	Suppressor of *AOX1a*.	Brosche et al. 2014
*AtWRKY40**	At1g80840	Negative regulator.	Van Aken et al. 2016
*ABI4**	At2g40220	Negative regulator of *AOX1a*.	Giraud et al. 2009
*MYB29**	At5g07690	Negative regulator of *AOX1a*.	Zhang et al. 2017
*DGS1**	At5g12290	*AOX* suppressor (post transcriptional).	Moellering and Benning 2010
AAACCASSSGC	Consensus sequence	Hypoxia responsive promoter element.	Gasch et al. 2016
CTTGNNNNNCAMG / YTTGNNNNNVAMV	Consensus sequence	Mitochondrial Dysfunctional Motif (MDM). Needed for transcriptional activation of mitochondrial retrograde regulation.	De Clercq et al. 2013
