# Supplementary material for: Genome-wide identification and analysis of the ALTERNATIVE OXIDASE gene family in diploid and hexaploid wheat
Source: PLoS One. 2018 Aug 3;13(8):e0201439. doi: 10.1371/journal.pone.0201439 (PMC6075773; doi:10.1371/journal.pone.0201439)
Supplement: S8 Table — The model used was c3vvaD. *Models to c3rylB. (PDF) [file pone.0201439.s017.pdf]

S8 Table. Summary of TaAOX 3-D structures obtained with Phyre2. The model used was c3vvaD. *Models to c3rylB.

Protein Name	Confidence	Alignment Coverage %	3D Image	Membrane Topology
TaAOX1a-2AL.sv1	100	75	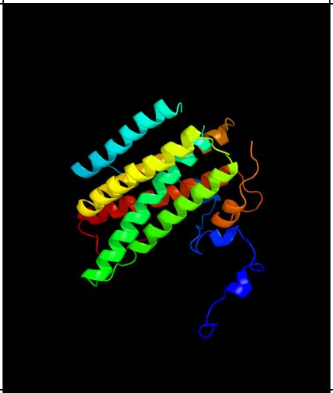	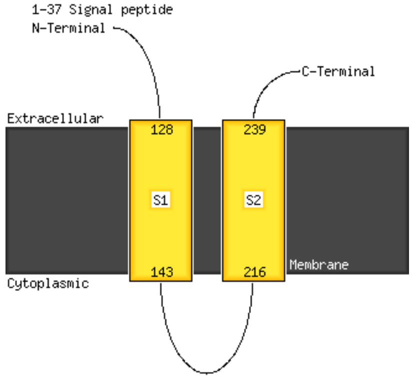
TaAOX1a-2AL.sv2	100	76	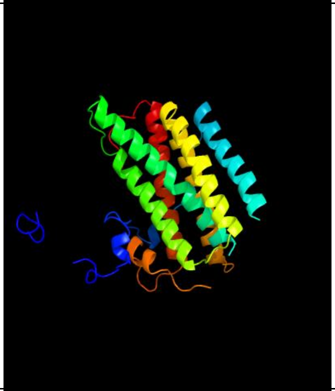	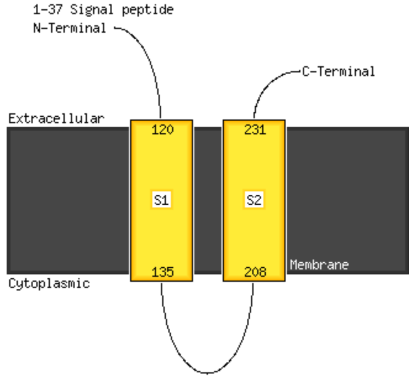
TaAOX1a-2BL	100	51	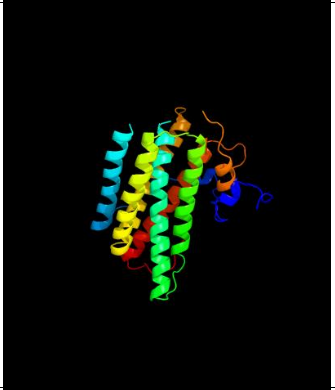	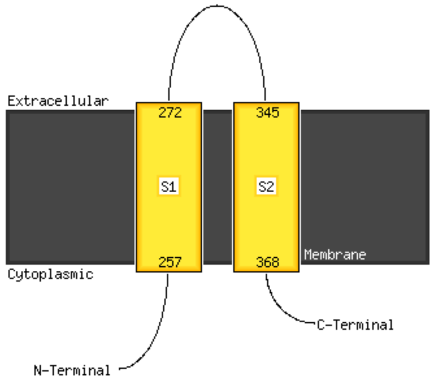
TaAOX1a-2DL.sv1	100	71	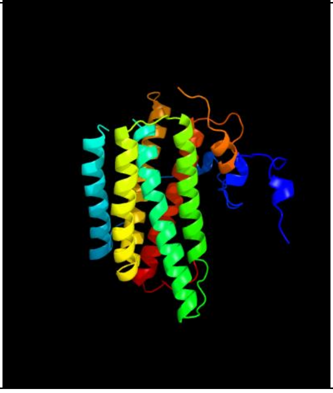	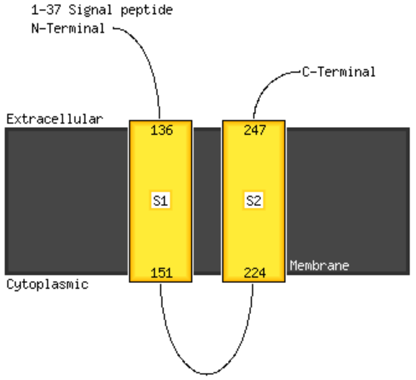

TaAOX1a-2DL.sv2	100	85	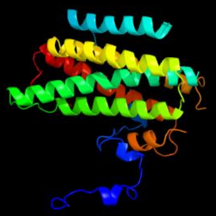	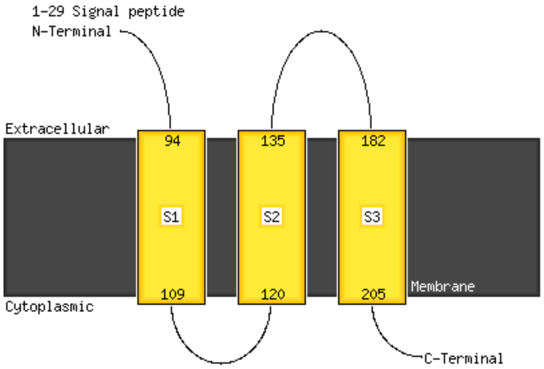
TaAOX1a-like-2DL	100	96	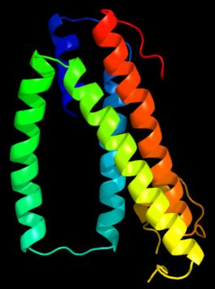	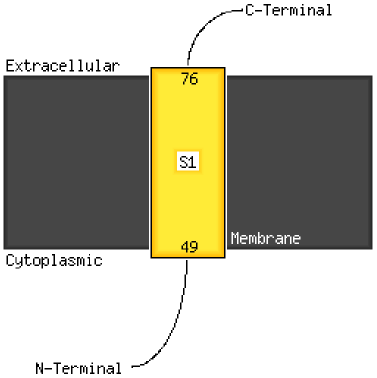
regTaAOX-4BL.sv1	97	25	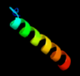	N/A
regTaAOX-4BL.sv2	97	32	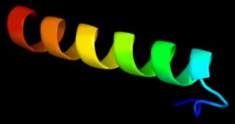	N/A
regTaAOX-4BL.sv3	97	30	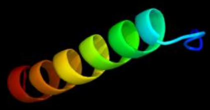	N/A

regTaAOX-4BL.sv4	97	25	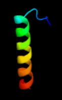	N/A
put.regTaAOX-3B	97.1	28	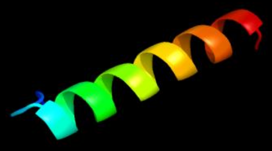	N/A
put.regTaAOX-6BL*	43.7	12	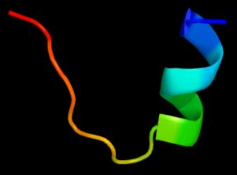	N/A
TaAOX1c-6AL	100	57	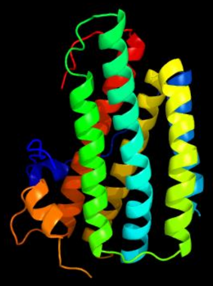	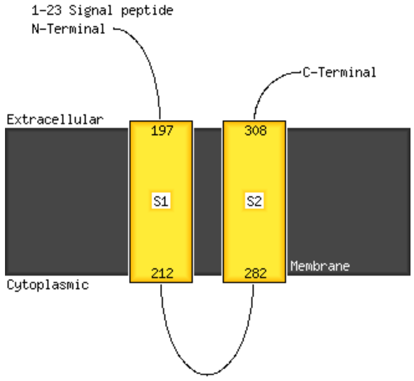
TaAOX1c-6BL.sv1	100	54	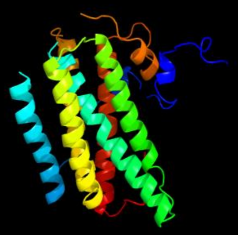	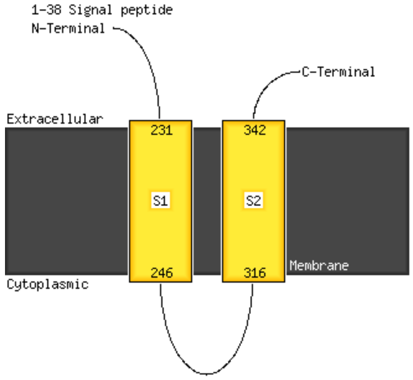

TaAOX1c-6BL.sv2	100	60	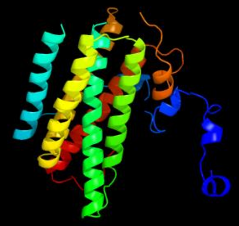	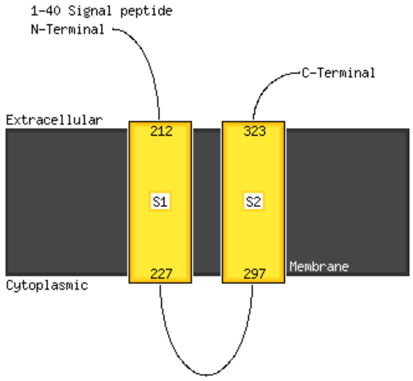
TaAOX1c-6BL.sv3	100	54	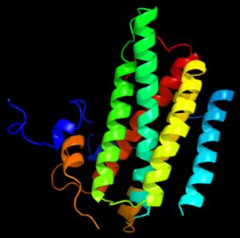	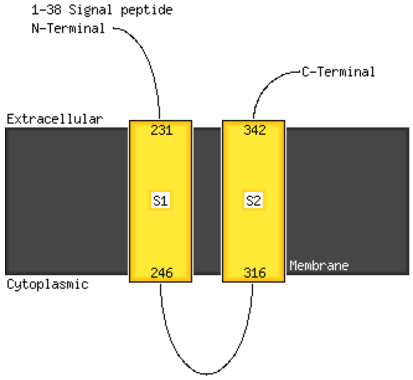
TaAOX1c-6DL	100	62	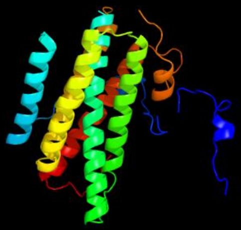	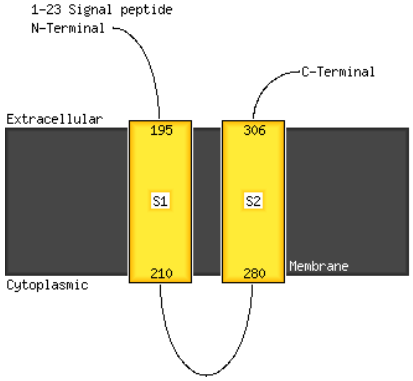
regTaAOX-3B	100	96	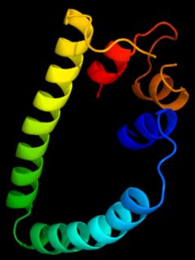	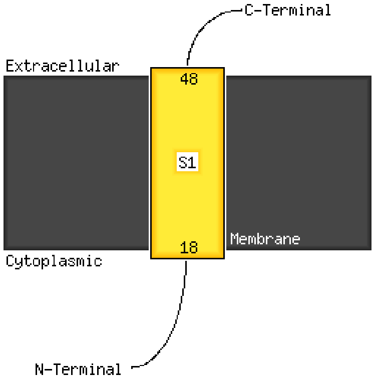
put.TaAOX1e-3DS	100	90	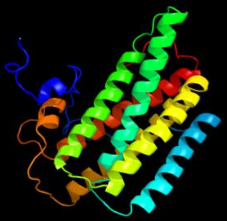	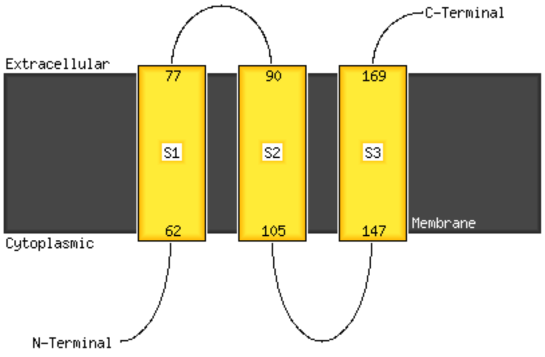

TaAOX1d-2AL.1	100	83	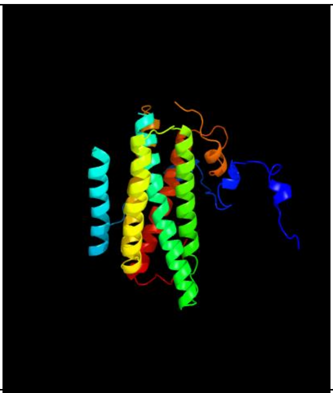	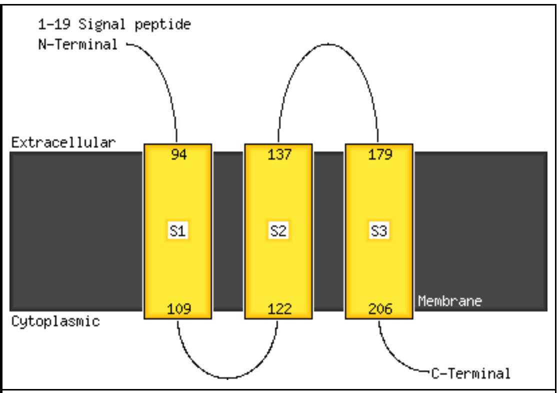
TaAOX1d-2AL.2.sv1	100	72	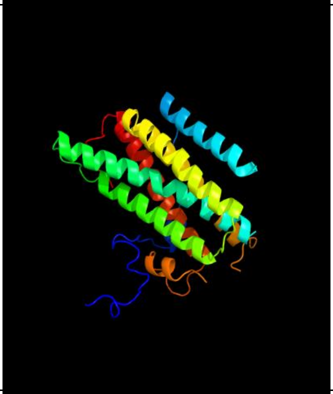	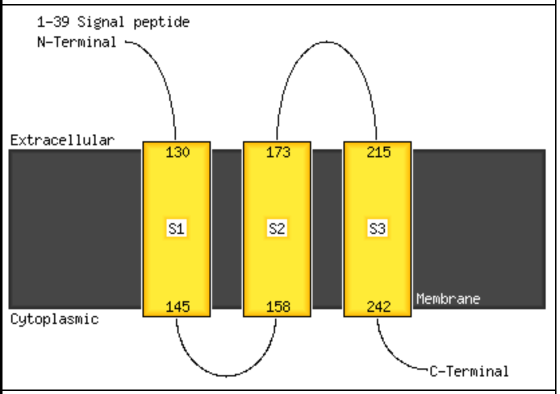
TaAOX1d-2AL.2.sv2	100	72	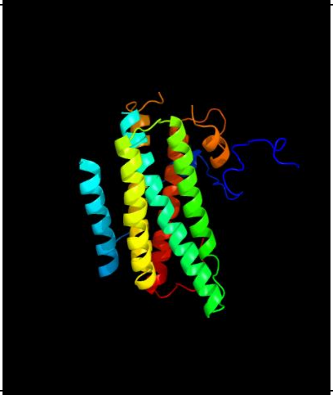	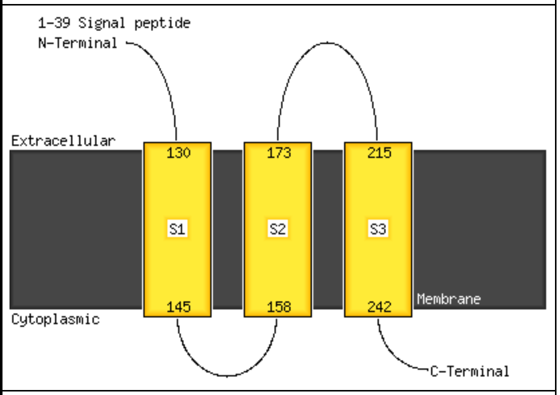
TaAOX1d-2DL	100	72	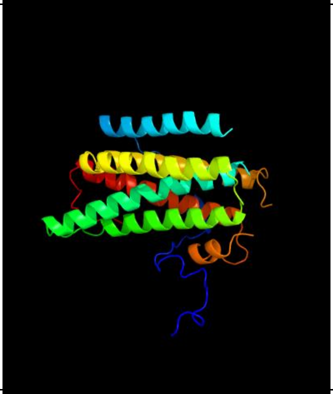	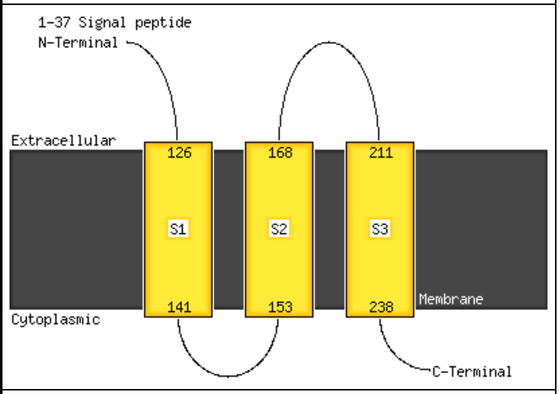
put.TaAOX1d-like-4AS	100	86	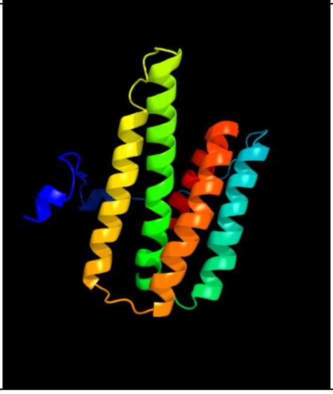	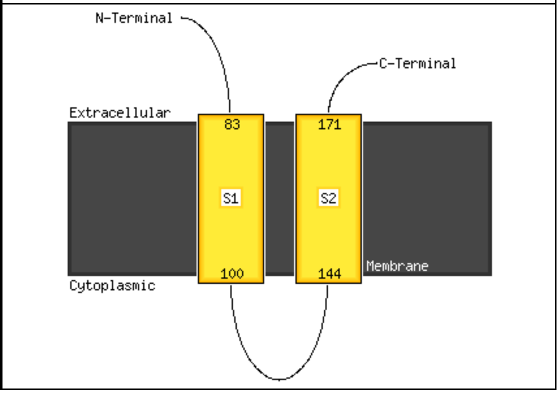
