# Supplementary material for: Genome-wide identification and analysis of the ALTERNATIVE OXIDASE gene family in diploid and hexaploid wheat
Source: PLoS One. 2018 Aug 3;13(8):e0201439. doi: 10.1371/journal.pone.0201439 (PMC6075773; doi:10.1371/journal.pone.0201439)
Supplement: S9 Table — The model used was c3vvaD. (PDF) [file pone.0201439.s018.pdf]

S9 Table. Summary of diploid AOX 3-D structures (TuAOX and AetAOX) obtained with Phyre2. The model used was c3vvaD.

Protein Name	Confidence	Alignment Coverage %	3D Image	Membrane Topology
TuAOX1a	100	97	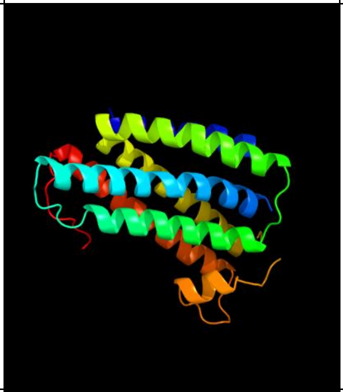	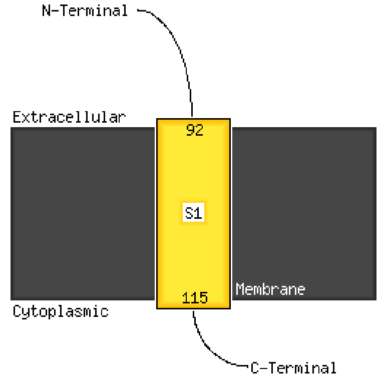
TuAOX1c	100	48	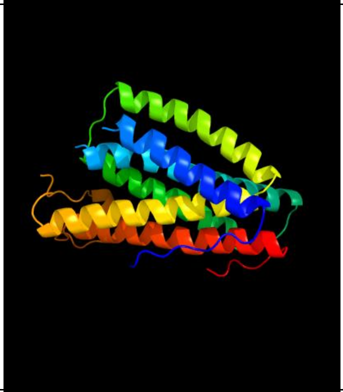	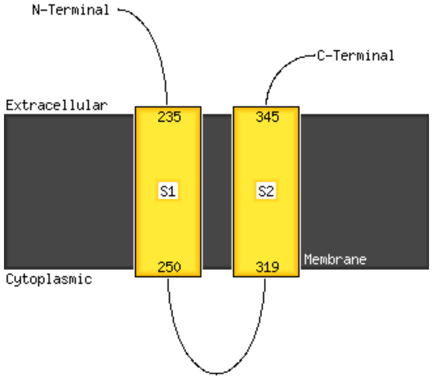
TuAOX1d.1	100	88	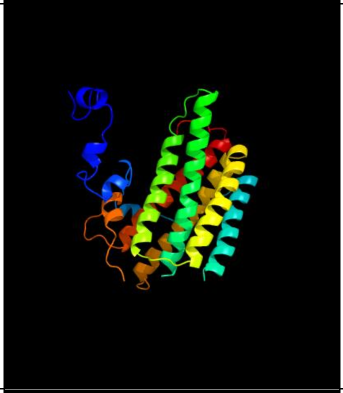	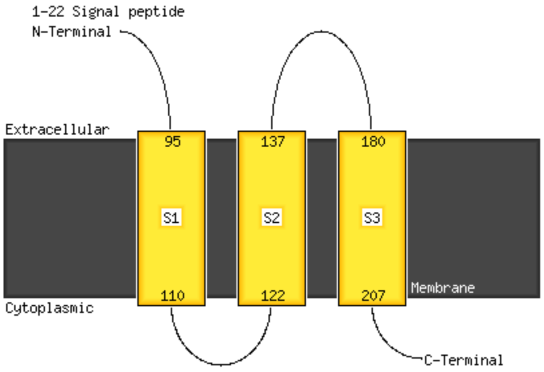
TuAOX1d.2	100	58	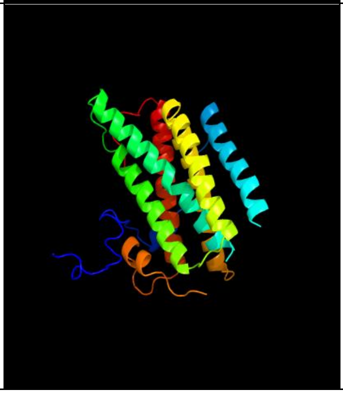	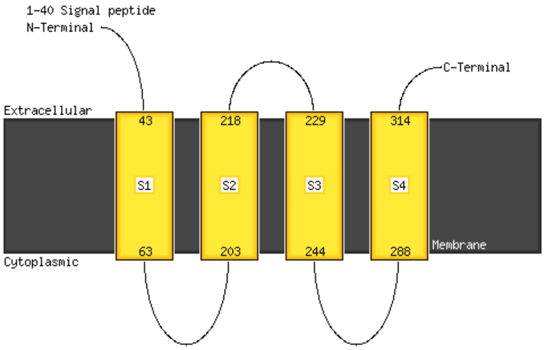

AetAOX1a	100	97	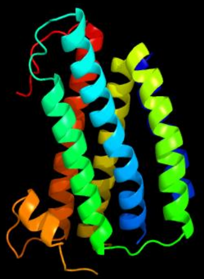	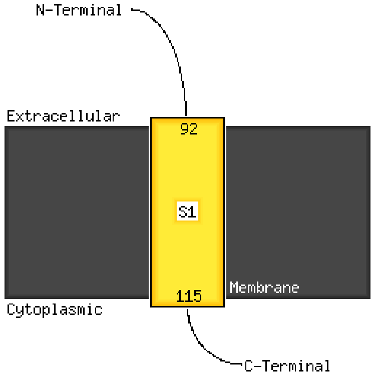
AetAOX1e	100	66	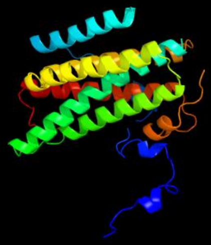	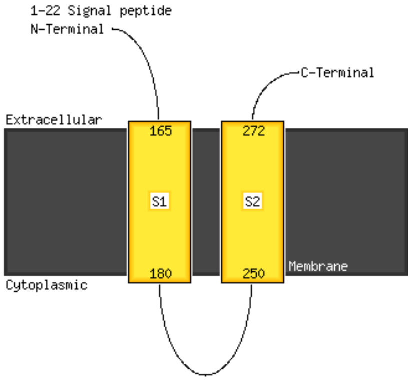
AetAOX1d	100	88	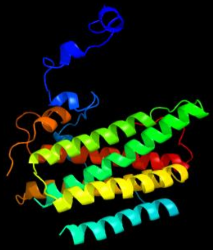	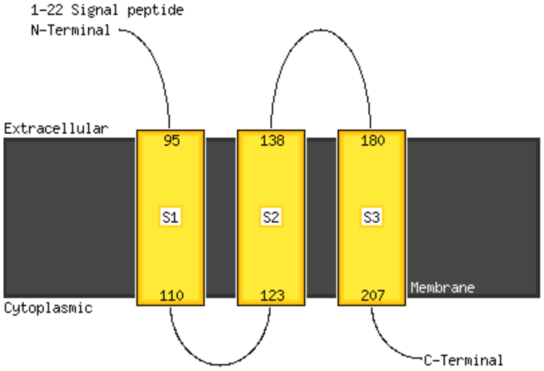
AetAOX1d-like	100	63	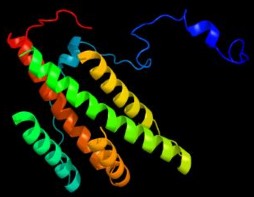	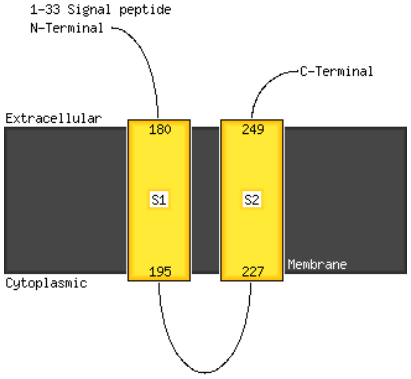
