# Supplementary material for: Genome-wide identification and analysis of the ALTERNATIVE OXIDASE gene family in diploid and hexaploid wheat
Source: PLoS One. 2018 Aug 3;13(8):e0201439. doi: 10.1371/journal.pone.0201439 (PMC6075773; doi:10.1371/journal.pone.0201439)
Supplement: S10 Table — *Denotes diploid AOX proteins. (PDF) [file pone.0201439.s019.pdf]

S10 Table. Comparison of active site diiron residues between TbAOX and wheat AOX.
*Denotes diploid AOX proteins.

Protein Name	Active Site Residues					
TbAOX	Glu123	Glu162	His165	Glu213	Glu266	His269
TaAOX1a-2AL.sv1	Glu157	Glu196	His199	Glu247	Glu298	His301
TaAOX1a-2AL.sv2	Glu149	Glu188	His191	Glu239	Glu290	His293
TaAOX1a-2BL	Glu286	Glu325	His328	Glu376	Glu427	His430
TaAOX1a-2DL.sv1	Glu165	Glu204	His207	Glu255	Glu306	His309
TaAOX1a-2DL.sv2	Glu123	Glu162	His165	Glu213	Glu264	His267
TaAOX1a-like-2DL	N/A	Glu32	His35	Glu83	Glu134	His137
put.TaAOX1e-3DS	Glu91	Glu130	His133	Glu181	Glu232	His235
TaAOX1c-6AL	Glu226	Glu265	His268	Glu316	Glu367	His370
TaAOX1c-6BL.sv1	Glu260	Glu299	His302	Glu350	Glu401	His404
TaAOX1c-6BL.sv2	Glu241	Glu280	His283	Glu331	Glu382	His385
TaAOX1c-6BL.sv3	Glu260	Glu299	His302	Glu350	Glu401	His404
TaAOX1c-6DL	Glu224	Glu263	His266	Glu314	Glu365	His368
TaAOX1d-2AL	Glu123	Glu162	His165	Glu213	Glu264	His267
TaAOX1d-2AL.2.sv1	Glu159	Glu198	His201	Glu249	Glu300	His303
TaAOX1d-2AL.2.sv2	Glu159	Glu198	His201	Glu249	Glu300	His303
TaAOX1d-2DL	Glu155	Glu194	His197	Glu245	Glu296	His299
put.TaAOX1d-like-4AS	Glu88	Glu130	His133	Glu181	Glu232	His235
TuAOX1a*	Glu33	Glu72	His75	Glu123	Glu174	His177
TuAOX1c*	Glu263	Glu302	His305	Glu353	Glu404	His407
TuAOX1d.1*	Glu124	Glu163	His166	Glu214	Glu265	His268
TuAOX1d.2*	Glu232	Glu271	His274	Glu322	Glu373	His376
AetAOX1a*	Glu33	Glu72	His75	Glu123	Glu174	His177
AetAOX1e*	Glu194	Glu233	His236	Glu284	Glu335	His338
AetAOX1d*1	Glu124	Glu163	His166	Glu214	Glu265	His268
AetAOX1d-like*	Glu183	Glu222	His225	N/A	Glu259	His262
