# Supplementary material for: Genome-wide identification and analysis of the ALTERNATIVE OXIDASE gene family in diploid and hexaploid wheat
Source: PLoS One. 2018 Aug 3;13(8):e0201439. doi: 10.1371/journal.pone.0201439 (PMC6075773; doi:10.1371/journal.pone.0201439)
Supplement: S11 Table — The residues that are not highlighted are from Moore et al. 2013; red highlights are residues from Shiba et al. 2013; green highlights are residues from Young et al. 2014 and Crichton et al. 2010. (PDF) [file pone.0201439.s020.pdf]

S11 Table. Critical TbAOX residues known to cause reduction in activity when mutagenized. The residues that are not highlighted are from Moore *et al.* 2013; red highlights are residues from Shiba *et al*. 2013; green highlights are residues from Young *et al.* 2014 and Crichton *et al.* 2010.

TbAOX numbering	Role	% Reduction in Activity
**R96**	Interaction with hydroxyl of quinol	76
**D100**	Stabilizing active site	69
**R118**	Inhibitor binding, Interaction with hydroxyl of quinol	99.8
**L122**	Substrate-binding channels 1 and 2	61
**E123**	Fe-Fe ligand; substrate-binding channels 1 and 2	100
**T124**	Stabilizing iron-ligating residues	72
**H138**	Membrane-binding region/dimer interface	62
**W151**	Hydrophobic interaction with helix α6	95
**E158**	Substrate-binding channels 1 and 2	90
**N161**	Secondary ligation sphere; hydrogen bond network	90
**E162**	Fe-Fe ligand	100
**H165**	Fe-Fe ligand	100
**Q187**	Dimer interface	94
**Y198**	Dimer interface; hydrogen bonds to His-206	53
**H206**	Membrane-binding region	98
**L212**	Hydrophobic cavity	59
**E213**	Fe-Fe ligand	100
**E215**	Inhibitor binding, Stabilize active site	97.5
**A216**	Substrate-binding channel 2	92
**T219**	Inhibitor binding, Interaction with hydroxyl of quinol	99.5
**Y220**	Catalytic cycle	100
**Y246**	Stabilizing the structure, diiron hydrogen network	95
**E266**	Fe-Fe ligand	100
**H269**	Fe-Fe ligand	100
