# Supplementary material for: Genome-wide identification and analysis of the ALTERNATIVE OXIDASE gene family in diploid and hexaploid wheat
Source: PLoS One. 2018 Aug 3;13(8):e0201439. doi: 10.1371/journal.pone.0201439 (PMC6075773; doi:10.1371/journal.pone.0201439)
Supplement: S13 Table — (PDF) [file pone.0201439.s022.pdf]

S13 Table. Summary of wheat AOX residues in dimerization domain.

Residues Conserved with TbAOX	H138, L142, R143, R163, L166, Q187^, M131, L139, S141, A159, M167, R180^
Unique to D and a-like subclassifications	M135V, R147H, L156M
Conserved within all subclassifications in wheat only	M145F, I183V^

^-except where nonexistent in a 'like'
