# Supplementary material for: Genome-wide identification and analysis of the ALTERNATIVE OXIDASE gene family in diploid and hexaploid wheat
Source: PLoS One. 2018 Aug 3;13(8):e0201439. doi: 10.1371/journal.pone.0201439 (PMC6075773; doi:10.1371/journal.pone.0201439)
Supplement: S14 Table — (PDF) [file pone.0201439.s023.pdf]

S14 Table. Summary of wheat AOX residues in hydrophobic cavity.

Residues Conserved with TbAOX	F102^, L122^, V125^, A126^, V128^, Y198, S201, V209^, L212^
Unique to all D subclassifications	S117&, R118H, F121L, P178W^, F208&
Unique to D subclassification Group 1	S91I, T94&~, L98G~, F99S, T186&
Unique to D subclassification Group 2	S91V, F99R, T186A
Unique to E subclassification	T94A, C95M or C95I, W97&, A197T, I200A, F204V, F208M
Conserved within all subclassifications in wheat only	L179E^, V181A^, S182L^, I189V, M190F, F193A, L194Y, V205A^

&-matches TbAOX
^-except where nonexistent in a 'like'
~-D group 2 residues at this location matches those of A/C/E
